# The nature of the GRE influences the screening for GR-activity enhancing modulators

**DOI:** 10.1371/journal.pone.0181101

**Published:** 2017-07-07

**Authors:** Karen Dendoncker, Steven Timmermans, Kelly Van Looveren, Lode De Cauwer, Karolien De Bosscher, Claude Libert

**Affiliations:** 1 Mouse Genetics in Inflammation, VIB Center for Inflammation Research, Ghent, Belgium; 2 Department of Biomedical Molecular Biology, Ghent University, Ghent, Belgium; 3 Medical Biotechnology Center, VIB, Ghent, Belgium; 4 Department of Biochemistry, Ghent University, Ghent, Belgium; Wayne State University, UNITED STATES

## Abstract

Glucocorticoid resistance (GCR), *i*.*e*. unresponsiveness to the beneficial anti-inflammatory activities of the glucocorticoid receptor (GR), poses a serious problem in the treatment of inflammatory diseases. One possible solution to try and overcome GCR, is to identify molecules that prevent or revert GCR by hyper-stimulating the biological activity of the GR. To this purpose, we screened for compounds that potentiate the dexamethasone (Dex)-induced transcriptional activity of GR. To monitor GR transcriptional activity, the screen was performed using the lung epithelial cell line A549 in which a glucocorticoid responsive element (GRE) coupled to a luciferase reporter gene construct was stably integrated. Histone deacetylase inhibitors (HDACi) such as Vorinostat and Belinostat are two broad-spectrum HDACi that strongly increased the Dex-induced luciferase expression in our screening system. In sharp contrast herewith, results from a genome-wide transcriptome analysis of Dex-induced transcripts using RNAseq, revealed that Belinostat impairs the ability of GR to transactivate target genes. The stimulatory effect of Belinostat in the luciferase screen further depends on the nature of the reporter construct. In conclusion, a profound discrepancy was observed between HDACi effects on two different synthetic promoter-luciferase reporter systems. The favorable effect of HDACi on gene expression should be evaluated with care, when considering them as potential therapeutic agents. GEO accession number GSE96649.

## Introduction

Glucocorticoids (GC) are a most effective therapy for the treatment of many inflammatory disorders, such as asthma, rheumatoid arthritis and inflammatory bowel disease [1–3]. Glucocorticoid actions are mediated by the glucocorticoid receptor (GR), a nuclear receptor. After ligand binding, GR translocates to the nucleus where it performs functions as a monomer or as a homodimer. By and large, the homodimer GR binds glucocorticoid responsive elements (GRE) to activate gene expression while monomer GR interacts with pro-inflammatory transcription factors (NFκB and AP-1) to mediate gene repression [4]. Recent studies suggest that besides GR monomers, also GR homodimers hold strong anti-inflammatory effects [5, 6], via the induction of genes coding for anti-inflammatory proteins, such as MAPK phosphatase 1 (MKP1) [7] and GILZ [8].

The therapeutic success of GCs is limited due to two main drawbacks. High doses and/or prolonged administration of synthetic GCs often result in side-effects, including glaucoma, type 2 diabetes, growth retardation and skin thinning [9, 10]. Moreover, the occurrence of GC irresponsiveness referred to as glucocorticoid resistance (GCR) hampers the success of GC-based therapies [11, 12].

We have previously described that the inflammatory cytokine tumor necrosis factor (TNF), both *in vivo* as well as *in vitro* using cell lines, induces a status of GCR [13]. We reasoned that a screening for compounds that prevent or reverse GCR or that increase the transcriptional capacity of GR dimers, may be beneficial for more efficient GR-based therapies [14, 15].

The access of transcription factors to gene promoters is regulated by epigenetic changes. Histone acetyl transferases (HATs) coordinate the recruitment and activation of transcription factors by acetylation of the histone tails, leading to unwinding of the chromatin and gene promoter exposure. In their turn, histone deacetylases (HDACs) counteract the HAT activity, which makes epigenetic changes highly dynamic and reversible [16–20]. Histones are not the only targets of HDACs. Recent proteomics reports have shown that also non-histone proteins, such as transcription factors, can be deacetylated by HDACs [21, 22]. This duality makes that HDACs play multiple roles [23, 24] in the complex regulation of gene expression and signal transduction [25–27], and regulate multiple biological processes such as cell differentiation, survival and cell cycle progression [28].

Epigenetic changes are reversible and can be targeted by small molecules, such as HDAC inhibitors (HDACi). Extensive research into novel HDACi [29] has resulted in series of diverse modulators, each with their own target specificity and potency [30–32]. Originally HDACi were used to treat solid and haematological cancers [33–36]. Nowadays, HDACi are used far beyond the cancer field to treat, amongst others, neurodegenerative diseases [37, 38] and inflammatory disorders [39, 40] such as rheumatoid arthritis [41], lupus erythematosus [42] and type 2 diabetes [43]. So far, four HDACi, namely Vorinostat (SAHA), Romidepsin, Panobinostat and Belinostat, are approved by the United States Food and Drug Administration (FDA). SAHA was the first approved drug in 2006 for the treatment of advanced cutaneous T-cell lymphoma [44]. Vorinostat, along with Belinostat, approved for the treatment of patients with relapsed or refractory T-cell lymphoma [45], are two pan-HDACi which can impair the activity of both class I and II deacetylases [46].

Sepsis is an acute systemic inflammatory disease, which can be considered as a GC resistant disease [47–50]. SAHA improves the survival in two rodent models of sepsis, namely lipopolysaccharide (LPS)-induced endotoxic shock [51] and the more clinical relevant model of cecal ligation and puncture (CLP)-induced septic shock [52]. Although, so far these data are not linked with the restoration of GR sensitivity, some HDACs influence the GR activity. HDAC6 for instance, acetylates Hsp90 and directly regulates the chaperone-mediated GR activation [53]. Furthermore, GR deacetylation by HDAC2 is required for NFκB-mediated inflammatory gene repression [54] and the acetylation status of HDAC1 dynamically modulates GR-induced gene transcription [55].

We investigated the ability of 37 chemical compounds, including SAHA, MTA (histone methylase inhibitor) and CPTH2 (HAT inhibitor) to increase the transcriptional activity of GR in a luciferase-based reporter assay i.e. GRE-luc stably integrated in lung epithelial A549 cells which contain the GR protein endogenously. Increased luciferase activity following enhanced expression levels, resulting from activated GR binding to glucocorticoid responsive elements (GREs), is a direct measure of the transcriptional activity of GR. We found that Vorinostat as well as Belinostat strongly induced the GR transcriptional activity as judged by a GRE-luciferase reporter system. However, whole transcriptome analysis revealed that Belinostat in fact strongly impaired the transcriptional activity of GR, confirming the recent work of Kadiyala [56]. After studying another GRE-driven reporter construct, i.e. the MMTV-luciferase reporter, we must conclude that, at least for the screening purposes of HDACi, results obtained solely from the GRE-luciferase screening system must be interpreted with caution.

## Materials and methods

### Cell culture

A549 cells (lung epithelial cells) were maintained and grown in Dulbecco’s modified Eagle’s medium (DMEM; house-made) containing 10% fetal calf serum (FCS), 1mM sodium pyruvate, 0,1mM non-essential amino acids, and 2mM L-glutamine.

### Reagents and plasmids

The GRE-luc plasmid was described previously [57]. Briefly, the luciferase reporter construct was driven by a synthetic GR-responsive promoter region containing two classic consensus GRE sequences (underlined) derived from the tyrosine aminotransferase (TAT) gene promoter AGATCTCTCTGCTGTACAGGATGTTCTAGCGGATCCTGCTGTACAGGATGTTCTAGCTACCTGCAG succeeded by a minimal IL6 promoter TATA box and followed by the luciferase gene, of which the quantified luciferase expression is a direct measure of GR transcriptional activity. The MMTV-luc (pGL4.36 [luc2P/MMTV/Hygro] Vector) plasmid was purchased from Promega. SAHA (Vorinostat, SML0061) was obtained from Sigma Aldrich and Belinostat (PXD101, S1085), CI994, Class I HDACi (Tacedinaline, S2818) and Abexinostat, pan HDACi, mostly targeting HDAC1 (PCI-24781) were purchased from Selleckchem. For all compounds, a stock dilution of 10^-2^M was prepared in dimethylsulfoxide (DMSO) and stored in -20°C. Dexamethasone (Dex, D-2915) was purchased from Sigma Aldrich and dissolved in water. Recombinant human TNF was produced in *E*. *coli* and purified in our department.

### Transfection and reporter assays

X-tremeGENE HP DNA transfection reagent from Roche was used to transfect the A549 cells in 24-wells plate according to manufacturer’s instructions. Six hours after transfection medium was changed to Optimem Medium (Gibco, Invitrogen) and cells were exposed to HDACi. After the indicated duration, cells were stimulated with 10^-6^M Dex for five hours. Cells were then harvested and luciferase activity, expressed in arbitrary light units, was quantified with the Glomax instrument, measuring the D-luciferin (L-1349, Duchefa) conversion. For transiently transfected cells, luciferase activity was corrected for the protein concentration in the sample by normalization to constitutive β-Gal levels. β-Gal levels were quantified with a chemiluminescent reporter assay, using CPRG substrate (Sigma Aldrich).

### RNA isolation and quantitative PCR

Total RNA was isolated from A549 cells using TRIzol (Gibco, Life Technologies) and the InviTrap Spin Universal RNA Mini Kit (Invitek, Isogen Life Science) according to the manufacturer’s instructions. RNA concentration as measured with Nanodrop (Thermo Scientific), and 1μg of RNA was used to prepare cDNA by reverse transcription with the iScript advanced cDNA synthesis kit (Bio-Rad). qPCR was performed with SYBR Green Master Mix (Bioline) using the Roch LightCycler 480 system (Applied Biosystems). Luciferase mRNA was detected by RT-qPCR using the forward primer: 5’-ATACAAAGGATATCAGGTGG- 3’ and the reverse primer: 5’- TTGCGTCGAGTTTTCCGG-3’. Results are given as relative expression values normalized to the geometric mean of the housekeeping genes 36B4 and Cyclophilin. The stability of these housekeeping genes in the appropriate experimental model and condition was verified using the geNorm algorithm [58].

### RNA sequencing

A549 cells were seeded in 6-well plates (0,5 x 10^6^ cells/well) in DMEM. Cells were exposed to Belinostat (1μM) equal dilution of DMSO, 16 hours before Dex (10^-6^M, five hours) or PBS stimulation. Total RNA was isolated using TRIzol (Gibco Life Technologies) and the InviTrap Spin Universal RNA Mini Kit (Invitek, Isogen Life Science) according to the manufacturer’s instructions. RNA concentration was measured and quality was checked with the Agilent RNA 6000 Pico Kit (Agilent Technologies Belgium), and 5μg of RNA was sent to the Nucleomics core facility of VIB (http://www.nucleomics.be/) for sequencing on an Illumina Genome Analyzer. Library preparation was performed according to the Illumina truseq RNA stranded library protocol. The library was subjected to single end sequencing. Raw sequencing data underwent a quality control analysis with FastQC [59]. To avoid low quality data the reads were trimmed and filtered with Trimmomatic (version 0.33) [60]. Quality checked data were mapped to the human reference genome (hg38; University of California, Santa Cruz) with Tophat2 [61]. Read counts at the gene level were obtained with the Python tool htseq-count [62] and differential gene expression was obtained using DESeq2. We used a false discovery rate (FDR) of 1% and a log_2_ fold change |LFC| of 1 for comparisons to the control condition. To detect subtler changes between Dex and Belinostat (single and co-treatment) the LFC cut-off was lowered to 0.5 for the direct comparisons between these conditions (FDR was kept at 1%). RNAseq data are deposited under accession number GSE96649.

### Statistical analysis

**Fig 1:**
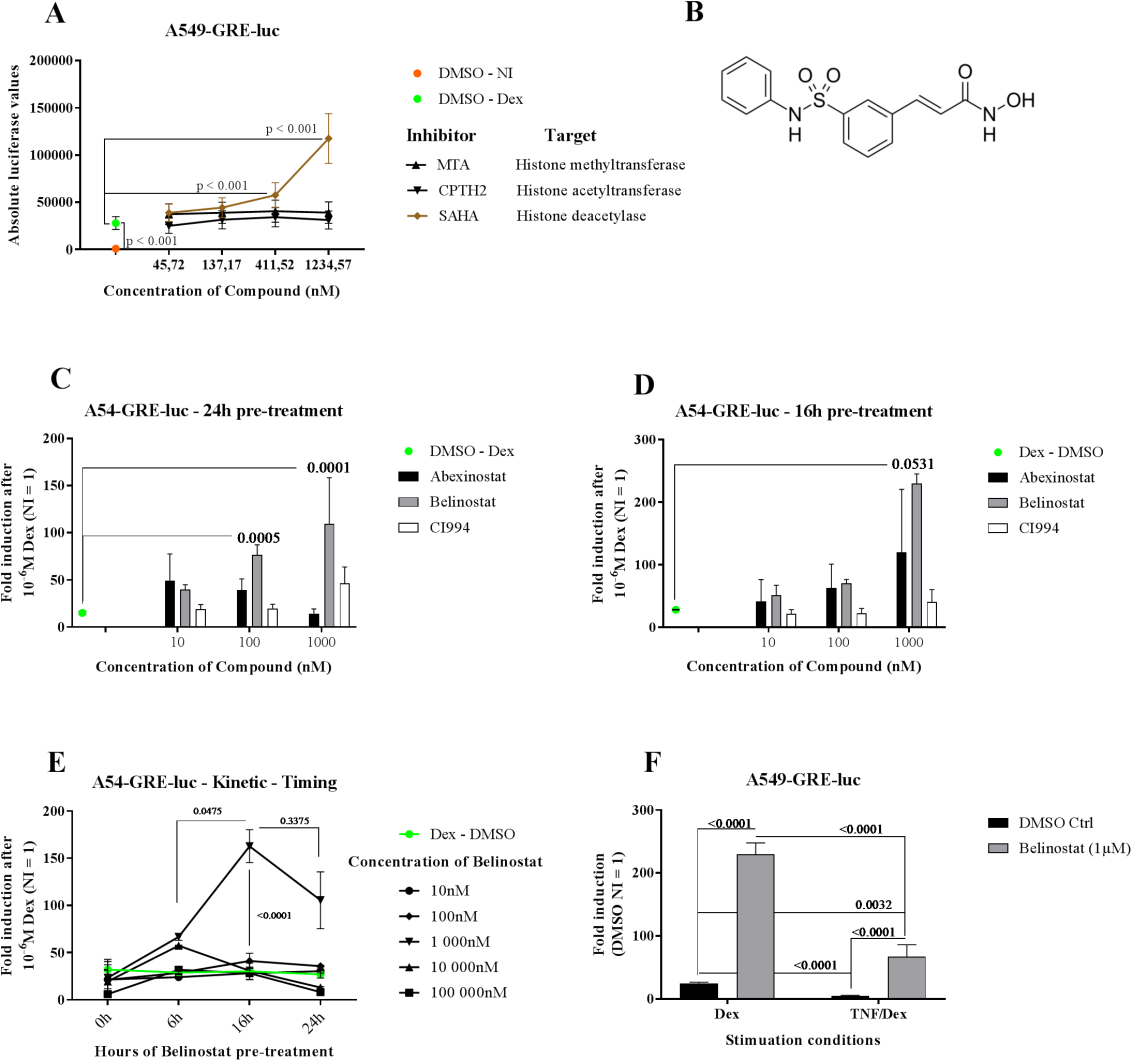
Belinostat strongly induces GRE-luciferase expression. (**A**) A549 cells, stably transfected with the GRE-luciferase plasmid, were exposed to several inhibitors at different concentrations, 24h before Dex stimulation (10^-6^M for 5h). Data are shown as mean ± SEM (n = 2). A Generalized Linear Mixed Model has been fitted to the luciferase values of the three compounds jointly obtained from two independent experiments, with compound–concentration set as fixed factor, and experiment as random term. T statistics were used to assess the significance of all compound–concentration combination effects estimated as differences (on the log-transformed scale) to the Dex-DMSO control (green dot). (**B**) Chemical structure of Belinostat (**C-D**) Cells were treated with three different HDAC inhibitors 24h before (C) and 16h before (D) Dex stimulation and luciferase was measured 5h after Dex stimulation. Data are shown as mean ± SEM (n = 3). One-way ANOVA of log (Y) transformed fold inductions (non-induced (NI) = 1, red dot) followed by Dunnett’s multiple comparisons test were applied to compare each concentration of compound to the DMSO (Dex only) control (green dot). P-values are depicted above the bars. (**E**) A549 cells were treated with different concentrations of Belinostat 24h, 16h and 6h before Dex (10^-6^M, 5h) stimulation or together with Dex (0h pre-treatment). Fold inductions were obtained by dividing the compound condition by the proper non-induced, DMSO treated condition per time point. Two-way ANOVA of log (Y) transformed fold inductions in combination with the Tukey test was performed to obtain statistical p-values (n = 4). (F) 16h before TNF treatment, A549-GRE-luc cells were stimulated with 1μM Belinostat. GR activity was induced by 10^-6^M Dex given 1h after TNF. Significant differences in Log (Y) transformed fold inductions were tested with two-way ANOVA and subsequently Sidak’s multiple comparisons test (n = 4). The obtained p-values are reported.

Except for Fig 1A, data were expressed as means ± standard errors of the means (SEM). Fold changes were calculated by dividing the compound conditions by the proper DMSO control condition and log (Y) transformation was applied before statistical analysis. Two-way ANOVA was performed to test whether there was a difference between differential treatments. Subsequently, pairwise multiple comparisons based on the Tukey’s procedure was followed for post hoc analysis to identify the differential compound/treatment. Obtained p-values are depicted above the data points in the graphs.

For Fig 1A, a Generalized Linear Mixed Model (GLMM; fixed model: poisson distribution, log link; random model: gamma distribution, log link) as implemented in Genstat v18 [63] has been fitted to the absolute luciferase values of three compounds jointly, with compound–concentration set as fixed factor, and experiment as random term. T statistics were used to assess the significance of all compound–concentration combination effects estimated as differences (on the log-transformed scale) to the Dex-DMSO control.

## Results

### Belinostat strongly induces GRE-luciferase expression

The success rate of glucocorticoid therapy as a treatment for inflammatory disorders is limited by side-effects and GCR [64, 65]. Screening for chemical compounds that ameliorate the GR transcriptional output could be of great interest to circumvent both the side-effects and GCR. Using stably transfected A549 human lung epithelial cells, we developed a GRE-luciferase reporter-based assay that measures the GR transcriptional activity and allows evaluating compounds, at different concentrations and time points. In a first orienting screen, we tested a small set of compounds, including inhibitors of several enzymes. A549-GRE-luc cells were treated with a dilution series of compounds or DMSO, 24h prior to Dex stimulation (10^-6^M for 5h). One compound, SAHA, known as a broad-spectrum HDAC inhibitor [66] strongly augmented the Dex-stimulated and GRE-driven luciferase signal (Fig 1A), while other compounds, e.g. the histone methylase inhibitor MTA and the HAT inhibitor, CPTH2, had no effect. We next explored the effect of additional HDAC inhibitors in the assay, namely the pan-HDAC inhibitors Abexinostat and Belinostat and the HDAC class I-specific CI994. A549-GRE-luc cells were exposed to these inhibitors 24h as well as 16h prior to Dex treatment (Fig 1C and 1D respectively). Dex-induced luciferase expression was divided by the non-induced expression to obtain the fold induction. The impact of the compounds on the fold induction is displayed. The effect of Belinostat alone on the luciferase expression was not tested in the first screening in Fig 1. However, as will be clear later on in the paper, Belinostat already induces the GRE-luc system in the absence of Dex especially when cells are pre-treated for a long time. The maximal GR transcriptional activity was observed after 16h pre-treatment of inhibitor. With a 4.1 fold higher luc signal compared to Dex-DMSO, the broad spectrum HDAC inhibitor Belinostat (structure see Fig 1B) was selected for further follow-up. Belinostat also has the additional advantage of being an FDA approved drug. Based on concentration-response and kinetics studies (see Fig 1E), we found that pre-incubation of 16h of 1μM Belinostat was the optimal condition to proceed. Next, the potential of this Belinostat treatment was tested in a TNF-induced glucocorticoid resistance model. When A549-GRE-luc cells are treated with TNF, 1h before Dex stimulation, the GR transcriptional activity is strongly reduced (Fig 1F). This result correlates with the findings of Van Bogaert *et all*. who demonstrated GCR *in vivo* [67]. Interestingly, in the GCR condition, Belinostat pre-treatment induces the GR activity in such a way that the luciferase levels are significantly higher compared to the DMSO–Dex condition. Although, the GCR still occurs in presence of Belinostat, suggesting that Belinostat interferes with the GR activity, rather than with the mechanism that leads to TNF-induced GCR.

### Belinostat impairs the Dex-induced expression of GR target genes

To next study the impact of Belinostat on endogenous GR-induced (GRE-driven) genes, we performed a transcriptome profiling using RNA sequencing in A549 cells. The impact of a 5h incubation of 10^-6^M Dex on the transcriptome of A549 cells was significant, and numerous GRE genes were strongly induced, e.g. FKBP5 (21-fold induction) and SGK (4.89-fold induction). In total, 938 genes were strongly and significantly affected by Dex (S1 Table), i.e. with a |LFC| ≥ 1; FDR: 1% of which 610 were upregulated and 328 were downregulated by Dex. Belinostat by itself was found to already have a large effect on the gene expression profile: 5082 genes were significantly differentially expressed (S2 Table) (|LFC| ≥ 1; FDR: 1%) following a 16h Belinostat treatment at 1μM (2934 up, 2148 down).

**Fig 2:**
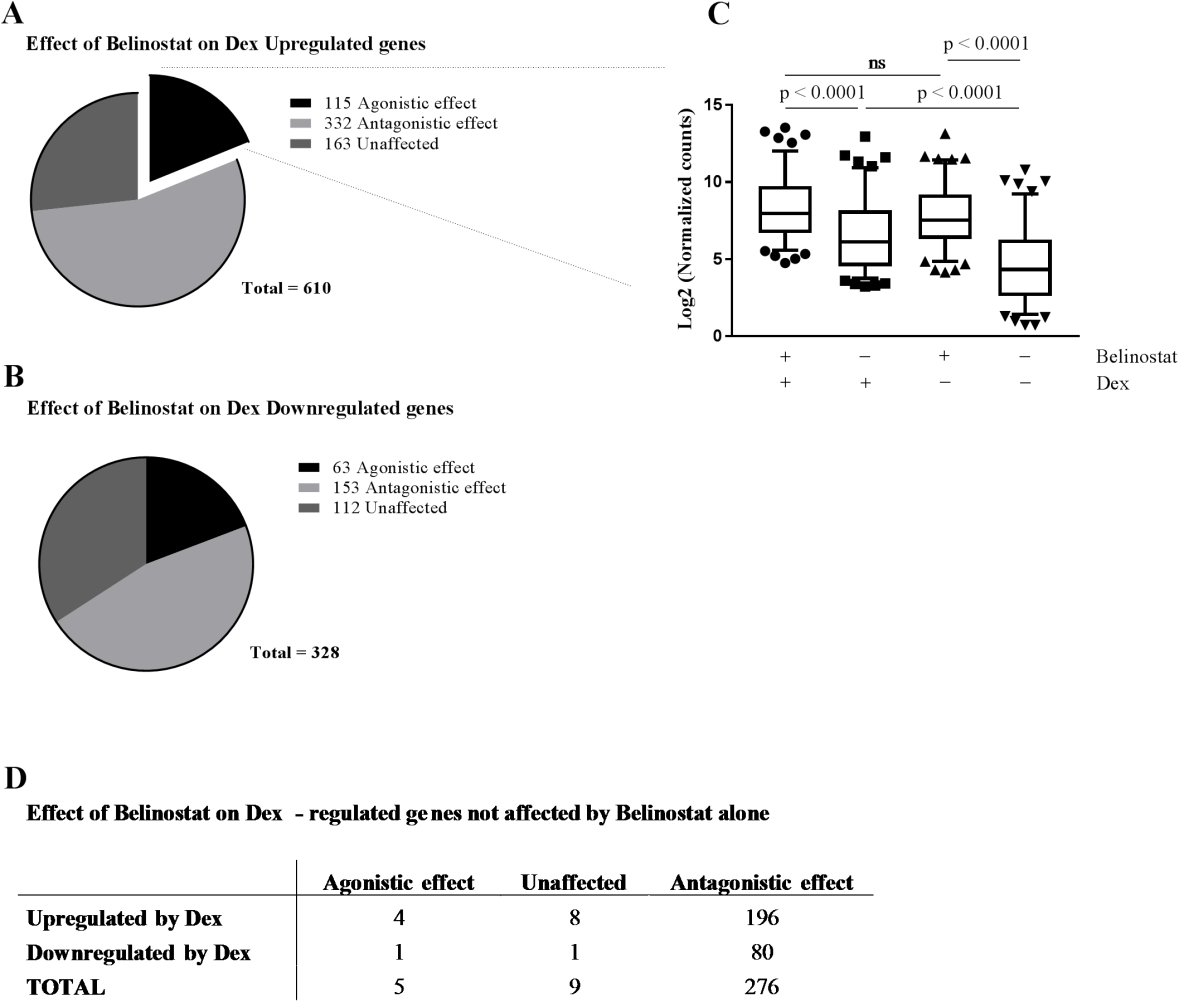
Belinostat impairs Dex-induced expression of GR target genes. A549 cells were exposed to Belinostat (1μM) or DMSO (same dilution) for 16h before Dex stimulation (10^-6^M, 5h) (n = 3). Data were obtained from an expression profiling performed by means of RNA-seq. (**A**) Effect of Belinostat pre-treatment on the expression of Dex-induced genes. The majority of genes are negatively affected (i.e. less upregulated) compared to Dex only treatment (LFC < -0.5; FDR: 1%; Dex and Belinostat vs Dex). Less than one fifth (18.85%) of all Dex-induced genes are positively affected by Belinostat (LFC > 0.5; FDR: 1%; Dex and Belinostat vs Dex). (**B**) Effect of Belinostat pre-treatment on the expression of Dex-inhibited genes. The majority of genes are negatively affected (i.e. less downregulated) compared to Dex only treatment. About one fifth (19.21%) of all Dex inhibited genes are more inhibited by Belinostat. (**C**) Box-and-whisker plots, with the box comprising the 25–75 percentile and the whiskers the 5–95 percentile range of the log2 transformed normalized counts from the 115 Dex-induced genes positively affected by Belinostat. One-way ANOVA of log2 transformed counts was applied for analysis of statistical significance. ns, not significant. Without any treatment, the median expression of these genes is the lowest. Either Dex or Belinostat alone can cause a significant upregulation of these genes. The co-treatment condition causes the median expression level to be significantly higher than what can be done by Dex alone, but there is no significant difference with the Belinostat only treatment, i.e. the Belinostat treatment is the dominant effect. (**D**) In order to exclude Belinostat only (or Belinostat dominant) genes that showed a significant effect of Belinostat only treatment, were removed from (A) and (B). When this correction is applied, only 5 Dex-regulated genes are positively affected by Belinostat (of 290), showing that the positive effect of Belinostat is greatly outweighed by its negative effect.

To validate the GRE-luciferase assay result on a genome-wide scale, we studied the influence of Belinostat and Dex co-treatment on the subset of Dex-regulated genes. Only a minority of these genes are higher expressed by Belinostat together with Dex, compared to Dex alone. Fig 2 shows that for both Dex-upregulated (A) and Dex-downregulated (B) genes, the subset of genes on which the effect of Dex is enhanced by Belinostat treatment is smaller than the group of genes on which Belinostat has no effect or counteracts the Dex effect. Out of the 610 Dex-upregulated genes (LFC ≥ 1; FDR: 1%) only 115, (18.8%), are further upregulated by Belinostat co-treatment (LFC ≥ 0.5; FDR: 1%). For downregulated genes, only 63 (19.2%) out of the 328 are affected by Belinostat in a positive way (i.e. stronger downregulation). The majority of the genes is adversely affected by the co-treatment compared to Dex alone: 332 Dex-upregulated genes (54.4%) and 153 Dex-downregulated genes (46.6%) are in this group. Because of the genome-wide effect of Belinostat alone, Belinostat affects also the expression of many GR-independent genes. In addition, in many of the cases where Belinostat exerts an agonistic effect, the effect is that of Belinostat alone: there is no significant difference between Belinostat alone and the co-treatment of Dex and Belinostat (Fig 2C). To exclude these Belinostat-only effects, we excluded genes that were differentially expressed by Belinostat alone, i.e. genes that are regulated independent of GR activity. After this filtering step, 290 Dex-regulated genes were left to consider (out of 938 genes differentially expressed by Dex) (Fig 2D). Hereof, the majority (276 genes) were adversely affected by Belinostat; nine genes showed no effect of Belinostat treatment and only 5 genes showed evidence of a positive influence by Belinostat on the effect mediated by Dex. We conclude that Belinostat treatment is clearly the dominant factor in the co-treatment and that there is little evidence for a co-operation between Dex and Belinostat treatment.

### No difference of Belinostat stimulation on stably and transiently transfected GRE-luc reporter

HATs and HDACs dynamically and reversible regulate the chromatin compaction along the genome and so HDACi are expected to have an impact on chromatin condensation and transcription factor accessibility to promoter regions. To exclude that the opposite findings between the luciferase assay and the RNAseq are caused by an effect of Belinostat on the region surrounding the GRE-luc integration site, rather than on GRE-luc itself, the impact of Belinostat on an exogenous GRE-luc plasmid was investigated.

**Fig 3:**
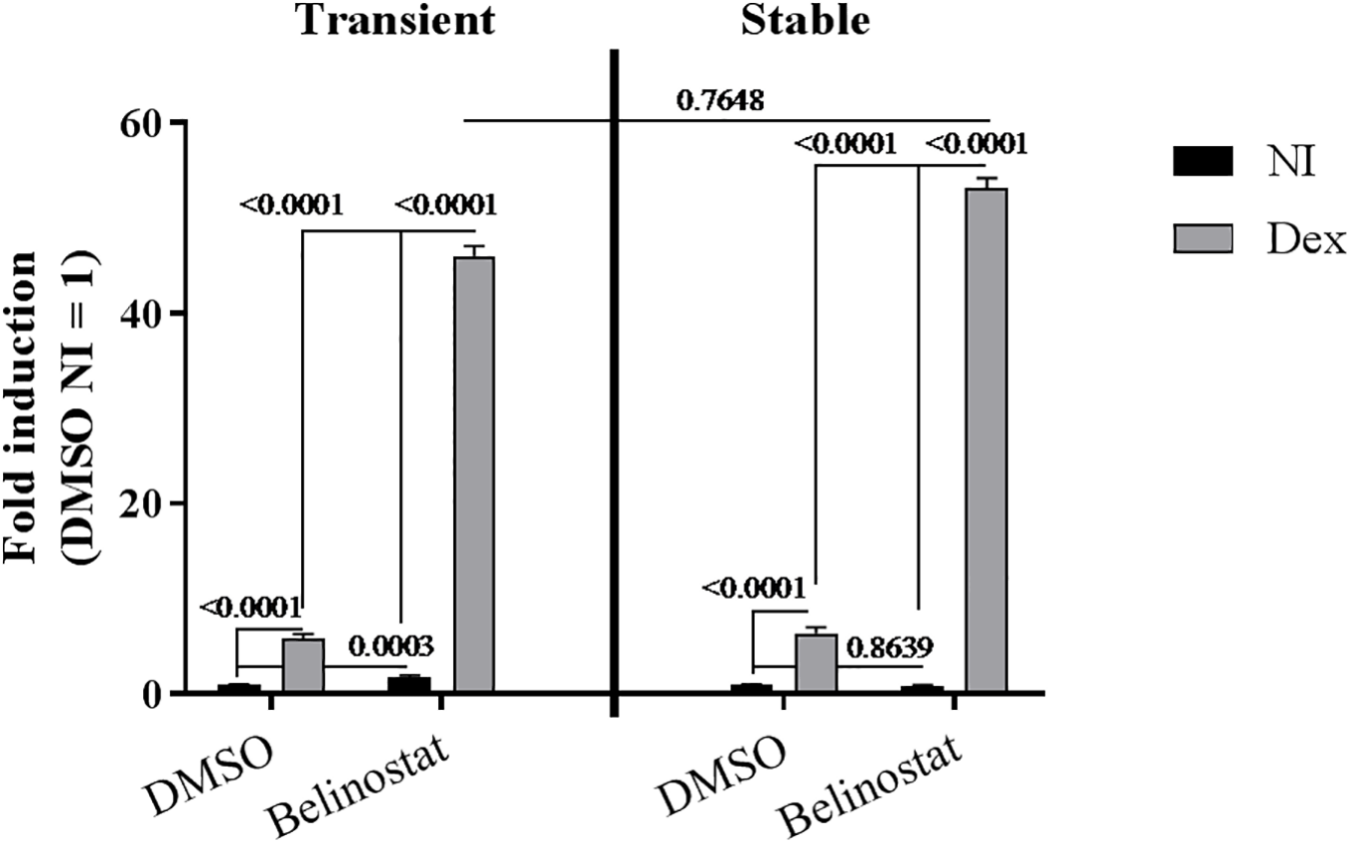
No difference of Belinostat stimulation on stably and transiently transfected GRE-luc reporter. A549 cells were transiently or stably transfected with the GRE-luciferase reporter plasmid, respectively left or right panel. Belinostat (1μM) or DMSO (same dilution) were exposed 16h before Dex stimulation (10^-6^M, 5h). The results are represented as fold inductions relative to the control (non-induced), mean ± SEM. Two-way ANOVA of log (Y) transformed fold inductions in combination with Tukey’s multiple comparisons test were applied for analysis of statistical significance, P-values are depicted (n = 3).

The effect of Belinostat on the luciferase expression of stably and transiently transfected reporter constructs was compared in Fig 3. Belinostat stimulated Dex-induced luciferase expression in stably as well as transiently transfected A549 cells, ruling out that the positive effect of Belinostat on the luciferase expression is influenced by the specific integration site of the plasmid in the stable cells.

### Belinostat has no direct impact on luciferase biological activity

**Fig 4:**
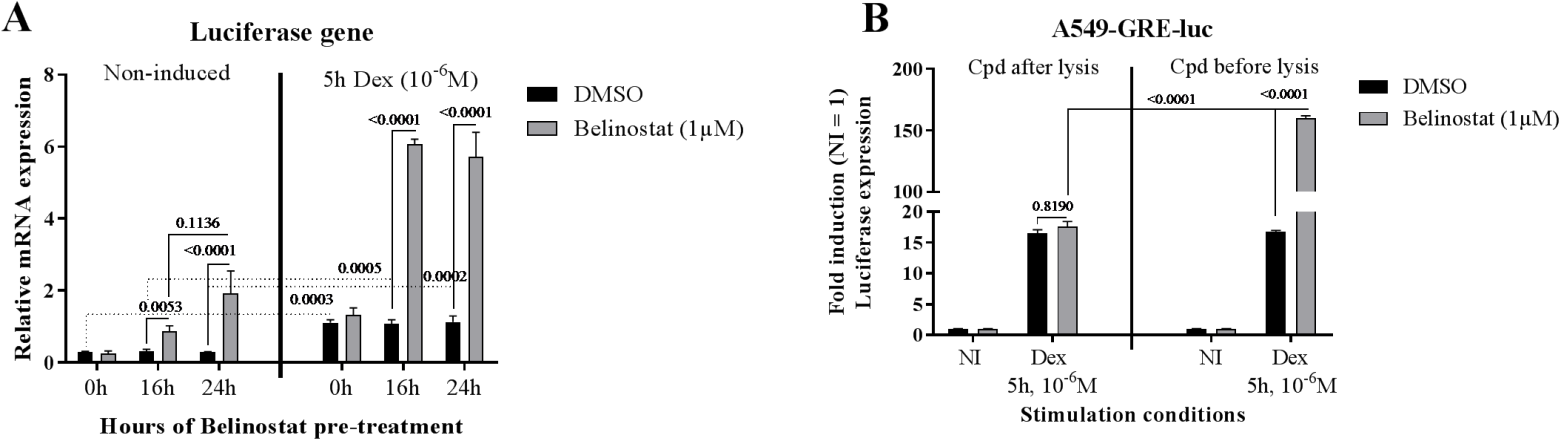
Belinostat does not influence the enzymatic activity of the luciferase protein. (**A**) A549 cells were exposed to Belinostat (1μM) or DMSO (same dilution) for 24h or 16h before Dex stimulation (10^-6^M, 5h) or together with Dex. Cells were harvested at 5h after Dex stimulation. RNA was isolated and subjected to RT-qPCR to measure luciferase mRNA expression. (**B**) After the five hours during Dex stimulation (10^-6^M, 5h), A549 cells were lysed in lysis buffer supplemented with 1μM Belinostat (left panel). In the right panel, Belinostat was added 16h before Dex stimulation (n = 3). Statistical significance in (A) and (B) was proven by two-way ANOVA and Tukey’s multiple comparison test on log (Y) transformed data.

The compound screening was performed in cells using a previously described luciferase reporter construct under influence of a synthetic GR-responsive promoter region with GRE sequences. These sequences were derived from the TAT promoter [57] driving the expression of the heterologous luciferase gene, of which the quantified luciferase expression is a direct measure of GR transcriptional activity. Reporter gene-based systems, depending on an enzymatic read-out, have to be considered with care, since the stability and activity of the gene product can be influenced by the treatment leading to potentially misleading conclusions. We measured luciferase mRNA levels after Belinostat treatment in A549-GRE-luc cells, to confirm that Belinostat modulates GR-mediated luciferase mRNA induction, not protein stability or activity. Cells were exposed to Belinostat either 24h or 16h prior to Dex, or simultaneously with Dex, and RNA was extracted from the cells five hours after Dex. The relative luciferase mRNA expression (Fig 4A) demonstrates that Belinostat induces luciferase expression at the mRNA level, illustrating the ability of Belinostat to activate transcription of the GRE-containing promoter. Remarkably, Belinostat was also able to induce luciferase mRNA expression independent of the presence of the GR ligand Dex. We believe that in absence of ligand, some leak expression is observed with Belinostat, due to a more open chromatin state. Indeed, inhibition of HDACs leads to an enhanced chromatin acetylation, which relax the chromatin and make it accessible for pioneering transcription factors. Furthermore, upon adding 1μM Belinostat to lysates of A549 cells, previously stimulated with Dex, before luciferase measurement no stimulatory effect on luciferase readout was observed, confirming that Belinostat does not influence the activity and stability of the luciferase protein (Fig 4B).

### The Effect of Belinostat depends on the nature of the GRE-dependent promoter construct

**Fig 5:**
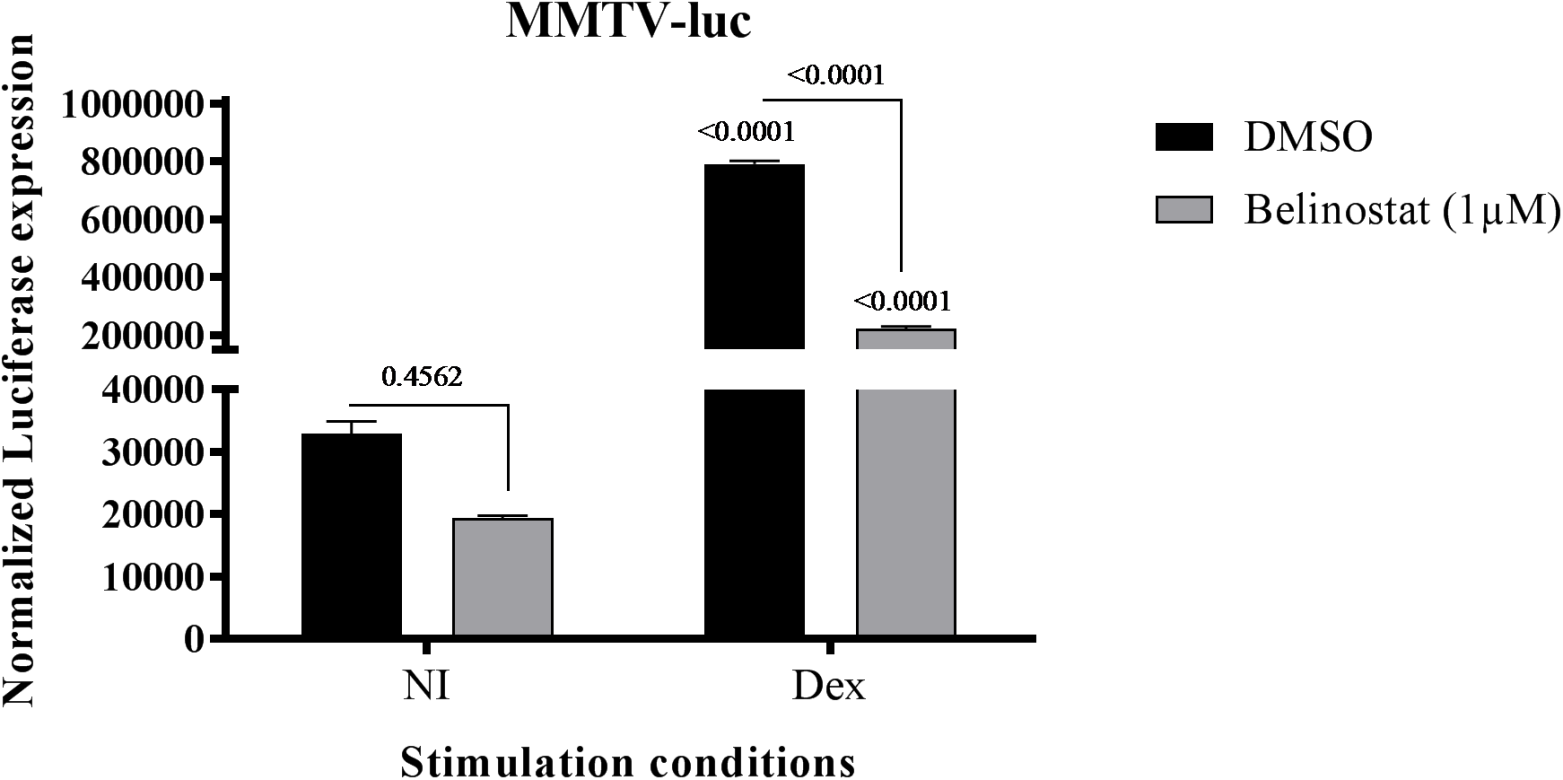
The Effect of Belinostat depends on the nature of the GRE-dependent promoter construct. A549 cells were transiently transfected with MMTV-luc and β-Gal and stimulated with 1μM Belinostat, 16h before Dex treatment (10^-6^M, 5h). Transcriptional activities, normalized against β-Gal expression ± SEM (n = 6).

As shown in Fig 4, Belinostat had stimulatory effects on the Dex-stimulated and TAT promoter-derived GRE-luciferase, stably transfected in A549 cells [57]. The MMTV-luc vector contains the MMTV-LTR (Murine Mammary Tumor Virus Long Terminal repeat) that drives the transcription of the luciferase reporter gene in response to activation of GR. This MMTV-LTR contains six TGTTCT GR responsive half-sites [68]. This reporter, when studied in A549 cells upon transient transfection, responds to Dex (10^-6^M, 5h), but no stimulatory effects of Belinostat (1μM, 16h pre-treatment) were observed in the absence or presence of Dex. On the contrary, Belinostat reduces the luciferase expression (Fig 5). To conclude, the effect of Belinostat on the transcriptional activity of GR heavily depends on the nature of the GRE-driven reporter constructs, with an enhancement of Belinostat on a GR-driven consensus GRE-luc and a diminution of Belinostat on a half-site GRE dependent MMTV-luc.

## Discussion

GCs are potent anti-inflammatory molecules. It is estimated that at least 80 million prescriptions per year in the USA deal with GCs [8]. They are cheap and efficient and found in pills, creams, inhalators, etc. Despite their overall success, doctors and patients are concerned about two negative aspects. First, long term treatment of GCs leads to serious side-effects. Second, large patient groups display a certain degree of GCR [64]. This GCR is related to the degree of inflammation and numerous *in vitro* and *in vivo* studies have investigated the mechanism underlying GCR. Several hypotheses have been described. In mouse models, inflammatory cytokines such as TNF, when injected, lead to a strong and acute GCR [13].

Hundreds of genes have been identified as GRE-dependent genes and are known to be regulated by GCs. In many cases, one or more GRE elements can be coupled to a transcript, based on genome-wide GR binding experiments, but many transcripts have not yet been associated to a GRE element and, conversely, not all GRE elements found in the genome are bound by GR [69]. It is clear that there are number of regulation steps involved in the choice of GRE elements to be bound by GR and in his specific outcome after GRE binding. It is known that particular DNA sequences can modify the structure and function of transcriptional regulators as if they were a ligand, as postulated by Lefstin and Yamamoto [70]. The expression from a consensus GRE or a GRE half-site has a distinct regulation, as stated by the fact that dimerization-defective GR mutants are unable to express the TAT gene, although PNMT expression is normal [71].

HDACs have been linked to GR and other nuclear receptors and transcription factors as they perform repressive activities by reverting histone acetylation. Some papers report that HDACs are also required for transcriptional activity. HDAC6 for instance, acetylates Hsp90 and directly regulates the chaperone-mediated GR activation [53]. HDAC3 acetylates NFκB and consequently stimulates the IL-1-induced gene expression, being a co-activator in inflammatory signaling pathway [25, 72]. On the other hand, reduction in HDAC2 expression and activity leads to enhanced inflammation and reduced steroid responsiveness in chronic obstructive pulmonary disease (COPD) [73]. It has been proven that GR deacetylation by HDAC2 is necessary for suppression of NFκB-mediated inflammatory gene expression, but not for the GRE-mediated gene expression [54]. GR-induced gene transcription is dynamically modulated by the HDAC1 acetylation state. The necessity of HDAC1 as coactivator for GR was first proven for the MMTV gene [55] and later on for the entire GR transcriptome [56].

Since inhibition of HDACs may stimulate transcriptional activity of transcription factors, several HDACs have been studied in detail and numerous HDAC inhibiting drugs have been developed. Some of these broad spectrum HDAC inhibitors have been approved for clinical use in several types of cancers.

We and others have shown before that under TNF-induced GCR conditions, the transcriptional activity of GR is heavily compromised [13]. One way of preventing or reverting GCR might be to search for molecules that stimulate GR transcriptionally activity so that even under GCR conditions, GCs, in combination with such a stimulator, would reach sufficient levels of GR activity. In this respect, we embarked on a screening of chemical compounds using a simple *in vitro* system, consisting of lung epithelial A549 cells, stably transfected with a GRE-luciferase reporter system. Of course, our results and conclusions must be considered within the experimental limits set by the A549 cell line, including its aneuploidies, epigenome, differentiation status, etc. A549 is a very validated cell line, but there is indeed no guarantee that our conclusions (like those of other authors), would still stand in other experimental system.

Based on our screening with A549-GRE-luc cells, it was clear that a number of broad spectrum HDAC inhibitory molecules, such as Belinostat, displayed a strong co-stimulatory effect with Dex, especially with a pre-treatment schedule. The long time course of Belinostat pre-treatment may suggest indirect effects mediated by the altered expression or acetylation of one or more component(s) of the glucocorticoid signaling pathway. The maximal luciferase fold induction obtained with Dex alone (10^-6^M) was increased about four- to ten-fold (Fig 1C).

When studying the impact of Belinostat on a genome-wide scale, only a minority of Dex-induced genes was further enhanced in the presence of Belinostat, or more repressed in case of Dex repressed genes. Belinostat alone had a huge effect on the mRNA levels of thousands of genes. When taking the impact of Belinostat into consideration on Dex-regulated genes without basal Belinostat effect, only 5 genes were found to be further regulated in the same direction by Belinostat (Fig 2D). Among them, the SAA1 gene contains a GRE like match in the promoter (AGATCACTCTGTGCA). We also checked the genes that did show a basal effect of Belinostat alone with a Dex effect for GRE elements and surrounding sequence. However, we could not find GRE elements any more than could be expected by random chance in these groups. Hence, the RNAseq experiment did not correlate in a direct manner to the reporter-based assay. We subsequently investigated several mechanisms that could explain the contradictory effects of Belinostat including the chromatin status, as transformed GRE-luc, integrated in the A549 genome is accepted to be wrapped around histones. These histones contain an N-terminal histone tail which can undergo posttranslational modifications including lysine ubiquitination, sumoylation, phosphorylation but also acetylation [74]. The last one is dynamically modulated by histone acetyltransferases (HAT) and histone deacetylases (HDAC). Acetylated lysines are bound by bromodomains, which are often found in HATs and chromatin remodelling complexes, such as the SWI/SNF complex, which open the chromatin and regulate transcription [75]. HDACi are thought to interfere with transcription by modulating the histone acetylation state. On the other hand, recent studies showed that HDACs could also deacetylate non-histone proteins, such as transcription factors [21, 22]. The transient GRE-luc assay was performed to investigate the role of Belinostat on non-histone proteins since transiently transfected plasmids are not integrated in the genome and aren’t prone to chromatin remodelling. Hence, these findings support the observation that the beneficial effects of Belinostat on the GRE-induction are not caused by histone modifications of target promoters or depends on the genomic integration site of the reporter but rather by effects on components of the GR signalling pathway including nuclear cofactors that link GR with the transcriptional machinery. We investigated the effect of SAHA and Belinostat treatment on GR acetylation, but could not detect any difference in acetylation after HDACi treatment (data not shown), suggesting the involvement of proteins others than GR. Furthermore, the impact of Belinostat on the GRE-luciferase system appeared to be at the level of transcription of the luciferase gene, not on luciferase protein stability or activity. Remarkably, in accordance with the RNAseq data, Belinostat enhances the luciferase expression in absence of GR ligand. We believe that this leakage expression is caused by histone modification and chromatin opening, which favors luciferase expression in absence of activated GR. Although, after Dex stimulation, the main mechanism of which Belinostat exerts its effects is thought to be mediated via components of the GR signalling pathway.

Finally, by testing Belinostat effects in another, and differentially regulated validated GC reporter system, i.e. the MMTV-luciferase reporter, no co-stimulatory effect of Belinostat was observed, but on the contrary, a repressive effect was seen. Although additional experiments will be needed to further pinpoint the exact mechanisms of action of Belinostat on the GRE-luc and MMTV-luc reporter construct, it is of interest that signalling pathways involving different GR responsive elements can be modulated differentially by Belinostat. We have considered a possible mechanism by which Belinostat have an altered effect on GRE-luc and MMTV-luc expression. It has been proposed that the bound DNA sequence acts as an allosteric ligand that alters GR conformation and hence interactions with other proteins such as cofactors which ultimately affect the transcriptional output [76]. Consequently, the sequence of the palindromic GRE site have strong implications for the composition and structure of the regulatory complexes and the mechanisms of context-specific transcriptional regulation [77]. Therefore, we suggest that the differential outcome on GRE-luc and MMTV-luc of a Belinostat treatment is due to a different impact of Belinostat on each cofactor profile. Blind *et al*. demonstrated that the histone acetyltransferase p300 is recruited to the TAT-GRE promoter in a Dex responsive way, indicating a role of p300 in GR transcriptional activity [78]. On the other hand, Qiu *et al* identified HDAC1 as coactivator for GR-induced transcription of the MMTV promoter [79]. More specifically, the acetylation state of HDAC1 with the activity state of the promoter and affects the exchange rate of HDAC1 at the promoter site. This indicates that HDACi may affect this acetylation status and consequently negatively regulate MMTV expression. Taken together, the GR binding sequence in the promoter region modulates the GR conformation which attracts different nuclear cofactors depending on the promoter context. These nuclear TFs contain chromatin remodeling activities and are prone to HDAC/HAT activities. They link the GR to the transcriptional machinery and affect gene regulation. One additional note, as seen by ChIPseq, GR binding sites are often located far from promoter regions and transcription start sites, suggesting that responsive elements can loop towards promoter regions of target genes in order to regulate transcription [80]. These looping events are mediated by a large nuclear protein complex which is prone to additional post-translational modifications. The synthetic reporter constructs GRE-luc and MMTV-luc contain the GR binding site closely to their promoter, which make looping unnecessary. This difference in regulation between endogenous genes and the luciferase gene might be an additional reason for the difference in results of the screening assay and the RNAseq.

Our work has contributed to identify opposite GRE-specific regulatory effects by the HDACi Belinostat, with a stimulatory effect on the classic palindromic GRE derived from the TAT promoter and an inhibitory effect on the specific GRE from the MMTV promoter. To extract generalizing conclusions based on reporter gene assays, our take-home message would be to not solely rely on one type of promoter-luciferase reporter, but invest in different ones and also invest in non-reporter dependent validation assays.

## Supporting information

S1 Table List of genes differentially expressed after Dex. (PDF)

S2 Table List of genes differentially expressed after Belinostat. (PDF)

## Abbreviations

AP-1: activator protein 1
CLP: cecal ligation and puncture
Dex: dexamethasone
DMSO: dimethylsulfoxide
GC: glucocorticoid
GCR: glucocorticoid resistance
GR: glucocorticoid receptor
GRE: glucocorticoid receptor element
HAT: histone acetyltransferase
HDAC: histone deacetylase
HDACi: HDAC inhibitor
LPS: lipopolysaccharide
MMTV: mouse mammary tumor virus
MKP1: MAPK phosphatase 1
NAD: nicotinamide adenine dinucleotide
NFκB: nuclear factor kappa B
RNAseq: RNA sequencing
SAHA: suberanilohydroxamic acid
TNF: tumor necrosis factor.
