# Supplementary material for: The nature of the GRE influences the screening for GR-activity enhancing modulators
Source: PLoS One. 2017 Jul 7;12(7):e0181101. doi: 10.1371/journal.pone.0181101 (PMC5501670; doi:10.1371/journal.pone.0181101)
Supplement: S1 Table — (PDF) [file pone.0181101.s001.pdf]

List of genes differentially expressed after Dex (|LFC| >1; adjusted p-val < 0.01)

**Ensembl id**	**Symbol**	**LFC**	**adj. p-value**
ENSG00000182902	SLC25A18	11.9079514198414	5.00990978234684e-05
ENSG00000090376	IRAK3	11.5292200619895	9.47101430014692e-05
ENSG00000087495	PHACTR3	10.9103580794871	0.000253372505317457
ENSG00000092051	JPH4	10.3166127300225	0.000611486521700989
ENSG00000133401	PDZD2	10.00588651625	0.000975101598402001
ENSG00000039987	BEST2	9.77052889427244	2.06509690993621e-10
ENSG00000109906	ZBTB16	8.75137711783978	0.00524308831459395
ENSG00000122787	AKR1D1	8.71989850660782	0.0058717899566542
ENSG00000130701	RBBP8NL	8.68515389393369	0.00634805317256534
ENSG00000166589	CDH16	8.58756867073379	7.37389211207707e-38
ENSG00000174358	SLC6A19	8.52240342882853	5.88140918407863e-08
ENSG00000152086	TUBA3E	8.4575084053714	0.00775009883493679
ENSG00000075886	TUBA3D	8.38412612600321	0.00894725219527341
ENSG00000179593	ALOX15B	8.37955620861399	0.00832537093649854
ENSG00000196091	MYBPC1	8.3125697829119	0.00917565590580248
ENSG00000124205	EDN3	8.09232534624717	3.25274430648482e-07
ENSG00000161896	IP6K3	7.88068281547067	7.48971099952259e-07
ENSG00000141750	STAC2	6.32069468642489	1.6819709879093e-48
ENSG00000152779	SLC16A12	6.28340082295343	1.12768033526495e-34
ENSG00000167183	PRR15L	6.20762319849348	1.19624820941484e-25
ENSG00000084110	HAL	6.16927772735347	1.67620901369697e-11
ENSG00000166592	RRAD	6.1679362714822	1.79805816674834e-22
ENSG00000162391	FAM151A	6.165185029684	0.000349649818995546
ENSG00000168824	N.A.	6.06666596288526	0.000267213417857834
ENSG00000163884	KLF15	6.04893128142515	5.09558666700203e-11
ENSG00000198729	PPP1R14C	6.04480361070977	1.61393885872889e-21
ENSG00000166828	SCNN1G	6.03412707820934	2.9548775339448e-126
ENSG00000163283	ALPP	6.02171294247829	5.9605942404051e-31
ENSG00000096088	PGC	5.97952108864825	0.000432165922800658
ENSG00000120675	DNAJC15	5.80460591052714	1.12653541604605e-34
ENSG00000170369	CST2	5.77912143435762	0.00111609322369428
ENSG00000169583	CLIC3	5.74602340899402	0.000723819966054363
ENSG00000170373	CST1	5.6494744916249	3.05290601386306e-20
ENSG00000168874	ATOH8	5.63749234612669	2.5204982130242e-15
ENSG00000173262	SLC2A14	5.61977111392857	1.38609616451118e-14
ENSG00000183671	GPR1	5.54692496491294	7.69352799037109e-09
ENSG00000127083	OMD	5.42461110978569	1.14740126753927e-05
ENSG00000061337	LZTS1	5.40888184884099	0.00164893226847426
ENSG00000168135	KCNJ4	5.36759201354453	9.1992878297331e-06
ENSG00000163995	ABLIM2	5.28503759737237	4.38208917394061e-22
ENSG00000008516	MMP25	5.20442535383141	0.00395470664773113
ENSG00000188305	C19orf35	5.15441805227715	0.00619347638066061
ENSG00000011465	DCN	5.12674977909956	0.00409552796480488
ENSG00000170379	TCAF2	5.10308534838925	2.16105789723494e-15
ENSG00000122824	NUDT10	5.08357081223152	0.00453329820654407

ENSG00000168309	FAM107A	5.04358073123534	0.00585099937812232
ENSG00000131771	PPP1R1B	5.03987576787926	0.00505704449372469
ENSG00000129757	CDKN1C	5.03397874541288	3.80389431773132e-43
ENSG00000158292	GPR153	4.94172533935185	9.29436701493345e-172
ENSG00000243709	LEFTY1	4.90094899475713	0.00997537161150695
ENSG00000101082	SLA2	4.81054935437705	0.000281989430774671
ENSG00000054179	ENTPD2	4.79355148632707	3.40946429456805e-141
ENSG00000187288	CIDEC	4.79049506370985	5.28368601302232e-207
ENSG00000170271	FAXDC2	4.76538569363677	1.09509984338284e-56
ENSG00000107731	UNC5B	4.73966399773023	4.1506777392209e-30
ENSG00000143858	SYT2	4.73554618572815	5.90978168404328e-16
ENSG00000158014	SLC30A2	4.71226301202855	0.00848558930983026
ENSG00000069188	SDK2	4.70110496705319	2.60410112080778e-19
ENSG00000176387	HSD11B2	4.63473641541235	6.06051767767884e-30
ENSG00000157570	TSPAN18	4.59401913362459	7.22007621022123e-42
ENSG00000275465	N.A.	4.56566985669955	0.000716978957334248
ENSG00000143845	ETNK2	4.54975674075644	0
ENSG00000168675	LDLRAD4	4.4716770921454	1.2055853606552e-11
ENSG00000185924	RTN4RL1	4.45632577561318	9.56820283230887e-63
ENSG00000107551	RASSF4	4.41408819278166	3.7675864697854e-142
ENSG00000157445	CACNA2D3	4.40829750226095	0.00107710807604553
ENSG00000096060	FKBP5	4.4011278550336	0
ENSG00000106789	CORO2A	4.21077596597577	1.35136425286417e-245
ENSG00000145569	FAM105A	4.17763583343361	0
ENSG00000120057	SFRP5	4.14081909531553	2.13805528855358e-17
ENSG00000182489	XKRX	4.11770659185596	3.56002469431093e-08
ENSG00000107954	NEURL1	4.09604058150982	6.57777849386682e-20
ENSG00000165548	TMEM63C	4.04880202847541	2.67330293830512e-105
ENSG00000119138	KLF9	4.04200205595832	1.46191751988893e-84
ENSG00000089356	FXYD3	4.01531426177597	0.00356507173218181
ENSG00000183578	TNFAIP8L3	4.01137737466806	0.000137452092715016
ENSG00000178597	PSAPL1	4.00945368221681	2.92312055798619e-08
ENSG00000227695	DNMBP-AS1	4.00459591016428	0.00303794796436558
ENSG00000157514	TSC22D3	3.97256846059353	0
ENSG00000036672	USP2	3.94427565509431	7.1387502365276e-40
ENSG00000120129	DUSP1	3.90531957886777	0
ENSG00000185432	METTL7A	3.88738210076008	5.58237745010894e-90
ENSG00000276509	N.A.	3.81853850635296	0.00208111296602079
ENSG00000076641	PAG1	3.80116851999746	0.000102219443843381
ENSG00000197177	ADGRA1	3.70461378773432	0.000275933151910848
ENSG00000137198	GMPR	3.69403966435821	2.99552075040903e-51
ENSG00000117643	MAN1C1	3.67723766174685	5.12154426979627e-81
ENSG00000134042	MRO	3.66574650740431	0.00477550147427682
ENSG00000105641	SLC5A5	3.65012537091997	3.15334917913152e-15
ENSG00000115112	TFCP2L1	3.63439929918458	0
ENSG00000138615	CILP	3.6210958943125	1.13828805668056e-09
ENSG00000119686	FLVCR2	3.60714401889966	5.72014801279233e-29
ENSG00000172771	EFCAB12	3.60026842102647	2.87010424932626e-34
ENSG00000132965	ALOX5AP	3.58444402727106	1.00611788356122e-07

ENSG00000186081	KRT5	3.58282406547861	0.00507563804300352
ENSG00000171522	PTGER4	3.5489704799691	2.73685108468368e-51
ENSG00000280753	N.A.	3.51993967208207	0.000768262947938099
ENSG00000179094	PER1	3.48495161622747	0
ENSG00000127585	FBXL16	3.4827051111398	4.85146816734082e-62
ENSG00000183508	FAM46C	3.48109049965888	2.41695955038044e-06
ENSG00000150594	ADRA2A	3.4774757721992	8.78497763586717e-51
ENSG00000163993	S100P	3.47511879267264	6.0065623644037e-242
ENSG00000253882	N.A.	3.43017590516451	2.00076507745748e-10
ENSG00000107593	PKD2L1	3.41891076060643	0.00469206737325377
ENSG00000123342	MMP19	3.40625249501437	0.00165922879965228
ENSG00000068831	RASGRP2	3.39805850182914	9.75809835647915e-07
ENSG00000006071	ABCC8	3.39710483098789	1.06969840100975e-62
ENSG00000146678	IGFBP1	3.388347493288	1.46003652925671e-115
ENSG00000128512	DOCK4	3.37394157419821	8.40753611938605e-204
ENSG00000158104	HPD	3.37172866849463	2.92073067990867e-08
ENSG00000112561	TFEB	3.34417988686848	5.08622861237249e-78
ENSG00000143127	ITGA10	3.33187719252676	2.82182228501512e-23
ENSG00000132821	VSTM2L	3.30648276198991	3.931942813457e-69
ENSG00000110328	GALNT18	3.23756326843318	1.28283149542707e-107
ENSG00000179846	NKPD1	3.23718203997745	6.06422272583537e-08
ENSG00000185338	SOCS1	3.231898137205	3.69758498243225e-05
ENSG00000107159	CA9	3.2235024629496	2.35318715015645e-13
ENSG00000135116	HRK	3.18027840486802	4.1925407970338e-10
ENSG00000170153	RNF150	3.16653074200065	3.09717357462207e-10
ENSG00000159208	CIART	3.16082602268376	0.000695243239669143
ENSG00000147642	SYBU	3.15006404353449	0
ENSG00000158445	KCNB1	3.14394611619401	7.47755585354514e-20
ENSG00000160801	PTH1R	3.13947467763089	1.71283570173742e-12
ENSG00000166825	ANPEP	3.12323542656553	1.42085122661116e-77
ENSG00000103942	HOMER2	3.11761303299291	2.42221778754142e-227
ENSG00000188910	GJB3	3.08039603316284	7.69090427560665e-12
ENSG00000261068	N.A.	3.06458986265521	0.000499760891451092
ENSG00000135447	PPP1R1A	3.01627264361342	5.97114500545821e-05
ENSG00000044012	GUCA2B	3.00694127615996	0.000978088465356908
ENSG00000104967	NOVA2	2.99953313517424	5.53172620500574e-22
ENSG00000165507	C10orf10	2.99346420262254	0.000202107373029225
ENSG00000143001	TMEM61	2.99298651264535	3.65179906887867e-05
ENSG00000279700	N.A.	2.96851439585718	3.39531944002718e-06
ENSG00000204531	POU5F1	2.95981296401174	1.89737034969173e-23
ENSG00000131620	ANO1	2.94075810481758	7.75542239588496e-07
ENSG00000152463	OLAH	2.93575651389749	2.46126330354361e-05
ENSG00000185112	FAM43A	2.91408037845081	1.96435909115918e-72
ENSG00000187486	KCNJ11	2.90168503448901	1.14761740383276e-43
ENSG00000068615	REEP1	2.869861885225	1.85091159451174e-84
ENSG00000167653	PSCA	2.86690875740978	1.61051370218591e-05
ENSG00000203697	CAPN8	2.85070997537119	1.591112965669e-10
ENSG00000123689	G0S2	2.83429153245601	1.02196622071152e-05
ENSG00000080618	CPB2	2.83126211440965	3.89534584381235e-16

ENSG00000162426	SLC45A1	2.80955414014045	3.63703579545168e-75
ENSG00000134531	EMP1	2.80540919957339	3.34620647160201e-207
ENSG00000197142	ACSL5	2.80377393516866	1.38846862420666e-35
ENSG00000157343	ARMC12	2.7881576234327	1.74331615566608e-08
ENSG00000134830	C5AR2	2.78108787081604	3.73971533119078e-20
ENSG00000131746	TNS4	2.77057140002049	7.09088906408749e-69
ENSG00000274414	N.A.	2.7682654300773	1.27742104053045e-60
ENSG00000234076	TPRG1-AS1	2.76414626705489	0.00320183590242984
ENSG00000133808	MICALCL	2.7636120239568	0.00635244667664839
ENSG00000119782	FKBP1B	2.74599220693344	3.53357622633816e-09
ENSG00000279806	N.A.	2.71774705926765	0.000148220657874359
ENSG00000168209	DDIT4	2.69600353264039	0
ENSG00000236673	N.A.	2.69181653366849	0.000602573526033074
ENSG00000214357	NEURL1B	2.68602631780231	1.72512803662524e-124
ENSG00000128016	ZFP36	2.6823773601355	0
ENSG00000100906	NFKBIA	2.68052903459581	0
ENSG00000116254	CHD5	2.6712711549808	3.42973446775193e-35
ENSG00000166165	CKB	2.66577196835672	0
ENSG00000151136	BTBD11	2.65069408452539	1.56347876622758e-300
ENSG00000175287	PHYHD1	2.63823781950541	0.00921779079033406
ENSG00000162745	OLFML2B	2.63659371453118	9.0903434402122e-06
ENSG00000106852	LHX6	2.63487592971386	0.00195171527938784
ENSG00000127129	EDN2	2.62161824047602	0.00148827016328958
ENSG00000166741	NNMT	2.62070325679087	2.3106974960059e-93
ENSG00000189058	APOD	2.62062622434113	2.39958967129e-53
ENSG00000114812	VIPR1	2.60861327501132	3.81303118588459e-55
ENSG00000182575	NXPH3	2.60429629683433	0.0054381493399559
ENSG00000122862	SRGN	2.60349006625514	9.51075170090007e-27
ENSG00000075240	GRAMD4	2.59334406599011	8.89360806977533e-203
ENSG00000028137	TNFRSF1B	2.5881537581643	0.00488639264672251
ENSG00000026559	KCNG1	2.58718924547664	4.39107567418802e-31
ENSG00000261033	N.A.	2.5700452551265	5.44596258023645e-05
ENSG00000091129	NRCAM	2.56829223541206	3.01118595962755e-98
ENSG00000139132	FGD4	2.56446517020011	0
ENSG00000113763	UNC5A	2.54595819045341	0.000153923590731835
ENSG00000115468	EFHD1	2.54064900313691	1.84187862691452e-23
ENSG00000076356	PLXNA2	2.52480796410891	6.92564723353941e-215
ENSG00000023445	BIRC3	2.52169382063524	0
ENSG00000279117	N.A.	2.51523514467834	0
ENSG00000104044	OCA2	2.5139821880236	5.30392593832094e-07
ENSG00000112787	FBRSL1	2.49860290180012	0
ENSG00000168447	SCNN1B	2.49356348880703	5.73975159647726e-77
ENSG00000160179	ABCG1	2.49022903999318	2.67343052523894e-05
ENSG00000001617	SEMA3F	2.45457430721144	6.87367456136248e-40
ENSG00000122367	LDB3	2.45143794686703	0.00143854793169875
ENSG00000137699	TRIM29	2.4466105556743	0.000352614391436595
ENSG00000204528	PSORS1C3	2.42517800614357	9.21009012496862e-09
ENSG00000141449	GREB1L	2.41186263614157	4.94480105291697e-77
ENSG00000155846	PPARGC1B	2.41067560814102	1.37206709258248e-117

ENSG00000116741	RGS2	2.40966872943049	1.37593079785642e-178
ENSG00000160712	IL6R	2.40855826961478	5.17798990325628e-138
ENSG00000124126	PREX1	2.38897932650768	5.79404765551654e-119
ENSG00000125730	C3	2.38414333767738	6.27953991398949e-46
ENSG00000167772	ANGPTL4	2.38034134799976	2.04528142815525e-165
ENSG00000136378	ADAMTS7	2.37748965634488	7.93208053802118e-70
ENSG00000111319	SCNN1A	2.3770543746678	8.2188506160641e-180
ENSG00000158747	NBL1	2.37665834903192	1.21943320969431e-55
ENSG00000172216	CEBPB	2.37512221747251	5.20570395951779e-233
ENSG00000006638	TBXA2R	2.3627247183481	2.97321069684441e-06
ENSG00000121671	CRY2	2.33716094559713	1.87378121920752e-90
ENSG00000188112	C6orf132	2.33409933574913	5.05079902084971e-20
ENSG00000071242	RPS6KA2	2.32797459416747	8.41783770339021e-06
ENSG00000172824	CES4A	2.3200075538511	7.67930075869534e-30
ENSG00000103647	CORO2B	2.31840115697772	6.97674250971006e-11
ENSG00000135678	CPM	2.31751215960502	1.72416817109654e-83
ENSG00000231274	SBK3	2.31694669640075	7.44502644988289e-32
ENSG00000118503	TNFAIP3	2.30346478847234	2.12119685709009e-42
ENSG00000072071	ADGRL1	2.30254523023828	1.72037968944141e-149
ENSG00000170873	MTSS1	2.29158769840718	2.37685034860379e-27
ENSG00000118515	SGK1	2.28917571938387	4.33483225487818e-221
ENSG00000168631	DPCR1	2.2841182195052	0.00042829845043954
ENSG00000125148	MT2A	2.26605927256667	1.09966695729426e-99
ENSG00000147119	CHST7	2.25962898182423	1.52014020711963e-71
ENSG00000187957	DNER	2.25462715130428	1.4897029295064e-20
ENSG00000079308	TNS1	2.23993514754937	9.59394509718826e-08
ENSG00000164683	HEY1	2.19699955552985	9.2041299571097e-05
ENSG00000095383	TBC1D2	2.16903480778643	1.82382588747314e-45
ENSG00000213853	EMP2	2.16442765960222	0
ENSG00000149596	JPH2	2.15087076295844	1.63271121928241e-18
ENSG00000069812	HES2	2.14954289849602	4.46866133826177e-05
ENSG00000116285	ERRFI1	2.14866922531776	0
ENSG00000055118	KCNH2	2.14696151238837	2.26718077078641e-26
ENSG00000008311	AASS	2.14227895102635	1.42367245589368e-10
ENSG00000188738	FSIP2	2.12873367142229	2.4398729866344e-07
ENSG00000118689	FOXO3	2.12466994165324	1.68874477283235e-195
ENSG00000137486	ARRB1	2.12061695740731	0
ENSG00000221869	CEBPD	2.11737540495717	7.84779188279744e-132
ENSG00000008853	RHOBTB2	2.1172683215209	1.96239591552693e-223
ENSG00000248596	N.A.	2.09999684709426	1.45398195815026e-08
ENSG00000169085	C8orf46	2.09855764321812	0.00526134180481621
ENSG00000158246	FAM46B	2.09485238700407	1.022597186912e-06
ENSG00000263155	MYZAP	2.08772618225765	2.33042523015537e-05
ENSG00000175866	BAIAP2	2.07955216518089	2.1733505541903e-120
ENSG00000120162	MOB3B	2.07793850066926	2.58797669023579e-27
ENSG00000135127	CCDC64	2.07763420199438	4.97945392581313e-97
ENSG00000100994	PYGB	2.06500982110889	0
ENSG00000171119	NRTN	2.05895053337156	1.53776086782514e-06
ENSG00000125089	SH3TC1	2.04081505372246	7.89295264528123e-68

ENSG00000116690	PRG4	2.03070227806104	0.000106862052605606
ENSG00000235217	TSPY26P	2.02756595522739	0.00356342863430852
ENSG00000168481	LGI3	2.02458692032997	2.10326707641401e-08
ENSG00000187193	MT1X	2.02425852646076	1.38950527255816e-26
ENSG00000115756	HPCAL1	2.02398218629173	7.82412712390906e-278
ENSG00000131781	FMO5	2.02098052299031	1.75026166787309e-34
ENSG00000274307	N.A.	2.0161936595673	0.00466465063366422
ENSG00000196220	SRGAP3	2.01128925487822	3.41477734224397e-22
ENSG00000108387	SEPT4	2.00747388483514	2.93578264491905e-07
ENSG00000049130	KITLG	2.00600894695601	2.03840076421234e-73
ENSG00000180448	HMHA1	2.00223122517453	1.45496953120568e-10
ENSG00000153404	PLEKHG4B	2.00107846395013	1.76792346683954e-40
ENSG00000118804	FAM47E-STBD1	1.99762240537035	8.02745188857033e-06
ENSG00000185745	IFIT1	1.990302515464	6.68413638056249e-45
ENSG00000163171	CDC42EP3	1.97847282303976	3.52592647843199e-178
ENSG00000174460	ZCCHC12	1.95966053542488	0.000446117297967509
ENSG00000140939	NOL3	1.95887840830095	6.62477256238621e-95
ENSG00000166689	PLEKHA7	1.95885731100741	1.16600213688767e-258
ENSG00000163659	TIPARP	1.95701639885432	3.13469756763516e-266
ENSG00000164849	GPR146	1.94883371447004	0.0013752922833708
ENSG00000154065	ANKRD29	1.94697675349962	1.80824664617e-57
ENSG00000099998	GGT5	1.93553278879529	2.25539497216216e-06
ENSG00000074181	NOTCH3	1.92750118522265	2.86275418183642e-44
ENSG00000022567	SLC45A4	1.92200958835771	1.08444297450898e-250
ENSG00000185499	MUC1	1.92019129083987	2.13288287506315e-14
ENSG00000128274	A4GALT	1.91842360213862	5.49065148631074e-76
ENSG00000125850	OVOL2	1.91419712522878	0.00316502962306701
ENSG00000143797	MBOAT2	1.91212720771518	9.09879686535092e-217
ENSG00000198355	PIM3	1.90938495110557	1.70832922892476e-163
ENSG00000133328	HRASLS2	1.90929973965474	0.000117063361793701
ENSG00000175785	PRIMA1	1.90826067574744	0.00716967963551823
ENSG00000015413	DPEP1	1.90031640165886	0.000349126786179474
ENSG00000189223	PAX8-AS1	1.89926405530056	0.00703848881093172
ENSG00000091622	PITPNM3	1.89547717501993	1.71228902928229e-22
ENSG00000153902	LGI4	1.89532154837774	0.00470555889324784
ENSG00000280287	N.A.	1.89335786462418	4.63545149569018e-11
ENSG00000133121	STARD13	1.8928816544422	1.34967082043969e-50
ENSG00000163932	PRKCD	1.88885047402921	3.33608169754341e-169
ENSG00000148175	STOM	1.88656818473032	0
ENSG00000166106	ADAMTS15	1.88643261430656	1.56942327479362e-35
ENSG00000095203	EPB41L4B	1.88024264294656	9.80379334603029e-99
ENSG00000137571	SLCO5A1	1.87776410968502	2.10970291007893e-05
ENSG00000187123	LYPD6	1.87279667983402	8.18507095360895e-19
ENSG00000223657	KRT8P51	1.86465974085138	0.00361052939700886
ENSG00000184205	TSPYL2	1.86020548262961	6.03396028820818e-141
ENSG00000102760	RGCC	1.85745104301211	1.85526089111723e-10
ENSG00000134250	NOTCH2	1.85495173563722	0
ENSG00000240875	LINC00886	1.85341823596073	1.15665916009559e-34
ENSG00000271383	NBPF19	1.84928607659847	1.65533279408275e-34

ENSG00000104419	NDRG1	1.84790621945104	1.66454306933491e-186
ENSG00000165757	KIAA1462	1.84319714244387	1.7479022675588e-112
ENSG00000104327	CALB1	1.83619922524998	0.000386595308675696
ENSG00000099860	GADD45B	1.83037484058871	4.2062479457461e-60
ENSG00000132470	ITGB4	1.82541134189651	3.5049649858008e-304
ENSG00000230882	N.A.	1.82511473680327	4.41976169395453e-55
ENSG00000279970	N.A.	1.81619050192969	6.07037835530983e-05
ENSG00000179862	CITED4	1.81426260901979	1.67302511090367e-22
ENSG00000134917	ADAMTS8	1.8009222851523	2.06784624037285e-05
ENSG00000254815	N.A.	1.79988848516795	5.03018125425587e-08
ENSG00000275216	N.A.	1.79968440703112	1.23574475264725e-119
ENSG00000173698	ADGRG2	1.79887456526633	2.00549721041475e-87
ENSG00000186197	EDARADD	1.79849910999059	1.23876863160716e-65
ENSG00000125968	ID1	1.78913664332186	1.82933744251171e-218
ENSG00000106366	SERPINE1	1.78653941990719	0
ENSG00000175175	PPM1E	1.78339913084667	0.000834768853208086
ENSG00000172159	FRMD3	1.7832433015273	5.0514008204275e-23
ENSG00000105974	CAV1	1.77343720967313	0
ENSG00000163354	DCST2	1.76617709253309	2.9523010706807e-06
ENSG00000186198	SLC51B	1.7613570991398	7.71340679433203e-05
ENSG00000126870	WDR60	1.76058266007035	4.75927722959436e-121
ENSG00000124507	PACSIN1	1.75952581640442	2.13453780062592e-08
ENSG00000186648	LRRC16B	1.75552437271873	0.0015658981817913
ENSG00000163874	ZC3H12A	1.75538577755494	1.18950654822475e-63
ENSG00000151726	ACSL1	1.75180272429987	8.4007591178434e-157
ENSG00000170846	N.A.	1.74735313809457	2.05047942187035e-60
ENSG00000124942	AHNAK	1.74672296802727	0
ENSG00000189221	MAOA	1.74578556964129	1.22746183194397e-28
ENSG00000070182	SPTB	1.73539989408163	7.05798091990315e-35
ENSG00000264343	N.A.	1.72987869664851	2.84433180759801e-18
ENSG00000153208	MERTK	1.72940605682532	6.22045841852138e-45
ENSG00000087303	NID2	1.72508242595913	2.77815803030441e-43
ENSG00000125430	HS3ST3B1	1.72496995986683	0.000392320369021743
ENSG00000164120	HPGD	1.72201105606152	1.17683180225803e-82
ENSG00000090530	P3H2	1.72100172529335	1.36870595386934e-115
ENSG00000168497	SDPR	1.7151008888107	1.34417717578584e-44
ENSG00000162433	AK4	1.70781622791778	3.75513563355865e-131
ENSG00000133816	MICAL2	1.70778930159056	8.48225636630551e-136
ENSG00000128268	MGAT3	1.70380889470706	2.72550585312332e-07
ENSG00000140526	ABHD2	1.70319349839628	0
ENSG00000142733	MAP3K6	1.69828250671687	1.01853916654054e-48
ENSG00000153822	KCNJ16	1.69819304845389	3.44169303298329e-12
ENSG00000053747	LAMA3	1.69758286547363	5.39843597449831e-197
ENSG00000198959	TGM2	1.69692561977791	8.46363155929089e-190
ENSG00000164442	CITED2	1.69230444608108	4.4103246532422e-71
ENSG00000279662	N.A.	1.69027960564024	4.81208714620952e-05
ENSG00000197444	OGDHL	1.68605682188697	0.00994724508352205
ENSG00000144642	RBMS3	1.67475432652165	9.86965466453213e-13
ENSG00000170876	TMEM43	1.67369898431946	5.45931688940369e-251

ENSG00000107438	PDLIM1	1.67300944239373	8.676409572504e-201
ENSG00000226015	CCT8P1	1.67035765089559	1.17393436814724e-06
ENSG00000115129	TP53I3	1.66839352269748	1.463749658544e-160
ENSG00000106348	IMPDH1	1.66679295332689	3.95888355073119e-210
ENSG00000125637	PSD4	1.66559129509347	4.26941354846162e-20
ENSG00000151892	GFRA1	1.66247455716441	0.000510003747691224
ENSG00000057294	PKP2	1.65252855669374	1.16870928749838e-162
ENSG00000081041	CXCL2	1.64912198665882	2.9424325811624e-08
ENSG00000169933	FRMPD4	1.64797951404283	0.00603290496024283
ENSG00000227038	GTF2IP7	1.64040694749204	5.31217837077982e-10
ENSG00000111077	TNS2	1.63830321556755	1.45997049741401e-163
ENSG00000178726	THBD	1.63577217543696	7.14584066253986e-105
ENSG00000170345	FOS	1.63403774557635	8.28335021746293e-19
ENSG00000128482	RNF112	1.63360644290595	1.2280363310802e-05
ENSG00000125510	OPRL1	1.63208112130233	7.48420463838627e-09
ENSG00000103415	HMOX2	1.62849666255015	4.82191750576436e-113
ENSG00000197415	VEPH1	1.62608792582921	2.72875846994617e-63
ENSG00000117245	KIF17	1.62229142562204	0.000794342888482024
ENSG00000171368	TPPP	1.62168532224697	1.19707785048257e-30
ENSG00000004799	PDK4	1.61833053203945	2.39978274110251e-184
ENSG00000242759	LINC00882	1.61578087159405	0.000748655170931097
ENSG00000172943	PHF8	1.61553197794821	5.34613445434415e-150
ENSG00000122786	CALD1	1.61418166532715	2.17383939123253e-255
ENSG00000135406	PRPH	1.61223847051997	0.00232596359427827
ENSG00000165887	ANKRD2	1.60534089820187	4.23013002677654e-06
ENSG00000182749	PAQR7	1.60321120378444	8.99769201773109e-46
ENSG00000271425	NBPF10	1.60095084743351	1.55959702629287e-10
ENSG00000270629	NBPF14	1.60093007621212	4.17932834594009e-29
ENSG00000170776	AKAP13	1.60020326987802	0
ENSG00000261115	TMEM178B	1.59679601245	2.54192960953484e-10
ENSG00000130589	HELZ2	1.5895848180622	4.19267674472979e-62
ENSG00000184557	SOCS3	1.58789026182108	1.96121271498078e-72
ENSG00000226180	N.A.	1.58534795963822	0.000527591860438162
ENSG00000004468	CD38	1.57330439270862	2.29106734342088e-32
ENSG00000170871	KIAA0232	1.57239135356521	1.17086928958059e-191
ENSG00000164674	SYTL3	1.5715818063044	0.00233165911491511
ENSG00000128564	VGF	1.57076368299943	0.000499444988887981
ENSG00000218891	ZNF579	1.56555948877897	1.47744966477983e-66
ENSG00000197375	SLC22A5	1.55875116255042	2.49563191177793e-139
ENSG00000140479	PCSK6	1.55691655473265	4.66950628492117e-80
ENSG00000113594	LIFR	1.5566815921849	1.01787498622178e-54
ENSG00000273001	N.A.	1.55266738366012	0.00734751272141253
ENSG00000167202	TBC1D2B	1.53661213429656	3.27428902399744e-140
ENSG00000183943	PRKX	1.52964097716851	1.64810546461652e-69
ENSG00000197580	BCO2	1.52829029832604	7.3129632592593e-11
ENSG00000154217	PITPNC1	1.52493266207369	3.20855756682545e-18
ENSG00000227051	C14orf132	1.52071892571232	2.9424325811624e-08
ENSG00000260877	N.A.	1.52051593920877	2.9523010706807e-06
ENSG00000151151	IPMK	1.51758251507417	9.56435804410684e-74

ENSG00000276850	N.A.	1.5175128456901	0.000964907451182984
ENSG00000090975	PITPNM2	1.51709671312793	2.57337913511649e-52
ENSG00000166833	NAV2	1.51261246087589	5.96227373174121e-105
ENSG00000050344	NFE2L3	1.50198290982277	4.06295215761046e-49
ENSG00000110080	ST3GAL4	1.49394777863019	1.12009717048554e-37
ENSG00000104635	SLC39A14	1.49336847781006	0
ENSG00000142677	IL22RA1	1.49296863216132	7.08253485876465e-15
ENSG00000113916	BCL6	1.49224307512624	5.37279653949288e-72
ENSG00000273760	N.A.	1.49096740333166	5.12634102518446e-05
ENSG00000261578	N.A.	1.49000440108291	3.84916303105071e-16
ENSG00000141753	IGFBP4	1.48638794830153	0
ENSG00000119630	PGF	1.4833552079409	0.00603133819937157
ENSG00000173705	SUSD5	1.47688865832849	0.000453325587739447
ENSG00000008513	ST3GAL1	1.47604825252138	2.86382024258845e-118
ENSG00000057657	PRDM1	1.47182474382553	8.08853305844812e-05
ENSG00000118898	PPL	1.47122773661179	4.60966081666252e-63
ENSG00000153294	ADGRF4	1.4686170532502	8.35520712568472e-06
ENSG00000164251	F2RL1	1.46768518586164	1.79476507012169e-212
ENSG00000198417	MT1F	1.4621430599952	3.17847345283784e-08
ENSG00000197070	ARRDC1	1.46151210421786	1.55040438988098e-82
ENSG00000196208	GREB1	1.46055575649122	1.25520234016356e-07
ENSG00000214076	CPSF1P1	1.45869451783649	2.80710938902457e-09
ENSG00000274528	N.A.	1.45762824025991	0.00514505483791656
ENSG00000278266	N.A.	1.45718583593362	6.98563464692595e-06
ENSG00000181104	F2R	1.45653794650529	7.77176003911343e-133
ENSG00000170214	ADRA1B	1.45466415689649	2.5027771151295e-22
ENSG00000168398	BDKRB2	1.45078759327534	0.000253372505317457
ENSG00000078114	NEBL	1.4502214195182	5.95633993294604e-109
ENSG00000100266	PACSIN2	1.4489273564072	7.58235131226528e-214
ENSG00000092445	TYRO3	1.44367428190704	9.34184932225805e-41
ENSG00000065413	ANKRD44	1.43803925544158	7.67910272999764e-16
ENSG00000203867	RBM20	1.43603488483152	7.46067196378889e-30
ENSG00000035403	VCL	1.43561205330614	0
ENSG00000006704	GTF2IRD1	1.43551255527893	1.64859415728472e-56
ENSG00000151090	THRB	1.43540606593615	2.2006358190713e-12
ENSG00000153823	PID1	1.43118404523356	0.00023858495747756
ENSG00000214145	LINC00887	1.42379728859253	0.0012234195343636
ENSG00000169894	MUC3A	1.42299549713939	8.30902365143402e-05
ENSG00000182795	C1orf116	1.41961068213717	1.04344874255719e-65
ENSG00000166866	MYO1A	1.41633876254961	0.000332844988570299
ENSG00000116032	GRIN3B	1.41622854751541	0.000754030145909347
ENSG00000170684	ZNF296	1.41508572198343	0.000157294127203781
ENSG00000177721	ANXA2R	1.4142106617774	1.70028667619165e-07
ENSG00000140519	RHCG	1.41294440031752	7.73648215538047e-08
ENSG00000255366	N.A.	1.41284324830817	0.000138677276978582
ENSG00000241935	HOGA1	1.40943027463418	2.92557221802364e-15
ENSG00000164362	TERT	1.40911985663032	2.9868736966114e-11
ENSG00000185519	FAM131C	1.39912184809808	1.85772477416044e-09
ENSG00000229644	NAMPTP1	1.39599584694953	0.0053085430917065

ENSG00000135378	PRRG4	1.39377715102996	1.81212684204771e-19
ENSG00000133519	ZDHHC8P1	1.38979516323505	2.2344759651234e-19
ENSG00000117868	ESYT2	1.38760899995773	0
ENSG00000136155	SCEL	1.38756992423205	7.5593619168174e-19
ENSG00000250834	KRT18P54	1.386460927333	4.5400906407305e-06
ENSG00000116574	RHOU	1.38561047535707	6.70292593938642e-57
ENSG00000064300	NGFR	1.38550710388468	0.00579055560010947
ENSG00000102271	KLHL4	1.37251498810411	7.65806267752273e-26
ENSG00000141540	TTYH2	1.36930384593367	0.000599002568532858
ENSG00000171223	JUNB	1.36815114128759	1.91396787654941e-70
ENSG00000173706	HEG1	1.35717217924248	3.78905960035195e-54
ENSG00000223658	C1GALT1C1L	1.35002100468288	0.000188653248354309
ENSG00000174721	FGFBP3	1.34854936664383	2.53698354784955e-10
ENSG00000229619	MBNL1-AS1	1.34646551192986	0.000336130323312539
ENSG00000143590	EFNA3	1.34594324809966	2.02180610834575e-05
ENSG00000067113	PPAP2A	1.34467741837045	1.64579399213261e-96
ENSG00000067082	KLF6	1.34303178701707	2.99052009537688e-166
ENSG00000124782	RREB1	1.34298111046399	1.81675861267901e-82
ENSG00000125319	C17orf53	1.34281428773826	2.07956258985146e-34
ENSG00000100003	SEC14L2	1.34259183295419	7.23076871178297e-13
ENSG00000166922	SCG5	1.34250782009177	5.75289463800264e-06
ENSG00000146966	DENND2A	1.34190792729966	0.0030445138991957
ENSG00000180616	SSTR2	1.34138929838431	2.17932007163588e-05
ENSG00000039139	DNAH5	1.34113450674242	5.10777649786073e-21
ENSG00000120658	ENOX1	1.33689164806544	0.000682062742698377
ENSG00000090565	RAB11FIP3	1.33398429981175	3.36907441301098e-78
ENSG00000155850	SLC26A2	1.33295867995577	9.7640234038712e-122
ENSG00000197405	C5AR1	1.33260161337157	5.42613840216625e-08
ENSG00000155265	GOLGA7B	1.33202297026823	7.92217744638968e-06
ENSG00000136002	ARHGEF4	1.32946336084337	5.77888047948078e-09
ENSG00000070404	FSTL3	1.32192026707339	2.9903516944728e-113
ENSG00000110721	CHKA	1.31869392521248	3.50784113660764e-129
ENSG00000148426	PROSER2	1.31823438382019	2.67201990013569e-52
ENSG00000119771	KLHL29	1.31714361454648	3.94732939537717e-13
ENSG00000106799	TGFBR1	1.31688633766768	8.98285124109175e-153
ENSG00000120899	PTK2B	1.31629679048804	2.01401200759256e-69
ENSG00000183111	ARHGEF37	1.31501335322149	1.73997495516516e-16
ENSG00000204634	TBC1D8	1.31063692973421	3.12402195891524e-172
ENSG00000249430	N.A.	1.30936167602491	0.0065825521671799
ENSG00000132613	MTSS1L	1.309054160201	8.78297369721651e-113
ENSG00000247095	MIR210HG	1.30639960724211	0.00018215756016974
ENSG00000131697	NPHP4	1.30611955476338	1.50184640897533e-35
ENSG00000106785	TRIM14	1.30094800048904	2.15631739427396e-76
ENSG00000105643	ARRDC2	1.29266258101743	8.59129509002696e-63
ENSG00000136630	HLX	1.29108568451099	2.46556531477639e-06
ENSG00000101255	TRIB3	1.28906682230761	8.79427499420805e-149
ENSG00000197586	ENTPD6	1.28819017860477	1.6530729560884e-146
ENSG00000224520	KRT8P45	1.28741941132719	0.000835504282191521
ENSG00000156113	KCNMA1	1.28635652352665	1.78394698420752e-06

ENSG00000172927	MYEOV	1.27685650418987	2.57531850945062e-109
ENSG00000232093	N.A.	1.27355384369413	4.99343644565778e-06
ENSG00000135709	KIAA0513	1.27340414623804	1.34041172915706e-30
ENSG00000160796	NBEAL2	1.27226973012944	1.9368587006978e-87
ENSG00000009709	PAX7	1.26995353737572	0.00082477297512093
ENSG00000128335	APOL2	1.26812488291493	1.23420606376614e-26
ENSG00000152292	SH2D6	1.26346645078348	0.00528164735704591
ENSG00000078018	MAP2	1.2628408035952	2.84971549385451e-08
ENSG00000156675	RAB11FIP1	1.26259417622573	1.61382741855078e-165
ENSG00000138646	HERC5	1.26010521602873	1.07464113027092e-22
ENSG00000170085	SIMC1	1.25768877663616	6.55896807293599e-40
ENSG00000127124	HIVEP3	1.25597339800424	3.7530004107227e-06
ENSG00000129595	EPB41L4A	1.25276380994343	1.56749462150554e-143
ENSG00000104833	TUBB4A	1.25224520794938	1.87280967252827e-53
ENSG00000135480	KRT7	1.24631524518059	1.82474763006495e-185
ENSG00000147862	NFIB	1.24433694746621	5.28980258111689e-63
ENSG00000151689	INPP1	1.24430074991088	6.44687832539332e-21
ENSG00000163399	ATP1A1	1.24297546781566	9.90427644631892e-298
ENSG00000173825	TIGD3	1.24292403939566	0.000452874335010794
ENSG00000135111	TBX3	1.24222480441707	1.72624035804833e-28
ENSG00000072195	SPEG	1.24057834819587	0.000153356616752387
ENSG00000135835	KIAA1614	1.24006253999962	0.00177872796857038
ENSG00000143842	SOX13	1.2389641332204	5.28926752148775e-48
ENSG00000018408	WWTR1	1.23562055863792	2.40415121581808e-185
ENSG00000221968	FADS3	1.23407713339338	2.16561445442073e-36
ENSG00000162174	ASRGL1	1.22022597278793	6.330799109473e-71
ENSG00000155465	SLC7A7	1.21795676080256	1.02109838643973e-60
ENSG00000277938	N.A.	1.21716699071599	6.18606263822178e-07
ENSG00000126351	THRA	1.21558286672002	7.17210439811021e-85
ENSG00000233621	LINC01137	1.21099596700066	8.02284643349685e-11
ENSG00000124762	CDKN1A	1.20845561129529	7.18484385875465e-97
ENSG00000145703	IQGAP2	1.20277061371533	9.49657639889932e-08
ENSG00000234737	KRT18P15	1.20264732814487	0.00502276074795298
ENSG00000268460	N.A.	1.19274333820017	4.12314640946161e-05
ENSG00000151718	WWC2	1.19078074055102	3.92597916657687e-111
ENSG00000064687	ABCA7	1.18946824087052	4.81410770427803e-68
ENSG00000144452	ABCA12	1.18851927991308	5.3647769890752e-17
ENSG00000163644	PPM1K	1.18689704451891	1.78443831114985e-09
ENSG00000164292	RHOBTB3	1.18624426959455	4.56825223535706e-95
ENSG00000167711	SERPINF2	1.18535758522578	5.92397695989985e-16
ENSG00000170421	KRT8	1.18405722215132	1.04684012232064e-170
ENSG00000159216	RUNX1	1.18313889901494	5.78363168703989e-29
ENSG00000114019	AMOTL2	1.18254133838944	5.40546896611885e-89
ENSG00000137801	THBS1	1.18095765650628	8.25219450714735e-174
ENSG00000130720	FIBCD1	1.18042640258377	8.67789031036486e-11
ENSG00000184602	SNN	1.17841683864326	5.3201551168915e-19
ENSG00000165272	AQP3	1.17840494094183	9.37760308684145e-62
ENSG00000179241	LDLRAD3	1.17562436055368	1.6977030489124e-41
ENSG00000138771	SHROOM3	1.17037754843998	2.55436308152479e-82

ENSG00000205485	N.A.	1.16966769264637	6.9124489825611e-05
ENSG00000126947	ARMCX1	1.16825391357493	0.000290271623796906
ENSG00000162520	SYNC	1.16110747727582	1.10172889861899e-15
ENSG00000140323	DISP2	1.1603865062069	0.00304441191261973
ENSG00000129353	SLC44A2	1.15985674584665	1.02265742379983e-51
ENSG00000171227	TMEM37	1.15517726988387	7.18518447899405e-05
ENSG00000124664	SPDEF	1.15273999017284	1.21826734346844e-60
ENSG00000182919	C11orf54	1.15176621982867	5.45798543672333e-53
ENSG00000002746	HECW1	1.14895818653807	2.53444165843683e-27
ENSG00000049759	NEDD4L	1.14606376714066	3.31169855663976e-119
ENSG00000157978	LDLRAP1	1.14386429475543	2.55222895582458e-35
ENSG00000166924	NYAP1	1.14369757950329	0.000138384220015875
ENSG00000121690	DEPDC7	1.14327079331108	3.39847296007075e-24
ENSG00000075426	FOSL2	1.14320508436431	3.3295064180053e-95
ENSG00000103264	FBXO31	1.14038561275299	1.54473079264304e-47
ENSG00000185133	INPP5J	1.13833842642543	0.000449575660126726
ENSG00000196372	ASB13	1.13354429234138	4.39676603877308e-36
ENSG00000175764	TTLL11	1.12891616725349	0.000302353320639356
ENSG00000072832	CRMP1	1.12823627203343	4.59297893753839e-05
ENSG00000166979	EVA1C	1.12715262661468	4.68126429760516e-05
ENSG00000176438	SYNE3	1.12638491454063	0.0089448906847655
ENSG00000132763	MMACHC	1.12353370718473	3.49600700768978e-07
ENSG00000095637	SORBS1	1.12236980547675	3.49490543038699e-08
ENSG00000156515	HK1	1.12084222628289	5.61813117546065e-78
ENSG00000111962	UST	1.12032952790374	1.51296323587124e-07
ENSG00000086062	B4GALT1	1.11669790828047	1.64206278311947e-231
ENSG00000162434	JAK1	1.11238215736278	1.48932471268435e-265
ENSG00000112394	SLC16A10	1.10929783064859	0.0002760266208935
ENSG00000180354	MTURN	1.10845307044818	1.2835515209027e-08
ENSG00000174669	SLC29A2	1.10603166647915	1.47465797445942e-50
ENSG00000162946	DISC1	1.10551023157452	0.00150372692292067
ENSG00000179954	SSC5D	1.1053958675456	0.00630661704733524
ENSG00000111057	KRT18	1.10239361994169	1.22613578049042e-145
ENSG00000187550	SBK2	1.10139697765675	1.87241181367216e-13
ENSG00000165029	ABCA1	1.10025113125167	5.67994066948195e-10
ENSG00000166250	CLMP	1.09797947610872	7.62533616899969e-05
ENSG00000160685	ZBTB7B	1.09747642437843	1.42875291330713e-65
ENSG00000261971	MMP25-AS1	1.09470597011793	2.23778505344995e-06
ENSG00000163702	IL17RC	1.09170629472265	5.19197999190845e-28
ENSG00000165959	CLMN	1.09099959983592	9.11783966864459e-115
ENSG00000081913	PHLPP1	1.08809878352038	3.7487049377933e-35
ENSG00000121210	KIAA0922	1.08612592712279	8.44333601408683e-19
ENSG00000125266	EFNB2	1.08306389968837	9.34345427565652e-08
ENSG00000184669	OR7E14P	1.08259361016149	0.000253372505317457
ENSG00000186174	BCL9L	1.07974614521101	4.02724950384497e-118
ENSG00000011347	SYT7	1.07930195049442	4.2026905463235e-05
ENSG00000206190	ATP10A	1.07835109134882	1.55244695296444e-29
ENSG00000019144	PHLDB1	1.07697157876201	1.95485911185472e-46
ENSG00000182389	CACNB4	1.07651854668644	0.00854926128748107

ENSG00000114790	ARHGEF26	1.07531179814641	2.53934016049019e-41
ENSG00000073350	LLGL2	1.07482435309316	8.68657078960394e-83
ENSG00000163875	MEAF6	1.07070625483629	9.16609814325474e-46
ENSG00000136830	FAM129B	1.0699588736596	1.15448023479798e-216
ENSG00000198873	GRK5	1.06376857068022	1.58743829148272e-16
ENSG00000086544	ITPKC	1.05995889477916	4.37665422378031e-38
ENSG00000168234	TTC39C	1.05893523143672	4.70967449850642e-13
ENSG00000232434	C9orf172	1.05516015889129	1.26201668613461e-08
ENSG00000213694	S1PR3	1.05446716579786	3.04063463599331e-14
ENSG00000198648	STK39	1.05286402094743	8.75478859785842e-40
ENSG00000183044	ABAT	1.051356505535	0.00706000847324075
ENSG00000176597	B3GNT5	1.05000285991434	4.78681039532181e-39
ENSG00000164938	TP53INP1	1.04800225110923	6.57959089267769e-34
ENSG00000103253	HAGHL	1.04612741488313	8.99384410823368e-05
ENSG00000071205	ARHGAP10	1.04287333520878	2.30897245764395e-25
ENSG00000162614	NEXN	1.04054933220033	2.4264117290668e-07
ENSG00000169905	TOR1AIP2	1.03758338256872	3.73190455595439e-111
ENSG00000170921	TANC2	1.03661730378777	1.79313816774768e-68
ENSG00000105662	CRTC1	1.03251654599427	2.0973400468859e-14
ENSG00000004660	CAMKK1	1.03114217557988	1.39826678200177e-26
ENSG00000141314	RHBDL3	1.02840846521558	7.96835831655249e-06
ENSG00000103429	BFAR	1.0264108340554	5.40490479783536e-107
ENSG00000106351	AGFG2	1.02420136518982	1.00215506123224e-61
ENSG00000169213	RAB3B	1.01131408921011	1.95207616111763e-43
ENSG00000168118	RAB4A	1.00691559937283	8.15498050820839e-49
ENSG00000167994	RAB3IL1	1.00169992082184	4.50545011756455e-09
ENSG00000108821	COL1A1	-1.00008822920517	5.25847936738138e-09
ENSG00000217801	N.A.	-1.00210992386299	0.00669028315275732
ENSG00000151229	SLC2A13	-1.00226866573418	3.46240629459532e-29
ENSG00000168772	CXXC4	-1.005359248689	2.10833492648052e-10
ENSG00000130675	MNX1	-1.00674373659521	9.96706386433581e-10
ENSG00000118407	FILIP1	-1.01113995851043	5.30624839445459e-05
ENSG00000100292	HMOX1	-1.01154651399219	6.35037909197757e-70
ENSG00000154822	PLCL2	-1.01356520624086	1.50153193299648e-18
ENSG00000163449	TMEM169	-1.01454798834443	0.00488639264672251
ENSG00000153234	NR4A2	-1.01546927410032	0.000748044336573094
ENSG00000141668	CBLN2	-1.01558211982984	0.0034473906406286
ENSG00000130522	JUND	-1.01590978378614	2.10070293945882e-37
ENSG00000012504	NR1H4	-1.01623517621774	0.000280472565766175
ENSG00000272068	N.A.	-1.01638616307205	1.99988623080964e-14
ENSG00000099337	KCNK6	-1.01800719748616	8.02416851509552e-08
ENSG00000196639	HRH1	-1.0194852614395	1.85752712321704e-05
ENSG00000176771	NCKAP5	-1.02205486837625	7.046022720058e-16
ENSG00000171617	ENC1	-1.02598186266967	2.84034009135601e-36
ENSG00000132003	ZSWIM4	-1.02846587812895	9.73086652201627e-07
ENSG00000196535	MYO18A	-1.02871016067633	5.97979948965014e-94
ENSG00000131725	WDR44	-1.03601852747238	1.0459601410791e-57
ENSG00000100092	SH3BP1	-1.03647223639712	0.000191638940745354
ENSG00000272405	N.A.	-1.03766821625698	1.64793840914315e-26

ENSG00000253669	N.A.	-1.03818682164085	6.94514398196599e-06
ENSG00000138449	SLC40A1	-1.03846226332352	6.1708999795987e-34
ENSG00000198732	SMOC1	-1.03869460408183	0.000126249295282574
ENSG00000147324	MFHAS1	-1.03910791923109	5.01637634069535e-60
ENSG00000082497	SERTAD4	-1.03961607494224	0.00395156973643558
ENSG00000120756	PLS1	-1.03986363420239	4.18722854199832e-100
ENSG00000135905	DOCK10	-1.0402159690358	3.7568578308972e-33
ENSG00000167306	MYO5B	-1.04121884804807	1.64202169651302e-10
ENSG00000250799	PRODH2	-1.04546352952618	6.2878065455786e-08
ENSG00000115919	KYNU	-1.04653467481178	7.30518643100016e-96
ENSG00000174939	ASPHD1	-1.04680292401455	0.000423853424764639
ENSG00000140332	TLE3	-1.04934963973076	1.43633518018239e-28
ENSG00000215182	MUC5AC	-1.0513960751769	5.35675261659872e-21
ENSG00000171346	KRT15	-1.05299521908611	0.00500367927599078
ENSG00000185149	NPY2R	-1.05711678165326	1.26877453724417e-06
ENSG00000140297	GCNT3	-1.05792389035735	1.37397571416602e-108
ENSG00000125170	DOK4	-1.05901507759012	5.78117007541853e-88
ENSG00000144366	GULP1	-1.05977432747521	3.56574962050832e-43
ENSG00000070961	ATP2B1	-1.06326249389322	2.13427684944545e-149
ENSG00000139946	PELI2	-1.06435223939222	4.40362925361106e-15
ENSG00000198758	EPS8L3	-1.06436119996862	1.4368120788355e-06
ENSG00000242574	HLA-DMB	-1.06826362383928	5.7604396968406e-57
ENSG00000006468	ETV1	-1.07072825441495	1.86632164801411e-27
ENSG00000112183	RBM24	-1.07130395025189	1.7115117492971e-20
ENSG00000144959	NCEH1	-1.0744108319765	5.74856777042345e-106
ENSG00000089127	OAS1	-1.08173488650841	5.07537936457021e-50
ENSG00000143457	GOLPH3L	-1.08531377723354	2.61231782518193e-65
ENSG00000197822	OCLN	-1.08594246732196	1.01742751666377e-63
ENSG00000134253	TRIM45	-1.08602065852878	1.15765423342119e-08
ENSG00000179715	PCED1B	-1.08709317683944	3.25241815736165e-33
ENSG00000114346	ECT2	-1.08758507964508	2.08928359791859e-162
ENSG00000137393	RNF144B	-1.09243754338747	0.00078349599568752
ENSG00000164823	OSGIN2	-1.0946410405824	7.48400057023949e-84
ENSG00000064651	SLC12A2	-1.09498745692717	4.07089693014399e-149
ENSG00000177283	FZD8	-1.09601847345133	2.69382231365753e-09
ENSG00000103534	TMC5	-1.09627640062753	6.33398682326232e-26
ENSG00000134508	CABLES1	-1.09908215717755	1.57550742006133e-19
ENSG00000164220	F2RL2	-1.09942443023351	8.84270015232182e-05
ENSG00000170537	TMC7	-1.09982801176321	4.7860091774392e-15
ENSG00000270157	N.A.	-1.10220371067526	2.83488483502023e-05
ENSG00000130449	ZSWIM6	-1.10989884088049	8.13983260126003e-33
ENSG00000163491	NEK10	-1.11031009397377	6.09489545718744e-07
ENSG00000181649	PHLDA2	-1.11261162214756	6.30855221505478e-12
ENSG00000117226	GBP3	-1.11463700187418	4.48345375529189e-06
ENSG00000124429	POF1B	-1.11512068026032	1.20298996378189e-42
ENSG00000033327	GAB2	-1.11527175583573	2.05357754900668e-21
ENSG00000127152	BCL11B	-1.11711041482562	0.00437953434432833
ENSG00000197798	FAM118B	-1.11985644247509	2.12652523907455e-26
ENSG00000129514	FOXA1	-1.12035977100441	9.42842660343998e-49

ENSG00000232324	N.A.	-1.12441134132667	0.00391852011182452
ENSG00000121578	B4GALT4	-1.13166806433288	1.61498948310418e-102
ENSG00000124766	SOX4	-1.13893624521811	6.28540718150306e-70
ENSG00000163565	IFI16	-1.14361644071196	0.00181547055943933
ENSG00000170959	DCDC1	-1.14965310951197	0.0080477738802358
ENSG00000128641	MYO1B	-1.15074270854977	1.35813783296665e-87
ENSG00000130477	UNC13A	-1.15100716719975	2.91794331231689e-07
ENSG00000135083	CCNJL	-1.15470590189869	4.96277880629003e-48
ENSG00000172572	PDE3A	-1.16481041925722	2.22641566512701e-42
ENSG00000070526	ST6GALNAC1	-1.17200600059659	2.77547771679971e-06
ENSG00000150054	MPP7	-1.17681804058558	3.99921854992002e-05
ENSG00000146859	TMEM140	-1.17738407595918	0.00519069187097868
ENSG00000168646	AXIN2	-1.18014302262446	2.30290582523044e-06
ENSG00000110723	EXPH5	-1.18335352158526	1.00193781550924e-07
ENSG00000164342	TLR3	-1.18872329187598	0.000478871294953166
ENSG00000183023	SLC8A1	-1.19067485755525	3.91215888712054e-19
ENSG00000144847	IGSF11	-1.201189273528	2.62088694343159e-15
ENSG00000188064	WNT7B	-1.20859854994718	4.2371611752894e-140
ENSG00000162552	WNT4	-1.20916965739395	0.00272447571458739
ENSG00000146072	TNFRSF21	-1.20972378879507	3.13984491715173e-123
ENSG00000006283	CACNA1G	-1.21100140110338	5.24726949625295e-52
ENSG00000091844	RGS17	-1.21406960578118	3.63889048991284e-27
ENSG00000166974	MAPRE2	-1.21862566417721	4.69345735023897e-94
ENSG00000189143	CLDN4	-1.22053404930896	0.000252862008796998
ENSG00000001084	GCLC	-1.22248514006112	7.94546555379463e-209
ENSG00000132639	SNAP25	-1.2252835351171	2.61962184266447e-10
ENSG00000152527	PLEKHH2	-1.22850259117949	1.7749073081624e-72
ENSG00000090339	ICAM1	-1.22963306565127	0.00227059889885476
ENSG00000171310	CHST11	-1.23213170776094	3.39854433659296e-35
ENSG00000160888	IER2	-1.23341344840438	3.65515195359195e-62
ENSG00000198431	TXNRD1	-1.23786441537705	0
ENSG00000221995	TIAF1	-1.23810576561717	0.00208497004358566
ENSG00000167676	PLIN4	-1.24100562282075	0.0027967555164083
ENSG00000104312	RIPK2	-1.24186446934304	1.5971594348231e-67
ENSG00000164690	SHH	-1.24278038987793	0.000533177073506522
ENSG00000107968	MAP3K8	-1.24396941531808	5.20666163012045e-92
ENSG00000265962	GACAT2	-1.24506366730448	3.31728960372804e-25
ENSG00000077150	NFKB2	-1.24620186164427	5.96294392098543e-32
ENSG00000144857	BOC	-1.24761559545249	0.00367708742684505
ENSG00000071282	LMCD1	-1.24792195604445	1.7350592458043e-60
ENSG00000171658	N.A.	-1.24929489707797	2.311043249541e-19
ENSG00000145632	PLK2	-1.2504192907933	4.57412507429305e-51
ENSG00000197566	ZNF624	-1.25207077265273	3.16980086304723e-08
ENSG00000114771	AADAC	-1.25261411388783	6.23800980166542e-18
ENSG00000137449	CPEB2	-1.25405015575827	2.3469668495945e-30
ENSG00000116833	NR5A2	-1.2561423790656	3.7957632479994e-39
ENSG00000071991	CDH19	-1.25621602421087	0.000660342801400494
ENSG00000165929	TC2N	-1.25884095275176	5.6217194267816e-21
ENSG00000106546	AHR	-1.26002552898592	9.80949930638587e-119

ENSG00000108448	TRIM16L	-1.26168772150569	2.02581056990565e-120
ENSG00000075651	PLD1	-1.26203793097985	4.45777082068664e-52
ENSG00000109321	AREG	-1.26304849904255	8.53546492893969e-45
ENSG00000168237	GLYCTK	-1.26320592528907	1.41357195407488e-15
ENSG00000266094	RASSF5	-1.26325108351361	1.91191646565656e-10
ENSG00000139865	TTC6	-1.2641375433434	0.000266471508894761
ENSG00000156026	MCU	-1.2642876023599	6.7321087980128e-166
ENSG00000175832	ETV4	-1.26453756066042	1.48977802959597e-89
ENSG00000064787	BCAS1	-1.26605326428509	4.83883000525354e-48
ENSG00000143816	WNT9A	-1.26627183177843	1.06461466236423e-14
ENSG00000278709	NKILA	-1.26761499968865	1.84122960158104e-07
ENSG00000170485	NPAS2	-1.26924839073403	1.28330398045211e-104
ENSG00000174130	TLR6	-1.27467736332127	5.69095544688457e-24
ENSG00000181143	MUC16	-1.27819333373416	2.11511924926322e-68
ENSG00000184584	TMEM173	-1.28680874350455	3.00658943870146e-05
ENSG00000140859	KIFC3	-1.28789482466561	2.54962844564062e-157
ENSG00000135766	EGLN1	-1.28849252966183	3.16424236644848e-138
ENSG00000143507	DUSP10	-1.28859335144199	9.94122201142614e-08
ENSG00000125398	SOX9	-1.28890195465255	3.13718417898885e-51
ENSG00000125848	FLRT3	-1.29284157457666	3.83620203312442e-76
ENSG00000125798	FOXA2	-1.29832288621104	7.6417859319017e-44
ENSG00000167767	KRT80	-1.29958830894517	4.70084728606162e-112
ENSG00000147041	SYTL5	-1.30452216935063	0.00181492805987428
ENSG00000271303	SRXN1	-1.31796563179581	0.00811892145703237
ENSG00000003137	CYP26B1	-1.32131513519457	2.28468977973462e-21
ENSG00000064989	CALCRL	-1.32265825387912	0.000627887589725242
ENSG00000204956	PCDHGA1	-1.32391872451827	0.000244750302373466
ENSG00000125657	TNFSF9	-1.32596291117144	9.59344919621272e-36
ENSG00000236095	N.A.	-1.32699559765121	0.000158280147547979
ENSG00000167771	RCOR2	-1.32719648122172	0.000386554865013336
ENSG00000125772	GPCPD1	-1.33247649739605	3.83198445934099e-194
ENSG00000227640	SOX21-AS1	-1.33301957177678	9.67700249405813e-05
ENSG00000095739	BAMBI	-1.33536229628761	9.17518909480976e-25
ENSG00000163734	CXCL3	-1.33651920176391	0.000326283541612323
ENSG00000152580	IGSF10	-1.33708728903406	1.45972861563176e-21
ENSG00000135525	MAP7	-1.33782242181878	4.23917809149357e-119
ENSG00000135362	PRR5L	-1.33994545966394	3.71641401127597e-11
ENSG00000177875	CCDC184	-1.343852568539	2.97208832239505e-16
ENSG00000171631	P2RY6	-1.34542096007535	8.02123540518586e-20
ENSG00000197063	MAFG	-1.34997109348426	7.0102610177946e-130
ENSG00000166963	MAP1A	-1.36016393052509	5.89949173919224e-07
ENSG00000164742	ADCY1	-1.37725250173631	5.66741963725644e-85
ENSG00000114541	FRMD4B	-1.38081848306132	3.23258841726252e-27
ENSG00000203943	SAMD13	-1.38287252636562	0.00501571822470056
ENSG00000001626	CFTR	-1.3936920933055	0.00591658765811222
ENSG00000111087	GLI1	-1.39714320741242	1.33039757348638e-26
ENSG00000168453	HR	-1.40434388064074	8.89874629469155e-44
ENSG00000078401	EDN1	-1.41548668765134	1.28943875069885e-92
ENSG00000163435	ELF3	-1.43096886220906	1.78890509720196e-236

ENSG00000147650	LRP12	-1.43483615771957	2.39739572954871e-67
ENSG00000146674	IGFBP3	-1.43802563968509	4.69384085054634e-85
ENSG00000196628	TCF4	-1.43887989140563	7.46116597324015e-23
ENSG00000262001	DLGAP1-AS2	-1.44482383839456	6.06397337529902e-11
ENSG00000168672	FAM84B	-1.44552708252697	8.28108005529891e-37
ENSG00000100557	C14orf105	-1.45520289187719	4.9796439470259e-40
ENSG00000171431	KRT20	-1.45918337947375	8.82782138574034e-13
ENSG00000197122	SRC	-1.46124902567882	3.23221091737336e-191
ENSG00000197261	C6orf141	-1.4644083845389	3.36673651218979e-18
ENSG00000198517	MAFK	-1.47153782209509	2.62014365108609e-131
ENSG00000132669	RIN2	-1.47336700972535	2.78071484924048e-60
ENSG00000075213	SEMA3A	-1.47390176620886	5.57525554353724e-92
ENSG00000249199	N.A.	-1.47658482043433	0.00175847265344364
ENSG00000196668	LINC00173	-1.48144474376311	1.02008059373561e-06
ENSG00000157613	CREB3L1	-1.48171866478711	4.51358819528685e-34
ENSG00000137962	ARHGAP29	-1.48622633589799	4.06487583731559e-125
ENSG00000048052	HDAC9	-1.48703494060963	0.00300607779388028
ENSG00000261652	C15orf65	-1.48938862998419	4.37915479302597e-08
ENSG00000144802	NFKBIZ	-1.49643760155667	3.17179627411964e-42
ENSG00000005844	ITGAL	-1.49726245653392	0.00733176402562224
ENSG00000146038	DCDC2	-1.49863861838118	5.98214991632734e-91
ENSG00000134138	MEIS2	-1.49935939569436	2.46106682394734e-90
ENSG00000161011	SQSTM1	-1.50024012264318	0
ENSG00000185477	GPRIN3	-1.50695716000757	1.3182656575907e-162
ENSG00000135114	OASL	-1.5110542384646	0.00815652369689857
ENSG00000188906	LRRK2	-1.51625388979485	1.37630193721873e-103
ENSG00000171877	FRMD5	-1.52112059867781	2.3457251220455e-10
ENSG00000128342	LIF	-1.52688274105791	3.66438559739192e-33
ENSG00000249464	LINC01091	-1.52946968173714	0.00015619502417315
ENSG00000159263	SIM2	-1.53211342850613	4.8944915975712e-24
ENSG00000136881	BAAT	-1.53587242379797	1.54516838705432e-08
ENSG00000250271	N.A.	-1.53688901566142	2.52012721241572e-05
ENSG00000129521	EGLN3	-1.55092181176152	1.84624528420517e-08
ENSG00000179776	CDH5	-1.55513844383138	0.00468877202600139
ENSG00000171408	PDE7B	-1.5555192129632	3.70268102971074e-07
ENSG00000139971	C14orf37	-1.5571472953439	0.00364084786387712
ENSG00000240602	AADACP1	-1.58754451211078	1.15629406288608e-30
ENSG00000104856	RELB	-1.59421871443228	3.71134615841175e-14
ENSG00000120549	KIAA1217	-1.61614367503438	1.25739107635532e-39
ENSG00000172164	SNTB1	-1.63372562936481	5.66326509443481e-40
ENSG00000223414	LINC00473	-1.63557537293576	3.25533106663456e-62
ENSG00000248323	LUCAT1	-1.63578320058536	8.78082802880514e-15
ENSG00000005981	ASB4	-1.636296460086	0.000106191210304152
ENSG00000151474	FRMD4A	-1.65034099155012	2.06400766409265e-42
ENSG00000080493	SLC4A4	-1.67181943911664	1.16594208259359e-64
ENSG00000104043	ATP8B4	-1.67336432180145	6.54944069668796e-06
ENSG00000124882	EREG	-1.68279347969929	1.35384936547221e-90
ENSG00000023909	GCLM	-1.68467074585335	1.15575695321368e-166
ENSG00000248807	KRTAP9-12P	-1.68490550380978	1.08242612877954e-05

ENSG00000005471	ABCB4	-1.68493067662871	0.00982696406574526
ENSG00000119547	ONECUT2	-1.69324957928009	3.88760003185239e-26
ENSG00000125285	SOX21	-1.69375037963889	2.18035550118557e-07
ENSG00000114315	HES1	-1.69434494756896	1.74292853004885e-17
ENSG00000067798	NAV3	-1.69438757873494	2.6845611059403e-20
ENSG00000162878	PKDCC	-1.69530751479611	1.8461307200415e-126
ENSG00000163110	PDLIM5	-1.69871614711726	0
ENSG00000137440	FGFBP1	-1.71362697236474	8.7498918734685e-16
ENSG00000164488	DACT2	-1.73153045691819	7.79392277450449e-07
ENSG00000173702	MUC13	-1.74567172229604	7.66391030424195e-288
ENSG00000171303	KCNK3	-1.7458445285394	0.00149274787796276
ENSG00000125378	BMP4	-1.75266887368148	3.30848801041839e-29
ENSG00000153292	ADGRF1	-1.77860623035556	0.00213011836925878
ENSG00000234231	N.A.	-1.78126724029919	0.00709960763616062
ENSG00000092969	TGFB2	-1.78323515035763	1.82103875745313e-92
ENSG00000273706	LHX1	-1.79249355989022	5.03332846355319e-05
ENSG00000139679	LPAR6	-1.80719430368476	2.28649827935486e-12
ENSG00000137331	IER3	-1.80729889390219	4.43360354979459e-108
ENSG00000273129	PACERR	-1.82578713933042	0.00275343301090087
ENSG00000128917	DLL4	-1.83179040608624	4.26711400444949e-16
ENSG00000272502	N.A.	-1.83360801473312	0.00248385905833971
ENSG00000125462	C1orf61	-1.83435094275407	0.000240458533385359
ENSG00000145358	DDIT4L	-1.84517531823377	1.910383238821e-13
ENSG00000154678	PDE1C	-1.86873380904253	2.91844432835102e-81
ENSG00000080031	PTPRH	-1.87640579937184	4.67410743916791e-26
ENSG00000169297	NR0B1	-1.87824542010339	2.03634487587893e-53
ENSG00000118513	MYB	-1.88457907814315	2.64858631970774e-05
ENSG00000110693	SOX6	-1.89777831023878	9.5180075042224e-11
ENSG00000144583	MARCH4	-1.90863142027267	0.000169692128813625
ENSG00000151617	EDNRA	-1.91430785343743	1.11630072905635e-08
ENSG00000170961	HAS2	-1.92399540897374	6.39502295064234e-11
ENSG00000166387	PPFIBP2	-1.92765948688969	7.07882884297325e-70
ENSG00000120217	CD274	-1.9294368270204	2.55695542154953e-12
ENSG00000173917	HOXB2	-1.93886532206805	3.37367768008009e-05
ENSG00000120875	DUSP4	-1.93919417172054	0
ENSG00000275578	N.A.	-1.94544303505583	9.74015039815828e-06
ENSG00000144339	TMEFF2	-1.96498056324021	1.46188131141e-09
ENSG00000166750	SLFN5	-1.99432070249825	5.2320341354250e-82
ENSG00000182580	EPHB3	-2.00497079231959	6.67295422893724e-05
ENSG00000184545	DUSP8	-2.0135750560388	1.77567158641244e-11
ENSG00000137501	SYTL2	-2.02944189029118	5.97453462772637e-21
ENSG00000236345	N.A.	-2.04136973280419	0.00378281053161491
ENSG00000073282	TP63	-2.04266867455992	0.000600072085302715
ENSG00000253227	N.A.	-2.04551300584278	0.00520514433131372
ENSG00000164949	GEM	-2.05276842185262	1.44080471487821e-33
ENSG00000113361	CDH6	-2.06607334756295	3.30283495466616e-15
ENSG00000118526	TCF21	-2.07166813260839	1.6062785053756e-06
ENSG00000007944	MYLIP	-2.08551218998335	3.14608549406668e-43
ENSG00000203727	SAMD5	-2.10273155580717	3.25329538440291e-22

ENSG00000172296	SPTLC3	-2.10321386660197	1.70873822396659e-60
ENSG00000183778	B3GALT5	-2.12024368115531	8.46043759075464e-10
ENSG00000134363	FST	-2.12197716577717	1.30245508240412e-43
ENSG00000151623	NR3C2	-2.12263226260071	4.03337107952595e-19
ENSG00000002587	HS3ST1	-2.13245573718969	8.95851924040148e-06
ENSG00000163739	CXCL1	-2.14510921193093	4.6569418632651e-07
ENSG00000179674	ARL14	-2.14805948086686	0.00100232764477053
ENSG00000095752	IL11	-2.15790919155038	1.19380385922913e-10
ENSG00000144057	ST6GAL2	-2.15920814968005	1.06182006753804e-12
ENSG00000138735	PDE5A	-2.1730774366937	2.09792519010974e-81
ENSG00000181773	GPR3	-2.17544396902979	0.00147874283734481
ENSG00000076864	RAP1GAP	-2.17793799489131	1.84744925698967e-256
ENSG00000198443	KRTAP4-1	-2.19076627592228	0.00864536132211332
ENSG00000245750	DRAIC	-2.20556768298649	1.00347251224664e-15
ENSG00000163735	CXCL5	-2.20990030165836	8.71722142481925e-94
ENSG00000140961	OSGIN1	-2.21786588008027	5.70634185581304e-144
ENSG00000272168	CASC15	-2.24109573339345	8.36547486576099e-09
ENSG00000167105	TMEM92	-2.24462036332696	2.05742888066486e-48
ENSG00000181449	SOX2	-2.25782996508392	3.31510323541525e-73
ENSG00000119508	NR4A3	-2.26209720487807	4.08456761978576e-12
ENSG00000248771	LINC01207	-2.30089974172826	2.8541279161408e-15
ENSG00000175147	TMEM51-AS1	-2.30137546497837	0.000244147198309437
ENSG00000185015	CA13	-2.31215615872866	0.00359146600596682
ENSG00000151012	SLC7A11	-2.33550482667964	0
ENSG00000173114	LRRN3	-2.40376880588726	1.95419591400225e-07
ENSG00000164330	EBF1	-2.41493676012774	0.0011417743808006
ENSG00000152689	RASGRP3	-2.42288115731338	7.4177653887743e-08
ENSG00000179046	TRIML2	-2.42864603467743	9.87710881884012e-107
ENSG00000170608	FOXA3	-2.48909353818373	2.19779644557305e-20
ENSG00000107957	SH3PXD2A	-2.51865641316223	1.80587487218568e-123
ENSG00000087494	PTHLH	-2.52518478049057	1.7317687417434e-49
ENSG00000187185	N.A.	-2.65281049830759	9.76562400645012e-26
ENSG00000073756	PTGS2	-2.65304658993217	0
ENSG00000259974	LINC00261	-2.7240985512583	1.53835899070101e-27
ENSG00000158055	GRHL3	-2.74373084178706	3.07539760955811e-14
ENSG00000162772	ATF3	-2.82002613936926	6.08479781730901e-43
ENSG00000006210	CX3CL1	-2.86998582124456	1.06129046456986e-16
ENSG00000019991	HGF	-2.87183416329063	1.12421230414746e-05
ENSG00000152049	KCNE4	-2.90120811801384	3.45296251701642e-46
ENSG00000235269	N.A.	-2.90876473350042	0.00116166723466579
ENSG00000168955	TM4SF20	-2.94318327037641	1.01168573437766e-29
ENSG00000174125	TLR1	-2.95101032273138	2.17984566412527e-10
ENSG00000113532	ST8SIA4	-2.95644649779586	6.0017597750182e-54
ENSG00000174123	TLR10	-2.9797780437214	0.00239235269991512
ENSG00000142623	PADI1	-3.02183608635328	4.03782001392857e-25
ENSG00000175920	DOK7	-3.13916411525023	7.65371530336619e-15
ENSG00000172602	RND1	-3.25695263560521	1.5329271216607e-83
ENSG00000126262	FFAR2	-3.43313964740344	0.00705300996922918
ENSG00000178033	FAM26E	-3.45969708267635	0.000791719571968729

ENSG00000198829	SUCNR1	-3.67499301169258	1.42926151080766e-35
ENSG00000019186	CYP24A1	-3.80193413295353	0
ENSG00000234155	N.A.	-3.8157787143382	6.90993033273043e-15
ENSG00000180875	GREM2	-3.93043603630217	0.00197748448971785
ENSG00000181634	TNFSF15	-4.17019919098431	3.41154679924567e-15
ENSG00000277297	ATP5A1P10	-4.58510047793385	0.000703358944785062
ENSG00000130513	GDF15	-4.80138698296999	6.40570429977758e-298
ENSG00000134258	VTCN1	-5.1683119738877	2.38034242332496e-05
ENSG00000180861	LINC01559	-5.57752653656322	2.48438211930741e-06
ENSG00000189431	RASSF10	-5.76684453740881	1.35853049959754e-109
ENSG00000164761	TNFRSF11B	-6.57524234785405	7.58068999143608e-09
