# Supplementary material for: The nature of the GRE influences the screening for GR-activity enhancing modulators
Source: PLoS One. 2017 Jul 7;12(7):e0181101. doi: 10.1371/journal.pone.0181101 (PMC5501670; doi:10.1371/journal.pone.0181101)
Supplement: S2 Table — (PDF) [file pone.0181101.s002.pdf]

List of genes differentially expressed after Belinostat (|LFC| >1; adjusted p-val < 0.01)

Ensembl id	Symbol	LFC	adj. p-value
ENSG00000273777	CEACAM20	11.9312016294529	1.71224662888516e-05
ENSG00000235643	N.A.	11.7791109287975	2.22420140240024e-05
ENSG00000183813	CCR4	11.3924643507922	4.29976139184247e-05
ENSG00000118971	CCND2	11.3826036025218	4.38861698973884e-05
ENSG00000177464	GPR4	11.3633218356627	4.51800949826403e-05
ENSG00000250584	LINC01511	11.3385210938963	4.67479801397408e-05
ENSG00000112796	ENPP5	11.1726444229895	6.19727006845895e-05
ENSG00000105784	RUNDC3B	11.0001338418549	8.19600017081753e-05
ENSG00000177807	KCNJ10	10.9264112149642	9.29088091410079e-05
ENSG00000140600	SH3GL3	10.8903428651244	9.91289510660312e-05
ENSG00000008056	SYN1	10.8115925841594	0.000112293695881903
ENSG00000177098	SCN4B	10.7714040980004	0.000118523053162956
ENSG00000248810	N.A.	10.6032670923367	0.000154830831254639
ENSG00000248485	PCP4L1	10.4910773762688	0.000185951316887104
ENSG00000107317	PTGDS	10.4845367889724	0.00018802578515111
ENSG00000266117	FBXO36P1	10.3717145011619	0.000224460018252867
ENSG00000160282	FTCD	10.2922231006667	0.000252428109887177
ENSG00000156298	TSPAN7	10.2887962394333	0.00026003091995239
ENSG00000168491	CCDC110	10.2579671038939	0.000267955902213358
ENSG00000164741	DLC1	10.229116342242	0.000281018162827282
ENSG00000077264	PAK3	10.2083622771157	0.000291641068513115
ENSG00000115896	PLCL1	10.186841066816	0.000298066279272022
ENSG00000250198	N.A.	10.1197134843408	0.000333027508085851
ENSG00000259033	N.A.	10.1116177830976	0.000339036330971052
ENSG00000249849	N.A.	10.0953600934001	0.000346591849827438
ENSG00000174521	TTC9B	10.0347260485985	0.000376177619290761
ENSG00000265217	N.A.	9.98188297711532	0.000410305026708449
ENSG00000182040	USH1G	9.93912805173087	0.000433307433112586
ENSG00000237693	IRGM	9.92029959874598	0.000454683471177303
ENSG00000165194	PCDH19	9.91966009711877	0.000446979984022934
ENSG00000182578	CSF1R	9.91266331830187	0.000460583600928192
ENSG00000171954	CYP4F22	9.8970796509451	0.000451480189770176
ENSG00000174255	ZNF80	9.8703078987978	0.000480424020915559
ENSG00000243978	RGAG1	9.83693080976155	0.000504377021385682
ENSG00000129244	ATP1B2	9.73732205930155	2.09113106109573e-30
ENSG00000221818	EBF2	9.73536084766046	0.000598187100457578
ENSG00000168658	VWA3B	9.71029855407483	0.000604022467683185
ENSG00000002745	WNT16	9.62711928301879	0.00064771278126732
ENSG00000174844	DNAH12	9.60731383147732	3.56989633366881e-20
ENSG00000140986	RPL3L	9.60552530244894	1.14866496464401e-29
ENSG00000259617	N.A.	9.58425018584442	0.000742143685385488
ENSG00000206172	HBA1	9.58316502926465	0.000729551915179751
ENSG00000164694	FNDC1	9.54059119614816	0.000789949546003413
ENSG00000248431	N.A.	9.53737668803972	0.000804880134257493
ENSG00000178297	TMPRSS9	9.52762082630953	1.77345671102438e-10
ENSG00000223831	N.A.	9.52051995762679	0.000799638981761229

ENSG00000162706	CADM3	9.34581425923555	0.00106173405590388
ENSG00000188536	HBA2	9.3369351288242	0.0010571512455032
ENSG00000101331	CCM2L	9.32363206083867	0.00105923723285399
ENSG00000186517	ARHGAP30	9.22623274890926	0.00125093150003852
ENSG00000132026	RTBDN	9.16269707599613	9.10985803568472e-10
ENSG00000092051	JPH4	9.10482277818274	0.00138392232177531
ENSG00000105675	ATP4A	9.08567230863629	0.00146916564709175
ENSG00000245598	DACT3-AS1	9.06568685382314	0.00155736602133634
ENSG00000215120	N.A.	9.01788373571893	0.00161522762900257
ENSG00000104722	NEFM	9.01537188736375	0.00165841018753055
ENSG00000187987	ZSCAN23	9.00112193373425	0.00168051728069901
ENSG00000198483	ANKRD35	8.97160183718987	0.00174472171782871
ENSG00000072657	TRHDE	8.9633885145422	0.00180366875007372
ENSG00000273802	HIST1H2BG	8.94506704890628	0.00185386846315776
ENSG00000164746	C7orf57	8.93659281025982	2.49805066197051e-09
ENSG00000167749	KLK4	8.93486743370257	0.00182242733178192
ENSG00000137473	TTC29	8.89456648079829	0.00201476175082063
ENSG00000187017	ESPN	8.85711314695117	3.47527219046255e-09
ENSG00000102195	GPR50	8.83191801583057	0.0021572148907587
ENSG00000147234	FRMPD3	8.82943773898898	0.00213307212301531
ENSG00000167414	GNG8	8.81821769661186	0.00218057413992685
ENSG00000113196	HAND1	8.8048346964066	0.0021628630444684
ENSG00000268223	ARL14EPL	8.78166143760849	0.0023266982941254
ENSG00000128040	SPINK2	8.73481613854833	0.00242438934085118
ENSG00000258433	N.A.	8.64681062045123	0.00279152392233813
ENSG00000123360	PDE1B	8.62079889840109	0.00289943116959502
ENSG00000116983	HPCAL4	8.57801271098027	3.25919803975026e-16
ENSG00000261368	N.A.	8.5587887360365	0.00311162090153483
ENSG00000145075	CCDC39	8.55457529777017	0.00310032527730822
ENSG00000254946	N.A.	8.54214506923893	0.003334931587084
ENSG00000268089	GABRQ	8.54113481076188	0.00315387467707368
ENSG00000232273	FTH1P1	8.52951560147977	0.00329122922533408
ENSG00000179915	NRXN1	8.52335171640765	0.00342798378176786
ENSG00000108342	CSF3	8.52196463954854	0.00305812174502467
ENSG00000280283	N.A.	8.51160349295658	0.00339535747297685
ENSG00000185681	MORN5	8.48687814864671	0.00333437253560083
ENSG00000231233	CFAP58-AS1	8.48147309006073	0.0034157559931583
ENSG00000149654	CDH22	8.45235477769783	0.00352373264430923
ENSG00000184524	CEND1	8.44761167336971	2.61023268258183e-30
ENSG00000146216	TTBK1	8.44343226434237	0.00365007577178804
ENSG00000223865	HLA-DPB1	8.4385294534727	0.00358083685638634
ENSG00000246363	N.A.	8.41224026586029	0.00374689843119897
ENSG00000248144	ADH1C	8.38735980511474	0.00387908804159114
ENSG00000156076	WIF1	8.38583200643901	0.00405341235596149
ENSG00000175868	CALCB	8.37932964739891	0.00388055254388815
ENSG00000120729	MYOT	8.37682375980485	0.00382285880164201
ENSG00000196593	ANKRD20A19P	8.37513698872521	0.00397379161377129
ENSG00000243885	N.A.	8.37366418205166	0.00398776899251555
ENSG00000070019	GUCY2C	8.37219629713095	0.00401008350681079

ENSG00000101349	PAK7	8.37054455292227	0.00407264784594443
ENSG00000164604	GPR85	8.34583986300099	0.00411254942032978
ENSG00000104888	SLC17A7	8.33564166994184	8.17282956156986e-100
ENSG00000188848	BEND4	8.33381999292073	0.00413167932342154
ENSG00000185352	HS6ST3	8.32038531548456	0.00450145159930094
ENSG00000267614	N.A.	8.2997763364396	0.00440073367845964
ENSG00000080511	RDH8	8.29926985624396	0.00433710140701552
ENSG00000133101	CCNA1	8.22615397334955	4.94918417429284e-08
ENSG00000185669	SNAI3	8.2214446472015	0.00491469435893167
ENSG00000258435	N.A.	8.21790464423136	0.00493684411042778
ENSG00000170323	FABP4	8.20126456174432	0.00483223531073085
ENSG00000135333	EPHA7	8.186580348521	0.00513498102937086
ENSG00000133124	IRS4	8.17705064921699	0.00516134458935096
ENSG00000278740	N.A.	8.17288941358664	0.00532122532515953
ENSG00000167554	ZNF610	8.15858348490845	0.0054351552327854
ENSG00000197380	DACT3	8.15648011824687	7.97891754438747e-42
ENSG00000188505	NCCRP1	8.12402830527039	0.00548651542718018
ENSG00000142549	IGLON5	8.12365643731	0.00553269645245484
ENSG00000183166	CALN1	8.07612388194612	0.00591113375560591
ENSG00000060566	CREB3L3	8.05909451792136	1.00410947918161e-07
ENSG00000139549	DHH	8.04736930013025	0.00609178576386326
ENSG00000258082	N.A.	8.04416706717173	0.00615864263245033
ENSG00000110799	VWF	8.03906105155935	0.00626946237126128
ENSG00000229191	N.A.	8.03826310862133	0.00603277142572142
ENSG00000230086	VN1R96P	8.03476613235566	0.00614186844793595
ENSG00000170989	S1PR1	8.03226754520101	0.00603622780316935
ENSG00000221946	FXYD7	8.02839380144989	2.98247234114086e-14
ENSG00000261038	N.A.	8.02312056948318	0.00638665429584662
ENSG00000278811	LINC00624	8.01400606960492	1.22010541606264e-07
ENSG00000238076	MRPL48P1	7.99365847129066	0.00631845786237499
ENSG00000198453	ZNF568	7.98958883044623	0.00667529442785977
ENSG00000117322	CR2	7.97834670482816	0.00643133433978972
ENSG00000154553	PDLIM3	7.97295229207254	0.00676565154559788
ENSG00000270989	N.A.	7.96970868361878	0.00662347274673603
ENSG00000198865	CCDC152	7.93738332126061	0.00717414566340448
ENSG00000274447	N.A.	7.93332375881537	0.00722445529262759
ENSG00000279161	N.A.	7.91961872853676	0.00720889092027775
ENSG00000015592	STMN4	7.91902646035631	0.0074552072130183
ENSG00000204613	TRIM10	7.91652703967723	0.00713844393645501
ENSG00000257943	N.A.	7.91519122778175	0.00779447581801066
ENSG00000147257	GPC3	7.90053121030663	0.00760576010888157
ENSG00000204103	MAFB	7.89991914265425	0.00777784128803667
ENSG00000179593	ALOX15B	7.88419280366243	0.00694148582380076
ENSG00000147231	CXorf57	7.87442583637601	2.06796291461434e-07
ENSG00000259969	N.A.	7.84000302780039	0.00815210024841656
ENSG00000151490	PTPRO	7.8199264927979	0.00779087008021565
ENSG00000111885	MAN1A1	7.79551417898586	7.811512005826e-26
ENSG00000184515	BEX5	7.790757596583	0.00840882691220992
ENSG00000172201	ID4	7.77354781888626	0.00845279849463961

ENSG00000172061	LRRC15	7.76827339217276	0.00855262716329759
ENSG00000268756	N.A.	7.75065996812243	2.15462137129312e-19
ENSG00000080572	PIH1D3	7.75052469696489	0.00873538134171608
ENSG00000110076	NRXN2	7.73855163338194	2.28096364036072e-19
ENSG00000225778	PROSER2-AS1	7.72284010793216	0.00933715576514248
ENSG00000236154	N.A.	7.72082754055134	0.00901325394743861
ENSG00000259124	N.A.	7.71320568387224	0.00913396587371685
ENSG00000197721	CR1L	7.71282899322991	0.00920245830550519
ENSG00000204065	TCEAL5	7.71248639395179	0.00914215449404532
ENSG00000171487	NLRP5	7.70757608857891	0.00963646472275334
ENSG00000157103	SLC6A1	7.70338246214323	0.00921666144803971
ENSG00000075673	ATP12A	7.66260759370763	0.00962943360052939
ENSG00000255970	N.A.	7.65792846871473	0.00982982709220029
ENSG00000157303	SUSD3	7.65377540506797	0.00906840387759596
ENSG00000248596	N.A.	7.57596516857888	6.6054364784831e-127
ENSG00000154451	GBP5	7.46537048426138	2.4442582141604e-12
ENSG00000161905	ALOX15	7.38287801075339	1.39393863822299e-06
ENSG00000100867	DHRS2	7.37037616476833	0
ENSG00000064300	NGFR	7.34081642652633	5.77035254767691e-88
ENSG00000009694	TENM1	7.29621813821774	1.97691478021468e-48
ENSG00000255644	N.A.	7.2862895523266	8.53797730481525e-12
ENSG00000140465	CYP1A1	7.21026350118589	2.51366527165952e-32
ENSG00000261949	GFY	7.11186307452199	2.93158463040826e-11
ENSG00000128564	VGF	7.10101162648612	4.02715968371707e-91
ENSG00000075043	KCNQ2	7.08441346044034	3.83174676750889e-06
ENSG00000149294	NCAM1	7.0584582642994	3.90730020101443e-16
ENSG00000174460	ZCCHC12	7.03924754016118	1.27247457833865e-55
ENSG00000167654	ATCAY	7.03614880795602	6.52336891114643e-26
ENSG00000186115	CYP4F2	7.02843458005838	6.66985559743978e-21
ENSG00000212901	KRTAP3-1	7.02035628058479	1.12859979372528e-15
ENSG00000223722	N.A.	7.01265563025483	5.55262055812352e-06
ENSG00000095303	PTGS1	6.83639624102017	1.04057748846661e-05
ENSG00000231056	N.A.	6.76892778576102	1.27667117320962e-05
ENSG00000129990	SYT5	6.75709447599356	8.90900317311836e-15
ENSG00000141750	STAC2	6.70275212765584	3.44037519080162e-55
ENSG00000167434	CA4	6.67139490004844	1.59073637785034e-05
ENSG00000181856	SLC2A4	6.67059472019537	4.78116514406462e-32
ENSG00000279717	N.A.	6.61172446703628	1.99463006342375e-05
ENSG00000278965	N.A.	6.61028898940879	4.1489808589248e-14
ENSG00000006016	CRLF1	6.60887637490467	9.07729568985311e-23
ENSG00000076641	PAG1	6.58522340760926	4.55347878369028e-14
ENSG00000122877	EGR2	6.58214534854407	1.10703247862862e-09
ENSG00000080709	KCNN2	6.5743341273665	2.31356803811366e-05
ENSG00000187621	TCL6	6.48948452084711	1.19332414993798e-17
ENSG00000249174	N.A.	6.48893067581753	3.17284147831306e-05
ENSG00000281131	SCHLAP1	6.45318963106215	1.79574894977079e-17
ENSG00000280278	N.A.	6.44733138470141	3.74512263510698e-05
ENSG00000166501	PRKCB	6.43120487790149	1.48785786878375e-25
ENSG00000168824	N.A.	6.42813260267099	3.52297381749778e-05

ENSG00000173868	PHOSPHO1	6.39824414840161	3.43306787121071e-45
ENSG00000253880	N.A.	6.35221478692674	5.64004844677201e-13
ENSG00000282418	N.A.	6.32741368165297	5.65414034176397e-05
ENSG00000101680	LAMA1	6.31561639891991	2.57791719177611e-36
ENSG00000187672	ERC2	6.29590087151742	5.71725832254386e-05
ENSG00000125355	TMEM255A	6.28271090166299	1.21091932381056e-16
ENSG00000230183	CNOT6LP1	6.28141954409097	6.34300270606578e-05
ENSG00000263567	N.A.	6.27450019558685	8.26212185053857e-09
ENSG00000256615	N.A.	6.22072094719457	8.08967022218630e-24
ENSG00000255375	N.A.	6.2202554568235	8.66818775366958e-05
ENSG00000155657	TTN	6.19767755770146	1.32920943107972e-08
ENSG00000123243	ITIH5	6.15407757967909	1.14895529981551e-19
ENSG00000144868	TMEM108	6.14988584068455	1.63197888469369e-08
ENSG00000136274	NACAD	6.10472618269937	0.000106242724448432
ENSG00000007516	BAIAP3	6.09007366320053	1.72669309168201e-151
ENSG00000172137	CALB2	6.0858620920577	0.000114997911463124
ENSG00000139287	TPH2	6.07231905869263	4.74260176749714e-19
ENSG00000258789	N.A.	6.06241478393724	0.000119834475077724
ENSG00000104369	JPH1	6.05753647381833	0.000121796638753422
ENSG00000173208	ABCD2	6.05470743408498	2.92163517074627e-08
ENSG00000128510	CPA4	6.04274487950077	1.5174477311607e-11
ENSG00000225611	N.A.	6.04210841670183	1.07348342573286e-11
ENSG00000095932	SMIM24	6.03652738785802	3.77831970554604e-08
ENSG00000104967	NOVA2	6.0360430767498	1.60814865640215e-96
ENSG00000180720	CHRM4	6.01455873906579	3.33108504020115e-22
ENSG00000196361	ELAVL3	5.99616887536314	4.97807172866903e-08
ENSG00000184613	NELL2	5.99287605257324	6.338316665503e-15
ENSG00000006047	YBX2	5.99236381937841	1.12511917436882e-35
ENSG00000277586	NEFL	5.96178823281217	1.54659423034391e-35
ENSG00000267065	N.A.	5.95941544727706	0.000167897004611629
ENSG00000061337	LZTS1	5.92498637369683	0.000172502183733439
ENSG00000253898	LINC01419	5.91141611641623	7.89656454416271e-08
ENSG00000169918	OTUD7A	5.86795856012949	1.07427193991305e-07
ENSG00000205683	DPF3	5.86169701484546	4.11805229970626e-11
ENSG00000081181	ARG2	5.85806508196477	6.92224627867437e-83
ENSG00000130643	CALY	5.81198149688026	7.84724057700517e-11
ENSG00000135077	HAVCR2	5.80936477906896	0.000279558096524987
ENSG00000130876	SLC7A10	5.80261376556737	1.43531854135384e-07
ENSG00000234695	N.A.	5.79428862129909	2.31394230609376e-07
ENSG00000230666	CEACAM22P	5.77908560386546	7.94231749482082e-52
ENSG00000223638	RFPL4A	5.76017574519115	0.00030200109708372
ENSG00000154099	DNAAF1	5.75567686351204	2.81204147714526e-26
ENSG00000276509	N.A.	5.74137265700125	1.36266921252656e-07
ENSG00000126016	AMOT	5.73997357412597	9.66209459258559e-14
ENSG00000123405	NFE2	5.73655676463084	0.000323329910025275
ENSG00000187094	CCK	5.73347531827247	1.39106540762353e-16
ENSG00000169248	CXCL11	5.72941393542339	0.000355439694095549
ENSG00000092421	SEMA6A	5.72908045091156	1.29106868377889e-16
ENSG00000258545	RHOXF1-AS1	5.72090766579107	1.54982779857237e-10

ENSG00000119915	ELOVL3	5.7163161779745	7.54288183930967e-29
ENSG00000164744	SUN3	5.70473237062241	2.91944283586139e-07
ENSG00000087085	ACHE	5.68567952068806	5.8999715700219e-32
ENSG00000103740	ACSBG1	5.66032196080384	1.54979181702629e-58
ENSG00000155980	KIF5A	5.65376024439766	1.91993438674519e-22
ENSG00000167011	NAT16	5.65167606688666	0.000490174414979531
ENSG00000198963	RORB	5.6452335034135	0.000480890716679367
ENSG00000280639	N.A.	5.64511664992908	3.01836726555531e-16
ENSG00000120738	EGR1	5.63637061222917	8.85098706968189e-110
ENSG00000175877	WBSCR28	5.62478167416275	0.000467245703995446
ENSG00000137507	LRRC32	5.61960464203505	0.000472429564545452
ENSG00000160179	ABCG1	5.6074366722529	3.9221841134051e-28
ENSG00000155367	PPM1J	5.60426787870907	6.14236713098815e-19
ENSG00000107731	UNC5B	5.59512299275494	6.31288173961442e-43
ENSG00000109107	ALDOC	5.59287137637028	0
ENSG00000108387	SEPT4	5.59167078678826	4.32994017799287e-63
ENSG00000213939	N.A.	5.58499066040915	0.00054490341895641
ENSG00000238133	MLK7-AS1	5.57357296091529	0.000614182664912969
ENSG00000175175	PPM1E	5.569379390727	4.73632239389045e-39
ENSG00000227268	KLLN	5.56558589497868	8.88008150583864e-120
ENSG00000157782	CABP1	5.56088679087572	9.33535411320659e-16
ENSG00000230392	N.A.	5.55186851710522	2.43106848460731e-24
ENSG00000182575	NXPH3	5.53777890231902	6.3805589000278e-13
ENSG00000158553	POM121L2	5.5318358785664	0.000573583002513923
ENSG00000120820	GLT8D2	5.52154346764422	0.000598286843504535
ENSG00000143195	ILDR2	5.51645397142377	8.66513664122675e-07
ENSG00000184486	POU3F2	5.50607368805023	0.00066207024140772
ENSG00000233058	LINC00884	5.50486321384204	0.000766391819908592
ENSG00000010030	ETV7	5.49272230192282	1.82998417420109e-12
ENSG00000264451	N.A.	5.48952065955891	7.9568417622602e-07
ENSG00000119866	BCL11A	5.47950640603703	0.000765162132884502
ENSG00000119919	NKX2-3	5.47019563380843	0.000720311156972782
ENSG00000183914	DNAH2	5.46338592314533	1.42381585354715e-09
ENSG00000250423	KIAA1210	5.45691428971183	0.00066212934910897
ENSG00000143502	SUSD4	5.44381663319047	3.54356667850642e-15
ENSG00000256008	N.A.	5.43996219400369	0.00078247970619593
ENSG00000168280	KIF5C	5.42776046062933	5.36999088865153e-125
ENSG00000095713	CRTAC1	5.42493448023826	1.90939624925007e-09
ENSG00000162992	NEUROD1	5.41908114532027	0.000876249382812463
ENSG00000091592	NLRP1	5.39586532328692	1.35588880384626e-70
ENSG00000105696	TMEM59L	5.39120119170969	1.17778806819089e-68
ENSG00000004838	ZMYND10	5.36297630697763	2.42281699496599e-30
ENSG00000233098	CCDC144NL-AS1	5.35069679140567	1.97761159720518e-06
ENSG00000277200	N.A.	5.34437563985714	2.01808669156273e-06
ENSG00000135406	PRPH	5.34302673134071	1.58852496659794e-38
ENSG00000159307	SCUBE1	5.33382446983423	6.79065346793863e-25
ENSG00000183044	ABAT	5.32873293782285	1.0168039546321e-79
ENSG00000159167	STC1	5.32070404622569	5.04690741584589e-56
ENSG00000077616	NAALAD2	5.31731100934108	0.00114372617655575

ENSG00000085563	ABCB1	5.31112536846306	9.68994925040861e-12
ENSG00000129159	KCNC1	5.30004601806042	0.00105831862724407
ENSG00000175040	CHST2	5.29348420540108	4.02914300788596e-22
ENSG00000110675	ELMOD1	5.28806899635917	2.69263749477156e-06
ENSG00000124466	LYPD3	5.28555997221696	1.424800924227e-11
ENSG00000226306	NPY6R	5.27657275191847	5.37912461271012e-19
ENSG00000175445	LPL	5.27364460034553	0.00114086246794329
ENSG00000134873	CLDN10	5.26160476057278	1.02107963414767e-16
ENSG00000156453	PCDH1	5.25417254115002	7.76786215203569e-83
ENSG00000253130	N.A.	5.25143443903072	0.00156134408904236
ENSG00000171450	CDK5R2	5.24756821327661	1.83911607668445e-18
ENSG00000122735	DNAI1	5.24115704132768	2.00768817027499e-11
ENSG00000161681	SHANK1	5.23594270070527	0.00127491763426454
ENSG00000102879	CORO1A	5.22619040865394	7.0703541609114e-111
ENSG00000249379	N.A.	5.22135905141849	3.59553294999443e-06
ENSG00000179388	EGR3	5.22012851803231	5.03491145969537e-06
ENSG00000166793	YPEL4	5.21614262616314	3.60263178473087e-06
ENSG00000138100	TRIM54	5.20346702455217	0.00138292064494192
ENSG00000249790	N.A.	5.20000312749117	1.02251539129367e-101
ENSG00000236882	LINC01554	5.19712067299982	5.1548773065989e-19
ENSG00000074317	SNCB	5.19336755544775	5.70300487501071e-26
ENSG00000105642	KCNN1	5.19121179029728	1.01858546065616e-30
ENSG00000068831	RASGRP2	5.18161155629651	3.3336482389995e-16
ENSG00000130287	NCAN	5.15174529200426	0.00173434522364584
ENSG00000167614	TTYH1	5.11747646723619	0.00184307417751457
ENSG00000131831	RAI2	5.11538478901482	9.07895316367582e-11
ENSG00000130224	LRCH2	5.1078687296884	4.33276397831241e-34
ENSG00000163531	NFASC	5.10262322365455	2.45283691345055e-34
ENSG00000124440	HIF3A	5.09144638756617	0.00151128382354564
ENSG00000260103	N.A.	5.08924904406085	1.10766585154297e-42
ENSG00000152969	JAKMIP1	5.08858164114885	0.0020948741908563
ENSG00000119698	PPP4R4	5.0811789278557	2.70555689342876e-15
ENSG00000185272	RBM11	5.07550774681839	3.44804371108044e-08
ENSG00000132718	SYT11	5.07134190956283	2.01117814640929e-204
ENSG00000080573	COL5A3	5.05504752974877	8.25420898237436e-63
ENSG00000233791	LINC01136	5.05496339107731	0.00259545496274587
ENSG00000095370	SH2D3C	5.05364886691757	3.32237506212591e-15
ENSG00000187957	DNER	5.04844157016063	1.17031292742543e-116
ENSG00000111879	FAM184A	5.04765892439004	1.92589214239192e-10
ENSG00000197444	OGDHL	5.04307646886919	1.1947987228863e-23
ENSG00000010810	FYN	5.02138141231119	2.45917349336053e-56
ENSG00000165617	DACT1	5.01309180407772	0.00280488402728672
ENSG00000177108	ZDHHC22	5.00406661710129	0.00277323365476928
ENSG00000130812	ANGPTL6	4.99678913571516	0.00260005491500218
ENSG00000151117	TMEM86A	4.99425943912392	3.24748726281039e-30
ENSG00000087250	MT3	4.98905287861508	0.00269252753339154
ENSG00000261642	N.A.	4.97988778834899	1.49914193388061e-05
ENSG00000144285	SCN1A	4.97925893539095	8.21767939383165e-08
ENSG00000207331	RNU6-1263P	4.97819415930451	0.00275825504281619

ENSG00000233030	N.A.	4.9765726655793	1.71982281174245e-05
ENSG00000178821	TMEM52	4.97637891565123	3.91455116837854e-10
ENSG00000169583	CLIC3	4.96384020924878	0.00214734911152772
ENSG00000187902	SHISA7	4.96308397502495	0.00298505828558143
ENSG00000160862	AZGP1	4.94344017832744	1.71128796959832e-05
ENSG00000147676	MAL2	4.94336391312084	1.12310243421552e-07
ENSG00000184735	DDX53	4.93995655205226	0.00316866730567593
ENSG00000204252	HLA-DOA	4.92775051170957	0.00340138135280953
ENSG00000158473	CD1D	4.9219775464914	0.00346597055397246
ENSG00000135439	AGAP2	4.92046252518355	5.26342756478694e-42
ENSG00000102003	SYP	4.92027088370324	1.83707212889901e-197
ENSG00000265972	TXNIP	4.92019477073948	6.04026481782648e-202
ENSG00000071242	RPS6KA2	4.91781482378727	1.71969708640562e-27
ENSG00000169282	KCNAB1	4.90629049347392	1.91410409516062e-05
ENSG00000108309	RUNDC3A	4.90344457544148	1.64797031281018e-31
ENSG00000127252	HRASLS	4.89719871650482	0.00325695318206592
ENSG00000175264	CHST1	4.89709120288287	8.27333203872019e-10
ENSG00000172733	PURG	4.89286087468664	1.03813138884415e-09
ENSG00000176533	GNG7	4.89162307912066	1.61652023108097e-20
ENSG00000132677	RHBG	4.8904031694256	0.00316771676208935
ENSG00000156466	GDF6	4.87346471187413	0.00327584659705932
ENSG00000170379	TCAF2	4.86820748002809	1.48466815751594e-14
ENSG00000239467	N.A.	4.86731847207194	0.00351207691419201
ENSG00000115008	IL1A	4.8605077488423	0.00352230450208247
ENSG00000181085	MAPK15	4.84695510264531	0.00396613506275167
ENSG00000121871	SLITRK3	4.84537932587767	1.48779071968374e-13
ENSG00000165359	DDX26B	4.83972545330291	1.17052894531469e-15
ENSG00000213931	HBE1	4.83712914364991	0.00381275855361127
ENSG00000173727	N.A.	4.83224850000094	6.0853377549469e-193
ENSG00000257052	N.A.	4.83224744420807	0.0040044952776607
ENSG00000267424	N.A.	4.830596007794	0.0044155465645197
ENSG00000233922	N.A.	4.82160054471403	1.11543700520801e-19
ENSG00000049249	TNFRSF9	4.80960305353934	2.51183733327235e-11
ENSG00000177614	PGBD5	4.80601039303109	2.32701880712955e-09
ENSG00000130957	FBP2	4.8035361949832	4.07699619177301e-05
ENSG00000204610	TRIM15	4.80088432738583	3.45170078517061e-07
ENSG00000125740	FOSB	4.80012314272207	1.18599189637622e-33
ENSG00000178568	ERBB4	4.79902696787764	0.00394724527806218
ENSG00000280194	N.A.	4.78137579943922	0.00543480000232948
ENSG00000179542	SLITRK4	4.77894086019376	3.8576755955354e-05
ENSG00000158486	DNAH3	4.76122106730101	2.62833777263429e-88
ENSG00000158246	FAM46B	4.75859483455117	7.5437967787087e-38
ENSG00000165507	C10orf10	4.75812626390596	2.23788696558776e-11
ENSG00000158014	SLC30A2	4.7561284148428	0.00354272445155451
ENSG00000184357	HIST1H1B	4.75611249744015	0.00489209077243358
ENSG00000134042	MRO	4.75222235900358	3.41372127468018e-05
ENSG00000226359	ACTG1P24	4.74945022149114	0.00481743470399094
ENSG00000170044	ZPLD1	4.72852234551299	0.00583759514999236
ENSG00000100302	RASD2	4.72749297309258	7.19628325698608e-29

ENSG00000057704	TMCC3	4.72052688216149	3.7415589609139e-19
ENSG00000269729	N.A.	4.71354460980028	6.1526109045638e-05
ENSG00000085552	IGSF9	4.71253021212518	5.26504078969298e-11
ENSG00000267475	N.A.	4.7109367966346	1.00743619944703e-68
ENSG00000160471	COX6B2	4.70961029255722	2.18682818368106e-26
ENSG00000171815	PCDHB1	4.70680994515232	0.00527569085943736
ENSG00000204711	C9orf135	4.70554288604159	6.58206690683002e-05
ENSG00000250303	N.A.	4.69405035499937	2.74642532677529e-12
ENSG00000071575	TRIB2	4.69210837527029	5.9196301118864e-05
ENSG00000076716	GPC4	4.689816550735	8.75479196720551e-09
ENSG00000277453	N.A.	4.67977864665731	7.71053649096474e-05
ENSG00000205129	C4orf47	4.67317010394154	0.00590599029046494
ENSG00000139445	FOXN4	4.67255064041145	0.00575850773190362
ENSG00000178796	RIIAD1	4.66952410291925	7.85279212570946e-07
ENSG00000261963	N.A.	4.66359839930342	0.00571186414399909
ENSG00000104892	KLC3	4.658535502006	2.13179489553602e-56
ENSG00000143882	ATP6V1C2	4.65438800013614	7.98375625596653e-07
ENSG00000273199	N.A.	4.65211736486256	0.00549155431795885
ENSG00000205177	C11orf91	4.65079597248671	0.00616880248860577
ENSG00000143333	RGS16	4.65078885360702	6.54154256458534e-05
ENSG00000121743	GJA3	4.64945773306393	1.4983612272466e-48
ENSG00000101850	GPR143	4.64181955765282	6.72473794409973e-11
ENSG00000176641	RNF152	4.63748381079953	5.94397891817594e-05
ENSG00000175189	INHBC	4.6337707024923	0.00640975506142379
ENSG00000164287	CDC20B	4.63358010759019	0.00626946237126128
ENSG00000131771	PPP1R1B	4.63272455100982	0.005574694883479
ENSG00000119630	PGF	4.62558658855462	8.68769824091587e-29
ENSG00000139988	RDH12	4.62546079546678	2.50134145926697e-10
ENSG00000171817	ZNF540	4.62095573870206	8.87780214712405e-07
ENSG00000145087	STXBP5L	4.60411378343317	0.00756958849571164
ENSG00000089847	ANKRD24	4.59978420654543	1.46378401963411e-25
ENSG00000114251	WNT5A	4.59823693490851	0.00760071017079758
ENSG00000244468	N.A.	4.5970809743614	2.29890236120802e-52
ENSG00000246528	N.A.	4.59669029643286	0.00623641651057295
ENSG00000149633	KIAA1755	4.58746340766125	0.00774479607794864
ENSG00000184005	ST6GALNAC3	4.57667671927894	2.21997672888345e-08
ENSG00000163606	CD200R1	4.57566745130012	9.41896683799085e-05
ENSG00000231170	N.A.	4.57288702098482	8.75946940952914e-05
ENSG00000265778	N.A.	4.57214537289692	1.72884661897794e-08
ENSG00000115232	ITGA4	4.57079119120859	6.92414873982448e-14
ENSG00000164683	HEY1	4.56970958505767	1.38634124902978e-21
ENSG00000171812	COL8A2	4.55341304163985	0.00790562838306239
ENSG00000236393	N.A.	4.54785022484748	0.0083451378593867
ENSG00000237452	BHMG1	4.5301941895223	0.000125360322474111
ENSG00000167815	PRDX2	4.52595917641196	0.00913396587371685
ENSG00000186868	MAPT	4.52141632319227	4.73945773278749e-115
ENSG00000186310	NAP1L3	4.51953249148233	2.44991687903474e-32
ENSG00000130775	THEMIS2	4.5127899591041	7.67780604630479e-26
ENSG00000231131	LINC01468	4.50606907211494	0.000132011775764857

ENSG00000224728	N.A.	4.49934208561473	0.00919732251132799
ENSG00000132932	ATP8A2	4.49861116807041	0.000145246938755343
ENSG00000139971	C14orf37	4.49239125102815	2.19672571292778e-69
ENSG00000007314	SCN4A	4.48820785214088	4.13401551857692e-08
ENSG00000171724	VAT1L	4.48330698167165	0.00869099455049763
ENSG00000198576	ARC	4.47591996587309	1.10857791433571e-11
ENSG00000087589	CASS4	4.47416885259353	0.00920592212578861
ENSG00000198959	TGM2	4.47047926341374	0
ENSG00000188523	C9orf171	4.46272469852522	0.00949092477506373
ENSG00000196632	WNK3	4.44439421221424	2.52725956437549e-13
ENSG00000183570	PCBP3	4.43475637579424	1.20485508197667e-09
ENSG00000196972	SMIM10L2B	4.43285100841259	6.15558242115892e-18
ENSG00000149557	FEZ1	4.42577899527642	8.93545234225175e-18
ENSG00000076770	MBNL3	4.41697706413402	0.00021019387531748
ENSG00000259803	SLC22A31	4.41606410291188	6.17532221745155e-13
ENSG00000124575	HIST1H1D	4.41578303828166	1.15888425196913e-12
ENSG00000235621	LINC00494	4.41387583605669	4.4175882663327e-06
ENSG00000182397	DNM1P46	4.41114090303573	0.00971552291665119
ENSG00000165181	C9orf84	4.41101761509665	0.000236055407590041
ENSG00000233081	N.A.	4.40619468514672	0.000204015873029372
ENSG00000186648	LRRC16B	4.40572901897388	8.3215236447019e-23
ENSG00000164142	FAM160A1	4.40383582326169	4.47272315467054e-11
ENSG00000115009	CCL20	4.38923897995902	2.12882019327457e-06
ENSG00000123977	DAW1	4.38089919244004	0.000197272279411071
ENSG00000159409	CELF3	4.37603285885174	0.000286670047722296
ENSG00000204616	TRIM31	4.37137787653917	1.33672136903945e-67
ENSG00000267731	N.A.	4.36763772871211	0.000218854020417763
ENSG00000279064	N.A.	4.36701397401165	6.77198209472149e-06
ENSG00000116544	DLGAP3	4.36395252818557	3.92591585931218e-09
ENSG00000103723	AP3B2	4.35977179738209	0.000328955468307294
ENSG00000096088	PGC	4.34527810818539	0.00899666578570512
ENSG00000143341	HMCN1	4.34029098507598	5.67024601941594e-06
ENSG00000147206	NXF3	4.335599521759	0.0002580166440886
ENSG00000206113	N.A.	4.3175420842523	0.000287094922504816
ENSG00000276900	N.A.	4.3107113772689	1.99398927387915e-140
ENSG00000159399	HK2	4.30171711175418	3.65509504555561e-36
ENSG00000103196	CRISPLD2	4.30033963292642	2.11515670105418e-42
ENSG00000163888	CAMK2N2	4.29801730355694	4.25522438511177e-17
ENSG00000182568	SATB1	4.2974695117689	4.91286203847526e-23
ENSG00000121101	TEX14	4.29309922637969	5.31082465981975e-15
ENSG00000187608	ISG15	4.28402770253479	5.14729942460628e-78
ENSG00000233776	LINC01251	4.2688811497434	0.000345303698734343
ENSG00000169064	ZBBX	4.24775901530582	4.65680689428218e-07
ENSG00000231389	HLA-DPA1	4.24194224620024	0.000542527197952562
ENSG00000143590	EFNA3	4.24094159634724	8.72748518686954e-62
ENSG00000197872	FAM49A	4.24061676440691	0.000340232195765194
ENSG00000246016	LINC01513	4.2359276144854	0.000446314787888756
ENSG00000198729	PPP1R14C	4.22806492298134	4.90478241478533e-11
ENSG00000257732	N.A.	4.22296409754756	3.10174045833639e-27

ENSG00000236078	LINC01447	4.21424478037081	1.15089407162897e-38
ENSG00000011201	ANOS1	4.20888621873386	1.81899923891182e-14
ENSG00000124216	SNAI1	4.19870870471748	6.20136282320435e-38
ENSG00000163536	SERPINI1	4.1954646713432	2.11599330745183e-81
ENSG00000138028	CGREF1	4.19161547716689	1.40580793638285e-194
ENSG00000198910	L1CAM	4.19129857958171	4.62614105674314e-132
ENSG00000181908	N.A.	4.1771783053433	0.000547107314393056
ENSG00000229308	N.A.	4.17021408926998	2.12455877522125e-05
ENSG00000140450	ARRDC4	4.16769507380943	8.7308936200363e-69
ENSG00000236514	N.A.	4.16618410253096	0.000664039042287004
ENSG00000107859	PITX3	4.16311565599521	6.84485448312376e-10
ENSG00000224786	CETN4P	4.14891985699453	0.000716723047934715
ENSG00000131398	KCNC3	4.14633232413893	3.58008395621466e-78
ENSG00000175985	PLEKHD1	4.14504133214669	1.85074005542936e-05
ENSG00000140323	DISP2	4.14451479682881	7.15575375632029e-44
ENSG00000103522	IL21R	4.12883919758977	7.99277967625641e-24
ENSG00000169439	SDC2	4.12263775016746	9.9021908161791e-07
ENSG00000105409	ATP1A3	4.11074091886391	2.79820615720617e-248
ENSG00000128346	C22orf23	4.10686211234829	8.3726934029232e-11
ENSG00000125409	TEKT3	4.0969522447554	4.26804268387568e-05
ENSG00000164841	TMEM74	4.09256958078953	3.2546475679544e-08
ENSG00000223820	CFL1P1	4.08628516751545	5.6386482447301e-16
ENSG00000087258	GNAO1	4.07593502277301	8.53583340990265e-11
ENSG00000229807	XIST	4.06586287556816	0.000947876452029584
ENSG00000260132	N.A.	4.05991778605529	0.00118082027669296
ENSG00000111344	RASAL1	4.05898039162498	1.78354864275148e-30
ENSG00000121207	LRAT	4.03143552380556	1.36760555657228e-10
ENSG00000276846	N.A.	4.03111617298916	1.74874433871837e-06
ENSG00000141485	SLC13A5	4.02942364021141	0.0009011384966956
ENSG00000260101	N.A.	4.02528194346899	1.53937514283438e-10
ENSG00000100290	BIK	4.02430091788412	1.7679882733172e-26
ENSG00000145491	ROPN1L	4.01239878304864	3.17331177706218e-34
ENSG00000274292	N.A.	4.01059020536017	1.73027319411925e-17
ENSG00000257954	N.A.	4.00963841533891	0.00106112081571074
ENSG00000131015	ULBP2	4.0082747554236	1.7891303843978e-109
ENSG00000182175	RGMA	4.00782799644333	4.00340078557546e-05
ENSG00000120324	PCDHB10	4.00027030996506	1.43382438034983e-07
ENSG00000078725	BRINP1	3.99913111788616	5.39879100659619e-09
ENSG00000036672	USP2	3.99709988883297	1.368997090176e-41
ENSG00000143028	SYPL2	3.99558817895784	1.29740130510883e-12
ENSG00000236643	N.A.	3.99366168893211	0.00132438237005069
ENSG00000230583	GTF2IRD1P1	3.98873908802402	6.19143982693215e-05
ENSG00000185664	PMEL	3.9738837686481	1.82657688449264e-136
ENSG00000264569	N.A.	3.9687805289444	0.00116443520343428
ENSG00000168481	LGI3	3.96657219680652	3.37806842176032e-35
ENSG00000187686	KRT18P59	3.96311020150925	1.76719109148407e-07
ENSG00000171033	PKIA	3.96077900483138	9.13003724238415e-05
ENSG00000115255	REEP6	3.95052819564599	4.97515946205212e-226
ENSG00000144331	ZNF385B	3.95036141935633	0.00143471590168617

ENSG00000205835	GMNC	3.94826417631109	0.00155223058659639
ENSG00000198003	CCDC151	3.94725643971543	1.47759920421654e-08
ENSG00000183833	MAATS1	3.94642387199516	1.3291876413574e-13
ENSG00000123342	MMP19	3.94482044935548	5.83405491716221e-05
ENSG00000105668	UPK1A	3.94069984106243	1.14097853643508e-13
ENSG00000123095	BHLHE41	3.93602567515804	3.17720958349451e-11
ENSG00000197093	GAL3ST4	3.9347924664004	1.94139842185394e-12
ENSG00000164675	IQUB	3.93122692942158	4.11458397499556e-16
ENSG00000257839	N.A.	3.91664739129249	2.04839335315319e-10
ENSG00000215045	GRID2IP	3.91481331458258	0.00139780714983773
ENSG00000239620	N.A.	3.90683096195195	0.000577598334242644
ENSG00000183128	CALHM3	3.90652116297697	2.09338815097066e-08
ENSG00000135114	OASL	3.90466945253137	7.66996129975709e-45
ENSG00000143320	CRABP2	3.89839909488246	8.51864063168818e-33
ENSG00000095637	SORBS1	3.89686206457929	2.701519307793e-117
ENSG00000156140	ADAMTS3	3.89066120901498	0.00214359166801254
ENSG00000280401	N.A.	3.88717214116641	1.97508206620076e-10
ENSG00000173237	C11orf86	3.88253413421658	1.07038750701489e-58
ENSG00000226510	UPK1A-AS1	3.8645067233872	1.04054812788136e-05
ENSG00000158164	TMSB15A	3.86371198316149	2.12516150578509e-16
ENSG00000113073	SLC4A9	3.86363573104246	0.00210744787582777
ENSG00000253882	N.A.	3.85909130019757	7.97077534429617e-14
ENSG00000186510	CLCNKA	3.85482847135812	0.00198720928415289
ENSG00000205293	LINC01602	3.84830877566288	1.25716582551359e-05
ENSG00000213023	SYT3	3.84179270724824	5.45708615672186e-08
ENSG00000081479	LRP2	3.8327793264779	1.05085500200567e-11
ENSG00000164674	SYTL3	3.83232707977645	2.15014661548799e-20
ENSG00000182489	XKRX	3.8290097008262	1.40172425429008e-07
ENSG00000152495	CAMK4	3.82399726876084	0.0022374929957837
ENSG00000036530	CYP46A1	3.8233127987309	2.34943944446161e-25
ENSG00000231652	N.A.	3.82040543320011	8.51205747946726e-25
ENSG00000239462	N.A.	3.81931587504428	1.77088635060049e-09
ENSG00000258590	NBEAP1	3.81871762845974	0.000186548269084409
ENSG00000231256	C17orf105	3.81784381961874	8.78842465692395e-07
ENSG00000180596	HIST1H2BC	3.81147074367779	2.087052230574e-44
ENSG00000153902	LGI4	3.80268011739002	4.98397149938902e-12
ENSG00000163995	ABLIM2	3.80094661841207	8.18554840327542e-12
ENSG00000273102	N.A.	3.7977847247853	0.00338958622986313
ENSG00000164651	SP8	3.79592213152264	0.000189930017431581
ENSG00000183508	FAM46C	3.79206541333606	5.52497670920002e-08
ENSG00000078081	LAMP3	3.7905779297264	1.16970348300652e-24
ENSG00000169252	ADRB2	3.78961509730813	6.3133949739711e-08
ENSG00000255150	EID3	3.78906551606013	0
ENSG00000259438	N.A.	3.78338053701391	1.4452780767724e-06
ENSG00000274528	N.A.	3.78315097247691	3.01377546660251e-20
ENSG00000169071	ROR2	3.78150498056105	1.2059184452996e-05
ENSG00000137877	SPTBN5	3.77942799877465	1.68567204603931e-25
ENSG00000139269	INHBE	3.77230711205277	2.83383090800383e-07
ENSG00000157653	C9orf43	3.76277817693274	5.95541320406351e-20

ENSG00000130176	CNN1	3.75416106932095	0.000286795296590936
ENSG00000229891	LINC01315	3.74949790212893	1.10489157235594e-08
ENSG00000143469	SYT14	3.74843812306089	0.00279359793445774
ENSG00000111834	RSPH4A	3.74599100774318	2.8209110463834e-28
ENSG00000126259	KIRREL2	3.74032411640581	4.61750409436574e-54
ENSG00000228288	PCAT6	3.73877874257871	2.37163456779499e-16
ENSG00000268628	N.A.	3.73695297122663	0.00297812171038659
ENSG00000135447	PPP1R1A	3.73689913669089	5.65648649922812e-08
ENSG00000113389	NPR3	3.73674420872217	3.89975365989338e-10
ENSG00000008311	AASS	3.73606583668285	5.03863927136164e-35
ENSG00000244791	N.A.	3.73483494709959	0.000269540369903242
ENSG00000158258	CLSTN2	3.73470062602689	1.08706275708372e-08
ENSG00000181322	NME9	3.73066172725921	0.00403088336835594
ENSG00000231738	TSPAN19	3.71710479196166	1.25422273660151e-09
ENSG00000157423	HYDIN	3.71350602852475	1.27056108326847e-07
ENSG00000237810	N.A.	3.71325958135741	0.00616764166665831
ENSG00000164659	KIAA1324L	3.71250889465469	1.37525156717858e-17
ENSG00000172349	IL16	3.706465232614	0.00298113533859068
ENSG00000178997	EXD1	3.70437903627039	8.96794033140129e-09
ENSG00000075290	WNT8B	3.70317753952619	0.00320447505302746
ENSG00000214456	PLIN5	3.70198417192814	1.73206033771729e-10
ENSG00000055118	KCNH2	3.69522062767604	7.83769763947771e-86
ENSG00000120328	PCDHB12	3.69411395702121	0.00483232199136829
ENSG00000182759	MAFA	3.68964499610675	3.68767959868768e-05
ENSG00000228451	SDAD1P1	3.67857693803913	8.37567528192283e-65
ENSG00000273137	N.A.	3.67566876023608	0.00401293766112862
ENSG00000145451	GLRA3	3.67431548057098	0.00378414410837876
ENSG00000124092	CTCFL	3.67128537145865	0.00484222775958042
ENSG00000117245	KIF17	3.66764663712044	2.95532393258671e-20
ENSG00000142552	RCN3	3.66526463703777	2.03837194634064e-53
ENSG00000163623	NKX6-1	3.66520495572983	1.65095520155895e-10
ENSG00000224846	N.A.	3.66189769405483	0.000371032499030006
ENSG00000100985	MMP9	3.65917421029426	3.6182328246603e-05
ENSG00000118307	CASC1	3.65287371269978	1.08816917506437e-21
ENSG00000215014	N.A.	3.64861518488743	0.000335062961184384
ENSG00000125744	RTN2	3.64838863946001	3.78704623350599e-254
ENSG00000182379	NXPH4	3.64632085187283	3.71251249635833e-96
ENSG00000251127	N.A.	3.64596623409581	8.05565937424717e-05
ENSG00000180279	N.A.	3.64236124517401	0.00516134458935096
ENSG00000146005	PSD2	3.63931804840835	0.00369057253602198
ENSG00000176723	ZNF843	3.63770793760483	1.6955913908188e-09
ENSG00000153233	PTPRR	3.63460989456201	9.78829302318009e-15
ENSG00000272163	N.A.	3.63030780700745	0.00393067002933312
ENSG00000175984	DENND2C	3.62839405864109	1.4408572886671e-31
ENSG00000006638	TBXA2R	3.62068073768037	1.86947607072298e-15
ENSG00000102409	BEX4	3.6156285431246	1.23185407369524e-13
ENSG00000224888	N.A.	3.61274868723351	0.00686478731624705
ENSG00000007312	CD79B	3.61145827686114	0.00689509405188862
ENSG00000249992	TMEM158	3.60446840251498	8.50659861509171e-18

ENSG00000184908	CLCNKB	3.60440719733993	0.00477912497200916
ENSG00000129910	CDH15	3.60330183987284	4.42627188714412e-06
ENSG00000168993	CPLX1	3.60093501148021	2.55419999819488e-16
ENSG00000172508	CARNS1	3.59959009148845	0.0077352176897644
ENSG00000196337	CGB7	3.59709868570202	0.00455047556532346
ENSG00000166920	C15orf48	3.59679294194846	5.67021547218141e-25
ENSG00000050438	SLC4A8	3.59580276175351	6.08123942223584e-238
ENSG00000270021	N.A.	3.59413397697293	3.78429630879145e-06
ENSG00000167613	LAIR1	3.59342997054653	0.000663220611722184
ENSG00000178573	MAF	3.59083163294517	6.99013557604013e-05
ENSG00000133687	TMTC1	3.59029957774901	6.45765928772704e-08
ENSG00000171444	MCC	3.58866179784693	4.28274593234029e-13
ENSG00000234076	TPRG1-AS1	3.58757383340417	1.35913603046783e-05
ENSG00000182459	TEX19	3.5839200676059	7.27040569932579e-26
ENSG00000129451	KLK10	3.57753243918763	4.31154200884593e-06
ENSG00000272829	N.A.	3.577477614426	0.000541295807599253
ENSG00000100604	CHGA	3.5768228729934	2.39397665516026e-16
ENSG00000105605	CACNG7	3.57548914170718	4.87097525622206e-155
ENSG00000179292	TMEM151A	3.57440737777492	1.75858544758316e-37
ENSG00000007237	GAS7	3.56736670670862	4.77723288181642e-05
ENSG00000263155	MYZAP	3.56462624408033	2.22620065437064e-16
ENSG00000174607	UGT8	3.56258318126044	6.53131011259583e-16
ENSG00000103710	RASL12	3.56043062182484	0.00638568543916237
ENSG00000143603	KCNN3	3.55967483407526	6.22563793366434e-12
ENSG00000104081	BMF	3.55481110223455	4.88137014969281e-106
ENSG00000135678	CPM	3.55385948893117	4.18657359688292e-212
ENSG00000237595	N.A.	3.54427048740797	8.76496273002343e-18
ENSG00000272360	N.A.	3.5420808240764	4.9443786909272e-08
ENSG00000171368	TPPP	3.54118428726125	2.49614715076615e-168
ENSG00000282851	N.A.	3.54078181419117	0.0065997805278721
ENSG00000127863	TNFRSF19	3.53581333172657	8.33295704028545e-10
ENSG00000105650	PDE4C	3.53432437129195	9.04628473211972e-10
ENSG00000065320	NTN1	3.52776457505382	5.09738174460359e-30
ENSG00000105649	RAB3A	3.52692253962163	4.68930634591638e-27
ENSG00000095587	TLL2	3.52616763269976	1.04795050167825e-08
ENSG00000197191	CYSRT1	3.51518078603505	2.63215920354178e-11
ENSG00000249661	TNRC18P1	3.51305053349343	0.00815147670223909
ENSG00000197457	STMN3	3.51209916236121	2.87037000776463e-57
ENSG00000247157	LINC01252	3.51015334630735	3.41275459017836e-19
ENSG00000077092	RARB	3.50994412387573	1.88646853007957e-35
ENSG00000213401	MAGEA12	3.50492880785608	6.503200746336e-05
ENSG00000108370	RGS9	3.50318120202835	7.11910606582691e-21
ENSG00000144668	ITGA9	3.50214020123027	0.00892401559139023
ENSG00000237330	RNF223	3.50135638259423	0.000833563754866134
ENSG00000110324	IL10RA	3.49977670294939	4.86115287165821e-07
ENSG00000092068	SLC7A8	3.49939933222101	8.75956125174333e-21
ENSG00000136531	SCN2A	3.49333092023958	0.000766443507685374
ENSG00000106066	CPVL	3.49311653849121	0.000707225031797946
ENSG00000258240	N.A.	3.49158289901798	1.01249086431645e-06

ENSG00000084110	HAL	3.48835179896135	0.000250644245808618
ENSG00000185518	SV2B	3.48176855449387	0.00619762588061119
ENSG00000183935	HTR7P1	3.4817678846383	2.40407402617687e-152
ENSG00000153237	CCDC148	3.47166599889583	9.1107026147659e-22
ENSG00000105246	EBI3	3.46915127473467	0.00640403512352357
ENSG00000187837	HIST1H1C	3.46195822416197	1.30438384262824e-295
ENSG00000133134	BEX2	3.44389556804193	3.97765443473582e-32
ENSG00000028137	TNFRSF1B	3.44364487179551	1.83360269691898e-05
ENSG00000169894	MUC3A	3.44097633378731	2.65809990193624e-30
ENSG00000011347	SYT7	3.4405626283943	1.06087787742333e-58
ENSG00000066468	FGFR2	3.44010789194941	1.68492087193593e-18
ENSG00000247675	LRP4-AS1	3.43793857535883	8.79411662825538e-10
ENSG00000104044	OCA2	3.43197753821416	9.61016343840617e-14
ENSG00000159648	TEPP	3.42842372642451	0.00943086840650325
ENSG00000280407	N.A.	3.42627931231594	0.00838867677131067
ENSG00000088726	TMEM40	3.42377170785635	0.00216983570007715
ENSG00000255507	N.A.	3.41864482359963	2.98209317569775e-05
ENSG00000170345	FOS	3.41802059696989	2.54338629775226e-93
ENSG00000276166	N.A.	3.41731301729662	0.00829311312769856
ENSG00000206532	N.A.	3.41583256549405	0.000310406069316482
ENSG00000119138	KLF9	3.41429088982821	1.71038199080605e-59
ENSG00000139537	CCDC65	3.41283322755171	1.06692479582172e-17
ENSG00000178053	MLF1	3.40760474522391	1.78780219778621e-289
ENSG00000135604	STX11	3.40648480341156	1.30293194109195e-12
ENSG00000245468	N.A.	3.40643050064468	1.71251237958622e-05
ENSG00000255847	N.A.	3.40403347432034	0.000877120571732828
ENSG00000255346	NOX5	3.40247795665593	0.000279117552905107
ENSG00000170153	RNF150	3.40180282366127	2.09269272283091e-12
ENSG00000139364	TMEM132B	3.39078174558601	0.000268019349813086
ENSG00000165731	RET	3.38712250266449	5.92498263973619e-14
ENSG00000168298	HIST1H1E	3.38694977690948	1.56104848226978e-09
ENSG00000179168	GGN	3.38336574423151	0.000298352994094315
ENSG00000255874	LINC00346	3.38324467530544	3.75619696951487e-08
ENSG00000204386	NEU1	3.37931883439203	0
ENSG00000118160	SLC8A2	3.37889183816489	2.72600045546982e-32
ENSG00000183682	BMP8A	3.37584314970015	3.42710349171515e-06
ENSG00000205744	DENND1C	3.37210875261068	3.25491514221727e-18
ENSG00000263551	N.A.	3.3704891882909	0.000219387739824787
ENSG00000101098	RIMS4	3.36673392171358	6.8247467715849e-49
ENSG00000138623	SEMA7A	3.36576451068447	5.92509596399488e-12
ENSG00000105516	DBP	3.36004880542658	4.35248320769315e-28
ENSG00000135547	HEY2	3.35983002201348	1.61353848000294e-07
ENSG00000149599	DUSP15	3.35977702696875	6.06876502326182e-07
ENSG00000139714	MORN3	3.35729454796313	5.37243212801769e-10
ENSG00000135424	ITGA7	3.35585017170491	8.25604509288931e-141
ENSG00000130294	KIF1A	3.3534046127583	1.59575899748941e-75
ENSG00000105376	ICAM5	3.35204139204651	1.2129046203902e-09
ENSG00000196890	HIST3H2BB	3.35103656620959	2.94878902629579e-11
ENSG00000178878	APOLD1	3.35098667162767	3.98565398850085e-37

ENSG00000142910	TINAGL1	3.34893996398579	2.21906410231427e-07
ENSG00000149564	ESAM	3.3475476772647	2.0442014023972e-49
ENSG00000113763	UNC5A	3.34561423882906	3.36912760035738e-08
ENSG00000129538	RNASE1	3.34495694875236	0.00201181267691105
ENSG00000279086	N.A.	3.34013759450862	0.00994589396088697
ENSG00000261441	N.A.	3.33590668355398	0.000217855521083845
ENSG00000133665	DYDC2	3.33340030367394	0.00183360792877781
ENSG00000204044	N.A.	3.33103762859011	4.85421843183009e-08
ENSG00000080644	CHRNA3	3.33043521781799	9.22795163694908e-12
ENSG00000184156	KCNQ3	3.32700923072291	0.00186649665231821
ENSG00000115844	DLX2	3.32547925121992	1.86952254862945e-06
ENSG00000257137	C12orf80	3.32519981840548	0.00131331823801079
ENSG00000186897	C1QL4	3.32171498416838	1.90826556187977e-50
ENSG00000166963	MAP1A	3.31890870979156	3.25328738362717e-88
ENSG00000156042	CFAP70	3.31781772897077	1.08867480555369e-23
ENSG00000274827	LINC01297	3.31435011373797	4.16161100652877e-05
ENSG00000113070	HBEGF	3.30879823631533	8.29482728111164e-36
ENSG00000188672	RHCE	3.29872990210176	0.00281229903309149
ENSG00000111837	MAK	3.29268219438255	5.7552832775669e-14
ENSG00000142920	AZIN2	3.28696630198953	2.94864167233001e-22
ENSG00000177374	HIC1	3.28258686880444	8.80088631944452e-05
ENSG00000121653	MAPK8IP1	3.28172255650825	1.37018173273344e-177
ENSG00000279602	N.A.	3.27973668065879	8.10101494000368e-13
ENSG00000279879	N.A.	3.27870444094056	1.43181676248236e-05
ENSG00000266904	N.A.	3.27800133205765	0.00207655969742717
ENSG00000218336	TENM3	3.2776210515658	7.33129314475328e-06
ENSG00000135144	DTX1	3.27713554761369	8.62745939111022e-10
ENSG00000206077	ZDHHC11B	3.27415651602495	1.17786479204761e-30
ENSG00000108771	DHX58	3.27320626276196	1.38607628521881e-09
ENSG00000006747	SCIN	3.27001531434906	2.04785839044362e-42
ENSG00000119411	BSPRY	3.26392539929814	1.67890438074477e-06
ENSG00000179399	GPC5	3.26235989920666	1.26138558812604e-05
ENSG00000162873	KLHDC8A	3.26188627142982	8.05384068074266e-10
ENSG00000197959	DNM3	3.25274212775097	1.23008990199003e-133
ENSG00000010310	GIPR	3.24837798560266	2.74213382128227e-41
ENSG00000177453	NIM1K	3.239827451912	7.72020097263863e-11
ENSG00000234996	N.A.	3.23488476510488	4.23051865614403e-10
ENSG00000164099	PRSS12	3.23364682821025	1.46450994848799e-30
ENSG00000166670	MMP10	3.23348444720988	0.00109565009236542
ENSG00000260495	N.A.	3.23322853994576	0.00237664991885558
ENSG00000105251	SHD	3.22786716068086	1.61954832395285e-06
ENSG00000272031	ANKRD34A	3.22202622322657	2.78152695919805e-16
ENSG00000089558	KCNH4	3.22082299620417	1.56814353672747e-07
ENSG00000136352	NKX2-1	3.22032888052183	0.00338102594489582
ENSG00000167646	DNAAF3	3.22016156451753	1.06712621816218e-99
ENSG00000185745	IFIT1	3.21946743848559	2.65241216177281e-128
ENSG00000206567	N.A.	3.21741217280236	2.32348308241009e-14
ENSG00000123338	NCKAP1L	3.21407402379065	2.47241843623164e-07
ENSG00000108947	EFNB3	3.21252107032269	1.11898450782595e-39

ENSG00000105825	TFPI2	3.20968865577226	0
ENSG00000203814	HIST2H2BF	3.20178606933263	1.03604601948694e-32
ENSG00000183691	NOG	3.20136906990626	0.0043763244093133
ENSG00000273274	ZBTB8B	3.20029890450012	2.57279670481188e-08
ENSG00000263826	N.A.	3.19517403070922	0.000485431637376044
ENSG00000256116	N.A.	3.18991468391988	1.64891387918761e-07
ENSG00000059915	PSD	3.18961566654342	1.81148979683726e-08
ENSG00000134962	KLB	3.18822959617335	1.08368154810432e-91
ENSG00000173083	HPSE	3.1874464197845	2.19055493591699e-71
ENSG00000244558	KCNK15-AS1	3.18412102137681	3.16018870973017e-05
ENSG00000126709	IFI6	3.18372270875709	2.27202505829982e-97
ENSG00000261033	N.A.	3.17979854171809	4.78518685013596e-08
ENSG00000156103	MMP16	3.17683889152538	5.86500956358868e-06
ENSG00000250722	SEPP1	3.1764623248185	6.81383034891031e-52
ENSG00000172771	EFCAB12	3.17644385633722	7.61527142532354e-27
ENSG00000126264	HCST	3.17459783167376	0.00358102167089672
ENSG00000187867	PALM3	3.17387736778347	2.99575710325375e-21
ENSG00000260641	N.A.	3.17228653341793	0.000154909886404639
ENSG00000257512	N.A.	3.16539690342377	1.96617807117165e-19
ENSG00000170498	KISS1	3.16451640551631	0.000859971516789737
ENSG00000230526	N.A.	3.16216744828833	3.47375590512651e-06
ENSG00000131094	C1QL1	3.16162380563693	1.59460030175618e-203
ENSG00000204758	N.A.	3.16025955905125	1.08525706184268e-34
ENSG00000203441	LINC00449	3.15816403210401	0.000142693456575319
ENSG00000236673	N.A.	3.15466732123688	9.84213862058794e-06
ENSG00000271122	N.A.	3.15163378230018	8.01391623583623e-199
ENSG00000225489	N.A.	3.15035306479	0.000792747655659067
ENSG00000230679	ENO1-AS1	3.14995186213283	0.000948124385763897
ENSG00000110876	SELPLG	3.14841762940814	1.03628395388996e-12
ENSG00000138166	DUSP5	3.1481155564445	3.06252313135525e-255
ENSG00000186952	TMEM232	3.14438381705423	1.13405734816564e-06
ENSG00000113212	PCDHB7	3.1442962107635	3.99050410629133e-05
ENSG00000171236	LRG1	3.14422889004077	7.88117560766504e-19
ENSG00000176746	MAGEB6	3.14328798248389	1.09030323425188e-09
ENSG00000188687	SLC4A5	3.14326776971579	2.24527396662147e-14
ENSG00000104327	CALB1	3.13331659129771	1.65340886167558e-12
ENSG00000261040	WFDC21P	3.13323670912852	1.26920784283652e-13
ENSG00000266304	N.A.	3.13152306720855	0.00699197639825345
ENSG00000165091	TMC1	3.13060987261301	0.00600771493605663
ENSG00000184497	TMEM255B	3.12929249524946	0.00330551650628911
ENSG00000273143	N.A.	3.12825018306245	0.00111038056165621
ENSG00000146592	CREB5	3.12569571227799	1.53880985537082e-12
ENSG00000204052	LRRC73	3.12489439381295	2.05681401026784e-15
ENSG00000136059	VILL	3.12040774790081	0.000167300042051149
ENSG00000187554	TLR5	3.11792344922185	0.00056781094813821
ENSG00000111424	VDR	3.11791047568275	3.422686284534e-69
ENSG00000204219	TCEA3	3.11679011298546	4.13605228876782e-07
ENSG00000223658	C1GALT1C1L	3.11365144489616	4.49799570397146e-25
ENSG00000154118	JPH3	3.1116318507263	3.16421431248193e-11

ENSG00000106772	PRUNE2	3.10455650685631	2.63766696342592e-12
ENSG00000012124	CD22	3.10375309462008	1.78595394212002e-13
ENSG00000197181	PIWIL2	3.10109318564254	1.58831561785048e-11
ENSG00000258733	N.A.	3.10059275231406	0.000912619564135286
ENSG00000161642	ZNF385A	3.09693455430544	2.62647388609183e-284
ENSG00000204950	LRRC10B	3.09272489687913	0.0074570935540944
ENSG00000137878	GCOM1	3.08980288231257	3.94940604844025e-05
ENSG00000105613	MAST1	3.08674842921944	9.68909222725661e-25
ENSG00000090534	THPO	3.08584943118828	0.00498668232572801
ENSG00000277399	GPR179	3.08466811867981	0.00869145523231655
ENSG00000169085	C8orf46	3.08321101176615	1.69276301608995e-06
ENSG00000176884	GRIN1	3.08145623126851	8.7210348040443e-08
ENSG00000170965	PLAC1	3.07690875694854	1.2849511844264e-18
ENSG00000253256	N.A.	3.07393176802589	0.00558916669610891
ENSG00000198626	RYR2	3.07391271684911	0.00120692685038971
ENSG00000168843	FSTL5	3.07319080500478	2.62093254215901e-06
ENSG00000259251	N.A.	3.07122807506713	9.63798851013897e-05
ENSG00000281538	N.A.	3.0579628460908	7.0645793153952e-07
ENSG00000251095	N.A.	3.05720558006951	1.17733898377467e-26
ENSG00000100234	TIMP3	3.05600745500183	1.82124818049999e-05
ENSG00000155265	GOLGA7B	3.05371283580421	1.67238204468361e-33
ENSG00000065609	SNAP91	3.05221562883234	1.08243686675129e-09
ENSG00000130751	NPAS1	3.05080045091937	7.30863230560394e-52
ENSG00000160460	SPTBN4	3.0500268849414	2.64541646969747e-40
ENSG00000261079	N.A.	3.04945758559369	0.00576073855147159
ENSG00000108830	RND2	3.04878083504086	1.91739584226769e-11
ENSG00000270964	N.A.	3.0473859172662	4.06820082279157e-09
ENSG00000233077	LINC01271	3.04582654101136	4.39658507643665e-07
ENSG00000164076	CAMKV	3.04573877374052	6.61169763448607e-06
ENSG00000008323	PLEKHG6	3.04228495962946	5.53031369756746e-09
ENSG00000133808	MICALCL	3.04211636431843	0.000831456705965015
ENSG00000158406	HIST1H4H	3.04183063067082	2.50928137549098e-63
ENSG00000130303	BST2	3.04100583951478	8.52605610062867e-07
ENSG00000277235	N.A.	3.03999146683677	0.000249195313226765
ENSG00000187210	GCNT1	3.03286004478974	4.44868440898614e-29
ENSG00000230359	TPI1P2	3.03259515365031	3.23590037619484e-27
ENSG00000059728	MXD1	3.03207986957211	2.5727690026337e-131
ENSG00000235781	N.A.	3.0320090149498	0.00175612121118917
ENSG00000155961	RAB39B	3.03129178077995	1.56517866692858e-48
ENSG00000157152	SYN2	3.030177938217	0.000364405953706138
ENSG00000273061	CDC37L1-AS1	3.02704807819422	8.27674409471794e-05
ENSG00000109705	NKX3-2	3.02347167720673	0.000351532473116393
ENSG00000062282	DGAT2	3.0207790015674	6.25370136291429e-16
ENSG00000147168	IL2RG	3.02032033638616	0.00940375808262123
ENSG00000101605	MYOM1	3.01153420083065	3.75422037787282e-11
ENSG00000158270	COLEC12	3.00986667731516	0.00708009774464899
ENSG00000206828	N.A.	3.00944874414087	0.00145290186158714
ENSG00000217801	N.A.	3.0082483379273	1.43530038679753e-44
ENSG00000104814	MAP4K1	3.00643787163122	2.44495137352056e-32

ENSG00000215612	HMX1	3.00635292802831	0.00699952480609527
ENSG00000151468	CCDC3	3.00323355794018	0.0014136088956121
ENSG00000162551	ALPL	3.00023353703048	1.60419545609848e-08
ENSG00000090339	ICAM1	2.99908466798185	8.73953837771486e-38
ENSG00000159784	FAM131B	2.99849002801707	2.28890194541095e-07
ENSG00000179148	ALOXE3	2.99581917848486	7.0038502476657e-13
ENSG00000203804	ADAMTSL4-AS1	2.99318785933966	0.000461673606152961
ENSG00000107954	NEURL1	2.99121261225244	6.28359451253029e-11
ENSG00000266278	N.A.	2.99040250747929	1.1283756606609e-10
ENSG00000156687	UNC5D	2.98699770373734	3.5087998407778e-05
ENSG00000259172	N.A.	2.98542482595951	1.29228867098711e-12
ENSG00000134716	CYP2J2	2.9852190628962	0.000151493234639915
ENSG00000197580	BCO2	2.97579249788882	1.24238713259937e-45
ENSG00000143847	PPFIA4	2.9727724734	5.13173268023375e-09
ENSG00000170745	KCNS3	2.97195647089647	2.83185916139299e-17
ENSG00000181418	DDN	2.96967636529775	4.15732427942945e-14
ENSG00000172985	SH3RF3	2.96439311028414	0.00872289556246687
ENSG00000157851	DPYSL5	2.96389757513899	4.27158064223088e-92
ENSG00000054179	ENTPD2	2.96224065948079	3.73325135499552e-51
ENSG00000163884	KLF15	2.96056329008629	0.00267217545843389
ENSG00000126583	PRKCG	2.95966476213516	8.04959730468193e-60
ENSG00000151224	MAT1A	2.95677460678788	0.00051771298209953
ENSG00000010319	SEMA3G	2.95252191489462	1.56894950815793e-09
ENSG00000072818	ACAP1	2.95228573146922	1.14520569504599e-07
ENSG00000197153	HIST1H3J	2.95155062449615	0.000711207939155373
ENSG00000088340	FER1L4	2.95121065071501	6.91496737523587e-67
ENSG00000260360	N.A.	2.94809660747849	0.000573410150522286
ENSG00000131849	ZNF132	2.94638030628083	0.00856923229072944
ENSG00000167608	TMC4	2.94475999806004	2.57876024994051e-29
ENSG00000272717	N.A.	2.94349306937995	0.00080544243596529
ENSG00000102996	MMP15	2.94054393681282	1.26084862861321e-270
ENSG00000165548	TMEM63C	2.93980050418914	1.34208126131281e-53
ENSG00000245552	N.A.	2.9396609833351	0.000162800670000842
ENSG00000166446	CDYL2	2.93825821740781	1.93405932595278e-08
ENSG00000162645	GBP2	2.9353056493497	2.9234172879506e-103
ENSG00000147117	ZNF157	2.9336688451117	0.002286005094911
ENSG00000260886	TAT-AS1	2.93359892914976	4.4207579013424e-05
ENSG00000261549	N.A.	2.92942469201212	3.53889594915284e-08
ENSG00000104808	DHDH	2.92780834574134	1.06811515889073e-07
ENSG00000274180	NATD1	2.92513073551644	1.87809711572228e-184
ENSG00000259687	LINC01220	2.9223402240756	0.00210165552703826
ENSG00000101222	SPEF1	2.91618440837992	2.19644255837747e-13
ENSG00000258655	ARHGAP5-AS1	2.91608337320165	2.19069263168295e-92
ENSG00000257830	N.A.	2.91428453576786	0.000111118480363003
ENSG00000167103	PIP5KL1	2.91328388344115	4.02448113533841e-18
ENSG00000272688	N.A.	2.91182664862782	2.6982812163934e-30
ENSG00000280426	N.A.	2.91182440177658	2.10439478678223e-08
ENSG00000137098	SPAG8	2.91165591666213	3.01054373957844e-07
ENSG00000069812	HES2	2.91136861011748	8.8231794879158e-10

ENSG00000106123	EPHB6	2.91117221424577	1.56898154955311e-06
ENSG00000163293	NIPAL1	2.90409913794069	2.15052802886265e-14
ENSG00000196368	NUDT11	2.9025402317146	4.56315355838229e-16
ENSG00000125931	CITED1	2.90166853360945	9.76177694412928e-06
ENSG00000186642	PDE2A	2.8988695833701	1.61950501160672e-07
ENSG00000182687	GALR2	2.8984755464417	8.22305855583611e-07
ENSG00000273702	N.A.	2.89813254533804	1.76023311274171e-38
ENSG00000091536	MYO15A	2.89570225172093	0.000618188900038849
ENSG00000186326	RGS9BP	2.89479292319628	1.76499369649722e-11
ENSG00000187193	MT1X	2.89459019899187	9.84661314626316e-59
ENSG00000175352	NRIP3	2.89242704829249	4.2119497983906e-51
ENSG00000183578	TNFAIP8L3	2.89239111635909	0.00582716479927633
ENSG00000111181	SLC6A12	2.89228109046098	0.0082948467686172
ENSG00000272081	N.A.	2.88971877539136	0.00955397984601231
ENSG00000273456	N.A.	2.88620129042976	6.80836033265848e-07
ENSG00000167748	KLK1	2.88579240859785	0.00018167060156585
ENSG00000272121	N.A.	2.88309399549027	4.1556812015298e-18
ENSG00000183287	CCBE1	2.88080509183303	3.77790973795154e-06
ENSG00000226386	PARD3-AS1	2.88005940267457	0.000963954950380887
ENSG00000243224	N.A.	2.87920553580202	3.09508973246237e-07
ENSG00000185614	FAM212A	2.87835157358228	0.000942222209335175
ENSG00000225173	N.A.	2.87797238193086	0.000102394057229687
ENSG00000260430	N.A.	2.87732369978845	1.60430894469109e-09
ENSG00000184678	HIST2H2BE	2.87701369515983	2.83510196374312e-94
ENSG00000188760	TMEM198	2.87693383554353	0.000607786453997718
ENSG00000153253	SCN3A	2.8769194102457	2.71788553472667e-16
ENSG00000120051	CFAP58	2.8763598847882	0.00569616902395472
ENSG00000125531	C20orf195	2.87632506762214	0.00324922601836695
ENSG00000228742	N.A.	2.87320251269036	1.3533030183916e-16
ENSG00000138347	MYPN	2.87141695845903	3.23875360616387e-127
ENSG00000023892	DEF6	2.87108756884547	0.000257155578350836
ENSG00000225968	ELFN1	2.86908249036784	0.000802707874426483
ENSG00000257285	N.A.	2.86823321224802	0.00270158081903985
ENSG00000213420	GPC2	2.86752680551247	7.79613338259852e-33
ENSG00000272767	JMJD1C-AS1	2.86574152558717	4.74917164790199e-11
ENSG00000249228	N.A.	2.86499022134867	0.00167844915663613
ENSG00000179111	HES7	2.86152021015437	3.40339403045317e-13
ENSG00000261693	N.A.	2.86107736356736	0.000336659813829754
ENSG00000145198	VWA5B2	2.85642002634866	1.83650547484481e-45
ENSG00000269559	N.A.	2.84857880744603	3.16835260070686e-05
ENSG00000149489	ROM1	2.84779733479958	3.03443303302627e-22
ENSG00000145911	N4BP3	2.84569795978091	5.48508838680237e-10
ENSG00000112297	AIM1	2.84125909738895	1.69723520845417e-24
ENSG00000141458	NPC1	2.83622083174636	0
ENSG00000174469	CNTNAP2	2.83573245252714	0.000892951830025571
ENSG00000244119	PDCL3P4	2.83542414118711	0.00011367545078504
ENSG00000105538	RASIP1	2.83088657726976	2.0656703499191e-07
ENSG00000106852	LHX6	2.83062647819156	0.000273362276504381
ENSG00000266401	N.A.	2.82955072479635	2.27174048442584e-32

ENSG00000117643	MAN1C1	2.82902693351882	1.1461750004495e-46
ENSG00000139160	METTL20	2.82865163371212	5.09296115535719e-64
ENSG00000115590	IL1R2	2.82804149341286	0.000205467358781852
ENSG00000128340	RAC2	2.82270617046603	2.86434227022102e-05
ENSG00000278916	CEP83-AS1	2.8179401343109	7.38106389171624e-11
ENSG00000137198	GMPR	2.81662696560182	2.89872976481032e-29
ENSG00000070759	TESK2	2.81598056017267	5.00472686980701e-20
ENSG00000186862	PDZD7	2.81456086748528	8.66049085366489e-29
ENSG00000280161	N.A.	2.81363955590286	2.81224362439018e-11
ENSG00000228172	N.A.	2.81344120700938	0.00179555155747166
ENSG00000175161	CADM2	2.81031752603501	1.04086413680743e-05
ENSG00000131650	KREMEN2	2.8101229071994	3.76413739066366e-17
ENSG00000149582	TMEM25	2.80911332801455	3.26998485825929e-13
ENSG00000133878	DUSP26	2.80598335663781	0.000634718638772535
ENSG00000226318	N.A.	2.80562277747951	1.17458889858046e-05
ENSG00000203722	RAET1G	2.80560917225287	5.67226558754452e-24
ENSG00000277159	N.A.	2.80325625183196	3.69918721933249e-06
ENSG00000160505	NLRP4	2.80319983880238	0.00168338604061803
ENSG00000135519	KCNH3	2.80294709173762	1.90904591975215e-96
ENSG00000124126	PREX1	2.80087651692109	1.29729749490304e-168
ENSG00000205362	MT1A	2.79838173049725	0.000191316673448712
ENSG00000197748	CFAP43	2.79615160748822	6.29154217853234e-10
ENSG00000128512	DOCK4	2.79215597921347	5.35249632020758e-136
ENSG00000125148	MT2A	2.79137377735867	1.63051335657784e-153
ENSG00000011332	DPF1	2.78929801503328	1.23147861005368e-42
ENSG00000120658	ENOX1	2.78408860135913	1.80987542796617e-17
ENSG00000203711	C6orf99	2.7805302526794	1.86510992220774e-18
ENSG00000136014	USP44	2.77694354368821	4.32220591751466e-11
ENSG00000273320	N.A.	2.77654086986153	1.157159423739e-05
ENSG00000278828	HIST1H3H	2.77188475521842	1.73017036673113e-51
ENSG00000130829	DUSP9	2.76818233258328	8.98495363367818e-08
ENSG00000099139	PCSK5	2.7679888521479	1.1045156400539e-21
ENSG00000142235	LMTK3	2.7650490004248	4.16925370858384e-97
ENSG00000196834	POTEI	2.76220028069604	0.00463024129470123
ENSG00000159871	LYPD5	2.7587521795631	0.00019294206413979
ENSG00000184716	SERINC4	2.75850118624984	0.0080368681573099
ENSG00000273599	N.A.	2.75768262385716	0.00088506320820174
ENSG00000270177	N.A.	2.75740969748228	1.18771566891266e-13
ENSG00000115423	DNAH6	2.75156323718656	4.8129716563249e-09
ENSG00000225793	N.A.	2.7469380411555	2.94669022271329e-09
ENSG00000263823	N.A.	2.73983066507446	0.00309114508971329
ENSG00000163520	FBLN2	2.73766641056555	5.53751590615424e-05
ENSG00000246877	DNM1P35	2.73752297984419	0.000859806388490202
ENSG00000181690	PLAG1	2.72886226729271	1.29202856025424e-28
ENSG00000280649	N.A.	2.72708545670048	0.000320380923323258
ENSG00000161270	NPHS1	2.72463005499301	1.9968576380049e-28
ENSG00000255568	BRWD1-AS2	2.72450425305234	0.00135248725482315
ENSG00000095397	DFNB31	2.72328004765948	1.50392589122685e-51
ENSG00000129991	TNNI3	2.72306001711237	4.62031253246093e-25

ENSG00000172350	ABCG4	2.72293058602001	5.63260842153599e-10
ENSG00000140678	ITGAX	2.72208128437272	5.38550161201657e-16
ENSG00000281189	GHET1	2.72132791835726	7.21222200464235e-20
ENSG00000184451	CCR10	2.72108451518274	0.00250393645866364
ENSG00000130477	UNC13A	2.72009059610493	4.39632980894838e-80
ENSG00000146233	CYP39A1	2.71913995216396	0.000255600659243536
ENSG00000143001	TMEM61	2.7127685754353	0.000101918129604799
ENSG00000133069	TMCC2	2.7069586477355	2.2938894903642e-54
ENSG00000058091	CDK14	2.70245906268362	0.00602168214985774
ENSG00000234028	N.A.	2.70222577864055	9.71833195670467e-24
ENSG00000258940	N.A.	2.69982299109887	8.75234608483088e-10
ENSG00000143355	LHX9	2.69944275278026	5.50916317390874e-05
ENSG00000171004	HS6ST2	2.69775275429961	0.00706049577359948
ENSG00000102109	PCSK1N	2.69606402526019	2.33832382918241e-76
ENSG00000214595	EML6	2.69600925175051	8.33145472707608e-49
ENSG00000264235	N.A.	2.69442527391355	8.32604475985929e-07
ENSG00000281912	LINC01144	2.69247604763831	0.000245798450722576
ENSG00000154146	NRGN	2.6879621493171	9.50354286965838e-89
ENSG00000164061	BSN	2.68675578108472	7.57931769160042e-09
ENSG00000168913	ENHO	2.68622123968032	1.83940269164568e-06
ENSG00000184481	FOXO4	2.68498676233485	8.71152821965738e-100
ENSG00000158813	EDA	2.68469337901881	4.46094224002413e-28
ENSG00000255621	N.A.	2.6818645234713	0.000749602034717736
ENSG00000147036	LANCL3	2.67668104744598	1.05980006460873e-06
ENSG00000186198	SLC51B	2.67664569720663	1.15129726342386e-11
ENSG00000161682	FAM171A2	2.67452907616676	1.44570206982104e-87
ENSG00000196787	HIST1H2AG	2.67327652212455	2.37218436683918e-55
ENSG00000173926	MARCH3	2.67297414890167	4.52429857817646e-16
ENSG00000274425	N.A.	2.67082750392938	2.40629957901279e-22
ENSG00000144406	UNC80	2.67050562985157	0.00093360221617717
ENSG00000271780	N.A.	2.66988441625189	1.37726326630716e-11
ENSG00000130304	SLC27A1	2.66962482329494	1.67037719484275e-88
ENSG00000149131	SERPING1	2.66917036378698	0.0080609228043919
ENSG00000164176	EDIL3	2.66451868094942	2.96990796666174e-21
ENSG00000270681	N.A.	2.66250786491347	0.00193515062374471
ENSG00000137880	GCHFR	2.65624571583745	3.15648553663114e-35
ENSG00000233822	HIST1H2BN	2.6546946152139	1.45792095506862e-18
ENSG00000165972	CCDC38	2.65310892749839	2.20617040154452e-05
ENSG00000233785	N.A.	2.65244830284225	0.000172555203577834
ENSG00000105641	SLC5A5	2.64561991637733	2.23471871980845e-08
ENSG00000167555	ZNF528	2.64468390037787	0.00176731026448801
ENSG00000164951	PDP1	2.6432204429844	8.12129847263627e-221
ENSG00000189060	H1F0	2.64131787216435	0
ENSG00000255571	LINC00925	2.64026050519063	0.000911759042837391
ENSG00000274976	N.A.	2.6337925116577	0.00157793829506125
ENSG00000223891	OSER1-AS1	2.63340177641829	2.10246724824879e-30
ENSG00000184602	SNN	2.63218196691214	5.82888106919271e-108
ENSG00000213760	ATP6V1G2	2.63141831302786	5.26288875668161e-07
ENSG00000171951	SCG2	2.63046985520938	3.60631230633941e-21

ENSG00000116661	FBXO2	2.62904950209074	1.08052476911162e-21
ENSG00000280399	N.A.	2.62776130792147	0.000386781460259314
ENSG00000204060	FOXO6	2.62705054467711	2.02281221530861e-08
ENSG00000130783	CCDC62	2.62529378233805	0.0033844483563058
ENSG00000059804	SLC2A3	2.62147789600793	0
ENSG00000100116	GCAT	2.62117394820178	5.2218604011375e-46
ENSG00000118402	ELOVL4	2.61894559136596	9.82387348100566e-96
ENSG00000126878	AIF1L	2.61807409764031	3.92128099433082e-67
ENSG00000267549	N.A.	2.6127623268738	0.000588342697248759
ENSG00000101460	MAP1LC3A	2.61163548747491	4.86944458174962e-84
ENSG00000228903	RASA4CP	2.61141482989259	4.17949992747226e-07
ENSG00000259315	ACTG1P17	2.61086846879234	2.59981381736068e-05
ENSG00000279227	N.A.	2.61032169295922	4.37692888181345e-11
ENSG00000188659	SAXO2	2.60962935616666	9.81591678271204e-14
ENSG00000188322	SBK1	2.60962635671635	7.76835016197894e-22
ENSG00000099954	CECR2	2.60736953345269	3.11280143812038e-10
ENSG00000127507	ADGRE2	2.6062278371365	7.66635981592542e-07
ENSG00000180818	HOXC10	2.60338921005768	3.62815076045077e-24
ENSG00000267041	ZNF850	2.60227991431768	3.0383747763473e-14
ENSG00000166924	NYAP1	2.60134331007474	2.93575550222895e-25
ENSG00000228701	TNKS2-AS1	2.59990913337427	0.00030568191441419
ENSG00000170542	SERPINB9	2.59918465758416	5.97067620770437e-279
ENSG00000260367	N.A.	2.59591505653001	0.00989404942971754
ENSG00000141526	SLC16A3	2.59238946301893	3.27169388616683e-220
ENSG00000206344	HCG27	2.59115735547104	2.34278126136125e-13
ENSG00000185634	SHC4	2.59095413480745	3.90234350590465e-07
ENSG00000272902	N.A.	2.59056726370669	1.12441499018754e-14
ENSG00000227051	C14orf132	2.59047069123107	7.32218227129219e-26
ENSG00000278002	N.A.	2.58901801075328	0.00859315620100326
ENSG00000168874	ATOH8	2.58859758092469	0.00083903886925907
ENSG00000160678	S100A1	2.58851869052253	3.42005023215372e-05
ENSG00000222043	N.A.	2.58618295323481	0.000153732335343282
ENSG00000100626	GALNT16	2.58582553549671	5.22928572043202e-31
ENSG00000184068	N.A.	2.58564595040344	0.000612578780222623
ENSG00000177138	FAM9B	2.58511875140489	0.000851901035092698
ENSG00000218274	N.A.	2.58108075989919	0.00384148505936509
ENSG00000277369	N.A.	2.58026529578718	1.66760577470205e-06
ENSG00000141505	ASGR1	2.58001464761925	0.000260901967212162
ENSG00000281205	LINC00950	2.57935809177628	9.8695619831672e-09
ENSG00000185291	IL3RA	2.57878853022635	3.93006721769085e-07
ENSG00000060140	STYK1	2.57332233129168	2.13193237273505e-12
ENSG00000105877	DNAH11	2.57111112278013	1.46390302500751e-07
ENSG00000259802	N.A.	2.56863615814034	1.85343882285456e-19
ENSG00000166323	C11orf65	2.56841486898394	0.000337616745280477
ENSG00000166448	TMEM130	2.56797730498824	0.0014113184880494
ENSG00000129214	SHBG	2.56650931693693	2.58965310164363e-07
ENSG00000169093	ASMTL	2.56526016334626	1.21393925267919e-238
ENSG00000227543	SPAG5-AS1	2.56285921169464	1.36892191729135e-06
ENSG00000099866	MADCAM1	2.5627144476739	0.0015660008491426

ENSG00000172346	CSDC2	2.5606346292972	1.34268131090972e-05
ENSG00000260018	N.A.	2.55891829571945	6.13403740001061e-07
ENSG00000231770	TMEM44-AS1	2.55798066586111	4.05666861234847e-31
ENSG00000070729	CNGB1	2.55252810793903	0.000103495666682972
ENSG00000225077	LINC00337	2.55010532498529	1.02210309969754e-12
ENSG00000160396	HIPK4	2.54927280576608	0.00053929873057026
ENSG00000168490	PHYHIP	2.54927185666726	5.14858313466778e-05
ENSG00000142459	EVI5L	2.54851399994	1.52613495646238e-124
ENSG00000177666	PNPLA2	2.54781188699007	0
ENSG00000174871	CNIH2	2.54727541018522	5.75085892631939e-07
ENSG00000261889	N.A.	2.54375919477466	0.000150171873378183
ENSG00000158555	GDPD5	2.54208785032617	9.95260068550363e-179
ENSG00000111254	AKAP3	2.54034038428851	6.52157769701174e-07
ENSG00000127954	STEAP4	2.54011897396491	8.38860142614554e-09
ENSG00000156299	TIAM1	2.53951764777478	1.12863214953871e-45
ENSG00000069188	SDK2	2.53924944522977	5.00398664311507e-06
ENSG00000168026	TTC21A	2.53821491089225	8.35126585226475e-31
ENSG00000166592	RRAD	2.53660894417025	0.000267802041663416
ENSG00000117595	IRF6	2.53633903155054	2.88714215918309e-07
ENSG00000176236	C10orf111	2.53554291528474	0.0020741811872649
ENSG00000176490	DIRAS1	2.53476167050675	1.37688256033852e-28
ENSG00000108840	HDAC5	2.53306076416105	3.19648266139658e-286
ENSG00000267291	N.A.	2.53224099324159	9.43160965373346e-10
ENSG00000271646	N.A.	2.53021378237302	1.20682189666267e-11
ENSG00000125730	C3	2.52848843367164	9.38101837061054e-53
ENSG00000178199	ZC3H12D	2.52668991626031	0.00411816050903612
ENSG00000058335	RASGRF1	2.52557254199739	0.000805394992383432
ENSG00000049192	ADAMTS6	2.52525121389926	6.52364549961036e-11
ENSG00000185130	HIST1H2BL	2.52337098427732	0.00040949493177161
ENSG00000180448	HMHA1	2.51597766590296	1.38451462696453e-17
ENSG00000246465	N.A.	2.51592876391679	0.000596077481014978
ENSG00000179855	GIPC3	2.51503808341215	0.000443706664581999
ENSG00000173227	SYT12	2.51491224057754	3.17668093870251e-26
ENSG00000243710	CFAP57	2.51371991233741	1.45819326089704e-14
ENSG00000188404	SELL	2.51328773213792	0.00375582178820211
ENSG00000260025	N.A.	2.51240518752569	9.26844669036983e-38
ENSG00000167772	ANGPTL4	2.51062107389026	3.01431801584894e-186
ENSG00000135218	CD36	2.51022567466518	0.00148588938282466
ENSG00000143375	CGN	2.50797877848509	6.76737874530031e-293
ENSG00000203952	CCDC160	2.50698521968498	3.88122244709989e-11
ENSG00000205809	KLRC2	2.50531775296745	2.96442830879041e-30
ENSG00000185002	RFX6	2.50528140918788	0.00711351666869443
ENSG00000125657	TNFSF9	2.50485760727911	1.53100018172782e-256
ENSG00000181804	SLC9A9	2.5044842828822	0.00751707547729704
ENSG00000280710	N.A.	2.50265961002777	0.00113553944273298
ENSG00000215246	N.A.	2.50252716901925	0.000103510040093113
ENSG00000247363	N.A.	2.50082129190615	9.63621079164339e-05
ENSG00000254531	N.A.	2.50035231741514	1.22478458670098e-39
ENSG00000197980	LEKR1	2.49831826193724	3.21781349273961e-10

ENSG00000138622	HCN4	2.496970964615	2.50724984847351e-07
ENSG00000133863	TEX15	2.49508463250669	1.31591450606277e-15
ENSG00000099625	C19orf26	2.49329614929765	4.70464654634361e-14
ENSG00000232677	LINC00665	2.4929625007493	2.79927557326713e-15
ENSG00000250241	N.A.	2.48719969289904	0.00691104710818705
ENSG00000135436	FAM186B	2.48574489377228	0.000213947410364895
ENSG00000165182	CXorf58	2.48447449317183	0.0013183060329516
ENSG00000150054	MPP7	2.48374235457424	3.03878547182116e-42
ENSG00000100592	DAAM1	2.4837272237089	8.80530604699572e-182
ENSG00000196415	PRTN3	2.48351423198402	0.000861198486880473
ENSG00000162643	WDR63	2.48288633529523	2.5002517849082e-10
ENSG00000168386	FILIP1L	2.48160251178361	0.00252336240689196
ENSG00000135414	GDF11	2.48153760880829	1.57909645548838e-247
ENSG00000273080	N.A.	2.4802331506931	4.72285400900559e-05
ENSG00000180537	RNF182	2.47893533834485	5.26164431935472e-06
ENSG00000226754	N.A.	2.47538814554738	0.000521264985833014
ENSG00000105479	CCDC114	2.46913461700331	2.16832419044705e-08
ENSG00000226913	BSN-AS2	2.46662089405313	0.00247172265439568
ENSG00000189184	PCDH18	2.46520718317956	3.31896168730582e-06
ENSG00000151572	ANO4	2.4636634891903	0.00703694262214658
ENSG00000143382	ADAMTSL4	2.45412241177595	8.32459683840019e-15
ENSG00000139200	PIANP	2.45342743297323	5.31994223022127e-08
ENSG00000236617	N.A.	2.45287785956497	3.82167460218856e-05
ENSG00000258334	N.A.	2.45264933401019	3.6221126703707e-06
ENSG00000171451	DSEL	2.45193759189324	4.716586468998e-39
ENSG00000099338	CATSPERG	2.45189667447859	6.15145932821168e-14
ENSG00000095383	TBC1D2	2.45178482271729	7.02558950910107e-60
ENSG00000221968	FADS3	2.45025375687759	1.57251861729131e-160
ENSG00000108821	COL1A1	2.44954843008207	4.60952974324004e-94
ENSG00000143858	SYT2	2.4486323327196	9.57339707028599e-05
ENSG00000182791	CCDC87	2.44779928052704	5.80506018513017e-13
ENSG00000159450	TCHH	2.44438856278091	7.71181736063173e-10
ENSG00000261737	N.A.	2.4422588913074	0.00887404779631333
ENSG00000116852	KIF21B	2.44191049015021	4.42088192301008e-140
ENSG00000112182	BACH2	2.43780600296998	9.66537175488691e-05
ENSG00000146411	SLC2A12	2.43658966143958	1.66362712472207e-26
ENSG00000275481	N.A.	2.43401680144975	8.57175695529269e-14
ENSG00000214796	N.A.	2.43133652702132	1.59385883581777e-09
ENSG00000189127	ANKRD34B	2.42835260846233	0.000872180075194228
ENSG00000131095	GFAP	2.42499203537851	2.22851152649289e-05
ENSG00000135643	KCNMB4	2.42279437861517	7.23620827153102e-18
ENSG00000130830	MPP1	2.42049389408189	2.13509686186252e-97
ENSG00000160188	RSPH1	2.42034190780588	1.91747593103789e-14
ENSG00000105472	CLEC11A	2.41871734099507	2.30655943378543e-34
ENSG00000160050	CCDC28B	2.41869831727278	2.99546681135147e-16
ENSG00000111729	CLEC4A	2.41608628586741	0.00516806349595301
ENSG00000164627	KIF6	2.41602773251177	9.82328731878377e-05
ENSG00000163659	TIPARP	2.4129290716002	0
ENSG00000134827	TCN1	2.41288180911033	4.96029516672052e-16

ENSG00000078018	MAP2	2.41018335231539	6.13930490799194e-33
ENSG00000155530	LRGUK	2.40764222337981	4.3181814166863e-08
ENSG00000173210	ABLIM3	2.40735017513658	6.01407494104121e-34
ENSG00000124406	ATP8A1	2.40728850192644	8.42962226660173e-30
ENSG00000213073	N.A.	2.40537169636248	2.84880268545215e-09
ENSG00000178075	GRAMD1C	2.40505027177824	2.73206768996111e-11
ENSG00000117707	PROX1	2.40313725188846	7.28961099150964e-10
ENSG00000135838	NPL	2.39814921278337	5.2230351047436e-11
ENSG00000061918	GUCY1B3	2.39773011216852	3.750268523421e-42
ENSG00000100167	SEPT3	2.39437205510559	4.02860565746248e-41
ENSG00000257704	INAFM1	2.38996114877251	1.59155197155869e-72
ENSG00000104368	PLAT	2.38995982625281	4.1678337814144e-21
ENSG00000173805	HAP1	2.38642567624278	4.87757224984059e-41
ENSG00000273654	N.A.	2.38223193627378	1.37106919039299e-08
ENSG00000146250	PRSS35	2.38117979225923	8.25213966949588e-14
ENSG00000025708	TYMP	2.38060240900885	5.93935482771049e-10
ENSG00000117115	PADI2	2.37979568448104	1.08224511209669e-177
ENSG00000124813	RUNX2	2.37828619167749	0.00104100359484022
ENSG00000167100	SAMD14	2.37674710381475	4.58829344420432e-08
ENSG00000230790	N.A.	2.37559015416746	0.00372167391559476
ENSG00000258102	MAP1LC3B2	2.37098108687986	0.00122102857351786
ENSG00000258900	HNRNPCP1	2.36891473488648	5.41987948947955e-06
ENSG00000153982	GDPD1	2.36739631117973	5.15013426252047e-42
ENSG00000072041	SLC6A15	2.36722568690842	8.6698313527436e-72
ENSG00000135127	CCDC64	2.36582334547056	2.96881302721948e-129
ENSG00000257135	N.A.	2.36269360063304	0.00199067492936771
ENSG00000164442	CITED2	2.35884938013295	9.71474356192444e-147
ENSG00000109321	AREG	2.35876624303066	2.81026175034855e-283
ENSG00000131471	AOC3	2.35822085790363	8.39423862273541e-16
ENSG00000168546	GFRA2	2.35818935006796	0.00243720466067597
ENSG00000072310	SREBF1	2.3565742277546	0
ENSG00000272273	N.A.	2.35156223120416	1.39749133684658e-07
ENSG00000204869	IGFL4	2.35074112963314	4.49825215625934e-05
ENSG00000180998	GPR137C	2.34735903893393	1.06517979106575e-19
ENSG00000225434	LINC01504	2.34181534820039	1.17677250366867e-56
ENSG00000185015	CA13	2.34158852706816	3.01271275987345e-10
ENSG00000196388	INCA1	2.34137277926824	7.25760523181308e-17
ENSG00000237017	N.A.	2.34005306266165	0.00687823846631019
ENSG00000112964	GHR	2.33911672163656	0.00590500800714018
ENSG00000175414	ARL10	2.33645697709326	0.0095186887653181
ENSG00000198417	MT1F	2.33611569607945	2.40629957901279e-22
ENSG00000137571	SLCO5A1	2.33577893241007	8.30016855837617e-09
ENSG00000272686	N.A.	2.33569183143288	1.64005732476638e-55
ENSG00000238287	N.A.	2.33486955387915	0.00520129343192203
ENSG00000273619	N.A.	2.33311678198864	3.32216061857533e-11
ENSG00000165309	ARMC3	2.33213738814859	5.54657469098021e-07
ENSG00000268129	N.A.	2.33032849049602	9.62490885406641e-13
ENSG00000249641	HOXC13-AS	2.32694402274349	0.00145606922493259
ENSG00000119950	MXI1	2.32690376468002	1.08349973644317e-100

ENSG00000103184	SEC14L5	2.32643184332983	0.000124967248975508
ENSG00000150625	GPM6A	2.32491885223984	4.75673465202845e-05
ENSG00000103599	IQCH	2.32334248230856	5.15663756253431e-45
ENSG00000204624	PTCHD2	2.32063748542827	0.00473165605977168
ENSG00000164128	NPY1R	2.31885329625176	7.69765671135794e-08
ENSG00000113083	LOX	2.3162815541904	1.68722906055234e-05
ENSG00000150672	DLG2	2.31395007825348	2.32680117792994e-06
ENSG00000132970	WASF3	2.31372359469483	6.96227237722677e-13
ENSG00000123989	CHPF	2.31336525173789	7.87533345234795e-134
ENSG00000110002	VWA5A	2.31295347776247	9.36482473900392e-18
ENSG00000124635	HIST1H2BJ	2.31092959607343	5.46658898420191e-31
ENSG00000087303	NID2	2.3085689761384	4.22228259945698e-82
ENSG00000176244	ACBD7	2.3083701183787	4.14218073328334e-31
ENSG00000230457	PA2G4P4	2.30744865017682	0.00507616243118873
ENSG00000177380	PPFIA3	2.30644616684118	3.43419000423872e-55
ENSG00000174938	SEZ6L2	2.30535777103729	3.80477257544392e-144
ENSG00000172986	GXYLT2	2.30010961595098	9.25945270212613e-14
ENSG00000118004	COLEC11	2.29752401681297	0.000428310971833581
ENSG00000272870	N.A.	2.29533151738661	9.82280594730421e-10
ENSG00000257913	N.A.	2.29423865111643	2.86100535785571e-25
ENSG00000130584	ZBTB46	2.29383734907434	2.78043613020413e-18
ENSG00000141540	TTYH2	2.29375384343512	2.56614865136159e-11
ENSG00000106789	CORO2A	2.290229156875	1.24635876370577e-66
ENSG00000232850	PTGES2-AS1	2.2883819697891	3.43444985650987e-05
ENSG00000242759	LINC00882	2.28824429425307	5.82147047670432e-08
ENSG00000240875	LINC00886	2.28544279821077	3.68982924709578e-55
ENSG00000109062	SLC9A3R1	2.28438910612882	0
ENSG00000087884	AAMDC	2.28265448888501	8.45109514282745e-77
ENSG00000242265	PEG10	2.28238044641355	0
ENSG00000165695	AK8	2.27874566950083	2.49951682297884e-10
ENSG00000178977	LINC00324	2.27594741889335	5.25345760012775e-08
ENSG00000278238	N.A.	2.27457084174966	1.16532928901855e-06
ENSG00000270127	N.A.	2.27374342946923	8.12667753111263e-06
ENSG00000168631	DPCR1	2.26659782444083	0.000193022137082901
ENSG00000012779	ALOX5	2.26629147185416	1.07033095420833e-12
ENSG00000103316	CRYM	2.2634274556615	1.69895931843283e-63
ENSG00000163249	CCNYL1	2.26340766851778	0
ENSG00000183018	SPNS2	2.26135159592115	9.09922406528352e-44
ENSG00000156515	HK1	2.25963625301446	0
ENSG00000138080	EMILIN1	2.25752298645736	0.00216681990597311
ENSG00000270882	HIST2H4A	2.25550015280385	0.00154330968788603
ENSG00000275216	N.A.	2.25504075766155	7.19545255941239e-196
ENSG00000181218	HIST3H2A	2.25499341315639	3.71606072105406e-30
ENSG00000163644	PPM1K	2.25343289803524	3.08372386758721e-37
ENSG00000248712	CCDC153	2.25241893179957	3.69640299823505e-05
ENSG00000164794	KCNV1	2.25120171261976	1.09431321689252e-12
ENSG00000181652	ATG9B	2.25009139872685	0.000502462208240008
ENSG00000259660	DNM1P47	2.2451250250888	0.00471148654029734
ENSG00000167676	PLIN4	2.24476018917006	1.23675198381772e-18

ENSG00000149926	FAM57B	2.24143016237296	0.000137766368129896
ENSG00000105327	BBC3	2.24078583598918	6.53334785506687e-70
ENSG00000205143	ARID3C	2.23907447604789	0.00582743041653428
ENSG00000162444	RBP7	2.23803067230372	1.65113761319544e-07
ENSG00000259205	PRKXP1	2.2365210890184	0.000162754139341899
ENSG00000189221	MAOA	2.23599857997024	2.18615901609966e-49
ENSG00000249602	N.A.	2.23431437886909	0.000219415218885972
ENSG00000105808	RASA4	2.23393413778612	7.18972196104301e-28
ENSG00000123384	LRP1	2.23355719587686	6.94425750712082e-302
ENSG00000186212	SOWAHB	2.22993286932569	0.000206608580132618
ENSG00000125266	EFNB2	2.2254445571079	2.07076943616308e-35
ENSG00000169427	KCNK9	2.22485377641638	0.00206778006162726
ENSG00000143344	RGL1	2.2241265804703	1.08928053627307e-103
ENSG00000272143	FGF14-AS2	2.22300482938614	1.5003206277545e-05
ENSG00000167680	SEMA6B	2.22176399999655	3.17996932566711e-58
ENSG00000086506	HBQ1	2.22077489683962	3.29555687381775e-07
ENSG00000273117	N.A.	2.22032015974486	1.07227646898689e-41
ENSG00000245248	USP2-AS1	2.2201717519419	1.01278187893382e-05
ENSG00000244968	LIFR-AS1	2.21914283548577	2.57162802224047e-06
ENSG00000235314	LINC00957	2.21877468566199	1.8222359074181e-08
ENSG00000087338	GMCL1	2.21776926986447	6.57048107272755e-281
ENSG00000179331	RAB39A	2.21756050982416	7.15032384854947e-05
ENSG00000090238	YPEL3	2.217156474664	9.39006778313151e-32
ENSG00000178033	FAM26E	2.21513679889869	7.67159451396801e-09
ENSG00000124374	PAIP2B	2.21181803121343	1.2198694156708e-77
ENSG00000266947	N.A.	2.20893151724201	2.96673021436005e-10
ENSG00000150628	SPATA4	2.20776485579323	0.000629799128904637
ENSG00000064687	ABCA7	2.20547267280643	5.23669464865316e-257
ENSG00000154479	CCDC173	2.20462684211017	0.00298280602658174
ENSG00000167771	RCOR2	2.20445030190453	3.02361344035589e-22
ENSG00000166165	CKB	2.20230568252315	0
ENSG00000273821	N.A.	2.20172605232208	0.0087504188221476
ENSG00000267432	DNAH17-AS1	2.19978754271667	0.000380240919611328
ENSG00000206559	ZCWPW2	2.19929363321371	0.00125300656808486
ENSG00000149260	CAPN5	2.19242838142354	1.21300772992342e-145
ENSG00000143845	ETNK2	2.19237902023197	4.67464155844191e-72
ENSG00000136999	NOV	2.19235205634553	1.5842582135398e-07
ENSG00000260805	N.A.	2.19150106942165	1.24280943287574e-07
ENSG00000224843	LINC00240	2.19042845968097	0.00725989097077152
ENSG00000006606	CCL26	2.18944958815116	0.0011800965950811
ENSG00000204963	PCDHA7	2.18898269230172	0.00148306029495626
ENSG00000205403	CFI	2.18742247776759	1.27784651929436e-05
ENSG00000138311	ZNF365	2.18663386412004	5.51533935130488e-12
ENSG00000272468	N.A.	2.18632093175691	3.47660173488802e-09
ENSG00000263280	N.A.	2.18612931916813	0.00557498173936028
ENSG00000150347	ARID5B	2.18603556101412	2.72969008031586e-75
ENSG00000278385	N.A.	2.18577203518217	0.000178843790965831
ENSG00000243155	N.A.	2.1842524595238	0.00128183474215198
ENSG00000115194	SLC30A3	2.18338408173602	4.56809389804944e-27

ENSG00000172318	B3GALT1	2.18337838746147	2.74673068283142e-05
ENSG00000188660	LINC00319	2.18046519384285	0.00083903886925907
ENSG00000167733	HSD11B1L	2.17969384230321	7.00546343594047e-09
ENSG00000260063	N.A.	2.17740163780855	3.23487415021872e-08
ENSG00000204642	HLA-F	2.17676247710467	4.62859166163683e-20
ENSG00000099840	IZUMO4	2.17466967779827	4.10056181028768e-07
ENSG00000166257	SCN3B	2.17437939116073	7.63735618367964e-09
ENSG00000012822	CALCOCO1	2.17263652570349	9.641813329203e-258
ENSG00000230615	N.A.	2.17155636040461	3.97009306020639e-05
ENSG00000107551	RASSF4	2.1714163228477	2.85515561969575e-31
ENSG00000228709	N.A.	2.16931277501556	5.15942538429197e-15
ENSG00000008735	MAPK8IP2	2.16886588788497	6.76726250081551e-27
ENSG00000175155	YPEL2	2.16511619643815	6.27309281765363e-112
ENSG00000276180	HIST1H4I	2.16401549009887	4.20970880026519e-05
ENSG00000223573	TINCR	2.1615018197534	6.23917137533685e-09
ENSG00000268460	N.A.	2.16125687534501	1.14280612140915e-17
ENSG00000272288	N.A.	2.16095354263743	1.89307023935406e-10
ENSG00000001617	SEMA3F	2.15972229324901	7.42034370912141e-31
ENSG00000148344	PTGES	2.15896741799669	6.03728677559339e-193
ENSG00000203326	ZNF525	2.15889859555719	1.65296666166877e-06
ENSG00000119508	NR4A3	2.15885995100833	9.13975126386356e-33
ENSG00000164404	GDF9	2.15802549593264	7.73721296370718e-09
ENSG00000145192	AHSG	2.15755774826827	0.00318344511268377
ENSG00000274922	N.A.	2.15636196099664	1.82377460007604e-06
ENSG00000137103	TMEM8B	2.15479058195872	1.26175953270729e-52
ENSG00000156804	FBXO32	2.15432196067481	5.37685292550374e-24
ENSG00000271826	PLS3-AS1	2.1532330564333	4.23692354166694e-07
ENSG00000172426	RSPH9	2.15086154828121	0.000341501546945006
ENSG00000238279	N.A.	2.15036546199184	0.00131574295847908
ENSG00000249846	N.A.	2.14801644302318	0.00857409740070057
ENSG00000263563	UBBP4	2.14703429377796	0.00450808353836864
ENSG00000153291	SLC25A27	2.14566822652366	7.26255014602513e-13
ENSG00000166816	LDHD	2.14442614290625	5.125586404528e-32
ENSG00000282386	N.A.	2.1440613196649	3.62558360725489e-05
ENSG00000203867	RBM20	2.14345211427605	9.83278844548059e-72
ENSG00000142619	PADI3	2.14247599112048	1.67379459364996e-77
ENSG00000236307	EEF1E1P1	2.14134599304832	0.0036342833567997
ENSG00000223572	CKMT1A	2.14053896610716	4.84000782479579e-06
ENSG00000203819	HIST2H2BC	2.14013750296343	0.0077462150142301
ENSG00000124762	CDKN1A	2.13975589284557	0
ENSG00000185055	EFCAB10	2.13458074177364	7.70832408938395e-19
ENSG00000196878	LAMB3	2.1304020653134	3.77905680868047e-89
ENSG00000127124	HIVEP3	2.12839286253282	1.26899523434908e-18
ENSG00000205740	N.A.	2.12757036765559	1.17686828316493e-05
ENSG00000197381	ADARB1	2.12669203423875	1.73752863225092e-50
ENSG00000162676	GFI1	2.12615574701408	1.84585219558504e-06
ENSG00000166578	IQCD	2.12504565300471	4.44710006265356e-41
ENSG00000258099	N.A.	2.12334283547418	0.00112953890405961
ENSG00000173599	PC	2.12333756538548	8.23691987785009e-172

ENSG00000279491	N.A.	2.12333357474211	0.000134492794791961
ENSG00000176973	FAM89B	2.12278961870425	2.44851260414425e-31
ENSG00000213889	PPM1N	2.12224697933431	2.52796282317902e-31
ENSG00000279673	N.A.	2.12174836586514	1.48200689185168e-10
ENSG00000176092	AIM1L	2.11994461325528	1.53687806414428e-10
ENSG00000243819	RN7SL832P	2.11963833219001	2.76243586696745e-06
ENSG00000119686	FLVCR2	2.11954771808509	5.67941298452296e-10
ENSG00000196747	HIST1H2AI	2.1175080458703	1.10526366617909e-05
ENSG00000259772	N.A.	2.11746824740862	0.00729800219290279
ENSG00000106992	AK1	2.11737530454191	3.25531950543926e-40
ENSG00000256061	DYX1C1	2.11565643269153	8.52171907485546e-10
ENSG00000130827	PLXNA3	2.1121123557178	4.82300108383188e-91
ENSG00000131773	KHDRBS3	2.11067843078997	1.63623930839236e-12
ENSG00000156587	UBE2L6	2.10961017172814	4.10821083046209e-80
ENSG00000101220	C20orf27	2.10909602959481	3.22104106304056e-276
ENSG00000134317	GRHL1	2.10888590005238	1.16533581749254e-32
ENSG00000129355	CDKN2D	2.10470255306861	2.76370701880207e-47
ENSG00000072062	PRKACA	2.10402892706239	0
ENSG00000197943	PLCG2	2.10306230776883	1.64026021594501e-72
ENSG00000105737	GRIK5	2.09791008087835	4.83781121585635e-10
ENSG00000261286	N.A.	2.09753745088972	0.000122262566670134
ENSG00000130956	HABP4	2.09499978993143	9.08242718277019e-288
ENSG00000166145	SPINT1	2.09252159332167	3.65472425594857e-06
ENSG00000162729	IGSF8	2.09236522036188	5.23726576002928e-116
ENSG00000259426	N.A.	2.09172882851454	7.46916166122508e-05
ENSG00000084764	MAPRE3	2.09108789008189	8.17231070241667e-146
ENSG00000281186	LINC00706	2.09058891346452	0.000560348005236978
ENSG00000167619	TMEM145	2.08915090425233	2.70415315968816e-13
ENSG00000183873	SCN5A	2.08851242678937	4.43118809938417e-05
ENSG00000235944	ZNF815P	2.08820163895894	9.96506365299559e-09
ENSG00000271614	LINC00936	2.08732128864777	2.15719964149826e-21
ENSG00000277149	TYW1B	2.08653575879855	1.62822752828803e-35
ENSG00000121966	CXCR4	2.08608873224175	4.69511632009343e-07
ENSG00000171862	PTEN	2.08596362932097	0
ENSG00000170703	TTLL6	2.08503268651015	2.62722646274387e-77
ENSG00000105520	N.A.	2.08500444283914	4.41732794730138e-115
ENSG00000167895	TMC8	2.08291715370119	1.2481037740924e-11
ENSG00000162426	SLC45A1	2.08286626057221	6.48994175298861e-40
ENSG00000165886	UBTD1	2.0828529626628	8.91448548137308e-72
ENSG00000166073	GPR176	2.08276054258287	1.57382585081221e-11
ENSG00000107738	C10orf54	2.08268356862904	2.18520332556176e-203
ENSG00000264964	N.A.	2.0819101049271	0.000262395823809762
ENSG00000261934	PCDHGA9	2.07906640144988	0.00629397749993403
ENSG00000128683	GAD1	2.07820173237327	8.77962063820121e-06
ENSG00000168062	BATF2	2.07681925086729	0.000208411740823537
ENSG00000134490	TMEM241	2.07660744642118	3.68002299405968e-99
ENSG00000260231	JHDM1D-AS1	2.07567810562465	1.75538489849433e-18
ENSG00000253426	N.A.	2.07563504819348	0.000187236486804816
ENSG00000235300	N.A.	2.07539290589516	4.15026614369597e-09

ENSG00000114423	CBLB	2.07535839868355	1.83012068881168e-171
ENSG00000144339	TMEFF2	2.07341563594134	1.36283156652822e-28
ENSG00000128872	TMOD2	2.07211382885925	1.04952315876642e-58
ENSG00000115461	IGFBP5	2.07136052611677	0.000312635318483314
ENSG00000169258	GPRIN1	2.0709690346957	1.76646659174868e-93
ENSG00000219200	RNASEK	2.07029406862853	9.67422420917919e-19
ENSG00000274267	HIST1H3B	2.0698965301968	9.85081658010049e-06
ENSG00000260625	N.A.	2.06936835533263	0.00528235603698666
ENSG00000163624	CDS1	2.06918102571168	1.00621210537483e-41
ENSG00000187091	PLCD1	2.06642767487374	5.02508929046372e-55
ENSG00000226179	LINC00685	2.06587064021637	0.000397855535954017
ENSG00000163576	EFHB	2.06322567637205	6.73357990120185e-07
ENSG00000049768	FOXP3	2.05911217422063	0.00328567150408008
ENSG00000154096	THY1	2.05845957284593	1.09783154996475e-44
ENSG00000237813	N.A.	2.05752984633499	1.62780271670541e-06
ENSG00000134245	WNT2B	2.05454717824763	1.16134966219759e-07
ENSG00000130066	SAT1	2.0545134756355	0
ENSG00000107518	ATRNL1	2.05220401229288	1.27014076645091e-16
ENSG00000135709	KIAA0513	2.05169695618733	3.05693718926327e-85
ENSG00000237276	ANO7P1	2.05028951973195	2.97889331595128e-05
ENSG00000273284	N.A.	2.04957186836437	1.59426345729045e-08
ENSG00000270673	YTHDF3-AS1	2.0456966988475	3.10671229467027e-10
ENSG00000273478	N.A.	2.04545942330825	2.28381223096472e-10
ENSG00000236829	N.A.	2.04545321908076	0.000138367251111583
ENSG00000088280	ASAP3	2.04391962227616	1.42383322674135e-73
ENSG00000231305	N.A.	2.04217951817632	0.00474505508753975
ENSG00000269814	N.A.	2.04160397096079	0.00698810607767586
ENSG00000270761	N.A.	2.04073500760844	0.000462506134045296
ENSG00000281903	N.A.	2.03916120536811	0.00688960706928038
ENSG00000163376	KBTBD8	2.03747666362765	4.51138278489701e-18
ENSG00000261335	N.A.	2.03508793450932	3.18236595973476e-13
ENSG00000260339	HEXA-AS1	2.03499152473596	0.000328586847619816
ENSG00000158571	PFKFB1	2.03387161770579	0.00619762588061119
ENSG00000254389	RHPN1-AS1	2.03365686510176	5.75332001425443e-09
ENSG00000110046	ATG2A	2.03339197748104	1.6468570986932e-205
ENSG00000152779	SLC16A12	2.0317058179046	0.00053340799033041
ENSG00000172638	EFEMP2	2.02814495296834	4.09242045708192e-06
ENSG00000230513	THAP7-AS1	2.02595742289778	7.21886652826599e-10
ENSG00000225746	SNHG23	2.02086010866605	0.00170963508733851
ENSG00000243024	RPS11P6	2.02063007865328	0.00100590581784992
ENSG00000273382	N.A.	2.02033972341848	9.46119326100231e-18
ENSG00000068903	SIRT2	2.01952662895613	1.50547062977991e-216
ENSG00000168743	NPNT	2.01910725394365	0.000235154670918632
ENSG00000226853	N.A.	2.01679774993261	1.47056971383523e-05
ENSG00000137463	MGARP	2.01543424979292	0.00584841855106164
ENSG00000228192	N.A.	2.01463330739162	0.00113870393426497
ENSG00000132688	NES	2.01444792778634	8.98717255797609e-54
ENSG00000158292	GPR153	2.013314637213	1.28681294121968e-25
ENSG00000198835	GJC2	2.01293895632837	0.00234287853250742

ENSG00000125430	HS3ST3B1	2.01040664003826	5.89299290812055e-06
ENSG00000188613	NANOS1	2.0086323203276	9.52675680487302e-12
ENSG00000271888	N.A.	2.00850986051629	0.00627997935706593
ENSG00000035862	TIMP2	2.00691327211006	0
ENSG00000237289	CKMT1B	2.00555030029536	2.49756910493224e-08
ENSG00000259706	HSP90B2P	2.00349988286026	5.18778065836889e-05
ENSG00000234745	HLA-B	2.00151044550624	1.46005746192866e-86
ENSG00000127585	FBXL16	2.00095641741919	2.01730745015471e-19
ENSG00000255337	N.A.	2.00065647111087	0.00806962784044757
ENSG00000259523	N.A.	1.99878370659986	2.6985968785187e-12
ENSG00000109654	TRIM2	1.99860461195904	4.53899343305967e-42
ENSG00000249572	N.A.	1.997262622384	2.03511471987364e-06
ENSG00000187244	BCAM	1.99664841788426	0
ENSG00000119782	FKBP1B	1.99656446323193	2.44189267360648e-05
ENSG00000050344	NFE2L3	1.99442913188343	3.84215787263838e-91
ENSG00000267248	N.A.	1.99162179500806	3.59946279694505e-05
ENSG00000246560	N.A.	1.98816132397219	1.27557496757047e-06
ENSG00000163359	COL6A3	1.98650872785463	1.55543353700946e-05
ENSG00000277969	N.A.	1.98642071271997	2.61405977432554e-14
ENSG00000108813	DLX4	1.98466492741596	1.89249073187349e-33
ENSG00000244242	IFITM10	1.98116391176511	6.88503445811007e-24
ENSG00000230658	KLHL7-AS1	1.98004357358224	8.86270178939275e-10
ENSG00000159713	TPPP3	1.97793618107638	3.17447640178603e-16
ENSG00000021300	PLEKHB1	1.97591287836851	1.62983389480599e-08
ENSG00000116032	GRIN3B	1.97465472745039	1.07205148673834e-07
ENSG00000186994	KANK3	1.97371500840095	5.44535973474026e-10
ENSG00000101400	SNTA1	1.97214333845079	6.2975804408009e-85
ENSG00000246982	N.A.	1.97071746155616	0.00169804469246829
ENSG00000005249	PRKAR2B	1.97017785751928	2.13020194030014e-77
ENSG00000273340	MICE	1.97016966413438	0.00710741421982625
ENSG00000257327	N.A.	1.96747710559636	0.00183875319657205
ENSG00000171811	CFAP46	1.96728436730196	1.16120239062277e-05
ENSG00000239887	C1orf226	1.96640728501723	2.16502538763747e-24
ENSG00000176845	METRNL	1.96523042977837	4.20494045815922e-17
ENSG00000162040	HS3ST6	1.96242423928056	4.83575197890119e-08
ENSG00000103966	EHD4	1.96113617695839	0
ENSG00000154654	NCAM2	1.95997261380672	0.00360202364492388
ENSG00000246763	RGMB-AS1	1.95869309313498	0.00217445500072424
ENSG00000232160	RAP2C-AS1	1.9582028748216	1.33827968896796e-05
ENSG00000177679	SRRM3	1.95756447307623	6.75217525290389e-41
ENSG00000224046	N.A.	1.95741042075381	2.90119883089714e-06
ENSG00000072832	CRMP1	1.95551870127498	9.60827936694566e-16
ENSG00000153563	CD8A	1.95490186492772	0.00216959121066791
ENSG00000213445	SIPA1	1.95467462994438	2.39116002641724e-92
ENSG00000241839	PLEKHO2	1.95384465875216	8.49487726643693e-40
ENSG00000151743	AMN1	1.95379588743293	3.20630905205427e-153
ENSG00000279384	N.A.	1.95359640836497	0.00942852731167786
ENSG00000257433	N.A.	1.95042193094638	8.33021139055148e-06
ENSG00000130518	KIAA1683	1.94459332972966	4.1229032134939e-22

ENSG00000130203	APOE	1.94319034567513	3.1794593433607e-15
ENSG00000010295	IFFO1	1.9411573179539	0.00641526670504626
ENSG00000186019	N.A.	1.9372573015585	1.46472508813152e-08
ENSG00000245904	N.A.	1.936249643485	8.19039682740166e-06
ENSG00000173825	TIGD3	1.93468420603345	4.47858166574482e-10
ENSG00000270175	N.A.	1.93415142738177	1.99643517310098e-08
ENSG00000275494	N.A.	1.93334839336286	0.000153044940362732
ENSG00000095596	CYP26A1	1.93316951589832	0.000996238279084654
ENSG00000236753	MKLN1-AS	1.9323975716837	2.54086170839845e-32
ENSG00000113211	PCDHB6	1.93217103482365	3.91535032171159e-08
ENSG00000179163	FUCA1	1.9315855464602	1.83915955505485e-152
ENSG00000243926	TIPARP-AS1	1.93059271123344	1.47494781950168e-07
ENSG00000273026	N.A.	1.92979649942968	0.00312062945119293
ENSG00000204970	PCDHA1	1.92819866860384	2.55574163552409e-08
ENSG00000272606	N.A.	1.92676970681729	0.000707640027923901
ENSG00000038532	CLEC16A	1.92656674087276	4.37174629058786e-204
ENSG00000087253	LPCAT2	1.92421473450072	2.49859816639144e-52
ENSG00000137501	SYTL2	1.92214054850963	3.3071465857006e-49
ENSG00000161544	CYGB	1.92193445608335	0.00107445455862543
ENSG00000179862	CITED4	1.92094181191447	5.34823879023621e-26
ENSG00000136960	ENPP2	1.920101724408	0.000365563155003572
ENSG00000272993	N.A.	1.91991354440203	1.4774038460004e-09
ENSG00000050405	LIMA1	1.91957540442122	0
ENSG00000167106	FAM102A	1.9188459629624	0
ENSG00000163704	PRRT3	1.91853424354681	3.07640773227665e-20
ENSG00000126458	RRAS	1.91594745375649	3.72261676422673e-176
ENSG00000135617	PRADC1	1.915155886851	3.78670181830735e-99
ENSG00000105289	TJP3	1.91446600679615	4.54511650867019e-99
ENSG00000245532	NEAT1	1.91416383145138	0
ENSG00000260563	N.A.	1.91309859757917	3.00104099625177e-19
ENSG00000072195	SPEG	1.911004170773	3.86519679888568e-11
ENSG00000163328	GPR155	1.91045016168113	1.16539165305864e-18
ENSG00000105767	CADM4	1.90915323111473	4.73657599740279e-113
ENSG00000272791	N.A.	1.90725800424735	0.00863615790989532
ENSG00000229021	N.A.	1.90541055145506	2.31735174432978e-05
ENSG00000144730	IL17RD	1.90538240070706	6.3610630788659e-42
ENSG00000136002	ARHGEF4	1.9046166208228	1.70174923444527e-19
ENSG00000138400	MDH1B	1.90129777237026	7.90329910455747e-07
ENSG00000121064	SCPEP1	1.90107892876974	5.32860013039563e-287
ENSG00000072071	ADGRL1	1.90099089402012	3.81863352545941e-99
ENSG00000213654	GPSM3	1.90054270643728	0.00013064329739077
ENSG00000257086	N.A.	1.8972403773529	1.28459845279361e-07
ENSG00000274220	N.A.	1.89671368984899	0.000153869651732099
ENSG00000138617	PARP16	1.896639114055	8.66087529731272e-108
ENSG00000110944	IL23A	1.89631123392172	0.000491367056531046
ENSG00000167549	CORO6	1.89545534013106	1.55701124101999e-48
ENSG00000160999	SH2B2	1.89544408503068	5.3626282849547e-13
ENSG00000158445	KCNB1	1.89116260986092	1.96762002001874e-07
ENSG00000259877	N.A.	1.88812098827019	1.05382516001349e-17

ENSG00000178385	PLEKHM3	1.88792736872157	1.51256428036889e-23
ENSG00000221990	EXOC3-AS1	1.88686744393938	1.25678696042474e-19
ENSG00000010318	PHF7	1.88666255751067	1.03144549790581e-15
ENSG00000198929	NOS1AP	1.87945352603889	9.66642958823677e-08
ENSG00000105991	HOXA1	1.87938426006165	4.21839761074795e-42
ENSG00000150907	FOXO1	1.87788749755936	3.18766339667203e-52
ENSG00000228623	ZNF883	1.87704386211283	2.52983403049864e-14
ENSG00000182809	CRIP2	1.87697886052741	3.35118483846829e-102
ENSG00000169239	CA5B	1.8767864823425	8.33916684071968e-44
ENSG00000095066	HOOK2	1.87605513076372	7.04388372082847e-43
ENSG00000171219	CDC42BPG	1.87558598628278	1.00523060787769e-14
ENSG00000136114	THSD1	1.87519620045946	8.89276150746246e-23
ENSG00000078804	TP53INP2	1.87379105979656	1.48963327892735e-88
ENSG00000168811	IL12A	1.87215007250285	0.000928990240288339
ENSG00000079156	OSBPL6	1.87116387276455	9.28837445236041e-20
ENSG00000267666	N.A.	1.87055529770428	0.000624006351548249
ENSG00000268895	A1BG-AS1	1.8702066504336	2.07034755031937e-22
ENSG00000196569	LAMA2	1.86923038216857	0.000247472960475471
ENSG00000105607	GCDH	1.86902360653185	7.54607993636407e-84
ENSG00000129521	EGLN3	1.86757156331878	7.47861025041808e-26
ENSG00000060762	MPC1	1.86659508646966	4.66264500721496e-43
ENSG00000178947	SMIM10L2A	1.86641271546234	6.99897612393995e-13
ENSG00000073605	GSDMB	1.86532814007889	8.60742420478473e-55
ENSG00000142347	MYO1F	1.86410719449148	0.000166460963421963
ENSG00000175874	CREG2	1.86404637103498	1.1460658066553e-05
ENSG00000245281	N.A.	1.86394826259921	0.00865520795085216
ENSG00000262587	N.A.	1.86301367869905	0.00013224817755032
ENSG00000280187	N.A.	1.86157562707149	1.46961327646021e-39
ENSG00000100968	NFATC4	1.86137424408497	0.000228025280041874
ENSG00000267348	N.A.	1.86018490385254	0.00998269397180177
ENSG00000164535	DAGLB	1.85936887070854	5.60265364732642e-115
ENSG00000181350	LRRC75A	1.85759155734319	3.20714087808129e-08
ENSG00000119938	PPP1R3C	1.85657950960711	1.11699278097316e-68
ENSG00000089101	CFAP61	1.85636512954337	0.00217651081168408
ENSG00000005020	SKAP2	1.85493141819809	1.76831735561176e-245
ENSG00000144061	NPHP1	1.85425993221385	6.34683821287897e-41
ENSG00000030419	IKZF2	1.85416997338523	2.49132680925678e-09
ENSG00000235052	N.A.	1.85254112882631	0.000772713186447027
ENSG00000140854	KATNB1	1.85244620725548	1.32870855046296e-181
ENSG00000260912	N.A.	1.85115127826605	1.9270489365878e-11
ENSG00000186480	INSIG1	1.85037193243065	0
ENSG00000267060	PTGES3L	1.84987392746518	6.05197296986361e-05
ENSG00000130766	SESN2	1.84985918278833	3.22323589577462e-60
ENSG00000114353	GNAI2	1.84867761529613	7.8273088559869e-210
ENSG00000028277	POU2F2	1.84845935686971	4.88322876570729e-09
ENSG00000229931	N.A.	1.84658714021185	0.00450824104617043
ENSG00000271869	N.A.	1.84567823492794	1.00686832325383e-06
ENSG00000256151	N.A.	1.84498311245719	1.37793238477087e-16
ENSG00000232586	KIAA1614-AS1	1.84276733601533	0.00382867869023779

ENSG00000159720	ATP6V0D1	1.84104118728631	5.32152360250305e-234
ENSG00000118898	PPL	1.84054687010033	2.39763025163834e-102
ENSG00000165097	KDM1B	1.83969976853354	7.63893903411351e-119
ENSG00000151136	BTBD11	1.8379637980038	9.63920714871317e-137
ENSG00000100292	HMOX1	1.83759335413054	0
ENSG00000197903	HIST1H2BK	1.83718393794317	5.33099065691404e-61
ENSG00000087301	TXNDC16	1.83690090304102	7.73604961266994e-62
ENSG00000089163	SIRT4	1.8364613362718	5.49360762556316e-16
ENSG00000177879	AP3S1	1.83563676792559	2.40186676238400e-300
ENSG00000117477	CCDC181	1.83386360479786	0.000481827449954775
ENSG00000169891	REPS2	1.83089772524866	6.79180953539791e-65
ENSG00000170396	ZNF804A	1.83055356592088	0.00260040507142784
ENSG00000023445	BIRC3	1.82916910741866	4.60526184813648e-216
ENSG00000159348	CYB5R1	1.82802744941809	6.81074899779412e-137
ENSG00000150967	ABCB9	1.82797565059079	5.40090680496797e-64
ENSG00000114796	KLHL24	1.82685923065578	1.10231550336835e-74
ENSG00000108852	MPP2	1.82569728932008	8.14898907230059e-70
ENSG00000123364	HOXC13	1.82416294406129	1.78993011101534e-36
ENSG00000167468	GPX4	1.82342558715847	1.51780295357262e-243
ENSG00000108106	UBE2S	1.82315717960093	2.54899547086514e-301
ENSG00000135407	AVIL	1.8231071832202	1.01342332468464e-10
ENSG00000157890	MEGF11	1.82265141073043	0.000428232601468464
ENSG00000068615	REEP1	1.82250344568901	2.28576364265154e-32
ENSG00000075618	FSCN1	1.82203066141246	0
ENSG00000214900	LINC01588	1.82157516338015	4.19742685690081e-21
ENSG00000226026	N.A.	1.82055348432571	0.00730513533312241
ENSG00000173930	SLCO4C1	1.81811821240034	7.43205047340235e-15
ENSG00000239213	NCK1-AS1	1.81708001468083	1.53881114496079e-10
ENSG00000115266	APC2	1.81627304633253	2.19231531798094e-07
ENSG00000267757	EML2-AS1	1.81572240865717	1.70482301292077e-10
ENSG00000160716	CHRNB2	1.8149714174376	0.000125852454325933
ENSG00000138769	CDKL2	1.81480817564451	8.76782370467829e-16
ENSG00000103489	XYLT1	1.81410204294404	0.00157214731914042
ENSG00000277232	GTSE1-AS1	1.81408669336143	0.00378744246121505
ENSG00000279352	N.A.	1.81286850142134	0.00115236585873021
ENSG00000155252	PI4K2A	1.81266669138213	5.65890562822777e-158
ENSG00000163346	PBXIP1	1.80961156100593	5.08869932130412e-209
ENSG00000176909	MAMSTR	1.80960799371368	0.000479478999740091
ENSG00000125388	GRK4	1.80861015347755	2.0779658129548e-08
ENSG00000007047	MARK4	1.80839983460796	2.50374686585871e-74
ENSG00000177359	N.A.	1.80787846426701	6.9817733234994e-05
ENSG00000259330	INAFM2	1.8074728182472	1.20906945729859e-08
ENSG00000121104	FAM117A	1.80710856702488	6.92568807638663e-47
ENSG00000067715	SYT1	1.80590752185374	3.63983759174086e-201
ENSG00000143367	TUFT1	1.80569289279586	1.54882431478229e-160
ENSG00000105255	FSD1	1.80529550709541	2.93498394175677e-21
ENSG00000196353	CPNE4	1.80478902195848	0.000742291358202713
ENSG00000256073	URB1-AS1	1.80437275739047	2.40790939584146e-26
ENSG00000084628	NKAIN1	1.80314355486714	0.00128048875644526

ENSG00000261572	N.A.	1.8023315232391	3.25673914987891e-08
ENSG00000185519	FAM131C	1.80191723340717	1.65083592320388e-16
ENSG00000101782	RIOK3	1.80189021838396	3.01051634994968e-183
ENSG00000135482	ZC3H10	1.80157037644447	5.25713930881583e-83
ENSG00000141384	TAF4B	1.80120877904149	2.12912443745827e-35
ENSG00000267632	N.A.	1.80050081002591	4.02522317191882e-06
ENSG00000273783	N.A.	1.8004633750907	1.10628459633617e-05
ENSG00000077150	NFKB2	1.79884396714778	1.27467286256108e-111
ENSG00000141665	FBXO15	1.798747083433	8.79756724832964e-09
ENSG00000169738	DCXR	1.79863955744216	2.25314076190527e-152
ENSG00000101670	LIPG	1.79810202148815	5.43716448643495e-29
ENSG00000186451	SPATA12	1.7953012629164	0.00271718355539959
ENSG00000274104	N.A.	1.79325726198426	0.00751047590376161
ENSG00000181885	CLDN7	1.79291107548539	1.12456458273903e-06
ENSG00000172361	CFAP53	1.79120744048626	1.08907833422274e-05
ENSG00000234289	H2BFS	1.79103394156004	6.63757065168652e-15
ENSG00000119711	ALDH6A1	1.7906055433751	6.01831557551567e-170
ENSG00000105290	APLP1	1.79010657511315	7.69796488272811e-246
ENSG00000257261	N.A.	1.78834590394389	5.08247943029436e-10
ENSG00000228436	N.A.	1.78800444358582	0.00851947876474946
ENSG00000237036	ZEB1-AS1	1.78780555253804	1.02373948001015e-23
ENSG00000008277	ADAM22	1.78770178337967	3.54324519174385e-16
ENSG00000204520	MICA	1.78756768819603	1.60401787363994e-55
ENSG00000212864	RNF208	1.786773935782	0.00180494332613708
ENSG00000107798	LIPA	1.78385965618806	0
ENSG00000143127	ITGA10	1.7820341062254	7.31506147239516e-07
ENSG00000167693	NXN	1.7819857576092	1.81895274393549e-123
ENSG00000261189	N.A.	1.78066573418064	3.59747568209225e-07
ENSG00000236682	N.A.	1.77972615940185	2.24850593140203e-06
ENSG00000103005	USB1	1.77636027121244	0
ENSG00000100280	AP1B1	1.77551477907074	5.8681177061873e-298
ENSG00000265401	N.A.	1.77525966588711	0.0073095679142923
ENSG00000166016	ABTB2	1.77460435024562	2.88398494009613e-147
ENSG00000050165	DKK3	1.77216831046054	3.84153006583715e-89
ENSG00000255224	N.A.	1.77113909117079	2.89929264100766e-07
ENSG00000246366	LACTB2-AS1	1.76536240622423	0.00306186033325481
ENSG00000140941	MAP1LC3B	1.76440956223431	4.91195731741984e-279
ENSG00000150773	PIH1D2	1.76352917808499	6.73318208972646e-07
ENSG00000167657	DAPK3	1.76316165488945	7.75108091872252e-114
ENSG00000151320	AKAP6	1.76139939707055	3.87683118724569e-25
ENSG00000101928	MOSPD1	1.7597001052487	5.39968805216196e-105
ENSG00000178397	FAM220A	1.75864856910771	1.66828644393849e-124
ENSG00000172216	CEBPB	1.75811938374134	5.89305733528615e-123
ENSG00000268621	N.A.	1.75656028579866	1.77294150224828e-65
ENSG00000224189	HAGLR	1.75513469629902	3.31278271156676e-06
ENSG00000185838	GNB1L	1.75507527141748	2.15205959106308e-30
ENSG00000155744	FAM126B	1.75417790815222	3.0044559191456e-88
ENSG00000198719	DLL1	1.75356835946229	0.0036342833567997
ENSG00000250486	FAM218A	1.75265119765473	4.90704722187087e-05

ENSG00000270605	N.A.	1.75177689475576	1.14974809363773e-06
ENSG00000013588	GPRC5A	1.75161205469268	0
ENSG00000269176	N.A.	1.75006927010095	8.49736502763649e-07
ENSG00000272654	N.A.	1.7488806481051	0.000252051948389345
ENSG00000139266	MARCH9	1.74866301742594	8.75722819267668e-88
ENSG00000110169	HPX	1.74770911261718	1.59022540939657e-05
ENSG00000280002	N.A.	1.74683994038968	1.11725274471572e-16
ENSG00000179134	SAMD4B	1.74545192252665	5.39858762110884e-301
ENSG00000266976	N.A.	1.74543501393755	0.00141288295539461
ENSG00000234678	N.A.	1.74375818787218	6.36088779281076e-48
ENSG00000270504	N.A.	1.74358773834434	7.83587557474219e-07
ENSG00000164663	USP49	1.74321416138583	3.08761553703894e-44
ENSG00000055813	CCDC85A	1.74288810852202	0.00686599278850116
ENSG00000279539	N.A.	1.74232869824969	0.00089333960773983
ENSG00000188051	TMEM221	1.74102799448297	0.000264412136261872
ENSG00000128274	A4GALT	1.74050985041891	3.70312443170443e-62
ENSG00000197355	UAP1L1	1.73954880367583	5.2706206704905e-58
ENSG00000183196	CHST6	1.73807324820465	9.86586681745629e-06
ENSG00000258457	N.A.	1.73409335605277	1.22337144904737e-08
ENSG00000272369	N.A.	1.73396258773199	7.26274623536266e-05
ENSG00000187186	N.A.	1.73361521023279	0.00109775446266412
ENSG00000100554	ATP6V1D	1.72810542229604	4.14444277658876e-268
ENSG00000101017	CD40	1.72343878814892	8.72207438942118e-18
ENSG00000279048	N.A.	1.72126576476869	8.5213151998361e-05
ENSG00000122515	ZMIZ2	1.72092763390264	1.60781853265816e-172
ENSG00000267750	RUNDC3A-AS1	1.71943965114096	6.86801837311925e-08
ENSG00000185437	SH3BGR	1.71863464434679	1.12944423908663e-05
ENSG00000171159	C9orf16	1.71812028040876	7.11282074511318e-82
ENSG00000197568	HHLA3	1.71642260547313	7.7492192388703e-17
ENSG00000143434	SEMA6C	1.71537593285876	5.27891063269684e-18
ENSG00000262155	N.A.	1.71495973046598	0.00564230634039322
ENSG00000168939	SPRY3	1.71363402729648	7.48365699897294e-07
ENSG00000120129	DUSP1	1.71351472857158	8.03174199781828e-232
ENSG00000205464	ATP6AP1L	1.7107451511948	0.000636969993281396
ENSG00000251129	N.A.	1.70844269781306	9.78611304722166e-32
ENSG00000079337	RAPGEF3	1.70787917441377	6.44227423978586e-07
ENSG00000175662	TOM1L2	1.70646015451252	7.47461415315106e-214
ENSG00000023608	SNAPC1	1.70615894293154	2.50928137549098e-63
ENSG00000167550	RHEBL1	1.70523972632053	7.10721367944978e-15
ENSG00000273311	DGCR11	1.70486974495857	2.67987487737282e-07
ENSG00000009844	VTA1	1.70418736537728	2.49315943156757e-274
ENSG00000198673	FAM19A2	1.7036646390781	0.000219580829611365
ENSG00000171303	KCNK3	1.70317740762176	3.17541596915517e-07
ENSG00000145990	GFOD1	1.70296468853252	1.26873407715429e-12
ENSG00000205791	LOH12CR2	1.70290062160814	0.0039991166521229
ENSG00000133083	DCLK1	1.70153848579891	2.294805158911e-09
ENSG00000072954	TMEM38A	1.70141662652419	0.000297274287507036
ENSG00000119771	KLHL29	1.7011389575898	5.27802367324433e-23
ENSG00000245060	LINC00847	1.70013825037836	5.48054488257658e-49

ENSG00000166246	C16orf71	1.69950310631539	1.07410223276608e-06
ENSG00000152503	TRIM36	1.69930627979159	4.01930960315122e-23
ENSG00000070601	FRMPD1	1.69887593448222	0.00745948677345765
ENSG00000260081	N.A.	1.69813034860274	0.00566754623076521
ENSG00000160445	ZER1	1.69754951578686	1.33961884299197e-176
ENSG00000114631	PODXL2	1.69616776038827	2.54424958978296e-53
ENSG00000174804	FZD4	1.69568871982689	6.77321196698766e-44
ENSG00000118997	DNAH7	1.69474242756264	1.03780042528338e-20
ENSG00000157734	SNX22	1.69407937924106	4.50167287005083e-10
ENSG00000101236	RNF24	1.69346799368617	5.7148549123026e-123
ENSG00000170458	CD14	1.69184695466855	1.30918504677539e-05
ENSG00000116667	C1orf21	1.69130949531898	0.000303456440554894
ENSG00000205810	KLRC3	1.68952078292035	0.00919925646258248
ENSG00000151640	DPYSL4	1.68892433031673	7.30777865451961e-34
ENSG00000134668	SPOCD1	1.68854254432445	2.92625504713723e-09
ENSG00000273001	N.A.	1.68780365732303	0.00119441340542356
ENSG00000272899	N.A.	1.68660672208066	3.36327211959272e-06
ENSG00000148832	PAOX	1.68641451375007	9.68153052834359e-10
ENSG00000004139	SARM1	1.68580851477983	2.1659319166603e-38
ENSG00000145335	SNCA	1.68578183284873	1.6542176446753e-74
ENSG00000146826	C7orf43	1.68564006831278	6.41865933072974e-60
ENSG00000030582	GRN	1.68500563718388	6.75859223524805e-304
ENSG00000280287	N.A.	1.68403322263319	3.1017801181256e-09
ENSG00000168461	RAB31	1.68351338474909	5.93627290959883e-145
ENSG00000180425	C11orf71	1.68299911092839	7.24913503673834e-16
ENSG00000246090	N.A.	1.68278909106565	0.00948272766025464
ENSG00000178531	CTXN1	1.68251414009606	1.19322741475329e-17
ENSG00000101347	SAMHD1	1.68243507764881	1.88250834163181e-164
ENSG00000151014	CCRN4L	1.67916641457158	3.93742244785516e-41
ENSG00000131459	GFPT2	1.67783401921306	4.64660742165480e-114
ENSG00000166046	TCP11L2	1.67630744529359	5.73162087435992e-33
ENSG00000108523	RNF167	1.67583707722923	1.14146614766644e-253
ENSG00000224063	N.A.	1.67404542671201	0.000778438263223622
ENSG00000100784	RPS6KA5	1.67381938453103	5.16763782409851e-34
ENSG00000137460	FHDC1	1.67326398720021	7.05399882112423e-19
ENSG00000175105	ZNF654	1.67262463263826	3.679862464266e-52
ENSG00000090674	MCOLN1	1.67195282077966	8.40057176977925e-47
ENSG00000137819	PAQR5	1.67025607817691	7.01541592462519e-75
ENSG00000135090	TAOK3	1.66932651359929	4.66425295534223e-205
ENSG00000221916	C19orf73	1.66832253096838	6.16950761392247e-06
ENSG00000229368	N.A.	1.66659131341687	5.34013960252156e-05
ENSG00000184205	TSPYL2	1.66197702674087	5.30909328105356e-111
ENSG00000197405	C5AR1	1.66169657223238	4.51234967196486e-13
ENSG00000171798	KNDC1	1.66078521769432	7.49075315717093e-08
ENSG00000138650	PCDH10	1.66038558366572	3.68606672108599e-44
ENSG00000278266	N.A.	1.65894178346833	4.37933113650343e-08
ENSG00000186301	MST1P2	1.65606625782497	0.00148460592527052
ENSG00000117461	PIK3R3	1.65422295706001	2.7369668569102e-68
ENSG00000248323	LUCAT1	1.65248591194306	3.40236633921142e-29

ENSG00000160233	LRRC3	1.65216601915456	2.13759665715105e-15
ENSG00000260329	N.A.	1.64850750226788	2.27321820267151e-10
ENSG00000177692	DNAJC28	1.64823616047882	0.000103156858402179
ENSG00000269887	N.A.	1.64817799406618	0.00160556397681777
ENSG00000254615	N.A.	1.64807391204914	9.46183619219278e-05
ENSG00000167912	N.A.	1.64635234661199	0.00056868735713187
ENSG00000203780	FANK1	1.64515399365899	2.82668053029219e-08
ENSG00000171310	CHST11	1.64404160811273	1.35683793035898e-109
ENSG00000128203	ASPHD2	1.64363406812628	1.22666554671486e-11
ENSG00000203930	LINC00632	1.64278165142438	2.92277707894147e-07
ENSG00000145506	NKD2	1.64231013250811	1.15380568878075e-15
ENSG00000165355	FBXO33	1.64159964382698	2.62658351779073e-87
ENSG00000282885	N.A.	1.63942551325918	0.000307687752773019
ENSG00000197182	MIRLET7BHG	1.63814828474389	5.19446743515517e-15
ENSG00000101844	ATG4A	1.63700871824483	1.15233692623472e-89
ENSG00000089060	SLC8B1	1.63686817528057	1.15564807578581e-84
ENSG00000025423	HSD17B6	1.63575595195124	0.000199233227928602
ENSG00000178662	CSRNP3	1.63310185762115	1.48518177750932e-12
ENSG00000148677	ANKRD1	1.63088824358957	6.979051333192e-09
ENSG00000188316	ENO4	1.62794186675467	0.000159558538222519
ENSG00000036448	MYOM2	1.62738083830637	1.53518163628446e-09
ENSG00000176124	DLEU1	1.62295752143826	1.1230765193114e-15
ENSG00000233237	LINC00472	1.62270836813813	7.65665767463412e-08
ENSG00000182771	GRID1	1.62250032273306	0.00133476477164398
ENSG00000145569	FAM105A	1.62209967386653	6.09473604947546e-52
ENSG00000260766	N.A.	1.62170116230521	3.14537318328603e-15
ENSG00000149212	SESN3	1.62114110581854	6.40290279335842e-78
ENSG00000089199	CHGB	1.61709221972363	1.06999798666778e-52
ENSG00000187800	PEAR1	1.61700631016574	0.00582045463876848
ENSG00000158373	HIST1H2BD	1.61676861033205	1.18728944012522e-47
ENSG00000227354	RBM26-AS1	1.61658939757374	0.000584842034516654
ENSG00000151715	TMEM45B	1.61654815645456	3.78800094034938e-08
ENSG00000227502	LINC01268	1.6160886573749	0.000512381496527939
ENSG00000135740	SLC9A5	1.61568760298031	2.31730348290439e-08
ENSG00000074370	ATP2A3	1.61563492382781	2.8253465292606e-61
ENSG00000188766	SPRED3	1.61541111865419	3.53587859576638e-13
ENSG00000171174	RBKS	1.6146967912434	2.56390621076457e-19
ENSG00000261553	N.A.	1.61384960698501	0.00854059179156302
ENSG00000113248	PCDHB15	1.61348545461569	0.00151084488465183
ENSG00000089723	OTUB2	1.61292249799951	2.91529579746032e-16
ENSG00000131759	RARA	1.61060462977921	2.18385473717566e-90
ENSG00000129353	SLC44A2	1.60896838206282	2.070198275524e-104
ENSG00000174007	CEP19	1.60717391822287	4.01665658384348e-24
ENSG00000131480	AOC2	1.60541285889412	4.84268631686998e-15
ENSG00000179403	VWA1	1.60260780888543	2.8452668780274e-79
ENSG00000196358	NTNG2	1.60136725796253	7.37190000283378e-08
ENSG00000173559	NABP1	1.5991528487664	4.80514635355239e-49
ENSG00000015285	WAS	1.59837146001116	0.00671214915030487
ENSG00000196428	TSC22D2	1.59800208244581	5.76760438955332e-100

ENSG00000124098	FAM210B	1.59723728336893	2.38030101708083e-82
ENSG00000151023	ENKUR	1.59656577197738	0.00308660592742286
ENSG00000147437	GNRH1	1.59488430824003	9.98887132899981e-08
ENSG00000130201	EXOC3L2	1.59359827577691	0.00461582315958105
ENSG00000167703	SLC43A2	1.59330600801797	9.82883647112804e-55
ENSG00000144583	MARCH4	1.59294789018379	1.71275222060748e-07
ENSG00000136770	DNAJC1	1.59259058659929	1.99859269269626e-130
ENSG00000149328	GLB1L2	1.59182948652951	3.42133700547191e-28
ENSG00000135049	AGTPBP1	1.59105421789857	4.51130607962052e-120
ENSG00000102904	TSNAXIP1	1.59058735527884	9.18668914194053e-06
ENSG00000196961	AP2A1	1.58954431467285	0
ENSG00000111684	LPCAT3	1.58638415974986	0
ENSG00000272525	N.A.	1.58597678344457	0.000108285803359899
ENSG00000267365	KCNJ2-AS1	1.58534050675298	0.00166151605561342
ENSG00000125462	C1orf61	1.58462075467783	1.93217998687898e-07
ENSG00000227540	N.A.	1.58348473611589	5.82340630909424e-08
ENSG00000232645	LINC01431	1.58313578087824	1.51683278007409e-05
ENSG00000171962	LRRC48	1.58292221625973	3.19755151348392e-12
ENSG00000121753	ADGRB2	1.58284546914344	1.30901065872363e-52
ENSG00000063438	AHRR	1.58164976271218	6.56042354896417e-18
ENSG00000134955	SLC37A2	1.58088183536953	0.00116506408013017
ENSG00000174567	GOLT1A	1.5800908524889	3.61409806460068e-17
ENSG00000197935	ZNF311	1.57904886800409	3.91905621746082e-06
ENSG00000164938	TP53INP1	1.5788559033767	8.97420892116545e-81
ENSG00000127948	POR	1.57854782202383	0
ENSG00000181016	LSMEM1	1.57746509483062	1.09026303677714e-06
ENSG00000109066	TMEM104	1.57666644840513	7.77168176753714e-128
ENSG00000251562	MALAT1	1.57444879204426	1.20432513029292e-236
ENSG00000145687	SSBP2	1.57369740090029	8.82699176247243e-75
ENSG00000021645	NRXN3	1.57275245173443	2.70680738334136e-05
ENSG00000180354	MTURN	1.57266840856394	3.74523131580142e-18
ENSG00000159423	ALDH4A1	1.56867920606239	7.95349801491542e-41
ENSG00000169991	IFFO2	1.56547052924516	1.89749436965941e-35
ENSG00000176658	MYO1D	1.56541718525238	1.56327430215814e-65
ENSG00000279821	N.A.	1.56460654420404	0.00162234011583543
ENSG00000183762	KREMEN1	1.56459620842503	7.82963881889826e-53
ENSG00000164543	STK17A	1.56440972088005	1.30212170972264e-83
ENSG00000107864	CPEB3	1.56394508981175	1.88047240224214e-10
ENSG00000164330	EBF1	1.5624638107211	8.49608276866263e-05
ENSG00000113594	LIFR	1.56046418109656	1.3601078766672e-55
ENSG00000225206	MIR137HG	1.55792990968504	2.68306739482308e-25
ENSG00000165029	ABCA1	1.55754867055788	5.81543234754694e-21
ENSG00000265458	N.A.	1.55686919645593	0.00064799885131346
ENSG00000182197	EXT1	1.5568123943512	8.392128482013e-146
ENSG00000100307	CBX7	1.55622022284747	1.43555208670021e-22
ENSG00000117016	RIMS3	1.55540630358464	2.43929056200966e-32
ENSG00000110047	EHD1	1.55400349491989	1.42422167020839e-177
ENSG00000182050	MGAT4C	1.5537879393287	0.00363862076513513
ENSG00000181035	SLC25A42	1.55349958742405	6.54741020983769e-19

ENSG00000225265	TAF1A-AS1	1.54924984762693	1.23006272764757e-05
ENSG00000117410	ATP6V0B	1.54924283028672	4.38503767584747e-142
ENSG00000188641	DPYD	1.54916520002231	3.39031510298885e-54
ENSG00000255121	N.A.	1.54877274132682	8.99262155466903e-10
ENSG00000140511	HAPLN3	1.54756657350592	2.7037580998817e-19
ENSG00000196396	PTPN1	1.54529269666359	3.32829737917706e-214
ENSG00000169992	NLGN2	1.54509167012068	4.04992384078507e-138
ENSG00000049759	NEDD4L	1.544363297528	3.59577892655572e-224
ENSG00000277142	LINC00235	1.54436007013809	0.000253382614161482
ENSG00000279865	N.A.	1.54420655909419	0.000231804734976595
ENSG00000120327	PCDHB14	1.54401299642538	0.000461974957636418
ENSG00000137070	IL11RA	1.54359300633573	3.91662038855153e-25
ENSG00000276073	N.A.	1.54282127486524	5.09873002606993e-05
ENSG00000004799	PDK4	1.54274336457166	1.78477116518802e-167
ENSG00000236496	GPS2P1	1.54257842454305	5.7880897452316e-11
ENSG00000174516	PELI3	1.54201709929014	5.41664365725274e-31
ENSG00000204118	NAP1L6	1.5412399734273	0.00279470512887193
ENSG00000165046	LETM2	1.54118071554121	2.99100592214285e-11
ENSG00000273486	N.A.	1.53883146718591	0.00747056171362242
ENSG00000261534	N.A.	1.53878384317682	0.0015845372374003
ENSG00000099785	MARCH2	1.53863277247667	4.11592182740074e-59
ENSG00000232098	N.A.	1.53773124129246	2.94505197662123e-28
ENSG00000173947	PIFO	1.53745498703934	8.18353775367867e-05
ENSG00000067836	ROGDI	1.53668395394435	2.37620993775666e-53
ENSG00000102760	RGCC	1.53623693454981	1.22323247948662e-07
ENSG00000144891	AGTR1	1.53424779339838	6.89469289913877e-06
ENSG00000177570	SAMD12	1.53420558999293	0.00147086315520543
ENSG00000158423	RIBC1	1.53283198553553	3.20970738563001e-08
ENSG00000198598	MMP17	1.53238486221795	1.21744044265479e-31
ENSG00000168610	STAT3	1.53168717755592	0
ENSG00000228175	GEMIN8P4	1.53165842623507	5.24356078506353e-06
ENSG00000214548	MEG3	1.53137624403312	4.10821083046209e-80
ENSG00000268049	N.A.	1.53075770679298	0.00101387809293738
ENSG00000267309	N.A.	1.53064088303665	2.17828940103644e-05
ENSG00000090539	CHRD	1.52984174667418	0.00607655855326445
ENSG00000130821	SLC6A8	1.52865379454669	8.15395483289628e-41
ENSG00000254858	MPV17L2	1.52848708146159	3.10353397266314e-51
ENSG00000261342	N.A.	1.52756706917644	1.78230548697659e-05
ENSG00000121413	ZSCAN18	1.52739491215754	1.47222004049813e-05
ENSG00000224424	PRKAR2A-AS1	1.52726410175779	0.000115887701350837
ENSG00000278709	NKILA	1.5267761177428	1.20287411114064e-19
ENSG00000139438	FAM222A	1.52584887692041	6.02602598389955e-25
ENSG00000100979	PLTP	1.52569357086101	6.92128937995768e-10
ENSG00000128965	CHAC1	1.52543734597402	5.88343471367539e-27
ENSG00000255046	N.A.	1.52409480272868	0.000325792500115902
ENSG00000255836	N.A.	1.52339466301032	2.24786351491138e-21
ENSG00000166839	ANKDD1A	1.52247312701939	9.16558786754981e-06
ENSG00000105717	PBX4	1.52241901573395	0.0001836291316196
ENSG00000118523	CTGF	1.52111009388401	1.09530499915767e-168

ENSG00000267100	ILF3-AS1	1.5210024865225	5.19607700841521e-30
ENSG00000000419	DPM1	1.52099888649917	1.91541477649206e-170
ENSG00000090020	SLC9A1	1.52074384408356	1.79400062519019e-106
ENSG00000085433	WDR47	1.52065314403794	1.13328096606618e-60
ENSG00000137821	LRRC49	1.51998925958502	2.23525698789049e-66
ENSG00000211451	GNRHR2	1.51886591616143	2.22718635484558e-07
ENSG00000047249	ATP6V1H	1.51803405166613	2.58023433434403e-136
ENSG00000123358	NR4A1	1.51722198104926	2.91456187008419e-158
ENSG00000102096	PIM2	1.51675247550926	2.71834092475174e-63
ENSG00000272189	N.A.	1.51624834466998	0.000179763158373443
ENSG00000276023	DUSP14	1.51563958003697	2.00390458541142e-98
ENSG00000238197	PAXBP1-AS1	1.51474422534442	1.88849060511928e-05
ENSG00000142546	NOSIP	1.51442769558545	9.47546810191338e-144
ENSG00000233621	LINC01137	1.51362339531657	9.12482482787922e-18
ENSG00000023287	RB1CC1	1.51351544255086	6.8175614321722e-154
ENSG00000180263	FGD6	1.51233333796181	9.23786644723722e-261
ENSG00000229729	N.A.	1.51223148513205	8.62224508998766e-13
ENSG00000108828	VAT1	1.5120689803198	1.31131693534603e-237
ENSG00000146112	PPP1R18	1.50856446003655	1.81943753302006e-68
ENSG00000135363	LMO2	1.50725580551967	0.00895465747188541
ENSG00000111012	CYP27B1	1.50552272128674	2.75212324019424e-06
ENSG00000148154	UGCG	1.50502746976327	7.53379820069151e-113
ENSG00000215196	N.A.	1.50413632488541	0.00218466854552616
ENSG00000168067	MAP4K2	1.50405233747104	9.99479493587209e-49
ENSG00000122507	BBS9	1.50129364505963	6.22155652779269e-39
ENSG00000260526	N.A.	1.50126750843356	0.00306864743906588
ENSG00000101445	PPP1R16B	1.4985853282472	9.49023450446538e-12
ENSG00000111348	ARHGDIB	1.49793721975036	1.49974841822752e-06
ENSG00000105639	JAK3	1.49789837369129	3.21844923677085e-12
ENSG00000152763	WDR78	1.495882903978	3.48460618414859e-06
ENSG00000068976	PYGM	1.49513944972384	0.0012281614617074
ENSG00000198948	MFAP3L	1.49479782000914	9.48224224838328e-30
ENSG00000037280	FLT4	1.49221253945396	1.02756654656453e-17
ENSG00000274290	HIST1H2BE	1.49158601335168	4.06924354766167e-11
ENSG00000254473	N.A.	1.49080060564846	1.46382908807747e-06
ENSG00000125746	EML2	1.49079247722002	1.17375873118741e-77
ENSG00000167920	TMEM99	1.48993956380018	2.05578479563096e-57
ENSG00000079150	FKBP7	1.4892806732032	4.86910038551321e-22
ENSG00000130475	FCHO1	1.48761118716861	1.64914828193942e-37
ENSG00000198324	FAM109A	1.48309321779672	3.8895285460607e-41
ENSG00000106546	AHR	1.48267928022607	2.82807972707979e-237
ENSG00000166886	NAB2	1.48263312469131	1.05953114753812e-53
ENSG00000272079	N.A.	1.48217241281502	0.00749735233716287
ENSG00000123983	ACSL3	1.48143954991343	5.43540909870346e-295
ENSG00000276570	N.A.	1.48143299070223	0.00193767881510773
ENSG00000139737	SLAIN1	1.48058778994069	1.01152254823716e-46
ENSG00000276728	N.A.	1.47889747790244	0.00781754825766933
ENSG00000197746	PSAP	1.47737557526946	0
ENSG00000020181	ADGRA2	1.47722504862768	7.35840584218269e-29

ENSG00000243364	EFNA4	1.47715592340811	1.2819758603725e-18
ENSG00000116954	RRAGC	1.47672337555623	1.62653397567235e-60
ENSG00000185361	TNFAIP8L1	1.47637909890617	1.27091971133492e-39
ENSG00000173442	EHBP1L1	1.47570161540275	8.12302625557957e-152
ENSG00000279329	N.A.	1.47534478291933	4.80711500457517e-05
ENSG00000179242	CDH4	1.47527683505266	0.00114813237801352
ENSG00000103066	PLA2G15	1.47485931684121	6.99121036967534e-65
ENSG00000106665	CLIP2	1.47343073388111	3.97956070021837e-157
ENSG00000240065	PSMB9	1.47282160118563	1.53735289737581e-19
ENSG00000101955	SRPX	1.47241524033008	7.74881970286122e-12
ENSG00000105402	NAPA	1.47213113651172	4.83859999949652e-146
ENSG00000136327	NKX2-8	1.47161164066915	0.000428210405027336
ENSG00000112394	SLC16A10	1.47020833318585	8.91719248248292e-08
ENSG00000187189	TSPYL4	1.46977624895862	9.98596190055624e-47
ENSG00000126947	ARMCX1	1.46909965317566	5.06912901412747e-07
ENSG00000137802	MAPKBP1	1.46674973665143	1.72917338694065e-57
ENSG00000229539	N.A.	1.46581230836953	0.000245876598343212
ENSG00000005238	FAM214B	1.46529433961723	4.00814602133919e-48
ENSG00000022267	FHL1	1.46516488262232	1.89192044817876e-22
ENSG00000203778	FAM229B	1.46479462776853	8.85959169882835e-16
ENSG00000067221	STOML1	1.46425944063256	7.54588189845189e-34
ENSG00000143669	LYST	1.46321483785701	1.36569485359225e-54
ENSG00000092096	SLC22A17	1.46174478492424	3.22363286769649e-20
ENSG00000253878	N.A.	1.45950383243196	0.00296817061773686
ENSG00000173890	GPR160	1.45929776220766	1.30124822848314e-23
ENSG00000013306	SLC25A39	1.45916135750991	7.57842997839291e-234
ENSG00000116670	MAD2L2	1.45741823767767	2.35318129035227e-63
ENSG00000135148	TRAFD1	1.45717433699961	2.86497145076974e-145
ENSG00000147852	VLDLR	1.45618803756755	6.39252446421446e-34
ENSG00000240849	TMEM189	1.455845366088	9.04131116779022e-93
ENSG00000145536	ADAMTS16	1.45530975266879	0.000706527648355232
ENSG00000240891	PLCXD2	1.45518101302504	3.26616951356982e-13
ENSG00000167702	KIFC2	1.45494011501945	3.16896645187429e-60
ENSG00000107745	MICU1	1.45488119632184	5.16833633056534e-274
ENSG00000176933	TOB2P1	1.45465585806425	0.000627706273941731
ENSG00000215022	N.A.	1.45371562536026	0.00502418124273043
ENSG00000120885	CLU	1.45135540884755	0
ENSG00000262468	LINC01569	1.45078151382465	3.78624224443849e-06
ENSG00000265800	N.A.	1.45060419495458	0.00985446617732322
ENSG00000196155	PLEKHG4	1.45012530908884	2.29193027408417e-49
ENSG00000177432	NAP1L5	1.44905996874727	2.08224358131145e-19
ENSG00000162783	IER5	1.44803937647101	7.69234193036862e-71
ENSG00000128294	TPST2	1.44759821592069	8.91728891757616e-28
ENSG00000247121	N.A.	1.44713851537469	1.4464696979582e-07
ENSG00000204815	TTC25	1.44703945916141	8.27888988225068e-09
ENSG00000135269	TES	1.44650953033789	1.04604387291856e-187
ENSG00000132510	KDM6B	1.4463047637401	1.77294150224828e-65
ENSG00000198736	MSRB1	1.44597692836571	3.04233112935819e-108
ENSG00000130635	COL5A1	1.44588699548582	4.52580325535902e-82

ENSG00000099875	MKNK2	1.44468010751437	5.62044843324607e-136
ENSG00000101695	RNF125	1.44260884955884	0.00247670168868272
ENSG00000239857	GET4	1.44200867982611	2.33659721281024e-09
ENSG00000167552	TUBA1A	1.44120606230374	1.31969808026376e-249
ENSG00000164620	RELL2	1.44013880966924	7.13463984187443e-26
ENSG00000276517	N.A.	1.4395839131722	0.00037875884509962
ENSG00000243701	LINC00883	1.43956405669381	1.27655279342258e-16
ENSG00000132376	INPP5K	1.43863925422097	2.04777449590965e-100
ENSG00000179743	N.A.	1.43742975503886	1.76984207379031e-06
ENSG00000033627	ATP6V0A1	1.43727667726961	1.72134050835772e-190
ENSG00000152133	GPATCH11	1.43699944209033	1.77548424570301e-81
ENSG00000106733	NMRK1	1.43689451667283	5.74538173270973e-18
ENSG00000130707	ASS1	1.43606907678609	1.28545067083734e-27
ENSG00000149541	B3GAT3	1.43580853368695	6.53783373287044e-107
ENSG00000224383	PRR29	1.43397438740485	1.46410647123398e-06
ENSG00000101986	ABCD1	1.43364203666035	1.66943208904438e-17
ENSG00000145016	KIAA0226	1.43175450641148	7.61320458067974e-50
ENSG00000231663	N.A.	1.42965172769447	0.000256193413676239
ENSG00000198873	GRK5	1.429607471218	3.16554947167421e-31
ENSG00000056487	PHF21B	1.42951205785806	4.01936281434638e-05
ENSG00000163354	DCST2	1.42902474548219	0.000132095821762067
ENSG00000157483	MYO1E	1.42875321053387	0
ENSG00000270069	MIR222HG	1.42785994550915	2.52165265802482e-14
ENSG00000159733	ZFYVE28	1.42600673016367	2.63222117821239e-17
ENSG00000162104	ADCY9	1.42531874735673	2.682761962402e-91
ENSG00000145882	PCYOX1L	1.42514359726446	3.26185277366582e-37
ENSG00000206341	HLA-H	1.42383261191692	9.28163202175871e-06
ENSG00000099308	MAST3	1.42369354858867	7.84485499294262e-34
ENSG00000180875	GREM2	1.41948054034143	0.00275177247133375
ENSG00000114573	ATP6V1A	1.41917239876895	1.32130939285679e-238
ENSG00000132823	OSER1	1.4191279169202	5.65882179983482e-100
ENSG00000101203	COL20A1	1.41711358078159	0.000493252619235089
ENSG00000130005	GAMT	1.41630670593372	7.30968885065162e-31
ENSG00000073670	ADAM11	1.4162012222977	2.62576155601648e-14
ENSG00000126243	LRFN3	1.41596774888252	2.41368108041051e-60
ENSG00000276791	N.A.	1.41568056015163	3.2393988774025e-05
ENSG00000178460	MCMDC2	1.4156765191621	6.56218524274875e-05
ENSG00000034152	MAP2K3	1.41504468449936	4.89612868131552e-174
ENSG00000138834	MAPK8IP3	1.41374195850005	1.03883854959172e-100
ENSG00000129562	DAD1	1.41206474949746	4.4349058267391e-219
ENSG00000085063	CD59	1.41194523977021	0
ENSG00000198825	INPP5F	1.41182278784042	2.73458767351846e-77
ENSG00000225362	CT62	1.41146516596908	5.16035355519494e-05
ENSG00000244567	N.A.	1.41095478543311	1.93054274881042e-07
ENSG00000133874	RNF122	1.41095346724554	5.94010823411472e-15
ENSG00000037241	RPL26L1	1.41050806573401	9.45132448969037e-67
ENSG00000231806	PCAT7	1.40888312765217	5.86856540067309e-08
ENSG00000110328	GALNT18	1.40702575924088	1.83293946263268e-18
ENSG00000234498	RPL13AP20	1.40667336463272	0.000161998624400606

ENSG00000188763	FZD9	1.40661629801935	0.000103548968476475
ENSG00000153561	RMND5A	1.4064560077247	1.45460313001937e-150
ENSG00000151892	GFRA1	1.40609889122546	0.00225247059196052
ENSG00000144485	HES6	1.40519152738759	3.48133825404992e-26
ENSG00000172375	C2CD2L	1.40472222492175	1.91982244468531e-34
ENSG00000166148	AVPR1A	1.40324086631919	7.81804260870979e-06
ENSG00000160785	SLC25A44	1.40304588812093	2.48617010734663e-213
ENSG00000135835	KIAA1614	1.40257519142228	9.8349272648464e-05
ENSG00000156500	FAM122C	1.40068732345257	7.59264718394346e-09
ENSG00000071246	VASH1	1.39917708044227	1.13425889795942e-31
ENSG00000107984	DKK1	1.39821026648001	1.03243583224861e-149
ENSG00000175416	CLTB	1.39789111198641	7.58906331527361e-103
ENSG00000128016	ZFP36	1.39668825819317	1.48471540852999e-80
ENSG00000162076	FLYWCH2	1.39629867312651	1.19464366815444e-44
ENSG00000275342	N.A.	1.3958625034427	7.20804824785926e-150
ENSG00000096654	ZNF184	1.39563804259911	4.07284819743046e-23
ENSG00000008294	SPAG9	1.39450661493684	0
ENSG00000055163	CYFIP2	1.39431345105371	4.03232216948436e-140
ENSG00000104043	ATP8B4	1.39370802724377	2.67129461290927e-09
ENSG00000058668	ATP2B4	1.39345600531821	8.33671247115234e-121
ENSG00000146426	TIAM2	1.39310057222282	1.35912657487206e-14
ENSG00000129295	LRRC6	1.39286139160051	4.11166303394196e-09
ENSG00000156675	RAB11FIP1	1.39278341467374	4.0392664539264e-204
ENSG00000120149	MSX2	1.3927652954984	2.43042411592152e-31
ENSG00000161653	NAGS	1.39273270655508	0.000261789817227401
ENSG00000061656	SPAG4	1.39138699462366	4.50016718520607e-09
ENSG00000136167	LCP1	1.39116509691727	0.0049136564776675
ENSG00000181773	GPR3	1.39035648509322	0.000552798666743056
ENSG00000261324	N.A.	1.38984150455438	5.13346165707775e-10
ENSG00000175906	ARL4D	1.38962278181389	9.79540938817842e-20
ENSG00000129757	CDKN1C	1.38747965191384	0.00143004666581185
ENSG00000087903	RFX2	1.38671531611073	1.208760691413e-24
ENSG00000173846	PLK3	1.38596884500912	8.20158127547058e-17
ENSG00000138764	CCNG2	1.38485989704926	2.80305924058851e-45
ENSG00000196391	ZNF774	1.38456478735262	5.46518152511709e-09
ENSG00000196923	PDLIM7	1.38334818778694	4.89593772430084e-52
ENSG00000134996	OSTF1	1.38285185199742	6.77505238977628e-49
ENSG00000159596	TMEM69	1.38260125555154	1.32722529223076e-55
ENSG00000261455	LINC01003	1.38190420904392	2.68327940319999e-07
ENSG00000181004	BBS12	1.38183038975683	2.37682065795189e-18
ENSG00000182134	TDRKH	1.38160353179763	3.00470531132284e-97
ENSG00000196951	SCOC-AS1	1.38017744564648	1.07410223276608e-06
ENSG00000165164	CFAP47	1.3799371781415	0.00497004832918336
ENSG00000117139	KDM5B	1.37942153920745	5.42593534366873e-111
ENSG00000236810	TCEB3-AS1	1.37927733389529	0.000355199465831116
ENSG00000152465	NMT2	1.37801612001387	1.19337557267765e-51
ENSG00000139926	FRMD6	1.3764875822528	3.06701481570482e-55
ENSG00000107742	SPOCK2	1.37629107369239	7.6731594770947e-05
ENSG00000100558	PLEK2	1.37474288050826	1.65609379132752e-18

ENSG00000171345	KRT19	1.37473259883645	5.28733014850099e-152
ENSG00000164885	CDK5	1.37158939656941	1.43763100935175e-45
ENSG00000179674	ARL14	1.37155016039175	0.000487575440022397
ENSG00000167995	BEST1	1.37127203038916	6.17837464349366e-05
ENSG00000102119	EMD	1.37115139672429	5.12974957528132e-143
ENSG00000263786	N.A.	1.37021268092294	0.000587659218852618
ENSG00000188559	RALGAPA2	1.3682313824169	2.31172255088873e-110
ENSG00000103187	COTL1	1.36699175169085	1.55828533954598e-237
ENSG00000272853	N.A.	1.36657279027771	0.000593292719596904
ENSG00000103266	STUB1	1.36638054805066	4.65822183354254e-118
ENSG00000197208	SLC22A4	1.36615474358094	5.4379116678273e-06
ENSG00000236200	KDM4A-AS1	1.3659515189177	0.000858860210596263
ENSG00000117408	IPO13	1.36586686610231	8.19548911853658e-84
ENSG00000260000	N.A.	1.36571284212812	1.30507539886215e-05
ENSG00000142871	CYR61	1.36468171672596	2.85553217944305e-76
ENSG00000261716	N.A.	1.36442625116324	7.63302947795109e-16
ENSG00000124615	MOCS1	1.36428891415283	0.00151773237521578
ENSG00000153487	ING1	1.36356329974361	1.54594901469924e-36
ENSG00000232818	RPS2P32	1.36320937196853	3.90968909806202e-05
ENSG00000131584	ACAP3	1.36309313357649	3.3886075672973e-63
ENSG00000158457	TSPAN33	1.36194124033924	2.2639279697551e-31
ENSG00000247498	N.A.	1.36169437408481	3.04026612001107e-11
ENSG00000204611	ZNF616	1.36083583010009	1.46296932778405e-09
ENSG00000166311	SMPD1	1.35963844419099	5.73354741198309e-57
ENSG00000278318	ZNF229	1.35925898151637	1.50014454671583e-23
ENSG00000197860	SGTB	1.35910040564718	4.52908097292468e-59
ENSG00000149809	TM7SF2	1.35892700153253	1.75508735647834e-80
ENSG00000160691	SHC1	1.35875847881559	1.64534407272428e-214
ENSG00000177469	PTRF	1.35825483228362	0
ENSG00000115758	ODC1	1.35745507077653	9.11116059588428e-296
ENSG00000242622	N.A.	1.3572716729682	0.00240608165854919
ENSG00000136449	MYCBPAP	1.35717953400156	5.69995494985597e-08
ENSG00000197635	DPP4	1.35627886725834	0.000471397129124999
ENSG00000137947	GTF2B	1.35617000251389	1.20855884536278e-58
ENSG00000138378	STAT4	1.35597315466666	9.83196515612254e-13
ENSG00000145703	IQGAP2	1.35589083577605	2.27878562293138e-10
ENSG00000183722	LHFP	1.35561364832863	0.00193527211053602
ENSG00000124507	PACSIN1	1.35528454641259	1.66368477613507e-05
ENSG00000182612	TSPAN10	1.35152317516985	4.48507649025388e-07
ENSG00000001561	ENPP4	1.35027932093729	1.47628247822212e-13
ENSG00000186591	UBE2H	1.35011606562065	4.80307474000757e-212
ENSG00000154917	RAB6B	1.34925778769808	2.48181888676855e-20
ENSG00000261575	N.A.	1.34925529511208	0.0061527909879143
ENSG00000115977	AAK1	1.34875662502325	2.73884569372588e-97
ENSG00000161082	CELF5	1.34844992822733	0.0042843193950239
ENSG00000270959	LPP-AS2	1.34756262925894	2.45983481535877e-13
ENSG00000260589	STAM-AS1	1.34690994571454	0.00119134444033717
ENSG00000186352	ANKRD37	1.3467592722325	0.00945753211352199
ENSG00000170231	FABP6	1.34641358860029	0.00276801229385139

ENSG00000168676	KCTD19	1.34574119852911	1.74210527414047e-06
ENSG00000196110	ZNF699	1.34570530666697	1.83940269164568e-06
ENSG00000105270	CLIP3	1.34553186934068	6.46271012746667e-07
ENSG00000161513	FDXR	1.34494959523406	8.54844415836716e-74
ENSG00000106330	MOSPD3	1.34491095903051	1.46782787045724e-29
ENSG00000034713	GABARAPL2	1.34487223848787	1.58357580896487e-140
ENSG00000229980	TOB1-AS1	1.34403379401444	0.000407995227603837
ENSG00000272523	LINC01023	1.34399018011733	0.00268528646466847
ENSG00000171877	FRMD5	1.34398383627844	7.99957337620795e-16
ENSG00000165905	GYLTL1B	1.34329491857772	1.03855991337597e-09
ENSG00000105559	PLEKHA4	1.34209701710494	0.000907600830772157
ENSG00000162191	UBXN1	1.34165265365931	4.22109974731861e-129
ENSG00000197694	SPTAN1	1.34104570905132	2.14988938897584e-241
ENSG00000136854	STXBP1	1.33960090907352	2.000817978549e-118
ENSG00000137100	DCTN3	1.33900115914932	1.1770245448603e-101
ENSG00000246705	H2AFJ	1.33878874251322	4.42088192301008e-140
ENSG00000161714	PLCD3	1.33775922405103	4.64987256233156e-154
ENSG00000104332	SFRP1	1.33496054596664	6.80902244511997e-69
ENSG00000281508	CDR1-AS	1.33462075633063	0.000277879746572543
ENSG00000124171	PARD6B	1.33426609113595	1.27938658081686e-41
ENSG00000099860	GADD45B	1.33295843307094	4.3997463149054e-31
ENSG00000112655	PTK7	1.33145913278658	3.84678029689698e-09
ENSG00000136867	SLC31A2	1.33138073868106	4.79115255719049e-22
ENSG00000171431	KRT20	1.33098666447535	8.3407291230805e-19
ENSG00000077420	APBB1IP	1.33045103280199	7.98324191055169e-05
ENSG00000143156	NME7	1.32987305867828	3.83692047006404e-72
ENSG00000263766	N.A.	1.32959985266987	0.00968279282661895
ENSG00000214279	N.A.	1.32918222872019	6.29744420790435e-05
ENSG00000116260	QSOX1	1.32912616007773	1.72854129580267e-159
ENSG00000148200	NR6A1	1.32910448548014	1.01826157607304e-22
ENSG00000182580	EPHB3	1.328854067359	1.72478311571321e-05
ENSG00000273247	N.A.	1.32815786715348	1.16346259385303e-05
ENSG00000165443	PHYHIPL	1.3277010113834	0.000103356854600459
ENSG00000169122	FAM110B	1.32750414664839	2.08405803462985e-10
ENSG00000095209	TMEM38B	1.32682495466538	2.13362345762215e-79
ENSG00000085185	BCORL1	1.32639721566704	9.18902107012142e-73
ENSG00000156384	SFR1	1.32564591996392	3.7167401382244e-42
ENSG00000211445	GPX3	1.32487328692596	3.88293405042125e-20
ENSG00000119655	NPC2	1.32465626551014	1.50854063196624e-116
ENSG00000241935	HOGA1	1.32451093682533	5.68971702880905e-14
ENSG00000147119	CHST7	1.32436780344691	3.04434078404953e-23
ENSG00000187531	SIRT7	1.3241791259369	4.5531361596907e-69
ENSG00000174721	FGFBP3	1.32205027896656	1.88830516227449e-10
ENSG00000187239	FNBP1	1.32195646592503	2.09935944568787e-71
ENSG00000101144	BMP7	1.32179988276377	0.00223378436181569
ENSG00000164332	UBLCP1	1.32108705912543	1.14829248009234e-119
ENSG00000257698	N.A.	1.32069873427677	3.83029871997397e-31
ENSG00000147082	CCNB3	1.32052241136968	4.10707837350691e-06
ENSG00000276131	N.A.	1.32015476958546	8.30311597189459e-05

ENSG00000269939	N.A.	1.31994453991238	0.00238185963914762
ENSG00000269906	N.A.	1.31939119453517	0.000109275846608999
ENSG00000100600	LGMN	1.318399884338	4.93374535269673e-169
ENSG00000245025	N.A.	1.31819530154336	0.000781575484649727
ENSG00000175567	UCP2	1.31792783899806	2.69840456544688e-113
ENSG00000111252	SH2B3	1.31710518661308	7.32211537298839e-55
ENSG00000112561	TFEB	1.3170970858963	2.74094892371529e-11
ENSG00000228594	C1orf233	1.31695994100367	8.09978749589894e-07
ENSG00000175550	DRAP1	1.31669339649146	1.59873346048073e-98
ENSG00000277283	N.A.	1.31627422847293	6.74910820351487e-13
ENSG00000134548	SPX	1.31582278363691	1.44408860371434e-25
ENSG00000107789	MINPP1	1.31502814663456	1.80464125934356e-120
ENSG00000180304	OAZ2	1.31467728439728	5.23622556296307e-166
ENSG00000145242	EPHA5	1.3144390550006	0.000193206700964811
ENSG00000067840	PDZD4	1.31438659196316	2.90368936943547e-18
ENSG00000175854	SWI5	1.31382527847924	2.94004063029914e-51
ENSG00000145022	TCTA	1.31305503707094	5.52608824766653e-50
ENSG00000237356	N.A.	1.31221493811153	0.00964813139684582
ENSG00000108262	GIT1	1.31157151581463	1.188938103563e-167
ENSG00000231742	LINC01273	1.31109618253575	1.98110932634635e-08
ENSG00000175866	BAIAP2	1.3089529354381	4.9349698437564e-45
ENSG00000164778	EN2	1.3086449064691	6.62664677584699e-05
ENSG00000174599	TRAM1L1	1.3083100638311	1.50558085440086e-05
ENSG00000123739	PLA2G12A	1.30789869558584	3.00468167603342e-53
ENSG00000100100	PIK3IP1	1.30746855585555	0.00169169941991882
ENSG00000283050	N.A.	1.30711700700822	8.80573178518407e-06
ENSG00000100564	PIGH	1.30689585828446	1.32309537432783e-49
ENSG00000151893	CACUL1	1.30661616740617	1.66471361502104e-142
ENSG00000144824	PHLDB2	1.3065102380961	1.84385553936997e-33
ENSG00000196843	ARID5A	1.3063223468457	6.82369340705745e-09
ENSG00000263004	N.A.	1.30611460383022	3.22369349038226e-06
ENSG00000279861	N.A.	1.30603367743199	0.0014075690041809
ENSG00000184897	H1FX	1.30594033240266	1.31268502371118e-124
ENSG00000128578	STRIP2	1.30495410836581	1.70252562774411e-49
ENSG00000097021	ACOT7	1.3046971776257	1.74392660597458e-138
ENSG00000079435	LIPE	1.30398230642428	4.87923579861822e-15
ENSG00000076706	MCAM	1.3034913368472	9.29525879441334e-19
ENSG00000253200	N.A.	1.30321432362084	1.75723606130917e-10
ENSG00000175197	DDIT3	1.30292389933151	1.31572717494162e-51
ENSG00000189159	HN1	1.30039663850054	7.04704380002012e-265
ENSG00000130413	STK33	1.30037541321806	3.8295654165071e-14
ENSG00000198346	ZNF813	1.30005927569084	0.00968844550063644
ENSG00000112033	PPARD	1.29928861448436	4.29293365995787e-40
ENSG00000136826	KLF4	1.29925229559963	1.64433002655922e-29
ENSG00000117395	EBNA1BP2	1.29868038291717	1.9658257334977e-165
ENSG00000141452	C18orf8	1.29815379939375	1.22856510653745e-65
ENSG00000224934	N.A.	1.29728832643614	7.70818758440291e-05
ENSG00000174306	ZHX3	1.29670168159078	6.13461358743933e-55
ENSG00000272831	N.A.	1.29625973414607	2.30203168093404e-19

ENSG00000118503	TNFAIP3	1.29596133627942	4.36795530101246e-13
ENSG00000165171	WBSCR27	1.29590178024809	4.23180755097529e-18
ENSG00000260686	N.A.	1.29585681516587	0.00680389400474393
ENSG00000084070	SMAP2	1.29536561706351	2.28936638423505e-54
ENSG00000255198	SNHG9	1.29495413272635	2.60203015373379e-16
ENSG00000163322	FAM175A	1.29482930997691	2.33192889973597e-37
ENSG00000136943	CTSV	1.29482024751599	2.06073883293164e-51
ENSG00000139044	B4GALNT3	1.29382570376163	4.75587919931748e-10
ENSG00000254402	LRRC24	1.2938137469752	4.18150220318434e-07
ENSG00000213533	TMEM110	1.29150038799529	0.00140273068356094
ENSG00000138138	ATAD1	1.29043906504314	5.61794969622356e-173
ENSG00000248971	KRT8P46	1.28920482981328	0.00658453401593753
ENSG00000057294	PKP2	1.28906513973783	5.60387341647912e-96
ENSG00000137364	TPMT	1.28905765628288	2.89598646212149e-53
ENSG00000198832	N.A.	1.28891794974609	4.36892156414194e-23
ENSG00000100271	TTLL1	1.28886890673423	5.10509685719147e-08
ENSG00000150977	RILPL2	1.28857291400516	1.24896367234275e-39
ENSG00000134569	LRP4	1.28856024332105	1.23614337784211e-42
ENSG00000138029	HADHB	1.28832401802102	1.47828251349579e-201
ENSG00000203879	GDI1	1.28768745227813	1.13697631160232e-146
ENSG00000069667	RORA	1.28737962190433	4.79827640076907e-05
ENSG00000223478	N.A.	1.28732459225013	2.17244356963025e-14
ENSG00000123154	WDR83	1.28638000570303	6.25032269462663e-19
ENSG00000003989	SLC7A2	1.28617609526147	3.56470377857881e-38
ENSG00000047634	SCML1	1.28464814167283	8.34624954071544e-83
ENSG00000141294	LRRC46	1.28358535403595	1.25563259009332e-09
ENSG00000088899	N.A.	1.28339063624147	3.62569402854403e-34
ENSG00000110721	CHKA	1.28319074593284	1.53748735454269e-122
ENSG00000101493	ZNF516	1.28314907485136	4.61565046402953e-15
ENSG00000184939	ZFP90	1.28077638772569	4.70134713503391e-61
ENSG00000166349	RAG1	1.28068541580178	0.00870557761753007
ENSG00000024862	CCDC28A	1.28023832525586	4.26389545678186e-38
ENSG00000068971	PPP2R5B	1.27988899667371	1.14069086653912e-31
ENSG00000125249	RAP2A	1.27978279109756	1.03368734598075e-68
ENSG00000009950	MLXIPL	1.27885208018621	2.15157989271149e-22
ENSG00000155816	FMN2	1.27629442950995	3.90084019890219e-19
ENSG00000119986	AVPI1	1.27600548186505	8.64847250546765e-107
ENSG00000141577	CEP131	1.27522152381929	5.73459787905135e-42
ENSG00000152642	GPD1L	1.27508352314283	5.86190952376796e-59
ENSG00000225138	N.A.	1.27497184168873	1.9244840102058e-11
ENSG00000181038	METTL23	1.27431583843394	1.7772043463456e-58
ENSG00000180573	HIST1H2AC	1.27431062625403	6.34846161380829e-38
ENSG00000272341	N.A.	1.27128606463796	0.000331699958627937
ENSG00000082014	SMARCD3	1.27017161254839	2.13486498186106e-31
ENSG00000151748	SAV1	1.27004287715434	9.73522318286659e-59
ENSG00000143537	ADAM15	1.26884220319396	9.2089831792567e-112
ENSG00000168785	TSPAN5	1.26872105434788	2.22573926452794e-26
ENSG00000165030	NFIL3	1.26841615195083	1.21558000708501e-39
ENSG00000221949	LINC01465	1.26670156359999	0.00175319253915494

ENSG00000173295	FAM86B3P	1.26622114459191	1.01651812537491e-12
ENSG00000168477	TNXB	1.26619430581767	5.19446255916134e-05
ENSG00000116005	PCYOX1	1.26520632657005	3.9637233851875e-205
ENSG00000006740	ARHGAP44	1.26458925259955	1.56400038874282e-09
ENSG00000137285	TUBB2B	1.26311522126574	4.59534378801568e-10
ENSG00000121766	ZCCHC17	1.26299437269353	1.21279362993384e-56
ENSG00000179094	PER1	1.26284583285158	9.03247726102748e-43
ENSG00000267321	N.A.	1.26248033733224	4.62885527549594e-21
ENSG00000105223	PLD3	1.2623504273857	9.53648712209852e-172
ENSG00000115419	GLS	1.26205130678434	2.00158188818237e-122
ENSG00000114473	IQCG	1.26100925249844	1.70902048404841e-14
ENSG00000120265	PCMT1	1.26066391071523	2.01152537079275e-138
ENSG00000001461	NIPAL3	1.25974045450101	2.61213656267355e-77
ENSG00000235374	SSR4P1	1.25921702376122	0.000170200778104449
ENSG00000177875	CCDC184	1.25693956168136	7.74529190151282e-26
ENSG00000114854	TNNC1	1.2562720190195	1.35693682366492e-09
ENSG00000231908	IDH1-AS1	1.25567844627949	0.00284971987887349
ENSG00000138646	HERC5	1.25454177820054	4.51382242730819e-23
ENSG00000133872	SARAF	1.25431474126077	1.44166209396386e-250
ENSG00000104765	BNIP3L	1.25410210640527	1.38782051583984e-136
ENSG00000180592	SKIDA1	1.25180746157961	7.78629729800403e-11
ENSG00000169220	RGS14	1.25118997275769	1.6462253295415e-14
ENSG00000073464	CLCN4	1.25047995095812	4.70147882704466e-54
ENSG00000139531	SUOX	1.24932997911559	7.19997625363536e-88
ENSG00000100325	ASCC2	1.24911400446063	3.33332112596798e-105
ENSG00000146151	HMGCLL1	1.24902156574881	1.88642280496226e-11
ENSG00000105982	RNF32	1.24873062512525	0.002476222752841
ENSG00000119013	NDUFB3	1.24639328454694	2.37524745549997e-84
ENSG00000224281	SLC25A5-AS1	1.24608127855827	0.00124533721119344
ENSG00000253404	N.A.	1.2454215182158	0.00914649393819702
ENSG00000182220	ATP6AP2	1.24500890437961	1.71545941662913e-248
ENSG00000115756	HPCAL1	1.24430626781575	1.31508987731276e-99
ENSG00000156642	NPTN	1.2435061904418	0
ENSG00000244005	NFS1	1.24338888779473	1.84102727416621e-64
ENSG00000030110	BAK1	1.24292284716092	4.08831995550864e-54
ENSG00000177076	ACER2	1.24208951189191	6.19437906797562e-08
ENSG00000152137	HSPB8	1.24185612565021	5.0140416203911e-37
ENSG00000125618	PAX8	1.24161607962719	0.00128316306450569
ENSG00000105401	CDC37	1.24151463442824	3.87061033401492e-186
ENSG00000260604	N.A.	1.24076853765669	3.55603317562893e-06
ENSG00000130270	ATP8B3	1.24056056790184	6.33324996028131e-16
ENSG00000125731	SH2D3A	1.23992046380027	1.07904698341584e-24
ENSG00000171720	HDAC3	1.23973919497095	3.44595041256184e-111
ENSG00000259153	N.A.	1.23850942567291	9.64634335419219e-11
ENSG00000265666	RARA-AS1	1.23848008558334	1.99003791145861e-05
ENSG00000125503	PPP1R12C	1.23844405596455	1.95603418784375e-136
ENSG00000250337	LINC01021	1.23559526366294	3.16488340331705e-36
ENSG00000017797	RALBP1	1.23415868685428	2.23936237524216e-149
ENSG00000173465	SSSCA1	1.23315060269536	5.41751449784329e-34

ENSG00000204923	FBXO48	1.23305656965509	2.22761102110753e-11
ENSG00000139344	AMDHD1	1.23294309261245	4.80093502809884e-15
ENSG00000134030	CTIF	1.23286755943902	2.98832536708554e-39
ENSG00000100241	SBF1	1.23283592972131	5.37950382364885e-153
ENSG00000143774	GUK1	1.2326235512997	7.15701259117361e-88
ENSG00000157216	SSBP3	1.2326115064655	3.31116085550288e-33
ENSG00000169641	LUZP1	1.23190728163007	1.63339320501289e-130
ENSG00000006459	KDM7A	1.23161121128604	1.01309387823069e-24
ENSG00000151151	IPMK	1.22999093334611	7.05697191410783e-48
ENSG00000064205	WISP2	1.22970916180018	1.16301722365632e-09
ENSG00000125726	CD70	1.22943951852586	2.87850030454385e-36
ENSG00000198839	ZNF277	1.22925874071654	2.16563887086784e-46
ENSG00000163590	PPM1L	1.22804433825601	6.30533287428316e-25
ENSG00000176531	PHLDB3	1.22746505895219	4.99040837886434e-15
ENSG00000042753	AP2S1	1.22714284833726	1.08495066196172e-131
ENSG00000154065	ANKRD29	1.22497939793364	5.85912061030189e-22
ENSG00000164877	MICALL2	1.22447460451392	1.29974652958461e-16
ENSG00000148180	GSN	1.22434177515794	2.33478927298839e-135
ENSG00000126368	NR1D1	1.22398926319485	2.30795156904505e-14
ENSG00000168779	SHOX2	1.22381018239811	9.65409033402486e-07
ENSG00000137494	ANKRD42	1.22377559667608	2.93265174218668e-42
ENSG00000029725	RABEP1	1.22319911922709	1.98382612905152e-171
ENSG00000258947	N.A.	1.22309791993483	0.000185923769524663
ENSG00000236859	NIFK-AS1	1.22270976258791	3.50194919301674e-06
ENSG00000196652	ZKSCAN5	1.22126211587734	7.87009183654713e-55
ENSG00000157578	LCA5L	1.22124590462537	4.03952427605126e-06
ENSG00000152409	JMY	1.22044565316878	5.05170345433864e-39
ENSG00000265692	N.A.	1.22036179179642	0.00901876250307417
ENSG00000165929	TC2N	1.22025059563331	1.10562177401129e-33
ENSG00000104833	TUBB4A	1.21848444260404	4.44448138559116e-51
ENSG00000137414	FAM8A1	1.21757041453463	8.32893256275704e-59
ENSG00000109472	CPE	1.2157339997754	8.46676546683202e-09
ENSG00000236255	N.A.	1.21495762401108	0.000736187593262694
ENSG00000111775	COX6A1	1.21295623839104	1.06147221547862e-130
ENSG00000146232	NFKBIE	1.21219940828298	1.1303605447571e-15
ENSG00000138111	TMEM180	1.21202952570754	5.87663640277981e-11
ENSG00000136840	ST6GALNAC4	1.21198625746232	2.74230917977285e-37
ENSG00000172345	STARD5	1.21182799683209	2.80084249161477e-06
ENSG00000235823	OLMALINC	1.21143331511532	1.07737693281503e-37
ENSG00000048471	SNX29	1.21008672362534	6.4990349714511e-42
ENSG00000158480	SPATA2	1.20872546334051	2.19057771583839e-29
ENSG00000131019	ULBP3	1.20865496021909	0.00127553232252319
ENSG00000271851	N.A.	1.2080273015408	0.000733166827572331
ENSG00000171503	ETFDH	1.20783746017817	7.72249662711655e-42
ENSG00000204604	ZNF468	1.20593201223013	6.0092076155979e-07
ENSG00000159239	C2orf81	1.20593176065953	1.63487547095052e-11
ENSG00000171843	MLLT3	1.20579903456417	9.22367129426869e-100
ENSG00000103811	CTSH	1.20499052878533	2.12563975724572e-26
ENSG00000261135	N.A.	1.20366117998885	6.61292763383046e-05

ENSG00000111144	LTA4H	1.20343003669438	1.20721915580432e-235
ENSG00000091039	OSBPL8	1.20326264923082	1.28560446445269e-114
ENSG00000169228	RAB24	1.20319872830098	3.17342324147811e-06
ENSG00000105053	VRK3	1.20265354806621	1.08957379605799e-67
ENSG00000143951	WDPCP	1.20231591529915	2.3690432407989e-16
ENSG00000162772	ATF3	1.20214548952034	3.4324874404415e-23
ENSG00000103642	LACTB	1.20145962024924	2.24428876140164e-57
ENSG00000173334	TRIB1	1.2011070038024	1.25343488198756e-47
ENSG00000196220	SRGAP3	1.20091619745124	3.20266488539269e-08
ENSG00000182993	C12orf60	1.20026268292754	1.57544733236298e-08
ENSG00000137216	TMEM63B	1.19954245691565	5.01701810747158e-65
ENSG00000123643	SLC36A1	1.19735571965437	2.1070449473294e-51
ENSG00000134108	ARL8B	1.19729578072061	8.20474787042657e-136
ENSG00000107959	PITRM1	1.19687468794031	1.92201634570381e-190
ENSG00000170917	NUDT6	1.19685183763109	1.80557530087895e-15
ENSG00000104976	SNAPC2	1.1966310882726	1.78643647695185e-22
ENSG00000057252	SOAT1	1.19506738039737	6.47809789557229e-135
ENSG00000083799	CYLD	1.19487551605293	3.65669744045453e-67
ENSG00000197261	C6orf141	1.19439235136335	1.18077230082955e-20
ENSG00000136842	TMOD1	1.19392943280277	1.46392733863892e-06
ENSG00000163521	GLB1L	1.19245901012706	2.60926604851889e-16
ENSG00000198794	SCAMP5	1.19244517902226	3.69805300725262e-37
ENSG00000182272	B4GALNT4	1.19079856696923	2.01622571714544e-12
ENSG00000226137	BAIAP2-AS1	1.19044754183169	7.35594900296959e-38
ENSG00000204560	DHX16	1.19043994719727	1.84216670778912e-93
ENSG00000125952	MAX	1.19026326703466	2.19869803327133e-81
ENSG00000170100	ZNF778	1.19006792483576	5.59022843344331e-26
ENSG00000100304	TTLL12	1.19000480304894	7.86988454405386e-51
ENSG00000140307	GTF2A2	1.18897812721355	4.66124180337736e-141
ENSG00000167487	KLHL26	1.18816041102475	5.90310577143525e-14
ENSG00000057657	PRDM1	1.18809030650885	0.00113653939396163
ENSG00000088826	SMOX	1.18776902043114	5.81343987144473e-63
ENSG00000235890	TSPEAR-AS1	1.18770598113688	0.00373697761918155
ENSG00000229619	MBNL1-AS1	1.18756160492983	0.000949687224721093
ENSG00000130449	ZSWIM6	1.18734020338563	5.81481493319547e-54
ENSG00000135677	GNS	1.18685527058794	3.39708231923205e-221
ENSG00000154310	TNIK	1.18659489262171	1.94936126496978e-25
ENSG00000129968	ABHD17A	1.18492474156397	8.89310164848589e-45
ENSG00000032219	ARID4A	1.18478649068589	9.76936291231569e-55
ENSG00000132003	ZSWIM4	1.18470767603376	9.90702592580989e-14
ENSG00000168306	ACOX2	1.18432766391772	1.26648608238059e-06
ENSG00000151276	MAGI1	1.18353677668054	6.75360960082331e-35
ENSG00000170836	PPM1D	1.18314975035111	4.26155057258705e-49
ENSG00000068137	PLEKHH3	1.18185935325967	5.44317830340146e-40
ENSG00000141576	RNF157	1.18105925148156	1.32919308525972e-37
ENSG00000107282	APBA1	1.18005560188214	0.00381144633930054
ENSG00000084092	NOA1	1.17959791682782	8.32844782979031e-54
ENSG00000232093	N.A.	1.17790681645787	1.20949877153323e-05
ENSG00000134243	SORT1	1.17787422030155	1.22069584173614e-206

ENSG00000013583	HEBP1	1.17728152149107	2.57444676057883e-63
ENSG00000169914	OTUD3	1.17672350409314	1.69268485021689e-25
ENSG00000103021	CCDC113	1.17487192453539	2.80526842934375e-25
ENSG00000157796	WDR19	1.17358285287884	3.27523572138315e-55
ENSG00000187860	CCDC157	1.17349194255776	6.42650784854498e-07
ENSG00000117971	CHRNB4	1.17317205975467	0.00105382254646969
ENSG00000170776	AKAP13	1.17188268394107	4.73225605530876e-184
ENSG00000142530	FAM71E1	1.1711732874021	0.000646304396111673
ENSG00000175602	CCDC85B	1.17099680100723	7.93335917429078e-22
ENSG00000165272	AQP3	1.17034088504477	1.86992852207831e-61
ENSG00000198792	TMEM184B	1.16965824172908	1.67678315910714e-86
ENSG00000146700	SSC4D	1.16926386327167	0.0068261214900768
ENSG00000162032	SPSB3	1.16925592642704	6.48767215330057e-28
ENSG00000137601	NEK1	1.16878693280215	1.19097944091054e-24
ENSG00000119471	HSDL2	1.16803762727263	3.01144947079754e-135
ENSG00000087299	L2HGDH	1.1677975248688	6.37736729322044e-70
ENSG00000197837	HIST4H4	1.16759196689075	0.00171268562476864
ENSG00000116016	EPAS1	1.16749021433087	8.90560375268617e-82
ENSG00000005882	PDK2	1.16697610954169	1.95023167687808e-40
ENSG00000162039	MEIOB	1.16654825259536	1.56768645314844e-12
ENSG00000247934	N.A.	1.16637306780155	0.00627432527034001
ENSG00000115468	EFHD1	1.16623601405894	2.85438819342881e-05
ENSG00000279207	N.A.	1.16579101295725	2.1967186313527e-45
ENSG00000276476	LINC00540	1.16493181975723	0.000102526055310067
ENSG00000119326	CTNNAL1	1.16489050687744	5.51211885197859e-121
ENSG00000148331	ASB6	1.16453462238101	1.70136448295645e-69
ENSG00000262621	N.A.	1.16397156743035	0.00379616865159784
ENSG00000273352	N.A.	1.16340968116029	0.00573532412589776
ENSG00000141480	ARRB2	1.16310753727075	1.24803745155846e-88
ENSG00000232040	ZBED9	1.16294531288321	1.11255005577818e-13
ENSG00000166762	CATSPER2	1.16287614561992	0.00083913419190269
ENSG00000250510	GPR162	1.16206184844687	1.10477416444666e-07
ENSG00000146555	SDK1	1.16156200681162	2.28893576381463e-09
ENSG00000177885	GRB2	1.16126046419698	4.6407778554944e-265
ENSG00000070540	WIPI1	1.16093240819741	1.13874051509527e-47
ENSG00000135046	ANXA1	1.16057210395561	4.06339030785299e-221
ENSG00000130479	MAP1S	1.16050984361021	7.98680806001463e-34
ENSG00000127419	TMEM175	1.16010418279527	1.7009632697916e-22
ENSG00000151229	SLC2A13	1.15929601309688	1.69720660927818e-49
ENSG00000186026	ZNF284	1.15924676619415	0.000508656198894658
ENSG00000149646	CNBD2	1.15916559003515	4.91078715877645e-05
ENSG00000159363	ATP13A2	1.15906043638249	3.16858511367312e-66
ENSG00000177675	CD163L1	1.15899088885958	5.64465859341107e-05
ENSG00000188290	HES4	1.15883282099702	3.17796920558525e-08
ENSG00000196235	SUPT5H	1.15860449681087	5.42754298825144e-175
ENSG00000240541	TM4SF1-AS1	1.15847288542459	0.00696526464475855
ENSG00000125734	GPR108	1.15728710162667	8.64302636833697e-68
ENSG00000148339	SLC25A25	1.15602434478233	1.06295937655215e-41
ENSG00000136235	GPNMB	1.15572575463916	1.38023470320202e-06

ENSG00000110651	CD81	1.15501106336762	2.75387546795808e-162
ENSG00000005187	ACSM3	1.15441511304575	2.2991726232359e-47
ENSG00000128581	IFT22	1.1543447495708	4.33346342239583e-68
ENSG00000137502	RAB30	1.15431650288637	1.77920979197616e-27
ENSG00000159082	SYNJ1	1.15399551589811	7.85399282211262e-33
ENSG00000105792	CFAP69	1.153312943138	1.19686444740699e-10
ENSG00000112406	HECA	1.15311926781421	1.80563974162536e-21
ENSG00000204178	TMEM57	1.15292055430348	1.47408068281697e-38
ENSG00000107175	CREB3	1.15099555133394	2.43107941020985e-72
ENSG00000102385	DRP2	1.15089270924219	4.35991875891825e-07
ENSG00000151611	MMAA	1.15082829454827	2.98860458454059e-09
ENSG00000140545	MFGE8	1.15053076661649	3.70809426400053e-91
ENSG00000184226	PCDH9	1.15043490052942	1.55257502958247e-131
ENSG00000166575	TMEM135	1.14853608220787	4.49507854169005e-76
ENSG00000153993	SEMA3D	1.14803261039172	0.003303207262085
ENSG00000115317	HTRA2	1.14766633314464	1.21432135039514e-38
ENSG00000104756	KCTD9	1.14760931330559	1.77648266779065e-127
ENSG00000115762	PLEKHB2	1.1472001370983	3.91521705919771e-189
ENSG00000154265	ABCA5	1.14696809335137	8.19305848008896e-37
ENSG00000140948	ZCCHC14	1.14666062409978	1.25243136663439e-46
ENSG00000034693	PEX3	1.14619771146576	4.41755512255764e-26
ENSG00000107872	FBXL15	1.14607768238064	1.48365082309972e-13
ENSG00000176438	SYNE3	1.14599454065226	0.00342934029318086
ENSG00000083812	ZNF324	1.14517731059106	6.31888197570735e-20
ENSG00000155097	ATP6V1C1	1.14363945780415	3.17688849044239e-118
ENSG00000108774	RAB5C	1.14249792515384	6.81382713102322e-104
ENSG00000184381	PLA2G6	1.14242659978223	6.39802423998145e-15
ENSG00000148842	CNNM2	1.14234869268255	3.69223653229136e-27
ENSG00000260233	SSSCA1-AS1	1.14221740331139	0.00298258494542469
ENSG00000186111	PIP5K1C	1.14151106156873	5.47636542331713e-56
ENSG00000113966	ARL6	1.14028454049867	2.39518910386722e-25
ENSG00000145632	PLK2	1.14026725441422	1.37716928849386e-63
ENSG00000131446	MGAT1	1.14024215915259	6.81592189165721e-119
ENSG00000197081	IGF2R	1.14022111062326	2.05565353454928e-152
ENSG00000070371	CLTCL1	1.13922086008643	1.71958864471159e-18
ENSG00000155876	RRAGA	1.13913550845827	1.02423161299479e-117
ENSG00000100583	SAMD15	1.13910876430414	3.55639922301152e-08
ENSG00000135916	ITM2C	1.13900751771742	1.88508406245466e-93
ENSG00000164347	GFM2	1.1372822257923	1.30625711359477e-84
ENSG00000203668	CHML	1.13723495052773	5.85537480830605e-101
ENSG00000272769	N.A.	1.13672675234562	3.73420308994399e-07
ENSG00000167930	ITFG3	1.13644857906486	2.05691944788372e-42
ENSG00000147400	CETN2	1.13602008173415	1.05744956720499e-116
ENSG00000180938	ZNF572	1.13575761432524	9.37162820929707e-06
ENSG00000183718	TRIM52	1.13313757251631	4.41310926237532e-39
ENSG00000121690	DEPDC7	1.1330569766038	2.51059517839393e-24
ENSG00000106299	WASL	1.1323060042059	3.48590915513628e-98
ENSG00000107771	CCSER2	1.13151202780331	2.79057483391055e-89
ENSG00000198130	HIBCH	1.13117093971074	6.34001861732015e-36

ENSG00000259642	ST20-AS1	1.1311258521689	7.70632018657745e-08
ENSG00000111231	GPN3	1.1309587068056	1.75053982460735e-81
ENSG00000100299	ARSA	1.13084446777441	2.14132372275056e-29
ENSG00000188786	MTF1	1.1302222331979	4.80039958436022e-43
ENSG00000174939	ASPHD1	1.12956957385968	3.06605456582104e-07
ENSG00000170629	DPY19L2P2	1.12862904489446	3.10649556386669e-11
ENSG00000169155	ZBTB43	1.12846915151768	1.37872147961914e-35
ENSG00000127054	CPSF3L	1.12828651740476	1.25839055239468e-65
ENSG00000198258	UBL5	1.12765169625873	7.77663052223596e-84
ENSG00000177606	JUN	1.12734562116503	1.36902987732743e-26
ENSG00000172270	BSG	1.1272574499607	4.5581011016203e-202
ENSG00000259429	UBE2Q2P2	1.12704876139567	3.65410567025187e-05
ENSG00000135919	SERPINE2	1.12697678088137	1.0436034067383e-33
ENSG00000160345	C9orf116	1.12673361470416	3.20103322569702e-12
ENSG00000213062	N.A.	1.12666786933831	0.00600771493605663
ENSG00000118680	MYL12B	1.12630072470337	2.03145464807756e-187
ENSG00000186166	CCDC84	1.1261505649918	8.98163564270335e-21
ENSG00000115414	FN1	1.12598785205377	1.77672473929148e-78
ENSG00000120709	FAM53C	1.1258431388004	4.58159950884305e-86
ENSG00000130517	PGPEP1	1.12579892343206	1.49157547112062e-61
ENSG00000188010	MORN2	1.12419861746977	4.88081194155819e-36
ENSG00000136436	CALCOCO2	1.12271244486047	4.27158447075702e-197
ENSG00000148225	WDR31	1.12256589041929	6.24314060336345e-16
ENSG00000196083	IL1RAP	1.12225419842586	4.80005975725952e-24
ENSG00000137274	BPHL	1.12208541797799	1.53680959162583e-33
ENSG00000110060	PUS3	1.12166935103631	8.33083764183029e-13
ENSG00000106537	TSPAN13	1.1214227770757	3.27268725296658e-147
ENSG00000215105	TTC3P1	1.12125642092158	7.25101901609755e-06
ENSG00000113916	BCL6	1.12070862602709	8.7954023318087e-40
ENSG00000186318	BACE1	1.12060633425283	1.32443258009757e-52
ENSG00000165887	ANKRD2	1.1194361349822	0.00150047000034798
ENSG00000117266	CDK18	1.11930969870825	4.8066370783716e-07
ENSG00000185467	KPNA7	1.11925788339476	0.00188809223193492
ENSG00000008256	CYTH3	1.11800633694997	7.20981163380356e-117
ENSG00000159905	ZNF221	1.11796808540666	0.000968288235785516
ENSG00000118855	MFSD1	1.11747000522401	1.27902746054421e-86
ENSG00000264207	N.A.	1.11731782571736	1.18041890076787e-06
ENSG00000171703	TCEA2	1.11699767197529	9.58052301498343e-27
ENSG00000165861	ZFYVE1	1.11695021231451	2.4408935533917e-43
ENSG00000105419	MEIS3	1.11601642668864	2.20974731371023e-78
ENSG00000167658	EEF2	1.11601616361544	6.19872310978397e-197
ENSG00000130363	RSPH3	1.11572544734934	1.33180995271455e-25
ENSG00000214562	NUTM2D	1.1151090865586	0.00464727015444592
ENSG00000117676	RPS6KA1	1.11473930992979	1.03992015309877e-49
ENSG00000182912	TSPEAR-AS2	1.11459810957229	0.000609695895876548
ENSG00000105085	MED26	1.11425447619822	5.33806278463323e-12
ENSG00000196118	C16orf93	1.11411747972875	2.37471489870164e-14
ENSG00000189241	TSPYL1	1.11407562431234	1.34434465027105e-98
ENSG00000066135	KDM4A	1.11372698050078	8.12262560771621e-72

ENSG00000125122	LRRC29	1.11261773944707	0.00273583385256108
ENSG00000149016	TUT1	1.11260655241899	2.18026195530947e-15
ENSG00000255112	CHMP1B	1.11108647598931	1.02199773070449e-89
ENSG00000106351	AGFG2	1.11038632221828	1.06518144507967e-73
ENSG00000088543	C3orf18	1.10873626262723	1.19543141820113e-08
ENSG00000147416	ATP6V1B2	1.10821718814671	5.84596830584791e-149
ENSG00000089693	MLF2	1.10768837791565	3.52017450588257e-226
ENSG00000173137	ADCK5	1.10719905154753	4.14498193514035e-17
ENSG00000171608	PIK3CD	1.10695565169855	3.24353365442948e-24
ENSG00000121680	PEX16	1.10601790321014	3.22967335309717e-25
ENSG00000230844	ZNF674-AS1	1.10599937681471	1.19447708385348e-08
ENSG00000122958	VPS26A	1.10599179816237	5.3934940058977e-127
ENSG00000178104	PDE4DIP	1.10532285979097	4.33051640699472e-43
ENSG00000025039	RRAGD	1.1045981725514	7.47179953860037e-27
ENSG00000164867	NOS3	1.10414202006843	0.00501417467363177
ENSG00000090776	EFNB1	1.10240795013792	1.49364808204434e-19
ENSG00000179833	SERTAD2	1.10029872249603	2.70991073235862e-50
ENSG00000164117	FBXO8	1.10028670661002	1.19421235756126e-30
ENSG00000061938	TNK2	1.10008604868504	4.16921012323967e-40
ENSG00000132563	REEP2	1.09985061625336	3.7724133592736e-17
ENSG00000130940	CASZ1	1.09973259428735	6.72753013468929e-15
ENSG00000116985	BMP8B	1.09959650150368	9.60640707815731e-27
ENSG00000010626	LRRC23	1.09946618710044	2.32661248803095e-32
ENSG00000124243	BCAS4	1.09883417542437	1.12813540733059e-42
ENSG00000132906	CASP9	1.09863281079612	2.17300013330946e-33
ENSG00000198720	ANKRD13B	1.09791641496773	2.65829929441062e-21
ENSG00000071794	HLTF	1.09784990063558	4.04638436446457e-107
ENSG00000168038	ULK4	1.09778318776387	7.20014162773048e-10
ENSG00000055609	KMT2C	1.09712755252989	1.54598145832926e-63
ENSG00000080298	RFX3	1.09709468164517	4.39481648628005e-24
ENSG00000113108	APBB3	1.09704532258868	1.0895216932943e-11
ENSG00000099330	OCEL1	1.09619704978814	1.71420106682026e-19
ENSG00000179240	N.A.	1.09591936803345	1.32833098459193e-08
ENSG00000170043	TRAPPC1	1.09579716083641	1.13023974576066e-65
ENSG00000182796	TMEM198B	1.09573420369762	1.17629567342915e-21
ENSG00000115738	ID2	1.09384619566643	3.27666066480273e-37
ENSG00000064309	CDON	1.09317464631961	9.6020238580168e-19
ENSG00000196743	GM2A	1.09314253334749	8.78729065959528e-70
ENSG00000186625	KATNA1	1.09293838781122	4.50745414626055e-25
ENSG00000176058	TPRN	1.09277863825564	7.44877575425723e-21
ENSG00000158109	TPRG1L	1.09264076594717	4.16495552314953e-41
ENSG00000048052	HDAC9	1.09104693572631	0.00156079582259176
ENSG00000164953	TMEM67	1.09086758498851	4.20800358194705e-30
ENSG00000083814	ZNF671	1.09024710644764	0.00227354636055586
ENSG00000167632	TRAPPC9	1.088813445461	1.19928665434726e-70
ENSG00000155621	C9orf85	1.08852040483302	6.75655478865395e-20
ENSG00000083535	PIBF1	1.08808265770902	5.38407673818681e-22
ENSG00000244187	TMEM141	1.08802011473409	7.08015131755691e-32
ENSG00000139697	SBNO1	1.08749326208036	4.84147899373828e-171

ENSG00000227908	N.A.	1.08743706404186	0.00925921928317523
ENSG00000140104	C14orf79	1.08742818316733	6.37281673907934e-26
ENSG00000125037	EMC3	1.08727761702519	2.45325055341734e-101
ENSG00000105339	DENND3	1.08724474728601	2.89886227987755e-60
ENSG00000146085	MUT	1.08699690532108	3.23270716675989e-57
ENSG00000105063	PPP6R1	1.08623332259053	5.47092743589878e-99
ENSG00000064601	CTSA	1.08565781969221	4.9097558208844e-97
ENSG00000114383	TUSC2	1.08557321423319	1.72609666682133e-37
ENSG00000169548	ZNF280A	1.0855397141682	9.32408769881842e-07
ENSG00000182648	LINC01006	1.08451788038381	2.60550979094848e-09
ENSG00000105355	PLIN3	1.08207534925874	2.44195094137346e-91
ENSG00000281501	SEPSECS-AS1	1.08187096040958	0.00405668842435811
ENSG00000101350	KIF3B	1.08164862958203	2.39744176355942e-167
ENSG00000143479	DYRK3	1.08154996452002	3.30251409440328e-18
ENSG00000240204	SMKR1	1.08016940501768	6.55308741181065e-12
ENSG00000126453	BCL2L12	1.0800350458199	3.47846771606469e-48
ENSG00000133318	RTN3	1.07981416599824	3.55273695666391e-163
ENSG00000169193	CCDC126	1.07978568950077	5.59041713587416e-17
ENSG00000143079	CTTNBP2NL	1.07972006445486	2.83692982308419e-45
ENSG00000116525	TRIM62	1.07908602488889	1.61094217880396e-09
ENSG00000180185	FAHD1	1.07891308817028	4.88952352819611e-40
ENSG00000140950	TLDC1	1.0788400068316	9.26384971448902e-65
ENSG00000091129	NRCAM	1.07853937218459	6.10206234894433e-16
ENSG00000089820	ARHGAP4	1.07732014482462	0.000699390679125286
ENSG00000137824	RMDN3	1.07727127313452	2.45724215588113e-45
ENSG00000103512	NOMO1	1.07717211891825	9.01721331859766e-96
ENSG00000143995	MEIS1	1.07695926015322	8.25919231036899e-09
ENSG00000132801	ZSWIM3	1.07689369989017	1.03160602491633e-06
ENSG00000197858	GPAA1	1.07653287815708	1.31289045064389e-34
ENSG00000253953	PCDHGB4	1.07630002605466	0.00231485600153996
ENSG00000140320	BAHD1	1.07590874262678	2.69484714872067e-35
ENSG00000165424	ZCCHC24	1.07574722326268	1.20500713212633e-19
ENSG00000260267	N.A.	1.07567822651468	1.09615363038548e-10
ENSG00000146950	SHROOM2	1.07547304861123	1.5117301219559e-13
ENSG00000228265	RALY-AS1	1.07518855722702	0.000179763158373443
ENSG00000150627	WDR17	1.07518267055897	5.08906620673015e-23
ENSG00000174705	SH3PXD2B	1.07515260295295	1.55170897870483e-82
ENSG00000248508	SRP14-AS1	1.07491350225048	1.35222927791461e-06
ENSG00000105427	CNFN	1.07342831108581	2.59510084670095e-09
ENSG00000134313	KIDINS220	1.07293610100124	1.17486706504593e-104
ENSG00000104154	SLC30A4	1.07204277459724	1.21218533320327e-17
ENSG00000173013	CCDC96	1.0715307667133	4.93315168999325e-05
ENSG00000119636	CCDC176	1.07149482911519	3.58716738999932e-11
ENSG00000101745	ANKRD12	1.07138881096675	3.74782624548918e-48
ENSG00000117758	STX12	1.07126794290111	1.95303045724375e-48
ENSG00000115486	GGCX	1.07123061028472	1.0910723369564e-76
ENSG00000167645	YIF1B	1.07073790161462	1.79307975235545e-59
ENSG00000147180	ZNF711	1.07055173313692	2.94432890935909e-16
ENSG00000179314	WSCD1	1.07017729674526	6.74231443900524e-25

ENSG00000089091	DZANK1	1.0696718074187	1.764956577171e-15
ENSG00000268069	N.A.	1.06966971415587	0.00115789035243286
ENSG00000123395	ATG101	1.06962619558524	1.1512662639405e-61
ENSG00000112096	SOD2	1.06899421020714	7.27305349128588e-101
ENSG00000272275	N.A.	1.06887590992142	0.00574032485112272
ENSG00000141971	MVB12A	1.06763608277553	5.86462831464674e-21
ENSG00000179454	KLHL28	1.06732822106036	5.46843831278466e-34
ENSG00000090006	LTBP4	1.06728569433862	1.98900083880069e-77
ENSG00000237310	N.A.	1.06668670782257	4.2428407076232e-06
ENSG00000161277	THAP8	1.06660339491854	1.33211958214961e-13
ENSG00000198730	CTR9	1.06623521307159	4.15595686916958e-122
ENSG00000161091	MFSD12	1.06597543550822	2.0016860542882e-78
ENSG00000135917	SLC19A3	1.06577808023587	1.02363639791992e-19
ENSG00000166780	C16orf45	1.06543649030039	4.24522082782922e-47
ENSG00000225151	GOLGA2P7	1.065187321426	6.14703493405993e-67
ENSG00000166734	CASC4	1.06405670675721	7.08676092672797e-123
ENSG00000003393	ALS2	1.06261422800116	1.0859873302672e-57
ENSG00000167291	TBC1D16	1.06246158770831	2.07035575396658e-77
ENSG00000197557	TTC30A	1.06203199825806	1.03612125235441e-22
ENSG00000100744	GSKIP	1.06177800377979	6.79444099164027e-85
ENSG00000156050	FAM161B	1.06040494731314	1.25752344994321e-09
ENSG00000258057	BCDIN3D-AS1	1.06028811360478	2.90060012972894e-06
ENSG00000204248	COL11A2	1.0586123333913	0.00212696019595088
ENSG00000115457	IGFBP2	1.05844799962199	1.76904822829239e-45
ENSG00000168781	PPIP5K1	1.05736026974981	7.95995921759399e-18
ENSG00000081320	STK17B	1.0572346610366	9.82763282978632e-27
ENSG00000197283	SYNGAP1	1.0570231456203	7.81312425421137e-14
ENSG00000185339	TCN2	1.05672701762756	1.02572578150303e-21
ENSG00000105656	ELL	1.05625924024887	1.33920041250328e-15
ENSG00000148798	INA	1.05545435982895	3.16724131139668e-58
ENSG00000162642	C1orf52	1.05496716488098	1.93847084313355e-21
ENSG00000006062	MAP3K14	1.05461464823832	3.02001176049526e-57
ENSG00000079277	MKNK1	1.05385421963742	3.30240611185717e-41
ENSG00000006283	CACNA1G	1.05312175890638	2.26903777708958e-59
ENSG00000158467	AHCYL2	1.05228469745233	4.48554855621721e-53
ENSG00000149927	DOC2A	1.05163639596934	0.000207491428723911
ENSG00000276390	N.A.	1.0511690071515	0.00357986665419589
ENSG00000104853	CLPTM1	1.05093978641499	3.96719101251248e-141
ENSG00000165752	STK32C	1.0506059190392	3.58557728085102e-28
ENSG00000100364	KIAA0930	1.05044611618988	1.43208977642061e-89
ENSG00000125648	SLC25A23	1.0499793578461	3.44698431340377e-57
ENSG00000125347	IRF1	1.04983503420679	4.14380533339687e-23
ENSG00000205629	LCMT1	1.04898160433639	6.34225050904137e-43
ENSG00000224914	LINC00863	1.04668363770242	3.53204722286067e-10
ENSG00000076604	TRAF4	1.0463175918813	7.64268006423484e-75
ENSG00000107819	SFXN3	1.0460486939763	6.22892259920066e-59
ENSG00000178826	TMEM139	1.04461105814884	1.96753634035084e-12
ENSG00000169490	TM2D2	1.04399261550735	5.91873533106354e-97
ENSG00000250159	N.A.	1.04376157421276	0.00724727364864548

ENSG00000028839	TBPL1	1.04374825010485	1.47875922353782e-32
ENSG00000089486	CDIP1	1.04356304339774	3.26070363176066e-11
ENSG00000177888	ZBTB41	1.04325950119878	1.62469576784552e-46
ENSG00000264920	N.A.	1.04211475247504	4.04736943355207e-05
ENSG00000130997	POLN	1.04156219782951	0.000218417973220232
ENSG00000122068	FYTTD1	1.04148582312774	6.51310773230973e-64
ENSG00000138756	BMP2K	1.03986818700499	6.30664964735928e-35
ENSG00000229320	KRT8P12	1.03982687986889	9.6467330523378e-11
ENSG00000073803	MAP3K13	1.03972053692452	2.99889211025463e-27
ENSG00000103657	HERC1	1.03893981290882	1.09491568099494e-82
ENSG00000145439	CBR4	1.03840923105167	7.44061021852981e-40
ENSG00000101335	MYL9	1.03824216994682	0.00976137837450433
ENSG00000132475	H3F3B	1.03813821646248	8.96258875338342e-224
ENSG00000164976	KIAA1161	1.03700125796281	6.04113193230672e-56
ENSG00000226200	SGMS1-AS1	1.03685657413702	1.25902456031615e-08
ENSG00000100417	PMM1	1.03669454960932	2.65003960291467e-39
ENSG00000077463	SIRT6	1.03522193982393	4.24511308126473e-19
ENSG00000125895	TMEM74B	1.03405026050058	0.001673171796231
ENSG00000175198	PCCA	1.03372189021531	2.82288152464835e-36
ENSG00000196754	S100A2	1.03368420875906	0.00145570303498757
ENSG00000119661	DNAL1	1.03274002187054	3.04694128816116e-43
ENSG00000120656	TAF12	1.03263903028625	4.45378987606454e-29
ENSG00000112576	CCND3	1.03219496942451	7.51647604913583e-83
ENSG00000168528	SERINC2	1.031774994347	2.37361173521714e-34
ENSG00000170684	ZNF296	1.03144760865424	0.00550590984931018
ENSG00000138604	GLCE	1.03114048085252	9.61851520323987e-67
ENSG00000068383	INPP5A	1.03045495481934	4.36301783951354e-33
ENSG00000092010	PSME1	1.03008058677604	7.50376411560823e-68
ENSG00000106415	GLCCI1	1.03002091515496	1.82507240179859e-09
ENSG00000124226	RNF114	1.02970375247771	5.29004816053424e-93
ENSG00000157045	NTAN1	1.02968900782616	4.6816696400086e-37
ENSG00000130165	ELOF1	1.02911583221842	2.09053992514823e-40
ENSG00000204956	PCDHGA1	1.02854897024668	0.000106071386683094
ENSG00000137434	C6orf52	1.02824374823331	0.00508863102413294
ENSG00000156345	CDK20	1.02791605645178	6.66209259827143e-12
ENSG00000160908	ZNF394	1.02736774018345	1.07026148442282e-22
ENSG00000122694	GLIPR2	1.02689278734964	5.7516652556169e-06
ENSG00000108107	RPL28	1.02672898639987	4.94792379700988e-26
ENSG00000148484	RSU1	1.02657407669264	1.14793413609413e-93
ENSG00000144560	VGLL4	1.02472414251723	1.32083137816967e-64
ENSG00000075426	FOSL2	1.02397098004998	4.1098011169493e-76
ENSG00000145332	KLHL8	1.02389776770635	4.47662173035079e-34
ENSG00000087074	PPP1R15A	1.02287888464252	4.7891855490809e-41
ENSG00000113369	ARRDC3	1.02284690433102	1.31211455000267e-55
ENSG00000126803	HSPA2	1.0225527366241	1.81434225258274e-49
ENSG00000168952	STXBP6	1.02222192397717	2.94595501042381e-11
ENSG00000080823	MOK	1.02089369217024	8.98000497715544e-17
ENSG00000174943	KCTD13	1.02074418545412	2.81419547985877e-21
ENSG00000171223	JUNB	1.02010147005364	1.15094874481485e-38

ENSG00000105357	MYH14	1.01974910809517	2.58659888596211e-38
ENSG00000196668	LINC00173	1.01972125648312	3.51660046690487e-06
ENSG00000135951	TSGA10	1.01953240937524	0.000386266740865445
ENSG00000189143	CLDN4	1.01822778399817	5.05094485628016e-05
ENSG00000186615	KTN1-AS1	1.01794129664787	1.65560197487455e-07
ENSG00000185187	SIGIRR	1.01765295368947	1.05326899649651e-19
ENSG00000155313	USP25	1.01723287333693	3.92128099433082e-67
ENSG00000183496	MEX3B	1.01699692335498	1.24062406336311e-07
ENSG00000086065	CHMP5	1.01590325338773	8.48121152683475e-95
ENSG00000168256	NKIRAS2	1.0155729349705	4.7595693193082e-80
ENSG00000242732	RGAG4	1.01498073807876	8.52021456468026e-13
ENSG00000198468	FLVCR1-AS1	1.0149652584165	1.4304413636015e-06
ENSG00000205476	CCDC85C	1.01469936209647	1.33447538664966e-75
ENSG00000150867	PIP4K2A	1.01330489171539	8.89874558407082e-73
ENSG00000170385	SLC30A1	1.01260039865937	6.1572251210394e-54
ENSG00000172731	LRRC20	1.01208328394345	3.8695015483733e-53
ENSG00000197180	N.A.	1.01108578897548	0.000404696908864805
ENSG00000233251	N.A.	1.01103831363078	0.00220788554738272
ENSG00000214530	STARD10	1.01103187551983	7.30181214763796e-14
ENSG00000188342	GTF2F2	1.01065003691451	1.15910328339374e-42
ENSG00000123159	GIPC1	1.01034404601812	1.51167381993553e-88
ENSG00000169032	MAP2K1	1.00944351387322	1.41130400711697e-116
ENSG00000251669	FAM86EP	1.00913776092804	2.49475731220793e-06
ENSG00000006007	GDE1	1.00865862344599	2.91027573252695e-158
ENSG00000169855	ROBO1	1.00759364283973	2.36257044391351e-80
ENSG00000106086	PLEKHA8	1.0072520025834	3.85345091201802e-44
ENSG00000197608	ZNF841	1.00671475317725	4.15256896702117e-15
ENSG00000144959	NCEH1	1.00632697795916	1.51929821761482e-122
ENSG00000049130	KITLG	1.00543480552178	1.21218820596275e-17
ENSG00000116903	EXOC8	1.00514563798413	1.11774201824066e-50
ENSG00000173258	ZNF483	1.00480184474784	0.000236237368894845
ENSG00000184384	MAML2	1.0046137340705	5.43252239095867e-06
ENSG00000240652	N.A.	1.0045454820302	0.000499459121972524
ENSG00000185880	TRIM69	1.00371675412644	1.43656134589115e-20
ENSG00000085117	CD82	1.00370713461571	7.42293111312643e-18
ENSG00000103264	FBXO31	1.00333511319458	5.37539203301283e-37
ENSG00000088256	GNA11	1.00308125476396	5.85225094578876e-86
ENSG00000168159	RNF187	1.00294288899125	3.84748583356263e-78
ENSG00000180479	ZNF571	1.00292886657599	0.00857409740070057
ENSG00000167397	VKORC1	1.00189260600787	4.88591793801892e-26
ENSG00000131495	NDUFA2	1.0016076105459	6.29102498914703e-50
ENSG00000141556	TBCD	1.00144206138524	6.81114438233491e-109
ENSG00000186577	C6orf1	1.00125941111238	9.61945600430362e-16
ENSG00000173114	LRRN3	1.00033023520646	0.000831456705965015
ENSG00000099817	POLR2E	1.00015911505229	2.66293196774063e-77
ENSG00000142669	SH3BGRL3	1.00013576426674	7.99738696303728e-46
ENSG00000137547	MRPL15	-1.00027321955677	7.94199360599359e-49
ENSG00000111011	RSRC2	-1.00061960293442	6.31520708409952e-68
ENSG00000103174	NAGPA	-1.0008864935272	4.93929292359748e-11

ENSG00000114686	MRPL3	-1.0012823346682	6.51961146702991e-57
ENSG00000140577	CRTC3	-1.00150518804643	8.18169118456239e-37
ENSG00000168275	COA6	-1.00193684314015	1.10885593420028e-24
ENSG00000214046	SMIM7	-1.00194006111294	1.10912212645757e-26
ENSG00000133773	CCDC59	-1.00210067356278	1.81951861128614e-24
ENSG00000149929	HIRIP3	-1.00252785764478	4.69620474817278e-17
ENSG00000261578	N.A.	-1.00297204135922	9.52194165431406e-06
ENSG00000146733	PSPH	-1.00319239959438	5.44262761200911e-38
ENSG00000126216	TUBGCP3	-1.00329162081468	4.08842496458553e-28
ENSG00000196576	PLXNB2	-1.00360074510034	2.43280581960897e-87
ENSG00000186501	TMEM222	-1.0039140437022	5.82765527152766e-26
ENSG00000145284	SCD5	-1.00398074596775	2.50994025492965e-27
ENSG00000104219	ZDHHC2	-1.00432672787047	3.48639539559957e-49
ENSG00000236088	COX10-AS1	-1.00457290295651	8.11599087794688e-11
ENSG00000181751	C5orf30	-1.00501301637365	2.82541035019641e-22
ENSG00000135245	HILPDA	-1.00515493031345	8.20498500131175e-28
ENSG00000005436	GCFC2	-1.00531094497547	5.48817127940245e-25
ENSG00000261556	SMG1P7	-1.00538447346396	0.00426965409872376
ENSG00000112893	MAN2A1	-1.00564216280533	3.17379737503379e-75
ENSG00000042980	ADAM28	-1.00593406107485	7.92147230704411e-11
ENSG00000151690	MFSD6	-1.00602768689525	2.1824460222143e-41
ENSG00000103326	CAPN15	-1.00707748164871	7.16170056059724e-43
ENSG00000105738	SIPA1L3	-1.00764664927609	3.85341027531021e-117
ENSG00000269713	NBPF9	-1.00782296948369	2.71811932734437e-22
ENSG00000189079	ARID2	-1.00791671816049	5.99206729553082e-50
ENSG00000106608	URGCP	-1.00833136705292	1.82765154183503e-28
ENSG00000257556	N.A.	-1.00850606230287	0.00615167456151706
ENSG00000100162	CENPM	-1.00853172602464	3.36991438711802e-17
ENSG00000131591	C1orf159	-1.00911853122584	6.61826619793102e-11
ENSG00000165338	HECTD2	-1.01005112206642	4.0824292173913e-16
ENSG00000125520	SLC2A4RG	-1.01011725401813	3.75652379355772e-43
ENSG00000180694	TMEM64	-1.01049952931816	3.35247308798329e-34
ENSG00000171604	CXXC5	-1.01095760680276	2.24709394438287e-30
ENSG00000119383	PPP2R4	-1.01285098844765	3.88023035304117e-74
ENSG00000186010	NDUFA13	-1.01288416741529	0.00129678891415818
ENSG00000101353	MROH8	-1.01294864262749	0.00649723326831646
ENSG00000226332	N.A.	-1.01299718264977	0.00154541939753981
ENSG00000144136	SLC20A1	-1.01372516402407	4.69824505585607e-59
ENSG00000184743	ATL3	-1.01400680112623	1.69353462968063e-62
ENSG00000212127	TAS2R14	-1.01442966469997	0.00304874942600428
ENSG00000138363	ATIC	-1.01454003887725	2.11550848192785e-85
ENSG00000171121	KCNMB3	-1.01463975385407	0.00489495099116978
ENSG00000112234	FBXL4	-1.01468824329016	7.54378664087111e-27
ENSG00000093217	XYLB	-1.01505275287881	1.55495998131312e-08
ENSG00000132953	XPO4	-1.0155442214821	5.467115435135399e-45
ENSG00000149792	MRPL49	-1.01604063142099	2.72085370008858e-46
ENSG00000105939	ZC3HAV1	-1.01646904994379	1.30869090110611e-92
ENSG00000204304	PBX2	-1.01711674329313	2.16482761070313e-21
ENSG00000048828	FAM120A	-1.01813960205204	6.74624883356187e-149

ENSG00000142396	ERVK3-1	-1.01850803530582	1.38105979958864e-23
ENSG00000066136	NFYC	-1.01865079324113	4.2475405417462e-27
ENSG00000206527	HACD2	-1.01866625330179	2.02469718522485e-82
ENSG00000132429	POPDC3	-1.0187076804909	2.23049804918472e-08
ENSG00000136463	TACO1	-1.01980529165572	1.41612208752248e-37
ENSG00000166012	TAF1D	-1.02053745115412	9.6822303744804e-46
ENSG00000166451	CENPN	-1.0212896877533	2.3754150467469e-52
ENSG00000129351	ILF3	-1.02167515019948	1.46004645824342e-169
ENSG00000054654	SYNE2	-1.02169715468466	5.41116862305162e-64
ENSG00000138794	CASP6	-1.0219071117475	3.36534367585161e-15
ENSG00000151240	DIP2C	-1.02213165514829	8.64457680481551e-36
ENSG00000259865	N.A.	-1.0226697966609	5.67172830651802e-06
ENSG00000173702	MUC13	-1.02273829858474	2.03735981900998e-113
ENSG00000231365	N.A.	-1.02277022880598	0.00105401021776347
ENSG00000178295	GEN1	-1.02284980235201	6.49345907371697e-38
ENSG00000151150	ANK3	-1.02374616452259	1.32448564462842e-26
ENSG00000073417	PDE8A	-1.02427699702668	9.96635558234605e-113
ENSG00000102038	SMARCA1	-1.02454488175246	7.44705245552266e-100
ENSG00000070814	TCOF1	-1.02486637152978	3.32381291516527e-69
ENSG00000162402	USP24	-1.02505028178734	1.50503669716283e-69
ENSG00000237187	NR2F1-AS1	-1.026067804174	5.6682689566352e-07
ENSG00000173068	BNC2	-1.02625550726677	2.24859782998793e-08
ENSG00000174791	RIN1	-1.02692939339951	7.11074335306757e-05
ENSG00000018189	RUFY3	-1.02706307970469	3.604370739121e-12
ENSG00000162777	DENND2D	-1.02788344543527	0.000665216924502714
ENSG00000163006	CCDC138	-1.02894966773243	1.14610782554265e-10
ENSG00000087586	AURKA	-1.02907719927287	2.24327020276246e-100
ENSG00000167969	ECI1	-1.02909425655431	1.485599076779e-34
ENSG00000254087	LYN	-1.03009137357277	1.1907603146534e-65
ENSG00000255583	N.A.	-1.03061884192161	0.00824199745397971
ENSG00000170485	NPAS2	-1.03146406504358	4.84772869264554e-73
ENSG00000138835	RGS3	-1.03187903836858	1.41213865497746e-24
ENSG00000185973	TMLHE	-1.032012742282	5.4376329222427e-14
ENSG00000137275	RIPK1	-1.0325388662181	1.06590234000989e-32
ENSG00000014138	POLA2	-1.03369705642662	2.48932798886845e-48
ENSG00000164163	ABCE1	-1.03465265894885	1.14317261193929e-79
ENSG00000086300	SNX10	-1.03477396088568	3.11150762683112e-13
ENSG00000079257	LXN	-1.03532486987728	1.07667926184509e-14
ENSG00000143013	LMO4	-1.03564504908002	3.44889531994738e-38
ENSG00000167118	URM1	-1.03714308123702	5.79923793980803e-39
ENSG00000197226	TBC1D9B	-1.03815032682007	4.65361874807499e-94
ENSG00000115053	NCL	-1.0383979991179	5.86048005498684e-187
ENSG00000105072	C19orf44	-1.03947655930303	1.93731269731771e-13
ENSG00000204946	ZNF783	-1.04002884323353	1.25702213970333e-10
ENSG00000221978	CCNL2	-1.04015327599671	2.84652071757959e-66
ENSG00000196305	IARS	-1.04023842547364	4.68932934670707e-186
ENSG00000109189	USP46	-1.04107744554712	2.27172011451629e-22
ENSG00000172071	EIF2AK3	-1.04123538193002	3.01958476725654e-28
ENSG00000172728	FUT10	-1.04232329406938	1.63090448996545e-15

ENSG00000136159	NUDT15	-1.0428351047126	1.54193151639575e-30
ENSG00000187630	DHRS4L2	-1.04332400018977	1.33286725225466e-09
ENSG00000171714	ANO5	-1.04488193636822	5.30117949939957e-34
ENSG00000130768	SMPDL3B	-1.0453536320799	2.85533389088946e-08
ENSG00000203709	C1orf132	-1.0457114868366	0.00010689055554499
ENSG00000172113	NME6	-1.04573623887624	3.22518083484848e-16
ENSG00000125901	MRPS26	-1.04609565919652	4.11642412216429e-50
ENSG00000196693	ZNF33B	-1.04696385643451	9.82666437131141e-16
ENSG00000183401	CCDC159	-1.04706816431662	9.95078831052334e-06
ENSG00000101574	METTL4	-1.04866401504185	2.16985724496708e-14
ENSG00000110693	SOX6	-1.04885446050975	6.07889237392807e-05
ENSG00000131116	ZNF428	-1.0493179254454	5.14108300888892e-20
ENSG00000137693	YAP1	-1.05008514541623	1.24660688624967e-85
ENSG00000226711	FAM66C	-1.05035196563407	4.36686001685216e-05
ENSG00000179913	B3GNT3	-1.05111952723674	5.96191320763995e-19
ENSG00000117751	PPP1R8	-1.05135167836316	1.90268822277556e-38
ENSG00000101447	FAM83D	-1.05146918963186	3.20664775643238e-75
ENSG00000185900	POMK	-1.0525148593976	3.36191056755382e-07
ENSG00000134852	CLOCK	-1.05281856005398	2.60844780217695e-41
ENSG00000089639	GMIP	-1.05303121043523	9.64095661816914e-10
ENSG00000163935	SFMBT1	-1.05330623136731	6.32701591009139e-17
ENSG00000184574	LPAR5	-1.05334600094136	0.00083903886925907
ENSG00000184564	SLITRK6	-1.05341028287551	0.000249428162006155
ENSG00000072840	EVC	-1.05377908876306	1.0396573197296e-28
ENSG00000087842	PIR	-1.05386329230808	4.26598805540898e-73
ENSG00000144161	ZC3H8	-1.05407343286428	1.475283058403e-12
ENSG00000168496	FEN1	-1.05418431364751	1.30388356323474e-91
ENSG00000062370	ZNF112	-1.05478509072867	4.14523981964465e-08
ENSG00000259120	SMIM6	-1.05485237346094	5.56110789034068e-10
ENSG00000170961	HAS2	-1.05495079428036	5.50544402725878e-05
ENSG00000083290	ULK2	-1.05496239503691	1.42002094885311e-58
ENSG00000198393	ZNF26	-1.05512105520577	2.74022329740859e-15
ENSG00000085999	RAD54L	-1.05610909195467	4.25689279972095e-16
ENSG00000196642	RABL6	-1.05646617851174	2.77445599355778e-20
ENSG00000037897	METTL1	-1.05700127691984	6.32669940236002e-17
ENSG00000148655	C10orf11	-1.0585801872708	0.00761284652305329
ENSG00000173621	LRFN4	-1.0590489125858	4.97527035481966e-21
ENSG00000161847	RAVER1	-1.05932348164919	6.02882699167655e-25
ENSG00000276234	TADA2A	-1.05942367396822	3.31602133141148e-25
ENSG00000223813	N.A.	-1.05948554692565	0.000577210849523327
ENSG00000124207	CSE1L	-1.05955539060095	5.67510366768961e-161
ENSG00000158286	RNF207	-1.05966442453422	1.46224310533304e-13
ENSG00000152782	PANK1	-1.05968977752467	1.01811944879551e-13
ENSG00000169045	HNRNPH1	-1.06004146108973	1.61043661089422e-203
ENSG00000088002	SULT2B1	-1.06020534709828	3.58389351625177e-09
ENSG00000111726	CMAS	-1.06022696851815	1.4633491468094e-61
ENSG00000101901	ALG13	-1.06043523790677	9.33191898887266e-36
ENSG00000149182	ARFGAP2	-1.0607767065669	8.587860296861e-41
ENSG00000136866	ZFP37	-1.06079287228069	0.000234960092656836

ENSG00000072756	TRNT1	-1.06253058269977	8.66493214022982e-27
ENSG00000158321	AUTS2	-1.06265099808203	9.21816189611262e-24
ENSG00000094804	CDC6	-1.06318049288857	7.14703360240156e-104
ENSG00000140416	TPM1	-1.06320225707352	5.68349949078361e-171
ENSG00000204839	MROH6	-1.06349848725265	4.97235917073851e-07
ENSG00000212232	SNORD17	-1.0636972137723	0.00654115671457248
ENSG00000100263	RHBDD3	-1.06453490861308	7.30698436139094e-20
ENSG00000104823	ECH1	-1.06454953785009	2.0695144326536e-67
ENSG00000114933	INO80D	-1.06460105714428	1.86470055060657e-18
ENSG00000100577	GSTZ1	-1.06472046483673	1.64433019056995e-29
ENSG00000133313	CNDP2	-1.06484593041479	5.36074587478609e-78
ENSG00000102796	DHRS12	-1.06487218201886	0.000750772225050818
ENSG00000177508	IRX3	-1.0649791408311	0.00260105660266189
ENSG00000266173	STRADA	-1.06502984584686	1.26959156939675e-06
ENSG00000196912	ANKRD36B	-1.06519164569005	2.45691676172362e-05
ENSG00000166582	CENPV	-1.06561282131472	8.30430243647661e-44
ENSG00000006125	AP2B1	-1.06575216774887	4.11584250602584e-204
ENSG00000196418	ZNF124	-1.06608749575342	3.03926461113789e-06
ENSG00000111319	SCNN1A	-1.06634646258187	6.92948760174055e-21
ENSG00000147130	ZMYM3	-1.06637036068568	1.24986606131311e-55
ENSG00000000971	CFH	-1.06706155676601	4.64030733958377e-127
ENSG00000269343	ZNF587B	-1.06735343193261	3.02113349633579e-14
ENSG00000046604	DSG2	-1.06766520510686	2.19461807452212e-147
ENSG00000107821	KAZALD1	-1.06788081793035	1.273201045954e-07
ENSG00000005243	COPZ2	-1.06929051215306	9.47317106914045e-11
ENSG00000109576	AADAT	-1.06948107745566	7.36994964753805e-13
ENSG00000116830	TTF2	-1.06975210013372	9.27577528833623e-47
ENSG00000107863	ARHGAP21	-1.06978133498301	9.08116951528291e-75
ENSG00000157833	GAREML	-1.07010234048255	4.4621018246453e-12
ENSG00000140400	MAN2C1	-1.07083852790376	2.50465578581784e-36
ENSG00000144579	CTDSP1	-1.07157064334774	6.9615264108894e-41
ENSG00000090372	STRN4	-1.07177768272911	4.16501985643562e-73
ENSG00000164078	MST1R	-1.07234685524208	1.64274323801811e-22
ENSG00000122482	ZNF644	-1.07272833095229	4.91408815098366e-39
ENSG00000101972	STAG2	-1.07316624892232	5.55638356491605e-85
ENSG00000168003	SLC3A2	-1.0733394213089	2.75007015383378e-171
ENSG00000164548	TRA2A	-1.07335332170923	3.11126648196003e-64
ENSG00000125089	SH3TC1	-1.07381890051089	2.47282401453625e-11
ENSG00000132694	ARHGEF11	-1.07391348500395	1.42056261298159e-62
ENSG00000119862	LGALSL	-1.07477806128618	1.9262816101039e-21
ENSG00000146263	MMS22L	-1.07512737771213	8.58716324230823e-34
ENSG00000133740	E2F5	-1.07526759089145	2.24409324349007e-15
ENSG00000176208	ATAD5	-1.07533476489865	1.1365547625266e-33
ENSG00000278600	N.A.	-1.07552973023063	3.00848939613669e-06
ENSG00000114405	C3orf14	-1.07571846138432	7.72645256906581e-10
ENSG00000143036	SLC44A3	-1.0757804406647	7.20804379580172e-13
ENSG00000113583	C5orf15	-1.07594621161883	6.78815413993152e-86
ENSG00000116898	MRPS15	-1.07596490814553	5.86374770672156e-50
ENSG00000137177	KIF13A	-1.07646304137892	1.91421315753043e-45

ENSG00000184719	RNLS	-1.07660076243695	1.28547297876992e-08
ENSG00000168502	MTCL1	-1.07679877982707	1.47610653497629e-129
ENSG00000115290	GRB14	-1.07736781938992	7.40732453588287e-22
ENSG00000065485	PDIA5	-1.07737187984581	1.41202987940165e-27
ENSG00000188895	MSL1	-1.07811293293923	6.8209524820213e-82
ENSG00000181619	GPR135	-1.07817759588107	2.46047765333689e-05
ENSG00000139180	NDUFA9	-1.07846083887069	3.53941396236634e-21
ENSG00000105388	CEACAM5	-1.07849091729906	3.66156402510441e-07
ENSG00000135314	KHDC1	-1.07956791927353	0.000966396134538643
ENSG00000105997	HOXA3	-1.0797221436872	3.47930388731167e-12
ENSG00000109814	UGDH	-1.08039027417059	7.53063509024316e-149
ENSG00000223749	MIR503HG	-1.08089161754488	5.58534707771758e-07
ENSG00000137504	CREBZF	-1.08115097883144	2.19404433780226e-73
ENSG00000142677	IL22RA1	-1.0816165649935	5.99344493421637e-06
ENSG00000105248	CCDC94	-1.08198921035896	8.68757052074615e-14
ENSG00000102870	ZNF629	-1.08199532133704	5.15777279992132e-43
ENSG00000256092	N.A.	-1.08353064551045	0.0001299337215158
ENSG00000070404	FSTL3	-1.08355018557395	7.63966451748601e-57
ENSG00000099956	SMARCB1	-1.08364583957155	4.26158388797229e-35
ENSG00000135842	FAM129A	-1.08364693748077	0.000163837271798582
ENSG00000129566	TEP1	-1.08376636781186	9.93288347628486e-40
ENSG00000116001	TIA1	-1.08418649423238	3.87506866056659e-69
ENSG00000167333	TRIM68	-1.08475727175104	6.62659607452042e-28
ENSG00000198189	HSD17B11	-1.08529813627376	4.02662938148769e-55
ENSG00000272335	N.A.	-1.0854165070112	4.48639047036258e-06
ENSG00000173320	STOX2	-1.08563810278803	1.5047662280986e-17
ENSG00000173041	ZNF680	-1.08578418620409	5.8768755202896e-11
ENSG00000093009	CDC45	-1.08587566572162	8.83161540709913e-34
ENSG00000171729	TMEM51	-1.08618447512393	1.35127458301293e-11
ENSG00000177106	EPS8L2	-1.08712204481849	6.99429518581951e-68
ENSG00000182324	KCNJ14	-1.08719215924363	0.000593224017570529
ENSG00000274471	N.A.	-1.08763510418404	0.000734492927926166
ENSG00000123908	AGO2	-1.08766921789514	3.40604466405589e-81
ENSG00000147100	SLC16A2	-1.08775510639806	9.08467805848887e-18
ENSG00000136824	SMC2	-1.08863298507844	1.63883995144922e-64
ENSG00000157510	AFAP1L1	-1.08930058553072	1.30179434644573e-06
ENSG00000243335	KCTD7	-1.08950928393963	9.70363529862512e-22
ENSG00000163528	CHCHD4	-1.08959670662754	4.20382653950324e-27
ENSG00000110422	HIPK3	-1.08978537073542	7.01816358117671e-74
ENSG00000152767	FARP1	-1.08993245760155	8.63234905981965e-38
ENSG00000212694	LINC01089	-1.09059429479763	1.39749382502247e-06
ENSG00000144455	SUMF1	-1.09070340321654	3.47216223199431e-24
ENSG00000178252	WDR6	-1.09086170226435	1.63299528783114e-60
ENSG00000139437	TCHP	-1.09134194801119	6.64175126889668e-35
ENSG00000141753	IGFBP4	-1.09148988969834	1.36741548848004e-172
ENSG00000130024	PHF10	-1.09199314629656	2.18560265459653e-34
ENSG00000188206	HNRNPU-AS1	-1.09260189120838	1.54467738589004e-16
ENSG00000165526	RPUSD4	-1.09348288021288	2.96462116942768e-18
ENSG00000148824	MTG1	-1.09406875055279	3.30152950274089e-08

ENSG00000198924	DCLRE1A	-1.09428367886979	9.83148608568962e-39
ENSG00000174130	TLR6	-1.09454229474519	4.81176368160566e-19
ENSG00000118777	ABCG2	-1.09457975174908	1.34777185135826e-61
ENSG00000241015	TPM3P9	-1.0946380280208	7.66170043695605e-24
ENSG00000158055	GRHL3	-1.09595663533355	9.2244767948416e-05
ENSG00000172183	ISG20	-1.09607868775031	5.78572758518269e-10
ENSG00000157881	PANK4	-1.09666719507876	1.64673420863724e-17
ENSG00000166833	NAV2	-1.09667713015267	3.87213998022904e-39
ENSG00000152219	ARL14EP	-1.09673661480974	1.1143301992495e-19
ENSG00000275023	MLLT6	-1.09703892824454	2.64735618650864e-68
ENSG00000071655	MBD3	-1.09712142805934	1.03333647693082e-48
ENSG00000126464	PRR12	-1.09745886827669	4.4145690432223e-51
ENSG00000112081	SRSF3	-1.09760834072554	2.82985897445554e-196
ENSG00000173482	PTPRM	-1.09812842367434	2.25101193967817e-144
ENSG00000188582	PAQR9	-1.09909480507736	4.55744145725957e-05
ENSG00000156052	GNAQ	-1.09974083889035	4.4536139956064e-70
ENSG00000132589	FLOT2	-1.10013072662915	4.39869136172894e-87
ENSG00000004660	CAMKK1	-1.10048514044852	8.81415152983276e-22
ENSG00000124225	PMEPA1	-1.10304472795215	1.56831669755841e-83
ENSG00000187772	LIN28B	-1.10483447623818	1.00788086956174e-13
ENSG00000239407	N.A.	-1.10509846482599	0.00117807837305244
ENSG00000185298	CCDC137	-1.10534929504037	2.19508131222853e-49
ENSG00000123485	HJURP	-1.10615737843412	3.66576909211607e-58
ENSG00000153944	MSI2	-1.10630189186002	2.6617281042925e-75
ENSG00000011422	PLAUR	-1.10677068108803	2.8090336691641e-16
ENSG00000138600	SPPL2A	-1.10691849388165	2.23562378404229e-66
ENSG00000083720	OXCT1	-1.10747917183287	0.000115964041722019
ENSG00000158427	TMSB15B	-1.10828268138351	0.00842415444456482
ENSG00000075461	CACNG4	-1.10854445534429	8.28634488516271e-08
ENSG00000154473	BUB3	-1.10897314992243	5.63183253591929e-150
ENSG00000109944	C11orf63	-1.10991760948491	2.8550356716783e-07
ENSG00000131051	RBM39	-1.11137017296244	9.54341571890912e-160
ENSG00000162517	PEF1	-1.11212145057501	2.12294316455892e-42
ENSG00000165501	LRR1	-1.11249765751161	1.30925225392575e-27
ENSG00000169057	MECP2	-1.11259466526939	1.82424650097197e-72
ENSG00000074416	MGLL	-1.11318893131954	7.0644318941254e-65
ENSG00000103227	LMF1	-1.11364183806619	5.12275616913337e-16
ENSG00000183840	GPR39	-1.11403669584439	8.25089134300771e-16
ENSG00000255517	N.A.	-1.11439758402707	2.70995603770227e-05
ENSG00000130545	CRB3	-1.11448577366905	3.76011320062616e-09
ENSG00000144642	RBMS3	-1.11489801385416	0.000207479324628476
ENSG00000137673	MMP7	-1.11491394864259	7.65392206853027e-41
ENSG00000131944	C19orf40	-1.11529951169029	2.45112916315395e-15
ENSG00000012817	KDM5D	-1.11582234484606	2.92834147234501e-33
ENSG00000235863	B3GALT4	-1.1162015704102	0.00826835552986822
ENSG00000184083	FAM120C	-1.11685479648817	1.86980319835675e-26
ENSG00000152804	HHEX	-1.11755897839052	5.34715342555001e-24
ENSG00000264230	ANXA8L1	-1.11767761696113	0.000190750135609377
ENSG00000110367	DDX6	-1.11884512186142	2.28105846274213e-101

ENSG00000004468	CD38	-1.11901452912401	3.64354324457575e-11
ENSG00000164031	DNAJB14	-1.11938977776832	1.76756873085082e-47
ENSG00000182903	ZNF721	-1.11970632393014	5.38129080494402e-16
ENSG00000114541	FRMD4B	-1.1221721310072	6.86554030802608e-20
ENSG00000159692	CTBP1	-1.12273057283517	3.20048180644077e-71
ENSG00000104824	HNRNPL	-1.12278670377205	3.7334941624326e-168
ENSG00000166681	NGFRAP1	-1.12364442109555	1.09635000194847e-82
ENSG00000159128	IFNGR2	-1.12391228596196	3.20208099844184e-62
ENSG00000159593	NAE1	-1.12406946024205	6.48825561876315e-82
ENSG00000260027	HOXB7	-1.12412658219588	5.0357378898875e-20
ENSG00000151692	RNF144A	-1.12424562353071	5.18357218157098e-23
ENSG00000176422	SPRYD4	-1.12427912715663	6.00040653298816e-18
ENSG00000185483	ROR1	-1.12460858103251	1.07886514566216e-26
ENSG00000273749	CYFIP1	-1.12475393944721	1.58760433090393e-176
ENSG00000229358	DPY19L1P1	-1.12475816004146	0.000123858457636999
ENSG00000282936	N.A.	-1.12545737634479	0.000891229644343518
ENSG00000176978	DPP7	-1.1258365668281	6.41170674044428e-38
ENSG00000148841	ITPRIP	-1.12650722027492	2.18764361840493e-26
ENSG00000077713	SLC25A43	-1.12651911316127	4.87067694096154e-47
ENSG00000152076	CCDC74B	-1.12678171858825	0.00025283461662946
ENSG00000018869	ZNF582	-1.12694176469734	0.0015847669854288
ENSG00000073008	PVR	-1.12703571747315	1.26507938594579e-122
ENSG00000113649	TCERG1	-1.12712942995849	3.13229996833344e-73
ENSG00000204147	ASAH2B	-1.12755480013555	7.01184418352761e-06
ENSG00000156521	TYSND1	-1.12763144982466	1.8694054859594e-39
ENSG00000172339	ALG14	-1.12763883703716	5.2268207008373e-13
ENSG00000164323	CFAP97	-1.12812245936557	2.79394470366187e-41
ENSG00000174446	SNAPC5	-1.12829332875236	1.3404703966194e-34
ENSG00000163393	SLC22A15	-1.12846791131136	0.000182218244486007
ENSG00000101003	GINS1	-1.12867913418893	3.45066765939537e-100
ENSG00000227036	LINC00511	-1.12943683583715	2.74692502003726e-30
ENSG00000042286	AIFM2	-1.12973719191906	1.97121720440422e-68
ENSG00000164306	PRIMPOL	-1.12974842637185	3.01997130746089e-15
ENSG00000204366	ZBTB12	-1.1297488230287	4.95900063310618e-07
ENSG00000145604	SKP2	-1.13003217933707	9.2599845146726e-35
ENSG00000223959	AFG3L1P	-1.13024683148893	3.70793345181707e-22
ENSG00000047365	ARAP2	-1.13074847933745	4.08919470558404e-12
ENSG00000160752	FDPS	-1.13110760795328	1.25597205115201e-169
ENSG00000100804	PSMB5	-1.13220425868704	1.27887474468865e-72
ENSG00000234616	JRK	-1.1322131789858	1.14075107046317e-37
ENSG00000104835	SARS2	-1.1329016156316	9.22324628896884e-09
ENSG00000182150	ERCC6L2	-1.13335476287638	4.9159223197583e-29
ENSG00000123219	CENPK	-1.13370748821457	2.04758940171975e-54
ENSG00000197747	S100A10	-1.13374753254841	3.54210295712339e-156
ENSG00000110852	CLEC2B	-1.13496541714886	0.000385855565403768
ENSG00000278950	N.A.	-1.13546877539853	0.00733387598471169
ENSG00000174501	ANKRD36C	-1.13550256369946	4.00386154382612e-07
ENSG00000152520	PAN3	-1.13555587531042	1.24684864515407e-29
ENSG00000139263	LRIG3	-1.13608675675356	6.18477218566159e-24

ENSG00000053372	MRTO4	-1.13615841684059	1.4548880665609e-54
ENSG00000102743	SLC25A15	-1.1363562430548	3.35448488853554e-12
ENSG00000176454	LPCAT4	-1.13662732766617	4.213761287467e-47
ENSG00000122965	RBM19	-1.13723012346379	2.50708608249063e-39
ENSG00000101940	WDR13	-1.13728653449059	1.73931612081251e-44
ENSG00000204116	CHIC1	-1.13759274911449	6.83961331763929e-28
ENSG00000275993	N.A.	-1.13773257655207	2.48287812187651e-39
ENSG00000163637	PRICKLE2	-1.13958190634527	1.43744650419341e-45
ENSG00000177602	GSG2	-1.13998329223571	4.50808783414016e-20
ENSG00000221926	TRIM16	-1.14063642547701	1.97834106881348e-65
ENSG00000168310	IRF2	-1.14128470359319	1.10796634645375e-19
ENSG00000116649	SRM	-1.14205092348155	9.60287061718809e-36
ENSG00000122566	HNRNPA2B1	-1.14211029621493	6.26647272187139e-203
ENSG00000169083	AR	-1.14328247096325	9.30863534493228e-57
ENSG00000164104	HMGB2	-1.14367122991957	8.49910289453082e-91
ENSG00000228223	HCG11	-1.14368044845702	1.82762598193001e-08
ENSG00000116791	CRYZ	-1.14385339724647	2.15357133998471e-77
ENSG00000005156	LIG3	-1.14388390110158	8.98151775953184e-73
ENSG00000184675	AMER1	-1.14408200252244	2.48980350631144e-14
ENSG00000257122	RRN3P3	-1.14461234214299	0.0010693644345505
ENSG00000173275	ZNF449	-1.14488572399454	5.59722937056745e-18
ENSG00000132825	PPP1R3D	-1.14500528943726	9.93583720333409e-13
ENSG00000125170	DOK4	-1.14502045027209	3.78322153250646e-102
ENSG00000100982	PCIF1	-1.14524357034185	3.80426219815268e-40
ENSG00000138411	HECW2	-1.14547209600674	4.18811411981018e-08
ENSG00000065268	WDR18	-1.14563524816119	1.20553690147726e-30
ENSG00000102699	PARP4	-1.14644567974639	3.30082680039507e-100
ENSG00000131943	C19orf12	-1.14696059095223	1.29502837517574e-48
ENSG00000232063	N.A.	-1.1477622933586	9.48427462961774e-06
ENSG00000099910	KLHL22	-1.14919240203385	2.48394307881396e-21
ENSG00000177084	POLE	-1.15049421230341	9.20947785722548e-111
ENSG00000182551	ADI1	-1.15068075583915	6.34159218134143e-57
ENSG00000128973	CLN6	-1.15074288137777	1.83193569084633e-63
ENSG00000111321	LTBR	-1.15096316146251	1.96127301728841e-105
ENSG00000106133	NSUN5P2	-1.15146887756928	1.66520650479308e-08
ENSG00000167513	CDT1	-1.15229752966227	4.75118474124485e-32
ENSG00000138092	CENPO	-1.15239511789379	2.27429598143846e-67
ENSG00000136379	ABHD17C	-1.15324229236347	3.59117073355004e-21
ENSG00000163507	KIAA1524	-1.15327905070984	1.16786561541832e-69
ENSG00000153551	CMTM7	-1.15444079992477	1.55714192536465e-14
ENSG00000166897	ELFN2	-1.15500082405804	3.27351996924275e-13
ENSG00000143258	USP21	-1.15612430418879	2.27937902940709e-22
ENSG00000258839	MC1R	-1.15681237237439	5.73146855756546e-07
ENSG00000143653	SCCPDH	-1.15686526352028	5.59063499066434e-76
ENSG00000171246	NPTX1	-1.15744029065754	3.79750095731409e-15
ENSG00000101158	NELFCD	-1.15765391705017	4.97042003041e-56
ENSG00000181027	FKRP	-1.15794381543675	1.49125275275115e-28
ENSG00000240429	LRRFIP1P1	-1.15844804584116	0.00450799786332558
ENSG00000008838	MED24	-1.1585390668096	2.90681954423784e-68

ENSG00000198039	ZNF273	-1.1590194624163	5.49444378627933e-09
ENSG00000188283	ZNF383	-1.15905181928197	9.27392933837058e-13
ENSG00000164124	TMEM144	-1.15928083859274	2.13012006218578e-29
ENSG00000135476	ESPL1	-1.16001108403712	9.66115137131594e-71
ENSG00000198952	SMG5	-1.16044007665276	1.17034439270114e-93
ENSG00000118620	ZNF430	-1.16060542097068	1.27506787550644e-08
ENSG00000262136	N.A.	-1.16062045260248	0.00981153509919065
ENSG00000197302	ZNF720	-1.16070801432426	6.58317147670385e-20
ENSG00000114805	PLCH1	-1.16081230987257	3.24168716368281e-64
ENSG00000163596	ICA1L	-1.16153321176683	3.68812854806762e-11
ENSG00000160360	GPSM1	-1.16154334915309	5.09008717311406e-10
ENSG00000158773	USF1	-1.16174935327418	1.16688274591165e-39
ENSG00000102048	ASB9	-1.16196025353898	1.64308218526339e-33
ENSG00000171208	NETO2	-1.16267713528511	9.23946292655328e-95
ENSG00000275183	LENG9	-1.16270173507288	3.05754657848429e-07
ENSG00000177103	DSCAML1	-1.16379180821308	1.10311224140689e-06
ENSG00000185220	PGBD2	-1.16450876761056	8.22233665394665e-09
ENSG00000146834	MEPCE	-1.16563429836889	7.94199360599359e-49
ENSG00000060688	SNRNP40	-1.16574813371503	1.59176949326087e-38
ENSG00000135750	KCNK1	-1.16643977600991	3.39365378982928e-36
ENSG00000176476	CCDC101	-1.16709104900203	5.30067749875962e-08
ENSG00000197324	LRP10	-1.16743278944769	8.61638738206019e-175
ENSG00000143436	MRPL9	-1.16760228650492	9.81172833227054e-50
ENSG00000128951	DUT	-1.16774745455161	4.6701098233552e-51
ENSG00000144724	PTPRG	-1.16786530649989	3.03874547775354e-19
ENSG00000124602	UNC5CL	-1.16787938633518	0.000255820160618602
ENSG00000124795	DEK	-1.16797469537677	5.12032660836531e-94
ENSG00000142661	MYOM3	-1.16826212207539	5.53086535921315e-11
ENSG00000155660	PDIA4	-1.1685659844156	5.15180098190254e-253
ENSG00000170190	SLC16A5	-1.16899425945321	2.5363655107006e-54
ENSG00000135966	TGFBRAP1	-1.1696144498702	6.17332140199865e-35
ENSG00000155561	NUP205	-1.16994607002536	4.49663976977103e-127
ENSG00000006282	SPATA20	-1.17031348755345	1.61470430507031e-91
ENSG00000183853	KIRREL	-1.17060202482996	6.28642590903327e-66
ENSG00000189180	ZNF33A	-1.17134323321536	2.58921602681979e-34
ENSG00000282826	N.A.	-1.17445186836757	1.43399977943569e-21
ENSG00000149636	DSN1	-1.1745423278143	1.73830572835194e-52
ENSG00000169752	NRG4	-1.17472653266917	1.55146213216519e-29
ENSG00000088367	EPB41L1	-1.17482842263958	2.47177650365546e-170
ENSG00000107669	ATE1	-1.17557898469893	7.92896251880084e-72
ENSG00000026103	FAS	-1.17614442000221	7.18157502027878e-12
ENSG00000196466	ZNF799	-1.17644125137609	0.000121887360884321
ENSG00000213853	EMP2	-1.1765750241992	5.88077195573425e-85
ENSG00000101004	NINL	-1.17690805945736	3.22665369649318e-34
ENSG00000104320	NBN	-1.17697602390077	5.86078734133347e-66
ENSG00000135537	LACE1	-1.17707287323121	2.87568175153588e-06
ENSG00000171206	TRIM8	-1.17752070198902	6.87853840226961e-43
ENSG00000204396	VWA7	-1.17762820427794	3.64176832829743e-07
ENSG00000270696	N.A.	-1.17775565346861	3.15693544287951e-15

ENSG00000102878	HSF4	-1.1780465709878	8.57555635440768e-07
ENSG00000239382	ALKBH6	-1.17809944838124	6.25856555510275e-07
ENSG00000139514	SLC7A1	-1.17817354326834	2.05697608514662e-105
ENSG00000132635	PCED1A	-1.17829571689249	2.79087024691332e-33
ENSG00000178919	FOXE1	-1.17907408317283	8.95432874900803e-07
ENSG00000084774	CAD	-1.17914514548298	1.6557385380577e-91
ENSG00000121068	TBX2	-1.1792683909255	2.06652150297149e-10
ENSG00000204348	DXO	-1.17936795572261	7.74238399842361e-09
ENSG00000231453	LINC01305	-1.17947104159707	0.000797654966816453
ENSG00000116679	IVNS1ABP	-1.17972600574056	8.8358658193811e-94
ENSG00000100941	PNN	-1.1797269259323	5.90999439468893e-104
ENSG00000269937	N.A.	-1.1797716995976	1.27621702071652e-05
ENSG00000073111	MCM2	-1.18015762024503	1.05928544779515e-92
ENSG00000133424	LARGE	-1.1802408363787	4.77752120418332e-38
ENSG00000259583	N.A.	-1.18033010402416	0.000360144424163326
ENSG00000177728	KIAA0195	-1.18071503176232	8.81708756852407e-61
ENSG00000198542	ITGBL1	-1.18091545900966	2.6240528316465e-10
ENSG00000166483	WEE1	-1.1813679919175	2.22357487887574e-48
ENSG00000164032	H2AFZ	-1.18238286034816	5.49154993817824e-145
ENSG00000154548	SRSF12	-1.18360880074015	0.000213947410364895
ENSG00000277161	PIGW	-1.18373982167008	8.62446811426715e-29
ENSG00000086717	PPEF1	-1.18513319360736	3.33850602633272e-07
ENSG00000270194	N.A.	-1.18515604221256	0.00956720188121086
ENSG00000278570	NR2E3	-1.18538361796237	0.00159032778636751
ENSG00000092853	CLSPN	-1.1859866151034	1.09260596634762e-36
ENSG00000066583	ISOC1	-1.1860118955574	3.9734842919713e-34
ENSG00000163071	SPATA18	-1.18602601605124	3.69640299823505e-05
ENSG00000250802	ZBED3-AS1	-1.18679767140854	0.0071370416556251
ENSG00000089127	OAS1	-1.18775731508998	1.23502537634268e-59
ENSG00000188611	ASAH2	-1.18776206746762	3.48330636708066e-05
ENSG00000142623	PADI1	-1.18843435691591	1.40186676657254e-07
ENSG00000163235	TGFA	-1.18852611343747	8.32755949883019e-28
ENSG00000184988	TMEM106A	-1.18876959070011	7.93769198573427e-05
ENSG00000213983	AP1G2	-1.18965065174721	8.46572699641503e-71
ENSG00000111602	TIMELESS	-1.18965401143083	1.58516411333933e-176
ENSG00000242110	AMACR	-1.19052870043541	9.45947761572183e-05
ENSG00000110628	SLC22A18	-1.19060455561319	1.37584502881029e-33
ENSG00000113441	LNPEP	-1.19162934399445	2.20228318996644e-73
ENSG00000112183	RBM24	-1.19170090866689	3.64507751158521e-25
ENSG00000161955	TNFSF13	-1.19219680889721	0.00439859308313743
ENSG00000111203	ITFG2	-1.19226046308287	9.05805082378226e-23
ENSG00000115687	PASK	-1.19353416941833	7.91179212691848e-35
ENSG00000267152	N.A.	-1.19373324116756	0.00756601526294727
ENSG00000099901	RANBP1	-1.19421350998896	4.1424070452658e-82
ENSG00000275234	N.A.	-1.19472423692678	8.85167674293149e-06
ENSG00000205041	N.A.	-1.19500381335598	0.00672850392817416
ENSG00000101846	STS	-1.19514575462437	2.48174533459284e-21
ENSG00000171658	N.A.	-1.19607313176542	1.35033721245083e-18
ENSG00000144395	CCDC150	-1.19611954806139	6.8485750932925e-09

ENSG00000177042	TMEM80	-1.19618917547112	4.14563129078774e-10
ENSG00000225210	DUXAP9	-1.19629841990737	3.23433957391183e-10
ENSG00000180921	FAM83H	-1.19636293912535	2.05964608033858e-69
ENSG00000100344	PNPLA3	-1.1968963231692	1.77053607071733e-10
ENSG00000184060	ADAP2	-1.19704280493977	1.65114829434779e-05
ENSG00000106258	CYP3A5	-1.19765190294089	1.55631113045105e-13
ENSG00000143179	UCK2	-1.19779081723755	2.24390290177797e-87
ENSG00000120068	HOXB8	-1.19795910220924	5.31967218250377e-22
ENSG00000073150	PANX2	-1.19858868069587	3.250853920619e-05
ENSG00000233016	SNHG7	-1.19911705515199	2.65620359775472e-40
ENSG00000160193	WDR4	-1.19955597928626	1.36038138383004e-16
ENSG00000172830	SSH3	-1.20083144140531	1.11023903993302e-42
ENSG00000109971	HSPA8	-1.20122961890687	0
ENSG00000109685	WHSC1	-1.20182417100409	8.96655770527854e-157
ENSG00000085840	ORC1	-1.2019609828208	2.84965215520741e-47
ENSG00000113140	SPARC	-1.20296512730252	0.00107496856781193
ENSG00000157036	EXOG	-1.20388328033419	4.51471000650177e-22
ENSG00000180155	LYNX1	-1.20448641188996	8.36164913859141e-08
ENSG00000011677	GABRA3	-1.20460051763394	5.76210053089337e-09
ENSG00000132359	RAP1GAP2	-1.20547959200814	8.48382739699429e-65
ENSG00000132424	PNISR	-1.20988013328563	1.53025643733344e-67
ENSG00000165238	WNK2	-1.21018024597335	2.13174704680784e-16
ENSG00000108691	CCL2	-1.21128934329842	0.000102248341427134
ENSG00000131153	GINS2	-1.21266652610332	6.06372852751716e-50
ENSG00000130810	PPAN	-1.21295586421219	0.00029916912872311
ENSG00000115641	FHL2	-1.21417478905208	1.07971320409108e-25
ENSG00000126091	ST3GAL3	-1.21533509110159	2.6223411827094e-06
ENSG00000214022	REPIN1	-1.21638911113487	2.50444554390491e-107
ENSG00000129173	E2F8	-1.21646143952578	7.12822177054906e-16
ENSG00000105127	AKAP8	-1.21833180133822	5.10913254944204e-32
ENSG00000254815	N.A.	-1.21929214607731	0.00569356208857595
ENSG00000072849	DERL2	-1.21945153338561	6.96781409950157e-87
ENSG00000049246	PER3	-1.21949412881782	2.12462619476807e-07
ENSG00000116793	PHTF1	-1.22005129056596	5.4333376640863e-36
ENSG00000085491	SLC25A24	-1.22063819630778	4.90855415100352e-62
ENSG00000131470	PSMC3IP	-1.22089719854442	1.85104857281386e-24
ENSG00000119777	TMEM214	-1.221242305525	1.61852243844252e-77
ENSG00000172465	TCEAL1	-1.22124457039933	3.50245859695014e-21
ENSG00000172594	SMPDL3A	-1.22134286038029	1.45645726022401e-06
ENSG00000119121	TRPM6	-1.22154428909628	8.20782636817821e-06
ENSG00000162738	VANGL2	-1.22192319510625	1.41866994818589e-05
ENSG00000076864	RAP1GAP	-1.22247592972569	2.89291481974818e-95
ENSG00000180071	ANKRD18A	-1.22274419664523	2.58435717722799e-18
ENSG00000146729	GBAS	-1.22329221515288	3.05693718926327e-85
ENSG00000112763	BTN2A1	-1.22498239826535	5.98800518319477e-36
ENSG00000176871	WSB2	-1.22531910324367	2.52737711404168e-135
ENSG00000166938	DIS3L	-1.22630704358579	7.44371118169456e-93
ENSG00000120802	TMPO	-1.22648946125975	5.0133160849021e-206
ENSG00000166741	NNMT	-1.22661576835824	1.45842725481055e-11

ENSG00000103485	QPRT	-1.22666882226379	5.04886627382917e-12
ENSG00000189339	SLC35E2B	-1.22692270557302	1.68963732386202e-62
ENSG00000271147	N.A.	-1.22811420823363	1.01648891943364e-05
ENSG00000129422	MTUS1	-1.22815116033357	6.06265672041532e-211
ENSG00000137628	DDX60	-1.22844586168071	8.22870916051752e-11
ENSG00000166479	TMX3	-1.22860441600458	2.52702770360956e-59
ENSG00000148834	GSTO1	-1.22883241712661	4.48774486814872e-81
ENSG00000225302	N.A.	-1.22908561118632	9.17354041326771e-07
ENSG00000168453	HR	-1.22909841273056	1.26202623212352e-35
ENSG00000227372	TP73-AS1	-1.2293043340457	3.98216106372235e-17
ENSG00000023902	PLEKHO1	-1.22956244852591	6.36070714426078e-06
ENSG00000223414	LINC00473	-1.22958639618268	2.9862605877092e-39
ENSG00000112701	SENP6	-1.2296496798452	5.86374770672156e-50
ENSG00000170522	ELOVL6	-1.229805070641	1.12940818074237e-81
ENSG00000109113	RAB34	-1.23005582685162	4.65282887005863e-78
ENSG00000183605	SFXN4	-1.23027134384583	1.21908954108384e-35
ENSG00000115526	CHST10	-1.2303501811276	5.91203503146988e-18
ENSG00000108773	KAT2A	-1.23087618386764	1.18682706254639e-57
ENSG00000081791	KIAA0141	-1.23187987490764	5.29833622036436e-60
ENSG00000173040	EVC2	-1.23251807143878	1.31428386741486e-08
ENSG00000142327	RNPEPL1	-1.23292857625067	4.72606305197442e-53
ENSG00000160209	PDXK	-1.23326016353131	9.58822615914168e-131
ENSG00000117481	NSUN4	-1.23370245151126	9.77460527599554e-27
ENSG00000118363	SPCS2	-1.23391527593863	6.44571898836498e-71
ENSG00000272763	N.A.	-1.23465364721299	1.52904349145259e-09
ENSG00000134986	NREP	-1.23513882489438	3.25919803975026e-16
ENSG00000253741	N.A.	-1.23695045762877	3.51874201760647e-05
ENSG00000267280	TBX2-AS1	-1.23770112259197	0.000968429402822974
ENSG00000165092	ALDH1A1	-1.23774096068686	5.65023301435803e-295
ENSG00000149571	KIRREL3	-1.23845599477197	2.08426046246324e-07
ENSG00000112679	DUSP22	-1.23853575150688	2.71331060956198e-20
ENSG00000172340	SUCLG2	-1.23878492977123	1.88480229217034e-84
ENSG00000112242	E2F3	-1.23901906596935	1.12683035174429e-42
ENSG00000172915	NBEA	-1.23963904717792	1.1819198229401e-101
ENSG00000223501	VPS52	-1.23984095823632	1.10127343786312e-31
ENSG00000103245	NARFL	-1.24011784286752	2.02700142308912e-30
ENSG00000004864	SLC25A13	-1.24029357697198	9.12371518774774e-78
ENSG00000130702	LAMA5	-1.24170568383519	4.71605867176343e-171
ENSG00000129317	PUS7L	-1.24240864585464	3.64702360632811e-46
ENSG00000101464	PIGU	-1.24380988437444	8.48507011164929e-51
ENSG00000115718	PROC	-1.24387426184648	6.22884144322674e-05
ENSG00000162522	KIAA1522	-1.24438135954636	4.22188935531 26e-113
ENSG00000171824	EXOSC10	-1.24456998792342	8.73525035407666e-74
ENSG00000136897	MRPL50	-1.24585117684375	2.68401945950502e-56
ENSG00000257315	ZBED6	-1.24595475743478	2.63631400907328e-23
ENSG00000187550	SBK2	-1.24749527269791	1.35393180123792e-11
ENSG00000114439	BBX	-1.24904761408602	1.84744097320135e-137
ENSG00000133195	SLC39A11	-1.24983214452573	4.3089957225126e-53
ENSG00000270055	N.A.	-1.25122888305728	1.53657128862656e-23

ENSG00000113597	TRAPPC13	-1.25140509228443	4.16941453503375e-39
ENSG00000178921	PFAS	-1.25147295066491	3.42405834819650e-87
ENSG00000263711	N.A.	-1.25159699741858	5.9540745836221e-65
ENSG00000139974	SLC38A6	-1.25210680694414	2.49470422934303e-23
ENSG00000137871	ZNF280D	-1.25214544515131	7.00481397971415e-51
ENSG00000254470	AP5B1	-1.252158701366	1.30315264391272e-26
ENSG00000269837	IPO5P1	-1.25243176807381	0.00194974201589729
ENSG00000163472	TMEM79	-1.25256238524147	1.90915272891237e-11
ENSG00000137135	ARHGEF39	-1.25320558662199	4.11306808502452e-33
ENSG00000280355	N.A.	-1.25374674317595	6.62542155048357e-07
ENSG00000146054	TRIM7	-1.25473558624893	2.373640887535e-10
ENSG00000137142	IGFBPL1	-1.25491417327449	1.05983411244114e-08
ENSG00000031081	ARHGAP31	-1.25531533276328	3.11110738255472e-07
ENSG00000198648	STK39	-1.25701814160848	1.32135970195645e-39
ENSG00000235687	LINC00993	-1.25770752598347	0.00218221453568226
ENSG00000271870	N.A.	-1.25899143853938	0.00436684697118482
ENSG00000155254	MARVELD1	-1.25989030628913	7.06514547899691e-42
ENSG00000147509	RGS20	-1.26045980419792	9.94998305211588e-13
ENSG00000136870	ZNF189	-1.26124553183874	2.28759832569796e-27
ENSG00000249042	N.A.	-1.2613955440583	3.65472425594857e-06
ENSG00000177311	ZBTB38	-1.2620199267567	1.09124279642423e-102
ENSG00000183628	DGCR6	-1.26233416431159	2.57656318537431e-11
ENSG00000141736	ERBB2	-1.26273749749142	9.83436893448951e-119
ENSG00000223705	NSUN5P1	-1.26314509452298	4.21440861326749e-19
ENSG00000163710	PCOLCE2	-1.26375066078389	5.68605171259622e-20
ENSG00000107104	KANK1	-1.26447808002913	1.14574160801964e-40
ENSG00000182141	ZNF708	-1.26524755608004	1.54471215252796e-06
ENSG00000111247	RAD51AP1	-1.26529233012638	3.22591200822732e-59
ENSG00000145362	ANK2	-1.26562935152089	1.40513747709069e-14
ENSG00000120694	HSPH1	-1.26573516159741	5.85937644325125e-153
ENSG00000121152	NCAPH	-1.26578232924721	6.74108946932243e-62
ENSG00000100650	SRSF5	-1.26628839275425	5.03839101727777e-139
ENSG00000168143	FAM83B	-1.26749610830334	0.00560792456874822
ENSG00000170364	SETMAR	-1.2675527801052	2.74985409003368e-13
ENSG00000187498	COL4A1	-1.26775382681945	1.24503200485005e-50
ENSG00000140044	JDP2	-1.26782036078457	3.37746895760766e-11
ENSG00000160200	CBS	-1.26875628512945	0.00765817161358812
ENSG00000138050	THUMPD2	-1.26910702129821	7.24040118228375e-21
ENSG00000100031	GGT1	-1.26918171685659	1.35043993776316e-41
ENSG00000143157	POGK	-1.26955053501564	5.90651680633463e-92
ENSG00000174586	ZNF497	-1.26960245054065	0.000452191710066148
ENSG00000185324	CDK10	-1.26985760668832	2.09656598924946e-42
ENSG00000131002	TXLNGY	-1.26999121315293	7.50390725839132e-54
ENSG00000232956	SNHG15	-1.27034578336238	4.44309505363138e-23
ENSG00000146267	FAXC	-1.27049823061894	5.86757531143473e-12
ENSG00000197372	ZNF675	-1.27074656893386	1.56503131337306e-10
ENSG00000099365	STX1B	-1.2707992594712	1.12593530681232e-06
ENSG00000267278	MAP3K14-AS1	-1.27091927487695	6.71221400207719e-09
ENSG00000123892	RAB38	-1.27132899274886	0.000148048518448143

ENSG00000134824	FADS2	-1.27169735108477	8.46653972661934e-189
ENSG00000236609	ZNF853	-1.27200613114518	0.000142854732088211
ENSG00000112425	EPM2A	-1.27232768513007	9.3867170674795e-08
ENSG00000107130	NCS1	-1.27238688507658	2.6360229767971e-66
ENSG00000145386	CCNA2	-1.27265406944208	1.15662416554383e-80
ENSG00000101337	TM9SF4	-1.27267465169608	3.4043041821898e-104
ENSG00000164418	GRIK2	-1.27565372157032	6.04262699378116e-29
ENSG00000272473	N.A.	-1.27587510856289	0.00719326465982517
ENSG00000118495	PLAGL1	-1.27595694903299	3.19641431422713e-18
ENSG00000184887	BTBD6	-1.27726266671734	7.99142865181539e-36
ENSG00000106333	PCOLCE	-1.27727175963221	3.29112474822745e-72
ENSG00000115112	TFCP2L1	-1.27832537052446	7.01004071363028e-46
ENSG00000214706	IFRD2	-1.27883555209841	1.03782098002357e-59
ENSG00000171889	MIR31HG	-1.27970652694615	1.07106624718175e-05
ENSG00000170439	METTL7B	-1.28080022442293	2.20784645402919e-38
ENSG00000272602	ZNF595	-1.28129210208045	2.35519305710709e-19
ENSG00000163923	RPL39L	-1.28168977021167	8.56084960065663e-31
ENSG00000177283	FZD8	-1.28194929334197	1.53828872693434e-12
ENSG00000138131	LOXL4	-1.28214635190862	3.48557707381921e-06
ENSG00000152689	RASGRP3	-1.28218899535895	0.000791813903293177
ENSG00000158717	RNF166	-1.28254391991704	2.08825096840036e-21
ENSG00000171792	RHNO1	-1.28330643718665	2.11045724399128e-88
ENSG00000198498	TMA16	-1.28399394344392	1.69520500424632e-27
ENSG00000117713	ARID1A	-1.28762164103833	1.82473336661643e-118
ENSG00000108557	RAI1	-1.2887311386801	3.90356731891559e-113
ENSG00000145996	CDKAL1	-1.2890023753611	1.84346741732614e-15
ENSG00000196730	DAPK1	-1.29002246895403	4.53528783817805e-156
ENSG00000130881	LRP3	-1.29033016266185	8.37100750294835e-45
ENSG00000148411	NACC2	-1.29076498969088	1.49492674407209e-101
ENSG00000124596	OARD1	-1.29082549841042	1.1934219640209e-31
ENSG00000204262	COL5A2	-1.29101983059405	1.42261482514116e-230
ENSG00000100479	POLE2	-1.29211606434485	2.13734713304376e-25
ENSG00000114021	NIT2	-1.29244839631798	7.02768362600178e-102
ENSG00000102317	RBM3	-1.29308235021702	2.11911006595132e-255
ENSG00000111206	FOXM1	-1.29427823241983	5.22765142582757e-141
ENSG00000167644	C19orf33	-1.29494779116929	1.17344482628852e-12
ENSG00000145779	TNFAIP8	-1.29562445675468	2.19965698223052e-47
ENSG00000075218	GTSE1	-1.29569523049867	7.08674427749775e-69
ENSG00000165476	REEP3	-1.2966845065777	2.99733378658739e-94
ENSG00000272398	CD24	-1.29725687617748	1.64612996580444e-127
ENSG00000109743	BST1	-1.29740225588182	2.34357776621899e-24
ENSG00000146409	SLC18B1	-1.29833093830845	9.7010318101719e-24
ENSG00000139910	NOVA1	-1.29845706267508	4.22066528496952e-22
ENSG00000181450	ZNF678	-1.29916332483885	1.89862438574645e-15
ENSG00000154978	VOPP1	-1.29926181857009	4.56295170857583e-124
ENSG00000164690	SHH	-1.29987872010616	0.000114042056833326
ENSG00000122481	RWDD3	-1.30007861508369	0.000150637788161889
ENSG00000196247	ZNF107	-1.30027361229504	6.22819200662019e-18
ENSG00000138119	MYOF	-1.30028235180872	1.0053836086937e-286

ENSG00000182979	MTA1	-1.30029838130476	9.05840260347726e-65
ENSG00000091947	TMEM101	-1.30037154571599	1.62770167634659e-26
ENSG00000079482	OPHN1	-1.30039339702772	5.27973805547078e-23
ENSG00000160818	GPATCH4	-1.3009206337808	1.14851085830656e-59
ENSG00000127423	AUNIP	-1.30105751144819	2.79936682324701e-10
ENSG00000131747	TOP2A	-1.30171406379891	2.30552244590465e-278
ENSG00000263528	IKBKE	-1.30205169375545	1.04756303335862e-16
ENSG00000164219	PGGT1B	-1.30242390447031	2.98180128531377e-47
ENSG00000051180	RAD51	-1.30299774121161	5.69101979847207e-20
ENSG00000187764	SEMA4D	-1.30312332307532	7.41160119798391e-32
ENSG00000167965	MLST8	-1.3034535960259	3.95997723404781e-40
ENSG00000134253	TRIM45	-1.30409186631151	3.25535938814997e-12
ENSG00000108424	KPNB1	-1.30452037103289	0
ENSG00000205664	N.A.	-1.3051560874338	2.48248439706247e-08
ENSG00000227954	TARID	-1.30528483204733	1.87538673821131e-07
ENSG00000127995	CASD1	-1.30621777452178	2.57322254655443e-50
ENSG00000198551	ZNF627	-1.30676068435102	1.74916024509084e-13
ENSG00000198833	UBE2J1	-1.30678022106075	4.72030429728585e-66
ENSG00000184441	N.A.	-1.30739800319461	0.00231282148659882
ENSG00000137393	RNF144B	-1.30773306928031	2.18288812566566e-05
ENSG00000213228	RPL12P38	-1.30805107898386	0.00667992249216907
ENSG00000130208	APOC1	-1.30830545251655	6.62052665857954e-15
ENSG00000100218	RSPH14	-1.30988944099088	0.00411695533885389
ENSG00000153246	PLA2R1	-1.31003159251131	1.25551551843839e-23
ENSG00000135722	FBXL8	-1.31013287542285	1.59653389913786e-05
ENSG00000100281	HMGXB4	-1.31033599638618	7.65834099626275e-47
ENSG00000156853	ZNF689	-1.3104234481506	1.35772220336899e-19
ENSG00000175711	B3GNTL1	-1.31133891590211	6.76957300004358e-09
ENSG00000119684	MLH3	-1.31252113945046	1.87438488700694e-56
ENSG00000140367	UBE2Q2	-1.31313111606808	1.58670293600761e-79
ENSG00000148719	DNAJB12	-1.31383777990297	2.17728685437853e-67
ENSG00000180855	ZNF443	-1.31396533207044	0.000288820720248153
ENSG00000124429	POF1B	-1.31444679961359	7.18435917287273e-58
ENSG00000166387	PPFIBP2	-1.31465762691045	2.10620698387353e-38
ENSG00000140263	SORD	-1.31467661627216	2.4299675984378e-38
ENSG00000223509	N.A.	-1.31516652148762	3.24378588767689e-07
ENSG00000155666	KDM8	-1.3154653309876	8.96419580561198e-06
ENSG00000136100	VPS36	-1.31576126923818	8.50926058331518e-68
ENSG00000274523	WBSCR16	-1.31582159546527	2.6454956372536e-65
ENSG00000166825	ANPEP	-1.31629607097993	1.46413394220572e-07
ENSG00000254469	N.A.	-1.31801735173697	0.000200065155590562
ENSG00000173218	VANGL1	-1.31902328599638	4.18789159499109e-74
ENSG00000178093	TSSK6	-1.32061059948087	0.00544648203406189
ENSG00000120699	EXOSC8	-1.32133573381597	1.08023260192574e-46
ENSG00000173757	STAT5B	-1.32264171803157	5.46171285489681e-75
ENSG00000174370	C11orf45	-1.32345690300512	6.01710332576413e-11
ENSG00000070614	NDST1	-1.32372490876497	6.09653814333149e-121
ENSG00000187605	TET3	-1.32395848773748	1.06433317869213e-49
ENSG00000166394	CYB5R2	-1.32471216223628	3.995379130806e-05

ENSG00000177045	SIX5	-1.32653620259749	3.07928154246173e-39
ENSG00000071539	TRIP13	-1.32666487090248	2.49665156230966e-85
ENSG00000234449	N.A.	-1.32679465010439	0.00298414541357658
ENSG00000136158	SPRY2	-1.32699598567389	3.87561064349926e-22
ENSG00000244560	N.A.	-1.32709734040323	0.00169943167426646
ENSG00000163625	WDFY3	-1.32711113481132	5.61696213885504e-79
ENSG00000093010	COMT	-1.32711194808912	2.20154627319635e-56
ENSG00000007968	E2F2	-1.3277057748037	3.65954549296209e-22
ENSG00000102547	CAB39L	-1.3280878784146	9.88408881395883e-07
ENSG00000175893	ZDHHC21	-1.32865316881816	3.10944103111156e-44
ENSG00000164402	SEPT8	-1.32914103340991	1.38762390084708e-85
ENSG00000154760	SLFN13	-1.33082178171977	5.43616268765377e-62
ENSG00000100139	MICALL1	-1.33135177324485	1.04512139169603e-30
ENSG00000196584	XRCC2	-1.33255645161403	2.02514624797186e-38
ENSG00000162368	CMPK1	-1.33263014367545	4.00378460931182e-126
ENSG00000063244	U2AF2	-1.3330917618008	4.60910878654694e-169
ENSG00000197429	IPP	-1.3344744375206	8.9804723663401e-22
ENSG00000177465	ACOT4	-1.33507226597185	1.69746460472837e-06
ENSG00000132386	SERPINF1	-1.33639016114808	2.5803837757361e-11
ENSG00000229921	KIF25-AS1	-1.33679738555042	3.43136519524908e-06
ENSG00000204967	PCDHA4	-1.33687594683219	2.08080737570411e-14
ENSG00000088881	EBF4	-1.33694212077144	1.47963142692275e-10
ENSG00000185379	RAD51D	-1.33694604930104	4.12504729181811e-41
ENSG00000218891	ZNF579	-1.33780541056145	1.80397005590114e-30
ENSG00000044459	CNTLN	-1.33829518132099	2.41015004451646e-37
ENSG00000156171	DRAM2	-1.33874547821225	3.37129600983867e-43
ENSG00000111906	HDDC2	-1.33917753498352	5.54127534745423e-58
ENSG00000141198	TOM1L1	-1.33960270530179	5.54153864032996e-114
ENSG00000166979	EVA1C	-1.33970480046435	9.1613774566086e-05
ENSG00000256802	N.A.	-1.34019619787895	4.01404268171346e-29
ENSG00000104129	DNAJC17	-1.34106009521691	8.22041482545612e-10
ENSG00000143418	CERS2	-1.34153638666793	3.2618252941184e-214
ENSG00000163125	RPRD2	-1.34291126098508	1.59238758153456e-77
ENSG00000162627	SNX7	-1.3431585825388	1.06395255336448e-44
ENSG00000116704	SLC35D1	-1.34397851087243	6.77733432825729e-48
ENSG00000189403	HMGB1	-1.34468098106682	4.46378817878082e-181
ENSG00000099337	KCNK6	-1.34479691716276	7.27994805921642e-13
ENSG00000187325	TAF9B	-1.34548453260269	3.39106979053783e-54
ENSG00000131778	CHD1L	-1.3471528634926	4.91363354897844e-89
ENSG00000197451	HNRNPAB	-1.34725748327136	2.85122546684337e-237
ENSG00000259295	CSPG4P12	-1.34770931403249	2.69930999399452e-05
ENSG00000112379	ARFGEF3	-1.34804135974374	1.49180959996155e-64
ENSG00000189362	TMEM194B	-1.3490731405479	9.92558187515134e-19
ENSG00000141258	SGSM2	-1.35014142898329	3.94690338463993e-65
ENSG00000151553	FAM160B1	-1.35045752344404	1.08191466799902e-57
ENSG00000144596	GRIP2	-1.35086000859966	0.00144724891420926
ENSG00000049239	H6PD	-1.35116771784143	1.95060449956006e-96
ENSG00000168970	JMJD7-PLA2G4B	-1.3514016321635	0.000569187764047264
ENSG00000137474	MYO7A	-1.35142455738014	2.0840870199744e-07

ENSG00000159921	GNE	-1.35225314682012	1.82666919031468e-117
ENSG00000167747	C19orf48	-1.35276817243959	1.59575899748941e-75
ENSG00000230148	HOXB-AS1	-1.35457394198773	0.000672270412504238
ENSG00000159063	ALG8	-1.35491851533118	4.88222065668372e-90
ENSG00000077684	JADE1	-1.35586940305244	2.42055595396952e-107
ENSG00000133119	RFC3	-1.35647390101485	1.34539503868198e-99
ENSG00000278530	CHMP1B2P	-1.35824215358978	1.90158486644209e-09
ENSG00000152284	TCF7L1	-1.35842923476753	2.87367818729315e-09
ENSG00000153064	BANK1	-1.3584678882933	8.27911281931214e-06
ENSG00000100395	L3MBTL2	-1.35864987310589	6.52241303598326e-42
ENSG00000142046	TMEM91	-1.35951676831762	1.91469498497383e-05
ENSG00000048342	CC2D2A	-1.35958255769617	4.35910699542715e-20
ENSG00000119673	ACOT2	-1.36021962362949	2.606436267801e-25
ENSG00000149292	TTC12	-1.36179752950424	1.04880131417556e-18
ENSG00000165716	FAM69B	-1.36285482754974	7.1650171830752e-53
ENSG00000236144	TMEM147-AS1	-1.36352488722288	1.89826657686386e-28
ENSG00000196954	CASP4	-1.36366999008229	8.35937275182185e-22
ENSG00000188130	MAPK12	-1.36443213426891	9.22411210002735e-22
ENSG00000244649	N.A.	-1.36451770643605	8.17151839230548e-16
ENSG00000092470	WDR76	-1.36476163569594	2.29500034351141e-45
ENSG00000175106	TVP23C	-1.36513704213335	4.56041939665039e-06
ENSG00000100473	COCH	-1.36578306109773	8.69472646223657e-82
ENSG00000133488	SEC14L4	-1.36582122188806	5.41150366503205e-17
ENSG00000065029	ZNF76	-1.36678893368395	3.48774440300552e-27
ENSG00000085465	OVGP1	-1.36723198677444	5.77778115133395e-07
ENSG00000126749	EMG1	-1.36914244787	9.22498079764452e-33
ENSG00000173638	SLC19A1	-1.36931405331328	3.11197910241858e-25
ENSG00000231925	TAPBP	-1.37272733192855	8.57923600488182e-108
ENSG00000171224	C10orf35	-1.3744840252962	1.04549953585335e-24
ENSG00000264112	N.A.	-1.37479631978126	2.89418761820876e-17
ENSG00000256304	CCDC150P1	-1.37541903305052	6.74906549501225e-06
ENSG00000140848	CPNE2	-1.37601098675137	2.74295813155475e-47
ENSG00000261061	N.A.	-1.37607597554285	9.73429202610562e-17
ENSG00000163808	KIF15	-1.37610010735715	3.355103704552e-46
ENSG00000160949	TONSL	-1.37694308302381	6.79629072832575e-44
ENSG00000168679	SLC16A4	-1.3771202346931	2.94728716939747e-80
ENSG00000263731	N.A.	-1.37741578760349	1.88559366818994e-05
ENSG00000280724	N.A.	-1.37764899340629	0.00585776021113267
ENSG00000132341	RAN	-1.37972750723646	0
ENSG00000184588	PDE4B	-1.38020207361369	2.97210057488364e-78
ENSG00000130508	PXDN	-1.38024598864336	1.84286571941151e-202
ENSG00000237357	N.A.	-1.38105213048344	0.00620372792714625
ENSG00000156535	CD109	-1.38157110454339	2.02787821576316e-208
ENSG00000024526	DEPDC1	-1.38174636306338	2.19135951080358e-80
ENSG00000169105	CHST14	-1.38210562875425	6.56284989453047e-24
ENSG00000090975	PITPNM2	-1.38221743790963	1.1717063667283e-26
ENSG00000175772	LINC01106	-1.38276727866392	0.0010408992052942
ENSG00000166250	CLMP	-1.38288525232501	4.30771323259183e-05
ENSG00000245556	SCAMP1-AS1	-1.38297186068954	2.13878951329532e-08

ENSG00000101255	TRIB3	-1.38311623028235	1.43567685288154e-126
ENSG00000165996	HACD1	-1.38353346184699	6.83485650716393e-25
ENSG00000091651	ORC6	-1.38447854374593	1.05375341820548e-60
ENSG00000086289	EPDR1	-1.38485992490657	2.37445779248435e-141
ENSG00000130052	STARD8	-1.38555045351523	1.13607516352628e-12
ENSG00000187676	B3GALTL	-1.38563096123771	5.43440232556505e-25
ENSG00000071051	NCK2	-1.38592119485081	4.91108004706329e-29
ENSG00000157326	DHRS4	-1.38685696566396	5.06834189315994e-20
ENSG00000263465	SRSF8	-1.3870610456261	5.70822415111874e-40
ENSG00000173698	ADGRG2	-1.38715048354696	3.61161015302484e-29
ENSG00000132846	ZBED3	-1.3881345056679	1.46506251986204e-44
ENSG00000103528	SYT17	-1.38858373169548	5.59844118751239e-20
ENSG00000241322	CDRT1	-1.39010287715948	0.000421365632775865
ENSG00000184860	SDR42E1	-1.39061386296865	4.02760507607054e-20
ENSG00000163781	TOPBP1	-1.39301910451603	1.93889917828609e-120
ENSG00000171492	LRRC8D	-1.39312275155846	3.24486846977601e-90
ENSG00000128408	RIBC2	-1.39367388145655	1.92065097829137e-06
ENSG00000143816	WNT9A	-1.39479082603441	9.6508497012575e-18
ENSG00000168769	TET2	-1.39547000254766	1.24256813911016e-36
ENSG00000140474	ULK3	-1.39794570307505	1.69894227515895e-59
ENSG00000174606	ANGEL2	-1.39817236897003	1.07347688316721e-58
ENSG00000158528	PPP1R9A	-1.39931042490552	6.25781531199102e-59
ENSG00000160602	NEK8	-1.3999733098105	1.5889572737101e-14
ENSG00000128191	DGCR8	-1.40017707152661	3.88193247926831e-54
ENSG00000234602	MCIDAS	-1.40034070733401	0.000482273663085384
ENSG00000139800	ZIC5	-1.40045756131871	2.94441507751094e-15
ENSG00000139178	C1RL	-1.4014107370946	1.06695851447237e-61
ENSG00000183684	ALYREF	-1.40148102859976	3.34448633421098e-78
ENSG00000132357	CARD6	-1.40222628363853	4.39257086249543e-18
ENSG00000147459	DOCK5	-1.40223242080854	6.15891644202641e-228
ENSG00000069974	RAB27A	-1.40229221418074	3.14316089401381e-25
ENSG00000258017	N.A.	-1.40401700052218	7.17859904761356e-05
ENSG00000168005	C11orf84	-1.4045955704057	3.38613991157778e-64
ENSG00000162545	CAMK2N1	-1.40562226462247	1.63749185500907e-47
ENSG00000108448	TRIM16L	-1.4057841370535	5.17455507862302e-146
ENSG00000106003	LFNG	-1.40657421207873	1.20767168059026e-39
ENSG00000256594	N.A.	-1.40762818431456	1.53828872693434e-12
ENSG00000127125	PPCS	-1.4090644743155	9.53051200853988e-55
ENSG00000279528	N.A.	-1.4096224464847	9.07972780896099e-07
ENSG00000103460	TOX3	-1.40970557242626	5.28695971557752e-46
ENSG00000154620	TMSB4Y	-1.41025672638173	0.00204103482519361
ENSG00000003402	CFLAR	-1.41106878318026	4.1348573215293e-81
ENSG00000238142	N.A.	-1.41174093664905	7.06234380315551e-06
ENSG00000142798	HSPG2	-1.41309727293149	9.95630042176087e-123
ENSG00000162614	NEXN	-1.41478280319256	5.64724634700887e-09
ENSG00000184162	NR2C2AP	-1.41480505093859	9.68289922107355e-24
ENSG00000115828	QPCT	-1.41519847752154	6.42835295059441e-95
ENSG00000077238	IL4R	-1.41545236256667	1.53772677824366e-34
ENSG00000170545	SMAGP	-1.4155494305551	5.67789295850091e-28

ENSG00000171617	ENC1	-1.41584946058925	1.19917556409942e-63
ENSG00000086475	SEPHS1	-1.41694388620389	3.10981584312744e-118
ENSG00000132478	UNK	-1.41695601490909	1.30307602220478e-80
ENSG00000103257	SLC7A5	-1.41735981937452	1.3641668927608e-293
ENSG00000166228	PCBD1	-1.41737193915188	2.00594655703851e-121
ENSG00000148840	PPRC1	-1.41777754232454	1.07592214851383e-89
ENSG00000079112	CDH17	-1.41821720371719	1.08863937462334e-61
ENSG00000262877	N.A.	-1.41988449263085	0.00125901880907252
ENSG00000166508	MCM7	-1.42020944629668	2.59342731874983e-235
ENSG00000178498	DTX3	-1.42046598886111	1.9761429156929e-34
ENSG00000169570	DTWD2	-1.42153462044306	1.20165724966873e-16
ENSG00000044090	CUL7	-1.42300188096154	9.89641371187566e-41
ENSG00000242498	ARPIN	-1.42323387520988	9.14894753082959e-113
ENSG00000278989	N.A.	-1.42340473682428	0.00193890183988197
ENSG00000169740	ZNF32	-1.42403866959976	5.84544708581649e-23
ENSG00000204271	SPIN3	-1.42425211610468	5.77361464602557e-11
ENSG00000129595	EPB41L4A	-1.42510491895804	1.48235013964047e-129
ENSG00000184489	PTP4A3	-1.4254832361619	5.7839498881504e-10
ENSG00000204767	FAM196B	-1.4260407302582	0.00207976980736833
ENSG00000162302	RPS6KA4	-1.42629570411214	8.19583825647504e-57
ENSG00000141002	TCF25	-1.42709110043047	4.31751242309575e-80
ENSG00000247982	LINC00926	-1.42721532951733	0.00714205250354777
ENSG00000151917	BEND6	-1.42726466375722	3.50153773198143e-05
ENSG00000139428	MMAB	-1.42780870529888	9.97803312950748e-107
ENSG00000107672	NSMCE4A	-1.42898025602314	1.00985513307573e-114
ENSG00000095539	SEMA4G	-1.42909909342055	4.58155641240821e-124
ENSG00000185149	NPY2R	-1.4292769431367	3.73102568879936e-11
ENSG00000142279	WTIP	-1.42963772588759	1.29683400433995e-28
ENSG00000152475	ZNF837	-1.43071515600658	0.00079867450604692
ENSG00000170412	GPRC5C	-1.43113712568714	5.62558236099308e-48
ENSG00000110237	ARHGEF17	-1.43159631357653	1.5041123526653e-44
ENSG00000121940	CLCC1	-1.43301791170015	8.89352690715851e-81
ENSG00000179409	GEMIN4	-1.43374462232371	1.99850297225815e-91
ENSG00000151353	TMEM18	-1.43453201068196	1.81956334462616e-42
ENSG00000198830	HMGN2	-1.43454340708931	3.38944349566256e-295
ENSG00000187134	AKR1C1	-1.43604089903311	3.07025584929912e-76
ENSG00000121058	COIL	-1.43628137120196	1.93519832051 75e-48
ENSG00000074964	ARHGEF10L	-1.43864708238481	6.54773673100917e-38
ENSG00000196227	FAM217B	-1.44062285504759	5.36183257641762e-32
ENSG00000126804	ZBTB1	-1.44121851013901	6.76150209515214e-45
ENSG00000131378	RFTN1	-1.44273430346322	8.37238714737515e-12
ENSG00000103202	NME4	-1.44369390822354	1.39099370844361e-107
ENSG00000233695	GAS6-AS1	-1.44500151252383	0.000116714735835668
ENSG00000175455	CCDC14	-1.44726758513449	3.84790223656269e-150
ENSG00000147202	DIAPH2	-1.4482747097509	1.48994254736676e-43
ENSG00000240053	LY6G5B	-1.44964431849955	0.00880149153270506
ENSG00000079387	SENP1	-1.44984278647034	2.47413295660003e-98
ENSG00000108179	PPIF	-1.44988890183996	1.44683212108264e-92
ENSG00000236287	ZBED5	-1.45094328041522	1.25716263029188e-64

ENSG00000120437	ACAT2	-1.45185053193338	5.25484155889245e-187
ENSG00000069011	PITX1	-1.45231753287695	1.54756802673139e-20
ENSG00000213047	DENND1B	-1.45371912198201	7.55995114072847e-37
ENSG00000128394	APOBEC3F	-1.45562221253898	6.01105593271653e-08
ENSG00000125257	ABCC4	-1.45647651538992	1.93669179935531e-204
ENSG00000115884	SDC1	-1.45855479322444	1.48158249004772e-172
ENSG00000060982	BCAT1	-1.45868589396747	1.48011141827293e-23
ENSG00000180891	CUEDC1	-1.45898421871556	3.59980883150627e-132
ENSG00000198586	TLK1	-1.4597560122254	2.52554541031697e-63
ENSG00000163378	EOGT	-1.46041756779502	1.36686372458452e-27
ENSG00000167604	NFKBID	-1.46256273721461	2.9299164762928e-08
ENSG00000139618	BRCA2	-1.46332080947885	3.00361266346298e-54
ENSG00000139116	KIF21A	-1.46457817451552	9.17558501794984e-108
ENSG00000100425	BRD1	-1.4663927660857	6.71442405456312e-40
ENSG00000166801	FAM111A	-1.46695606136923	1.55231113491075e-66
ENSG00000122778	KIAA1549	-1.4671251717095	5.6310956097946e-98
ENSG00000133475	GGT2	-1.46811644511023	0.00861441685708291
ENSG00000258474	N.A.	-1.4692369600663	2.74302452854935e-05
ENSG00000122863	CHST3	-1.46967426866757	1.58116369158314e-95
ENSG00000169174	PCSK9	-1.4706729851251	4.91773677851874e-175
ENSG00000196440	ARMCX4	-1.47247347370592	0.000244677916162712
ENSG00000131323	TRAF3	-1.47342019100176	1.70857490940452e-83
ENSG00000164466	SFXN1	-1.47343397330093	1.80650311330024e-150
ENSG00000163009	C2orf48	-1.47435865958278	0.00382867869023779
ENSG00000112293	GPLD1	-1.47641867295055	1.09740721805602e-05
ENSG00000022567	SLC45A4	-1.47645646177421	2.93190792901752e-90
ENSG00000146670	CDCA5	-1.47666636430315	6.19419236051082e-95
ENSG00000160226	C21orf2	-1.47729641227279	7.22460311737667e-12
ENSG00000176273	SLC35G1	-1.47746443254412	7.91513782880818e-35
ENSG00000230715	N.A.	-1.4775114386751	0.00093398357441622
ENSG00000137486	ARRB1	-1.47806203499802	3.97192126380016e-116
ENSG00000258818	RNASE4	-1.47871803392831	0.00564123920456867
ENSG00000107816	LZTS2	-1.47944285893617	1.17011049221527e-70
ENSG00000094916	CBX5	-1.48034024995666	1.34176028257341e-217
ENSG00000161547	SRSF2	-1.48064387621082	1.72864360011961e-300
ENSG00000089006	SNX5	-1.48070857457697	1.56080732669519e-179
ENSG00000162004	CCDC78	-1.4807747231377	0.00942632975840763
ENSG00000204264	PSMB8	-1.48204257084504	3.94623981398001e-22
ENSG00000269486	N.A.	-1.48251615427105	2.55904569965818e-13
ENSG00000174482	LINGO2	-1.48287394370931	0.00130447373821746
ENSG00000184271	POU6F1	-1.48294614071646	6.70608714673724e-06
ENSG00000121211	MND1	-1.48311598862756	3.96697526362463e-16
ENSG00000004866	ST7	-1.48325343229698	1.82956697426517e-44
ENSG00000112624	GLTSCR1L	-1.4845467633326	8.34748318445109e-31
ENSG00000100201	DDX17	-1.48686545604282	0
ENSG00000199753	SNORD104	-1.48699138568799	2.80281657392223e-05
ENSG00000166828	SCNN1G	-1.4871259146228	0.00267875129674325
ENSG00000101945	SUV39H1	-1.48838198637402	3.22677666349326e-51
ENSG00000172534	HCFC1	-1.48936246719177	7.76164549718967e-156

ENSG00000144504	ANKMY1	-1.48988444264374	1.38605118067428e-16
ENSG00000278619	MRM1	-1.49044751245535	4.99956749732035e-30
ENSG00000132849	INADL	-1.49328976415144	2.65665316176605e-79
ENSG00000140939	NOL3	-1.49390625095043	9.23856857256924e-32
ENSG00000227038	GTF2IP7	-1.49455949245698	2.73033877850245e-05
ENSG00000173207	CKS1B	-1.49460474895522	8.04385531745295e-142
ENSG00000233175	N.A.	-1.49476635534131	3.05673913317471e-07
ENSG00000149554	CHEK1	-1.49572685588115	1.12202416358962e-77
ENSG00000126790	L3HYPDH	-1.49638355457284	1.26870603504538e-42
ENSG00000178752	FAM132B	-1.49641314090283	4.29531707213449e-06
ENSG00000176720	BOK	-1.49679123008191	7.15383354981791e-18
ENSG00000114993	RTKN	-1.49759820891811	5.0019414952866e-30
ENSG00000198081	ZBTB14	-1.49802791183832	2.61656722277009e-27
ENSG00000197461	PDGFA	-1.49864329825176	1.88633742728218e-22
ENSG00000079739	PGM1	-1.49951722432098	1.52316436100748e-97
ENSG00000204176	SYT15	-1.50035793020435	0.000216300723885573
ENSG00000181031	RPH3AL	-1.50190849218961	4.5155913284969e-67
ENSG00000154237	LRRK1	-1.50263637539502	2.14793511924082e-66
ENSG00000180902	D2HGDH	-1.50340735773903	9.10783974856631e-16
ENSG00000161179	YDJC	-1.50469182287595	5.12730993076072e-34
ENSG00000183780	SLC35F3	-1.50572299332891	5.81694398613646e-20
ENSG00000184378	ACTRT3	-1.50595234437546	4.63513273551579e-05
ENSG00000074047	GLI2	-1.50691166271436	1.69212712845117e-37
ENSG00000235884	LINC00941	-1.50954245576754	0.000901698861333399
ENSG00000178401	DNAJC22	-1.51122368802327	7.45516088888019e-133
ENSG00000077514	POLD3	-1.51234205799258	5.04244656088416e-58
ENSG00000196968	FUT11	-1.51255600102441	5.87794319904571e-35
ENSG00000164930	FZD6	-1.51401186069505	1.13637456764826e-109
ENSG00000204531	POU5F1	-1.51460287882101	0.00162984792696783
ENSG00000105643	ARRDC2	-1.51608992506601	5.73354741198309e-57
ENSG00000183723	CMTM4	-1.51670531231608	5.69351920767675e-203
ENSG00000125741	OPA3	-1.51691168902477	5.2801590921615e-84
ENSG00000172244	C5orf34	-1.51894893874845	1.48633429011718e-22
ENSG00000145391	SETD7	-1.51907113687756	7.67197615173881e-173
ENSG00000171227	TMEM37	-1.51959461084611	2.77145089478042e-05
ENSG00000197813	N.A.	-1.5224357117535	0.000621666147325426
ENSG00000182095	TNRC18	-1.52247515255153	1.27001578862197e-166
ENSG00000083454	P2RX5	-1.52274830388343	0.00584893068761844
ENSG00000162341	TPCN2	-1.52295112771212	1.40869066876295e-39
ENSG00000014914	MTMR11	-1.5247234468001	2.72129491078861e-79
ENSG00000188385	JAKMIP3	-1.52726020396142	1.53953100738861e-14
ENSG00000120616	EPC1	-1.52783035316705	2.70535996850498e-39
ENSG00000196081	ZNF724P	-1.527885952115	0.000255688323140902
ENSG00000122861	PLAU	-1.52966592757698	1.86673366858743e-67
ENSG00000170962	PDGFD	-1.5301811280508	2.33852707762956e-81
ENSG00000123600	METTL8	-1.53022846758162	1.06831215497092e-31
ENSG00000245105	A2M-AS1	-1.53170013119179	0.00102227246168126
ENSG00000103544	C16orf62	-1.53179532595879	3.7785397270148e-113
ENSG00000014216	CAPN1	-1.5321343632328	5.22495481089666e-188

ENSG00000136982	DSCC1	-1.53248041652459	1.54343197350202e-30
ENSG00000135373	EHF	-1.53345366085126	6.01673070093819e-06
ENSG00000138759	FRAS1	-1.53360736154593	1.70406731163337e-167
ENSG00000149639	SOGA1	-1.5338448523717	7.19944574199978e-142
ENSG00000229970	N.A.	-1.53386731442492	0.0080870046941917
ENSG00000184227	ACOT1	-1.53438106009194	2.23224610407696e-12
ENSG00000197321	SVIL	-1.53540642478582	1.92490468154908e-117
ENSG00000118508	RAB32	-1.53590789328484	1.17468587182567e-42
ENSG00000175806	MSRA	-1.53624237162292	4.6918577498345e-20
ENSG00000198176	TFDP1	-1.53803979171607	8.46744682170071e-156
ENSG00000175274	TP53I11	-1.53838257023416	2.75721137963112e-174
ENSG00000237857	N.A.	-1.53891350436588	0.000166954779767728
ENSG00000162623	TYW3	-1.53930039658394	2.55816679726692e-47
ENSG00000178150	ZNF114	-1.53980406221195	2.73158155025574e-07
ENSG00000166546	BEAN1	-1.54094674870756	0.000189028613553367
ENSG00000145743	FBXL17	-1.54122722618235	3.61314258591214e-35
ENSG00000106610	STAG3L4	-1.54149236875879	2.11627117671535e-26
ENSG00000164695	CHMP4C	-1.54281683119639	3.37872246272211e-30
ENSG00000235109	ZSCAN31	-1.54318930899105	8.59332736743829e-07
ENSG00000176834	VSIG10	-1.54319625412828	9.10953267457686e-74
ENSG00000137834	SMAD6	-1.54347001312464	9.84843853136196e-129
ENSG00000152990	ADGRA3	-1.54371577634351	5.77874603052864e-59
ENSG00000165675	ENOX2	-1.54451636173504	8.89215651959255e-42
ENSG00000143409	FAM63A	-1.54503837089174	2.95782583236397e-16
ENSG00000131781	FMO5	-1.54574043603072	4.30790528029048e-11
ENSG00000181472	ZBTB2	-1.54648213791417	6.64436068212611e-31
ENSG00000184669	OR7E14P	-1.54745998137172	1.4163321003372e-05
ENSG00000156876	SASS6	-1.5475447333725	8.80701041637405e-36
ENSG00000178764	ZHX2	-1.5494642897923	1.48329967110535e-12
ENSG00000100003	SEC14L2	-1.5510572290182	2.44976803722427e-10
ENSG00000166503	N.A.	-1.55224655523306	2.85967375578646e-109
ENSG00000214274	ANG	-1.55227655223591	1.9466268490496e-37
ENSG00000065325	GLP2R	-1.55229314248848	2.68980955374866e-11
ENSG00000103742	IGDCC4	-1.55273374237312	2.96591843880851e-19
ENSG00000152240	HAUS1	-1.55304549428325	1.76558001637769e-49
ENSG00000196739	COL27A1	-1.5533561961512	1.51246596613347e-44
ENSG00000205763	RP9P	-1.55592072737704	2.36604496073768e-09
ENSG00000132481	TRIM47	-1.5561664683707	4.36459839240085e-79
ENSG00000150687	PRSS23	-1.55659645783164	0
ENSG00000248874	C5orf17	-1.55733479060896	0.00545390397746919
ENSG00000136193	SCRN1	-1.55746831262215	0
ENSG00000102181	CD99L2	-1.55762127668654	7.10458350129695e-214
ENSG00000146038	DCDC2	-1.55906225316318	7.9132487558324e-98
ENSG00000145476	CYP4V2	-1.55908516808752	8.0939180616004e-18
ENSG00000142733	MAP3K6	-1.55925171736266	1.26886269012118e-22
ENSG00000160447	PKN3	-1.56009824852957	6.15728569178902e-48
ENSG00000188738	FSIP2	-1.56276129747199	0.00817097629198517
ENSG00000101997	CCDC22	-1.56293564869962	4.19495013607074e-31
ENSG00000107719	PALD1	-1.56334727349051	7.62783646086433e-14

ENSG00000117569	PTBP2	-1.56387445663783	6.68488026496782e-41
ENSG00000083544	TDRD3	-1.56394145923556	2.98546479352574e-26
ENSG00000279041	N.A.	-1.56424292678421	1.00811444731e-08
ENSG00000169031	COL4A3	-1.5644159711481	1.20763749686047e-18
ENSG00000161888	SPC24	-1.56450859183324	7.70850992197663e-45
ENSG00000112667	DNPH1	-1.56505623034108	3.89736181680889e-43
ENSG00000142731	PLK4	-1.56547457341411	3.68041059141039e-62
ENSG00000244701	N.A.	-1.56549393936338	0.00064771278126732
ENSG00000231113	N.A.	-1.56556466269165	0.00175481712541795
ENSG00000140451	PIF1	-1.56602600521916	1.75124348089994e-34
ENSG00000215068	N.A.	-1.56792519248789	3.17290374086563e-09
ENSG00000128833	MYO5C	-1.56816557593537	4.68111700290456e-178
ENSG00000179698	KIAA1875	-1.56866322059349	9.37725646974235e-05
ENSG00000115159	GPD2	-1.56896739128795	4.16208410879506e-136
ENSG00000187720	THSD4	-1.56953093798315	9.2264617125351e-237
ENSG00000182858	ALG12	-1.56989511049749	2.08799073412057e-40
ENSG00000248905	FMN1	-1.57070176558855	2.26056958725206e-58
ENSG00000172296	SPTLC3	-1.57205974775555	3.94648670616627e-40
ENSG00000160124	CCDC58	-1.57269996398143	9.87154259117012e-32
ENSG00000171408	PDE7B	-1.57524036715377	8.61899316991757e-08
ENSG00000132182	NUP210	-1.57638893671816	4.63240229274804e-204
ENSG00000144741	SLC25A26	-1.57856185775074	6.91026787480924e-23
ENSG00000099849	RASSF7	-1.5789837363319	2.02608373672787e-73
ENSG00000111328	CDK2AP1	-1.58187637700626	3.38933964065824e-89
ENSG00000260552	N.A.	-1.58239699412022	0.00184375534261762
ENSG00000187583	PLEKHN1	-1.58356813264205	0.000926595041221231
ENSG00000011028	MRC2	-1.58394954078481	2.50257250022964e-146
ENSG00000104738	MCM4	-1.58678283083009	0
ENSG00000250903	GMDS-AS1	-1.59173644747315	1.25117655074503e-06
ENSG00000164308	ERAP2	-1.59235177011438	3.16512316412943e-07
ENSG00000196975	ANXA4	-1.59264622108501	0
ENSG00000250899	N.A.	-1.5929187317865	2.26323332057616e-24
ENSG00000138031	ADCY3	-1.59431158286071	1.5010492808778e-107
ENSG00000198680	TUSC1	-1.59445027118683	1.1722224358254e-19
ENSG00000134762	DSC3	-1.59556491771591	9.3491248936391e-19
ENSG00000270012	N.A.	-1.59661720829001	5.82617715503808e-05
ENSG00000028116	VRK2	-1.59794039556443	3.68283316320733e-59
ENSG00000205426	KRT81	-1.59905853445739	2.14764032156931e-280
ENSG00000165028	NIPSNAP3B	-1.60129202602271	3.8801690743189e-05
ENSG00000072135	PTPN18	-1.6014284635599	1.86477665273469e-42
ENSG00000156509	FBXO43	-1.6016239942912	1.14540671824388e-11
ENSG00000095752	IL11	-1.60175842807721	1.21622825609841e-07
ENSG00000007372	PAX6	-1.60238682147724	1.15042397256946e-06
ENSG00000091640	SPAG7	-1.60476510005458	1.69341380790466e-60
ENSG00000115841	RMDN2	-1.60482974356035	1.90722834729319e-09
ENSG00000171234	UGT2B7	-1.60488040886587	0.00594658371328679
ENSG00000135540	NHSL1	-1.60565962638246	4.37239720161031e-21
ENSG00000204860	FAM201A	-1.60720042555851	2.42765228842083e-17
ENSG00000065413	ANKRD44	-1.60785635843257	7.76371785122134e-12

ENSG00000078487	ZCWPW1	-1.60920949481982	5.2788598912793e-05
ENSG00000065833	ME1	-1.60989687190363	4.06527078404685e-170
ENSG00000010803	SCMH1	-1.61017946567213	2.33901005899259e-42
ENSG00000170275	CRTAP	-1.61072218563286	0
ENSG00000103335	PIEZO1	-1.61088834856882	7.65181609986295e-131
ENSG00000135821	GLUL	-1.61109751583624	1.0051250872292e-130
ENSG00000063601	MTMR1	-1.61229416401507	1.66341685085602e-85
ENSG00000055130	CUL1	-1.61246139107449	7.87741889187694e-156
ENSG00000253552	HOXA-AS2	-1.61328707496677	5.61716112305992e-08
ENSG00000111801	BTN3A3	-1.61391566950619	1.30050348280601e-06
ENSG00000272325	NUDT3	-1.61496274647896	5.46589118250911e-85
ENSG00000186684	CYP27C1	-1.61845194441362	0.000480258273318292
ENSG00000182109	N.A.	-1.61863398407773	7.42443012264896e-05
ENSG00000142686	C1orf216	-1.61885238295704	2.07421741925192e-29
ENSG00000169871	TRIM56	-1.61914158785149	6.96137490846354e-101
ENSG00000246228	CASC8	-1.6207279036276	0.004778859492187
ENSG00000168237	GLYCTK	-1.62106965533214	3.71147182076393e-24
ENSG00000110321	EIF4G2	-1.62215769885694	0
ENSG00000257151	PWAR6	-1.62323372297782	1.31201861413914e-08
ENSG00000049618	ARID1B	-1.62441556513095	7.78899164403504e-98
ENSG00000138074	SLC5A6	-1.62532205791416	2.80983121608613e-67
ENSG00000119227	PIGZ	-1.62646297503736	0.000806867282309049
ENSG00000186787	SPIN2B	-1.62679542167473	9.2212767187206e-06
ENSG00000145012	LPP	-1.62697297304565	3.04017749066783e-61
ENSG00000151208	DLG5	-1.62797667834539	2.40828049607968e-159
ENSG00000123636	BAZ2B	-1.62836727255971	5.36852480565659e-69
ENSG00000091490	SEL1L3	-1.62901291605366	2.18197604009273e-104
ENSG00000163072	NOSTRIN	-1.62909030311008	1.64835034191034e-14
ENSG00000147316	MCPH1	-1.63074674823678	1.15058997308199e-42
ENSG00000169908	TM4SF1	-1.63117590993056	1.4475376195395e-253
ENSG00000135632	SMYD5	-1.63173968101712	2.25411436596344e-78
ENSG00000134330	IAH1	-1.63243288772236	1.86720002433101e-70
ENSG00000148848	ADAM12	-1.63359956381711	0.000167031386067494
ENSG00000188643	S100A16	-1.63391499115308	3.36183345083076e-139
ENSG00000262903	N.A.	-1.63644889140279	7.30243845591184e-06
ENSG00000160703	NLRX1	-1.63665238892362	1.55110050287518e-39
ENSG00000154822	PLCL2	-1.63805765837489	3.76005970878811e-42
ENSG00000176014	TUBB6	-1.63990413439464	3.39579582933344e-142
ENSG00000153914	SREK1	-1.64010834947052	2.18305180201388e-121
ENSG00000136542	GALNT5	-1.64211354902704	3.06053395866875e-48
ENSG00000164038	SLC9B2	-1.64245046654489	2.12491078907955e-13
ENSG00000235961	PNMA6A	-1.64310219719106	0.00109888274487622
ENSG00000184470	TXNRD2	-1.64449427771701	1.09215488824021e-38
ENSG00000154102	C16orf74	-1.64488081965409	4.45904148768839e-07
ENSG00000151687	ANKAR	-1.64490154540124	0.000295333798953071
ENSG00000100906	NFKBIA	-1.6457147524146	2.24200016240602e-69
ENSG00000250538	N.A.	-1.64596444352361	1.19491927213161e-05
ENSG00000108984	MAP2K6	-1.64637279747041	5.04623482651049e-83
ENSG00000114638	UPK1B	-1.64961727010659	0

ENSG00000136783	NIPSNAP3A	-1.65015100980385	9.38445765432996e-40
ENSG00000140961	OSGIN1	-1.65041552025052	5.14946740783576e-92
ENSG00000147274	RBMX	-1.650552642703	0
ENSG00000179051	RCC2	-1.6538423584507	9.53787197273492e-194
ENSG00000119946	CNNM1	-1.65457310709491	2.04777219419811e-87
ENSG00000100478	AP4S1	-1.65521213951147	7.25509398277881e-32
ENSG00000076356	PLXNA2	-1.6552355880123	1.15401537114609e-48
ENSG00000109586	GALNT7	-1.65653429358657	1.24141431403224e-179
ENSG00000259699	HMGB1P8	-1.65800460102612	0.00974410804403219
ENSG00000153822	KCNJ16	-1.65916757848947	1.49148938772769e-06
ENSG00000269190	FBXO17	-1.65935681925611	3.05268723791981e-93
ENSG00000138336	TET1	-1.65995567153894	1.59112860100296e-14
ENSG00000273706	LHX1	-1.66057544312858	5.54386257091442e-05
ENSG00000166965	RCCD1	-1.66107978561021	9.07263140606652e-79
ENSG00000161618	ALDH16A1	-1.66556534495235	7.42032097105587e-44
ENSG00000035115	SH3YL1	-1.66695368417917	2.29030061522279e-11
ENSG00000152518	ZFP36L2	-1.66717664112269	1.88125613174797e-90
ENSG00000185442	FAM174B	-1.66757732772388	1.17076006817779e-28
ENSG00000082781	ITGB5	-1.67021627068435	0
ENSG00000276043	UHRF1	-1.67090908980589	6.2045630014688e-110
ENSG00000175536	LIPT2	-1.67098592993824	3.10745805318483e-05
ENSG00000102452	NALCN	-1.67168592249188	1.20584282998014e-24
ENSG00000221963	APOL6	-1.6743596613297	3.39404647202391e-43
ENSG00000111371	SLC38A1	-1.6755118968727	0
ENSG00000023697	DERA	-1.67595446753666	5.49988431381578e-134
ENSG00000132199	ENOSF1	-1.67607496582058	4.05546829095288e-75
ENSG00000127415	IDUA	-1.67619357881156	1.24160844182376e-13
ENSG00000076248	UNG	-1.67637508671539	7.59885584535383e-153
ENSG00000100106	TRIOBP	-1.67740707009289	1.58792805925311e-116
ENSG00000132256	TRIM5	-1.67745305084251	1.08470096162228e-61
ENSG00000175471	MCTP1	-1.67944987073132	1.08000405479855e-13
ENSG00000099326	MZF1	-1.67972733087183	1.27388357291226e-45
ENSG00000186297	GABRA5	-1.67999983762026	1.4363612777697e-64
ENSG00000168393	DTYMK	-1.68086007414979	2.34952253362102e-80
ENSG00000170365	SMAD1	-1.68117309035303	1.08485274745558e-18
ENSG00000172379	ARNT2	-1.68270143575139	6.35629535620254e-87
ENSG00000166548	TK2	-1.68381511679992	1.23594429200831e-48
ENSG00000152253	SPC25	-1.6841439321646	1.66602755876284e-38
ENSG00000189057	FAM111B	-1.68486395137717	7.91631442432872e-60
ENSG00000104472	CHRAC1	-1.68740825316875	7.46695284076122e-90
ENSG00000159788	RGS12	-1.68751363967257	1.09091348630089e-40
ENSG00000136827	TOR1A	-1.68782960907152	1.30030817760 58e-119
ENSG00000262558	N.A.	-1.68894146891551	0.00920112363033722
ENSG00000227946	N.A.	-1.68971493404087	0.00230826456134869
ENSG00000162736	NCSTN	-1.69003004434734	7.74444776184151e-137
ENSG00000112118	MCM3	-1.69240436640323	3.15818768437631e-202
ENSG00000142102	ATHL1	-1.69438014839822	4.58609378171492e-26
ENSG00000069275	NUCKS1	-1.69446116101919	0
ENSG00000143222	UFC1	-1.69451918163172	4.15925484534859e-91

ENSG00000185236	RAB11B	-1.69501245422947	1.78811061455896e-112
ENSG00000166510	CCDC68	-1.69565428284272	1.23228623596747e-61
ENSG00000237864	LINC00322	-1.6962533129143	0.00906068048529795
ENSG00000138376	BARD1	-1.69711023707921	1.20578783744433e-42
ENSG00000104964	AES	-1.69762094473765	1.30712525100276e-198
ENSG00000137767	SQRDL	-1.69773837974897	4.18067042205044e-56
ENSG00000115919	KYNU	-1.69836249081858	1.97530652074667e-226
ENSG00000143450	OAZ3	-1.69955051780889	0.00439583738730388
ENSG00000260693	N.A.	-1.69956078620448	0.00643791586705973
ENSG00000180801	ARSJ	-1.69978733611139	5.55013482538863e-41
ENSG00000257027	N.A.	-1.7001942604443	8.82181799008599e-12
ENSG00000279348	N.A.	-1.70032814841864	2.34381282175324e-11
ENSG00000185101	ANO9	-1.70103284632601	5.79022468996744e-07
ENSG00000140525	FANCI	-1.70114485060547	0
ENSG00000100403	ZC3H7B	-1.70309961305913	9.7524949475778e-122
ENSG00000276672	N.A.	-1.70345703150431	2.20605775255154e-09
ENSG00000138669	PRKG2	-1.70395502720491	1.91443121280643e-07
ENSG00000163840	DTX3L	-1.70692114668738	3.70935239458795e-62
ENSG00000129007	CALML4	-1.70745876959657	3.70549128182583e-16
ENSG00000106804	C5	-1.70769346883597	4.30887048976976e-209
ENSG00000115129	TP53I3	-1.7085697366461	4.39472707760237e-108
ENSG00000230061	TRPM2-AS	-1.70874364911038	0.00381144633930054
ENSG00000121578	B4GALT4	-1.70942815670692	1.98655616759694e-212
ENSG00000169668	BCRP2	-1.71146593834587	0.00665507260964539
ENSG00000171867	PRNP	-1.71178959587904	2.19330303213114e-290
ENSG00000229404	LINC00858	-1.71332722493514	0.000628440795184621
ENSG00000278259	MYO19	-1.71335226937506	5.06944435446495e-179
ENSG00000149289	ZC3H12C	-1.71648011636926	6.68425130337952e-28
ENSG00000181481	RNF135	-1.71772324640633	2.93672419100276e-57
ENSG00000092929	UNC13D	-1.71844055331029	2.664101340161e-38
ENSG00000196547	MAN2A2	-1.7188971717542	8.72355256221417e-114
ENSG00000134072	CAMK1	-1.71942415167598	6.56868706672802e-31
ENSG00000114270	COL7A1	-1.72122828746263	7.74154637368424e-174
ENSG00000164050	PLXNB1	-1.72168123131592	7.07976884492357e-67
ENSG00000280164	N.A.	-1.72169628817298	0.000263174892073287
ENSG00000175643	RMI2	-1.72192898841789	2.12692121278967e-62
ENSG00000165152	TMEM246	-1.72503184530557	4.67537254209397e-83
ENSG00000188191	PRKAR1B	-1.72509096463429	2.76016195009001e-52
ENSG00000138696	BMPR1B	-1.72618428481636	4.01460941092012e-86
ENSG00000055332	EIF2AK2	-1.72687640970996	3.7507529036203e-151
ENSG00000275410	HNF1B	-1.72719627793323	5.34406727651526e-70
ENSG00000111052	LIN7A	-1.72758226241854	1.8485277608261e-16
ENSG00000154930	ACSS1	-1.72947175373947	3.78371369218067e-55
ENSG00000006042	TMEM98	-1.73052626946074	4.2731983923064e-76
ENSG00000183778	B3GALT5	-1.73055085751082	5.88675938295528e-08
ENSG00000242193	N.A.	-1.73133138202776	3.46942972918152e-38
ENSG00000182310	SPACA6P	-1.73382058905419	6.61748553438074e-16
ENSG00000144452	ABCA12	-1.73396086069648	6.12828549543481e-20
ENSG00000131668	BARX1	-1.73693338495374	0.00011968680923527

ENSG00000178761	FAM219B	-1.73827086944809	4.148861597747e-76
ENSG00000274020	LINC01138	-1.73850746198749	2.96881331414951e-06
ENSG00000159263	SIM2	-1.73853277315608	8.65500395747550e-30
ENSG00000198455	ZXDB	-1.73988003730963	7.0701990258519e-43
ENSG00000149596	JPH2	-1.74012768800794	2.00341999449715e-06
ENSG00000197587	DMBX1	-1.74127446038609	9.66799990007999e-06
ENSG00000260804	N.A.	-1.74393688771312	7.11167081556191e-26
ENSG00000183814	LIN9	-1.74458072633483	2.33324744231086e-41
ENSG00000144840	RABL3	-1.74556317331516	5.12464866884618e-87
ENSG00000067798	NAV3	-1.74645166574661	7.51969537576191e-22
ENSG00000012171	SEMA3B	-1.74673527343961	4.66195690315854e-55
ENSG00000172197	MBOAT1	-1.74769628049769	4.36004595614219e-16
ENSG00000111331	OAS3	-1.75027056778813	3.00681474006159e-235
ENSG00000184995	IFNE	-1.75074799831899	0.000418161463477026
ENSG00000074657	ZNF532	-1.75121633305901	1.28574359214533e-121
ENSG00000069943	PIGB	-1.75121668338677	3.02316058077617e-52
ENSG00000214290	COLCA2	-1.75143522206734	0.000514650168545657
ENSG00000233608	TWIST2	-1.75149687898952	1.16778239563824e-08
ENSG00000212123	PRR22	-1.75202415200545	0.000116481330098709
ENSG00000054392	HHAT	-1.7521390044624	6.20652422016563e-15
ENSG00000101412	E2F1	-1.75291526441079	1.90020839571036e-96
ENSG00000261069	N.A.	-1.7534954807177	4.85931958279315e-05
ENSG00000076003	MCM6	-1.75648820146013	2.94345684442085e-215
ENSG00000157110	RBPMS	-1.75763130247612	1.38438265561369e-144
ENSG00000139192	TAPBPL	-1.75790610243464	2.7716406308389e-16
ENSG00000137449	CPEB2	-1.76097801069883	1.02916739260842e-54
ENSG00000132517	SLC52A1	-1.76117160312212	5.26834508012642e-05
ENSG00000151790	TDO2	-1.76176014292596	0.00141166131127164
ENSG00000123388	HOXC11	-1.76222256968118	0.00524850007779664
ENSG00000150722	PPP1R1C	-1.7642646006337	4.17193223544026e-14
ENSG00000230606	N.A.	-1.76510612466653	1.56325765979367e-11
ENSG00000138193	PLCE1	-1.76556227048161	6.27077613076667e-135
ENSG00000119943	PYROXD2	-1.76644284712813	5.66003399196423e-11
ENSG00000054598	FOXC1	-1.76761379642188	5.85867638132619e-30
ENSG00000178922	HYI	-1.76845626255645	6.48655817550383e-08
ENSG00000113504	SLC12A7	-1.76924051674844	1.58524141636965e-119
ENSG00000186364	NUDT17	-1.76954129919307	1.20298652807631e-13
ENSG00000182022	CHST15	-1.76987156744222	7.37970068145979e-34
ENSG00000197702	PARVA	-1.77135596961996	4.99283943112841e-108
ENSG00000276141	WHAMMP3	-1.77191060056391	5.87430368615353e-14
ENSG00000144554	FANCD2	-1.77291507205646	6.61443846314105e-139
ENSG00000018699	TTC27	-1.77402590087437	5.04936676256275e-61
ENSG00000135776	ABCB10	-1.77621408733304	1.25026991268064e-73
ENSG00000261801	LOXL1-AS1	-1.77663853029855	0.0053846347572264
ENSG00000114480	GBE1	-1.77773397405035	6.06064345277675e-105
ENSG00000145349	CAMK2D	-1.77841373750475	4.3908348121215e-146
ENSG00000170312	CDK1	-1.77947585710042	1.54300923175281e-228
ENSG00000162390	ACOT11	-1.77987810301922	2.66384224134797e-12
ENSG00000186188	FFAR4	-1.78449396248578	0.000874887063125523

ENSG00000079999	KEAP1	-1.78490614593812	3.19775744247106e-112
ENSG00000241634	N.A.	-1.78491789601227	0.00841519746026069
ENSG00000067646	ZFY	-1.78596535249072	4.43256899117724e-18
ENSG00000136490	LIMD2	-1.78875907019942	6.23090702032697e-22
ENSG00000261167	N.A.	-1.78986513965369	1.95037636074831e-07
ENSG00000105483	CARD8	-1.79047929326666	8.44067613182867e-71
ENSG00000112992	NNT	-1.79077005345806	6.80074763762502e-140
ENSG00000260136	N.A.	-1.79167811212585	3.46168280648015e-08
ENSG00000140406	MESDC1	-1.79196718652565	2.40898078425922e-45
ENSG00000115616	SLC9A2	-1.79404180510345	2.68011665244724e-12
ENSG00000172469	MANEA	-1.7943152536721	7.84208357241326e-45
ENSG00000075643	MOCOS	-1.79450504321466	1.30160593133293e-36
ENSG00000105854	PON2	-1.79496136044552	0
ENSG00000169136	ATF5	-1.79536402784151	1.12773791947649e-87
ENSG00000141569	TRIM65	-1.79543078794772	3.5672190644424e-95
ENSG00000164430	MB21D1	-1.7958065095125	7.12006852632177e-29
ENSG00000105928	DFNA5	-1.79622838453181	2.6824035751043e-68
ENSG00000115875	SRSF7	-1.79650248305026	1.2264258868266e-274
ENSG00000141994	DUS3L	-1.79682199379395	9.76583997828584e-36
ENSG00000228065	LINC01515	-1.79916530289726	2.18590146608793e-09
ENSG00000267147	N.A.	-1.80095194573122	0.00111087327837086
ENSG00000265787	CYP4F35P	-1.80104650967423	0.00647796927605005
ENSG00000182749	PAQR7	-1.80136204951515	6.91760316438002e-30
ENSG00000204802	N.A.	-1.80203211539869	0.0017512818974046
ENSG00000125458	NT5C	-1.80237376453444	1.43584849310851e-87
ENSG00000141905	NFIC	-1.8024049808156	1.32799514967706e-156
ENSG00000224078	SNHG14	-1.80317174139258	5.36583039566506e-141
ENSG00000260852	FBXL19-AS1	-1.80327570693787	3.02697885245402e-18
ENSG00000176532	PRR15	-1.80384524614488	6.06821140076988e-61
ENSG00000259985	N.A.	-1.80601147104366	0.00217828761422152
ENSG00000273036	FAM95C	-1.80625494870987	4.57132079733745e-17
ENSG00000245848	CEBPA	-1.80849470430765	1.55323492883807e-18
ENSG00000117411	B4GALT2	-1.81152001937943	3.5293485663675e-74
ENSG00000136104	RNASEH2B	-1.8115980231908	1.64753431160267e-54
ENSG00000086548	CEACAM6	-1.81478104199162	0
ENSG00000129187	DCTD	-1.81615854116866	1.97904821338665e-117
ENSG00000175322	ZNF519	-1.81644467907098	1.57699224549477e-12
ENSG00000247950	SEC24B-AS1	-1.81655327664824	0.0013183060329516
ENSG00000260597	N.A.	-1.81763991162628	0.000440402459353736
ENSG00000188917	TRMT2B	-1.81812664244597	1.70121970365791e-77
ENSG00000106868	SUSD1	-1.81878423554237	7.97789597259713e-64
ENSG00000275180	N.A.	-1.8197542120169	2.03902635207109e-15
ENSG00000279117	N.A.	-1.82098275867837	2.35980964823899e-108
ENSG00000110063	DCPS	-1.82109765823473	1.08021871776463e-26
ENSG00000228439	TSTD3	-1.82171164834178	0.00703626194674748
ENSG00000237172	B3GNT9	-1.82208708099776	2.00252178610286e-28
ENSG00000168758	SEMA4C	-1.82313776160093	4.20268661782789e-34
ENSG00000132749	MTL5	-1.82354916049203	5.1018425401259e-13
ENSG00000270157	N.A.	-1.82381431084386	9.79666465486835e-12

ENSG00000074219	TEAD2	-1.82502779985492	4.35238077229408e-63
ENSG00000138772	ANXA3	-1.82779649223466	9.30989351194078e-119
ENSG00000232931	LINC00342	-1.82807621247006	5.24075559691458e-13
ENSG00000106100	NOD1	-1.83178348285871	2.99759559346856e-25
ENSG00000186765	FSCN2	-1.83195689860046	0.000363844390472627
ENSG00000132434	LANCL2	-1.83333676430141	1.21730845045011e-40
ENSG00000249464	LINC01091	-1.83602561519268	2.76119827545712e-06
ENSG00000165804	ZNF219	-1.83633190739995	4.36794111076653e-41
ENSG00000162458	FBLIM1	-1.84026463128898	9.51925843060412e-59
ENSG00000144355	DLX1	-1.84094192390405	3.5424263014219e-06
ENSG00000164294	GPX8	-1.84218075771199	3.78745392016918e-144
ENSG00000279425	N.A.	-1.84257185831596	0.00274706572839272
ENSG00000100926	TM9SF1	-1.84500999617188	5.44469581241953e-17
ENSG00000039139	DNAH5	-1.84783507791515	4.40487693930823e-21
ENSG00000231107	LINC01508	-1.85057337577722	0.00459626639059313
ENSG00000172936	MYD88	-1.85120059459933	9.65645151497842e-97
ENSG00000064225	ST3GAL6	-1.85122126345434	6.46396936887193e-05
ENSG00000138346	DNA2	-1.85207634280829	6.016904724639e-99
ENSG00000142185	TRPM2	-1.85240326327051	4.57440755084931e-21
ENSG00000137968	SLC44A5	-1.85255343961921	9.01233786637519e-06
ENSG00000143499	SMYD2	-1.85454868445383	3.21271201179038e-70
ENSG00000261098	N.A.	-1.85531609148237	0.00649723326831646
ENSG00000100297	MCM5	-1.85566375640333	4.59946390398387e-166
ENSG00000107099	DOCK8	-1.85854012027484	0.00112190157915114
ENSG00000146858	ZC3HAV1L	-1.85983905422607	6.87429316292015e-23
ENSG00000253671	N.A.	-1.86059423380827	2.07107615205169e-14
ENSG00000091436	N.A.	-1.86169375589358	1.64554228595537e-212
ENSG00000198331	HYLS1	-1.86262581342481	2.14466922207777e-50
ENSG00000105672	ETV2	-1.86402116246967	0.0023109484521265
ENSG00000118507	AKAP7	-1.86510531262382	7.56698652806868e-16
ENSG00000213397	HAUS7	-1.86739251109508	0.00641904681869523
ENSG00000159217	IGF2BP1	-1.86898205360186	1.26994153874503e-223
ENSG00000174428	GTF2IRD2B	-1.8694394455001	0.00113526193588073
ENSG00000250312	ZNF718	-1.86953367957155	1.04054812788136e-05
ENSG00000099203	TMED1	-1.87005891915907	1.05554091157431e-41
ENSG00000259256	N.A.	-1.87180051638744	0.000375853964466352
ENSG00000166888	STAT6	-1.87226230478833	2.37514746539577e-182
ENSG00000234231	N.A.	-1.87347818084026	0.00214195578052818
ENSG00000162437	RAVER2	-1.87493454112247	7.30356521475559e-55
ENSG00000140548	ZNF710	-1.87627356851319	1.51870644923188e-40
ENSG00000055732	MCOLN3	-1.87633890742939	4.33624027886398e-35
ENSG00000214717	ZBED1	-1.87648798787591	9.33497102903811e-165
ENSG00000260265	N.A.	-1.87656257312993	0.000212252540489582
ENSG00000258711	N.A.	-1.87707200430168	1.355389716997e-05
ENSG00000112837	TBX18	-1.87847610760232	5.7914913989859e-210
ENSG00000242574	HLA-DMB	-1.87929651520955	9.49697627899383e-146
ENSG00000163739	CXCL1	-1.8802192497667	1.89035899431854e-06
ENSG00000144677	CTDSPL	-1.880413604318	2.97436434159842e-133
ENSG00000166831	RBPMS2	-1.88063679766374	5.07898518433427e-17

ENSG00000165105	RASEF	-1.88208322103264	8.44850130856599e-103
ENSG00000138496	PARP9	-1.88303895320278	2.84982077265747e-53
ENSG00000090661	CERS4	-1.88408075068999	7.79659141335126e-10
ENSG00000134755	DSC2	-1.88615599182891	9.40085702001169e-58
ENSG00000165655	ZNF503	-1.88704803155669	2.26935037966847e-66
ENSG00000132437	DDC	-1.88795992351716	1.29570735101574e-43
ENSG00000198780	FAM169A	-1.88842025180668	3.43146425095777e-71
ENSG00000113368	LMNB1	-1.88882061121971	8.74028712886333e-152
ENSG00000179046	TRIML2	-1.88937770498767	4.17473593656042e-76
ENSG00000224251	N.A.	-1.8939402712196	7.52057014419567e-08
ENSG00000057935	MTA3	-1.8957991893257	1.347481959379e-92
ENSG00000167460	TPM4	-1.89830159689825	0
ENSG00000142227	EMP3	-1.90144622551574	1.03670507625881e-146
ENSG00000155849	ELMO1	-1.9025587242738	8.74039578808089e-14
ENSG00000214425	LRRC37A4P	-1.90268518193389	5.05171590684977e-58
ENSG00000112208	BAG2	-1.90324361512168	3.01993030544262e-107
ENSG00000240800	ATP8A2P1	-1.90504874920672	1.69862743593873e-05
ENSG00000134215	VAV3	-1.90537317210235	1.42956213770287e-41
ENSG00000185090	MANEAL	-1.90593368834905	4.00252175707407e-51
ENSG00000140398	NEIL1	-1.90717894692276	6.36513281408875e-06
ENSG00000119139	TJP2	-1.90852181460472	1.77212470848329e-117
ENSG00000110455	ACCS	-1.90870376974762	2.13582628042826e-55
ENSG00000119917	IFIT3	-1.9087511454737	2.00085250777134e-11
ENSG00000172716	SLFN11	-1.90891481413491	3.56189608623886e-181
ENSG00000173535	TNFRSF10C	-1.91075479932391	2.56420367074553e-05
ENSG00000136938	ANP32B	-1.91180026698938	1.17574458710691e-221
ENSG00000111700	SLCO1B3	-1.91234626441284	1.25466726171152e-48
ENSG00000162496	DHRS3	-1.9126846564866	4.51555768869517e-90
ENSG00000198933	TBKBP1	-1.91427949991865	2.98715343428934e-19
ENSG00000174125	TLR1	-1.91666955859113	6.12063040144607e-07
ENSG00000065054	SLC9A3R2	-1.91667305471709	1.71526572174148e-197
ENSG00000259495	N.A.	-1.91942618554816	1.2112721031991e-20
ENSG00000111261	MANSC1	-1.92226356741152	1.05969063473004e-100
ENSG00000198203	SULT1C2	-1.92339989823833	0.00636162333417457
ENSG00000280152	N.A.	-1.92579866115843	0.00478615021437011
ENSG00000186889	TMEM17	-1.92612902222196	1.20256720758994e-15
ENSG00000204175	GPRIN2	-1.9262226340429	1.7357151795026e-42
ENSG00000230882	N.A.	-1.9263566165758	2.04240189422223e-28
ENSG00000064651	SLC12A2	-1.92692621755386	0
ENSG00000109674	NEIL3	-1.92712453556427	4.23487034332145e-49
ENSG00000245213	N.A.	-1.92774810901929	1.70311857020928e-05
ENSG00000243232	PCDHAC2	-1.92919700376831	1.36335722016952e-63
ENSG00000178026	LRRC75B	-1.9309978935801	1.08733623372645e-14
ENSG00000273203	N.A.	-1.93299340424122	0.000141770193470187
ENSG00000090447	TFAP4	-1.93841149700803	2.01613764419317e-24
ENSG00000165458	INPPL1	-1.94022066093368	3.05264482084692e-241
ENSG00000206159	GYG2P1	-1.94030140553764	0.0011263654058997
ENSG00000149600	COMMD7	-1.94042419331934	5.93490369468642e-105
ENSG00000006210	CX3CL1	-1.9407772289833	2.95735429831509e-11

ENSG00000248334	WHAMMP2	-1.94192455828219	5.4585500630442e-18
ENSG00000269916	N.A.	-1.94332228785176	4.58910419312929e-06
ENSG00000145014	TMEM44	-1.94880504041232	2.02727006922087e-29
ENSG00000144040	SFXN5	-1.9490387846384	1.0132427699698e-48
ENSG00000163131	CTSS	-1.95082281583409	2.83123737938534e-137
ENSG00000080839	RBL1	-1.95095251902678	3.89590855037574e-116
ENSG00000100599	RIN3	-1.95521011753047	9.89248683537628e-53
ENSG00000172575	RASGRP1	-1.95709997400392	0.00119667255655726
ENSG00000205730	ITPRIPL2	-1.96656135017288	1.00065145241945e-82
ENSG00000275854	N.A.	-1.96795571641436	0.00159032778636751
ENSG00000167723	TRPV3	-1.96894881827909	0.00638960208448367
ENSG00000203761	MSTO2P	-1.97023119162335	1.19386734560588e-05
ENSG00000225813	N.A.	-1.97059676947406	4.37625466735522e-09
ENSG00000280219	N.A.	-1.97349904232212	0.00318585385439864
ENSG00000085871	MGST2	-1.97350608812547	2.5677289015896e-79
ENSG00000112294	ALDH5A1	-1.97486244493667	2.94648663116734e-77
ENSG00000213339	QTRT1	-1.97730371110651	8.19406237509278e-35
ENSG00000164307	ERAP1	-1.97762331158299	1.64811570264443e-84
ENSG00000160062	ZBTB8A	-1.97799045814023	1.64325922396841e-09
ENSG00000188747	NOXA1	-1.98179114929212	3.00119000459177e-17
ENSG00000164125	FAM198B	-1.9838008483971	1.01452509058895e-25
ENSG00000279792	N.A.	-1.98441895328069	0.00124927338630752
ENSG00000179922	ZNF784	-1.9872175203146	1.41905484601588e-16
ENSG00000121316	PLBD1	-1.98831238145477	7.03627732122336e-11
ENSG00000023171	GRAMD1B	-1.98860331309236	2.73813777998589e-199
ENSG00000241316	SUCLG2-AS1	-1.98986150535472	4.69639223204867e-14
ENSG00000008394	MGST1	-1.99203138471513	0
ENSG00000185070	FLRT2	-1.99551279161101	3.11429409593139e-24
ENSG00000173898	SPTBN2	-1.99688020573934	2.1086253142595e-47
ENSG00000273129	PACERR	-1.99869943660891	0.00049281293475397
ENSG00000072163	LIMS2	-1.99918410081467	9.53808772828845e-05
ENSG00000170271	FAXDC2	-1.99924212398734	0.00154147478839153
ENSG00000257093	KIAA1147	-1.9998014594026	1.00692191511702e-167
ENSG00000005889	ZFX	-2.00006746822868	1.0281648703393e-136
ENSG00000178773	CPNE7	-2.00204599222704	4.67085220052527e-36
ENSG00000233903	N.A.	-2.00270872581549	0.000558690582914041
ENSG00000050327	ARHGEF5	-2.00327787706101	2.34428731165253e-07
ENSG00000158301	GPRASP2	-2.00358528833048	3.83051979934379e-37
ENSG00000165102	HGSNAT	-2.00547745726664	7.65179325271105e-168
ENSG00000029534	ANK1	-2.00836315196386	1.84242266706004e-05
ENSG00000162433	AK4	-2.01284225205892	1.87660889809491e-90
ENSG00000121039	RDH10	-2.014974925377	9.89142888554975e-183
ENSG00000143321	HDGF	-2.01549259954536	0
ENSG00000164045	CDC25A	-2.01596803334907	1.44207102014753e-51
ENSG00000119772	DNMT3A	-2.01678987616687	7.03880105721634e-76
ENSG00000115325	DOK1	-2.02032740589947	6.5210107167843e-10
ENSG00000198829	SUCNR1	-2.02095694216302	8.98997776050339e-21
ENSG00000152953	STK32B	-2.02412399848669	1.29230057410374e-86
ENSG00000082512	TRAF5	-2.02791960380361	4.48014756587398e-73

ENSG00000108312	UBTF	-2.02865551982804	0
ENSG00000102390	PBDC1	-2.02995625164952	4.98325580286256e-59
ENSG00000198205	ZXDA	-2.03935223773198	1.25342670693994e-08
ENSG00000155957	TMBIM4	-2.03947587583706	4.41232986362096e-31
ENSG00000279026	N.A.	-2.0477034092792	0.00104685441541382
ENSG00000164010	ERMAP	-2.04779073844953	1.37587221437097e-31
ENSG00000157693	C9orf91	-2.04822473088033	7.18617708143836e-57
ENSG00000135480	KRT7	-2.04854795432111	0
ENSG00000197774	EME2	-2.05182895069035	6.82091429155694e-43
ENSG00000168398	BDKRB2	-2.05288117461816	0.000201861518498519
ENSG00000182752	PAPPA	-2.05324315351105	3.07476680150954e-14
ENSG00000230650	N.A.	-2.05354280689367	1.60039729388295e-14
ENSG00000213096	ZNF254	-2.05474432687561	1.10410870747934e-50
ENSG00000142494	SLC47A1	-2.05499400799768	3.77806074756783e-87
ENSG00000196230	TUBB	-2.05504483760314	0
ENSG00000173918	C1QTNF1	-2.05510137611405	3.37405819499323e-99
ENSG00000233184	N.A.	-2.05643416461019	2.70900593738127e-12
ENSG00000145908	ZNF300	-2.059800413687	1.42007399283315e-54
ENSG00000167880	EVPL	-2.06032156736934	1.67806656561971e-117
ENSG00000106541	AGR2	-2.06040196348495	0
ENSG00000116815	CD58	-2.06134120590167	5.70249489156827e-34
ENSG00000164742	ADCY1	-2.06618637424966	2.968995584417446e-162
ENSG00000068079	IFI35	-2.0665879314664	1.57655701706546e-45
ENSG00000152217	SETBP1	-2.06682344528295	6.70280075256602e-47
ENSG00000132570	PCBD2	-2.06950965450398	4.01566598753198e-31
ENSG00000160352	ZNF714	-2.06965454324637	7.46383891505121e-41
ENSG00000166402	TUB	-2.06979171935489	1.71009999098959e-57
ENSG00000227388	N.A.	-2.07333876923854	0.0016193807183902
ENSG00000171016	PYGO1	-2.07379439624096	2.69924465262111e-63
ENSG00000125637	PSD4	-2.07530976898383	1.98580414278878e-14
ENSG00000059145	UNKL	-2.07667198634431	1.93038895265212e-86
ENSG00000263001	GTF2I	-2.077148588915	1.2014552841077e-149
ENSG00000120896	SORBS3	-2.07932372242576	2.0837903968239e-178
ENSG00000280145	N.A.	-2.07990958106249	1.63686757310623e-06
ENSG00000150526	MIA2	-2.07993951874883	0.00455830820565369
ENSG00000115107	STEAP3	-2.08093320781216	1.50294479813442e-87
ENSG00000112541	PDE10A	-2.08104765478125	1.70896736888726e-47
ENSG00000170571	EMB	-2.08144644138515	2.3365869958463e-120
ENSG00000122952	ZWINT	-2.08166366212471	1.99664994984579e-272
ENSG00000165891	E2F7	-2.08177471643778	9.24084578413635e-101
ENSG00000035141	FAM136A	-2.08200257102738	1.59635509801472e-203
ENSG00000262943	ALOX12P2	-2.08316971095001	5.12161710186042e-16
ENSG00000150455	TIRAP	-2.08326116388341	1.8531324964768e-26
ENSG00000100105	PATZ1	-2.08646604095323	1.19669551793953e-78
ENSG00000205089	CCNI2	-2.08791406209886	0.00641567366585136
ENSG00000116117	PARD3B	-2.0921646039983	1.43566743257129e-23
ENSG00000275578	N.A.	-2.09302163447552	9.27710012535255e-07
ENSG00000160191	PDE9A	-2.09363149240607	9.36057134657108e-23
ENSG00000172572	PDE3A	-2.09366375569232	6.65386845901898e-109

ENSG00000164647	STEAP1	-2.09616191472413	1.02746318753251e-83
ENSG00000239697	TNFSF12	-2.09675786336917	9.0340255064449e-20
ENSG00000253293	HOXA10	-2.09784292334362	5.07621124120299e-26
ENSG00000121075	TBX4	-2.10170586776225	0.000137498543093387
ENSG00000204584	N.A.	-2.10245505252667	0.000197099794139601
ENSG00000155465	SLC7A7	-2.10251193888075	1.59873346048073e-98
ENSG00000144354	CDCA7	-2.10526972190678	4.01262212938861e-136
ENSG00000144152	FBLN7	-2.10635612717106	7.03028399065769e-07
ENSG00000178814	OPLAH	-2.10709888741408	2.59223125870592e-34
ENSG00000166401	SERPINB8	-2.11188576740068	1.35554598285113e-14
ENSG00000109084	TMEM97	-2.1138011246165	0
ENSG00000183092	BEGAIN	-2.11536482927962	9.80641442587115e-06
ENSG00000146386	ABRACL	-2.1159648934895	2.58868551962865e-52
ENSG00000140396	NCOA2	-2.11671987214998	1.07886437895037e-165
ENSG00000064547	LPAR2	-2.11796898341228	1.09396679042406e-11
ENSG00000101198	NKAIN4	-2.11910943013092	0.00759535696669487
ENSG00000135362	PRR5L	-2.1241362328952	1.61519709132924e-22
ENSG00000129255	MPDU1	-2.12503101847821	7.88066503574446e-172
ENSG00000180861	LINC01559	-2.12659158734803	2.69581548069423e-05
ENSG00000134516	DOCK2	-2.12686375200926	2.83731464380482e-07
ENSG00000259953	N.A.	-2.12903242929474	9.58978589649836e-12
ENSG00000144843	ADPRH	-2.13047615622823	1.72527464977703e-16
ENSG00000188677	PARVB	-2.13270852170242	3.02226129910785e-61
ENSG00000132326	PER2	-2.13427675248147	7.8571165686533e-115
ENSG00000087448	KLHL42	-2.13437175044171	2.15906588971015e-155
ENSG00000133321	RARRES3	-2.13517113246241	2.11669508697082e-08
ENSG00000167889	MGAT5B	-2.13706138122116	1.23402613382677e-110
ENSG00000157214	STEAP2	-2.14120559676554	4.66302767032878e-147
ENSG00000151876	FBXO4	-2.14122457898424	1.32792977203744e-31
ENSG00000184216	IRAK1	-2.14363631609665	8.53885879424416e-288
ENSG00000119922	IFIT2	-2.14648274741651	1.1894675302566e-12
ENSG00000148288	GBGT1	-2.14766996361773	0.00106574389529127
ENSG00000078900	TP73	-2.14767647971678	0.00816491762604418
ENSG00000267751	N.A.	-2.15012218865366	5.07318667423857e-09
ENSG00000188906	LRRK2	-2.15103553362626	1.47223439064924e-184
ENSG00000114279	FGF12	-2.15114296306298	2.79265985806871e-15
ENSG00000149257	SERPINH1	-2.15142398306482	2.36304213300865e-232
ENSG00000150760	DOCK1	-2.15223143423337	4.04422337479644e-251
ENSG00000245750	DRAIC	-2.15270305655385	7.93713300460225e-16
ENSG00000137842	TMEM62	-2.15386560500726	1.02153256651494e-68
ENSG00000163362	C1orf106	-2.15406595536236	2.61128353834209e-253
ENSG00000184979	USP18	-2.15482991204173	1.03036253370173e-53
ENSG00000172878	METAP1D	-2.15571334643704	7.50181995166031e-18
ENSG00000186564	FOXD2	-2.15643141665347	3.0378632253125e-06
ENSG00000235423	N.A.	-2.15735319289561	5.35575043210306e-09
ENSG00000253187	HOXA10-AS	-2.15761322342142	0.000497432167978256
ENSG00000228526	N.A.	-2.15876197853734	1.85198416805843e-21
ENSG00000125780	TGM3	-2.15985610853683	0.00333781307957024
ENSG00000106927	AMBP	-2.16085472218336	1.32349543176613e-17

ENSG00000214575	CPEB1	-2.1609790819311	1.27151412321712e-09
ENSG00000170161	N.A.	-2.1617849627291	4.34378393367874e-06
ENSG00000111077	TNS2	-2.16532769728253	1.29004115734976e-144
ENSG00000158856	DMTN	-2.16588773883692	9.41780173147291e-162
ENSG00000134247	PTGFRN	-2.16898684319828	6.7716453160834e-141
ENSG00000111877	MCM9	-2.16953201489444	4.66251204038674e-50
ENSG00000169418	NPR1	-2.16957902009681	2.21853121417644e-99
ENSG00000221817	PPP3CB-AS1	-2.17024698544187	5.57124156955945e-14
ENSG00000136813	KIAA0368	-2.17255302338992	0
ENSG00000168404	MLKL	-2.17401004727907	2.69769335500135e-75
ENSG00000166922	SCG5	-2.17463947254893	2.28865643732531e-07
ENSG00000133067	LGR6	-2.17653879610193	0.00985557289290185
ENSG00000125247	TMTC4	-2.17716784676182	3.63530754000175e-76
ENSG00000176595	KBTBD11	-2.1893922027866	2.67831000920998e-43
ENSG00000058866	DGKG	-2.18949507154584	1.55043344426925e-47
ENSG00000136026	CKAP4	-2.19015714311083	0
ENSG00000168447	SCNN1B	-2.1902402058309	5.99503265091151e-22
ENSG00000276256	N.A.	-2.19087549935905	1.30223222871314e-35
ENSG00000116096	SPR	-2.19268494603945	1.01325868754458e-145
ENSG00000235609	N.A.	-2.19296083289729	8.60514009775383e-12
ENSG00000104518	GSDMD	-2.19299182147138	3.14444018521063e-92
ENSG00000256043	CTSO	-2.19552447017934	1.98965350943472e-07
ENSG00000139117	CPNE8	-2.19604893245701	2.15532572037581e-106
ENSG00000108511	HOXB6	-2.19614499566794	3.37094672548199e-52
ENSG00000124006	OBSL1	-2.19676456828552	6.21443386841996e-72
ENSG00000184900	SUMO3	-2.19714818245454	1.22973096951334e-139
ENSG00000196275	GTF2IRD2	-2.19829913794624	6.19674316569825e-08
ENSG00000132669	RIN2	-2.19854375914054	1.76041919073907e-112
ENSG00000186088	GSAP	-2.19916480936784	4.71258282057777e-27
ENSG00000184898	RBM43	-2.20009784887436	4.75837883592984e-26
ENSG00000130675	MNX1	-2.20150864178028	3.25370577425697e-34
ENSG00000099256	PRTFDC1	-2.20438551285342	5.96139989955725e-93
ENSG00000132773	TOE1	-2.20752626699681	4.56722850380098e-40
ENSG00000085831	TTC39A	-2.20848554796978	1.63003048662545e-25
ENSG00000002587	HS3ST1	-2.21020735490744	1.66625099536331e-06
ENSG00000219438	FAM19A5	-2.2120669675193	6.81788381232799e-35
ENSG00000196139	AKR1C3	-2.21271529775033	1.42484765456007e-130
ENSG00000183091	NEB	-2.21324979520499	2.68526265740891e-05
ENSG00000179715	PCED1B	-2.21407240655284	3.4277758941082e-107
ENSG00000188921	HACD4	-2.21492901841254	7.48427510319888e-45
ENSG00000167525	PROCA1	-2.21510416097619	5.87137641466006e-12
ENSG00000163430	FSTL1	-2.21733236449392	2.05929037131098e-119
ENSG00000205560	CPT1B	-2.22174055613405	0.00385039700673124
ENSG00000188483	IER5L	-2.22361130722237	1.91276391454073e-105
ENSG00000168077	SCARA3	-2.22732277105914	8.93260872990625e-170
ENSG00000166377	ATP9B	-2.22812714087617	1.97707475086659e-163
ENSG00000257167	TMPO-AS1	-2.22978596930176	2.39678007169166e-34
ENSG00000176022	B3GALT6	-2.22989562773168	2.71972287492632e-51
ENSG00000151632	AKR1C2	-2.23177626783834	0

ENSG00000184058	TBX1	-2.23282868780616	0.00438525125026841
ENSG00000029993	HMGB3	-2.23377950644819	0
ENSG00000235903	CPB2-AS1	-2.23793837324295	0.00312498359886963
ENSG00000132128	LRRC41	-2.23814976309149	6.28022592671005e-164
ENSG00000063127	SLC6A16	-2.23938805514013	3.1708402317264e-13
ENSG00000196517	SLC6A9	-2.24048419299497	4.30825257407096e-12
ENSG00000121895	TMEM156	-2.2410391885374	1.35803299227286e-104
ENSG00000178467	P4HTM	-2.24186982436518	1.01883287204675e-71
ENSG00000278948	N.A.	-2.24312408693488	1.42458218132114e-09
ENSG00000170004	CHD3	-2.24339266590047	0
ENSG00000135272	MDFIC	-2.24368716981988	2.34165693711791e-95
ENSG00000101076	HNF4A	-2.24917790366711	1.0380039173477e-158
ENSG00000175063	UBE2C	-2.24937631457155	3.63254364612554e-188
ENSG00000234373	SNX18P7	-2.2531861254569	0.000682229370613907
ENSG00000125798	FOXA2	-2.25605645212495	3.73412876811478e-103
ENSG00000178665	ZNF713	-2.25625463479058	1.59019162837665e-06
ENSG00000145248	SLC10A4	-2.2601109026705	1.06940923722374e-09
ENSG00000111676	ATN1	-2.26151938308659	5.145804465014e-287
ENSG00000134258	VTCN1	-2.26169280073182	0.000172247399280051
ENSG00000132793	LPIN3	-2.26509693637596	9.41143203509125e-68
ENSG00000182600	C2orf82	-2.26528979437701	6.07647176495504e-14
ENSG00000229719	N.A.	-2.26824267044635	6.68934084523409e-06
ENSG00000171126	KCNG3	-2.27114169331931	0.000523892909936426
ENSG00000021762	OSBPL5	-2.27448780388666	2.04560049742938e-42
ENSG00000120093	HOXB3	-2.27796368377907	1.18900338792757e-74
ENSG00000162337	LRP5	-2.27876602454489	0
ENSG00000108602	ALDH3A1	-2.28053413524888	0
ENSG00000230699	N.A.	-2.28090574470433	0.0014506197979065
ENSG00000178409	BEND3	-2.28224305584359	4.45309688625068e-31
ENSG00000183072	NKX2-5	-2.28382954909741	3.19925567751438e-16
ENSG00000241014	N.A.	-2.28427257450905	2.01877092946765e-05
ENSG00000109320	NFKB1	-2.28476985606776	2.00588227127335e-128
ENSG00000167695	FAM57A	-2.29063024343045	4.12906016080235e-95
ENSG00000226742	HSBP1L1	-2.29223917085052	6.55467883354236e-41
ENSG00000143452	HORMAD1	-2.29234639868297	4.48640192353755e-48
ENSG00000130762	ARHGEF16	-2.29382795307952	5.32769454046864e-13
ENSG00000064393	HIPK2	-2.2940903556447	0
ENSG00000123416	TUBA1B	-2.29751454168143	0
ENSG00000244300	GATA2-AS1	-2.30096178743356	1.95291100579497e-05
ENSG00000149243	KLHL35	-2.30148038278863	0.000152323450772155
ENSG00000174292	TNK1	-2.30220385935846	2.63683403793104e-07
ENSG00000120800	UTP20	-2.31099979049797	2.2764499277891e-224
ENSG00000236095	N.A.	-2.31141897614575	3.45806814440529e-10
ENSG00000188112	C6orf132	-2.311665554428	7.86677737589844e-08
ENSG00000061273	HDAC7	-2.31173501854657	2.72587719947157e-185
ENSG00000230453	ANKRD18B	-2.31177942887938	4.41827596323451e-23
ENSG00000169410	PTPN9	-2.31568835758616	3.98276347019871e-200
ENSG00000043143	JADE2	-2.31826275523895	1.22233592900254e-216
ENSG00000162542	TMCO4	-2.3245813695874	3.90943511656116e-28

ENSG00000127324	TSPAN8	-2.33706201948055	4.94082532192691e-06
ENSG00000180287	PLD5	-2.33736238743586	4.8863680499969e-22
ENSG00000034053	APBA2	-2.3376017005454	4.30666837706014e-97
ENSG00000181938	GINS3	-2.33819999787207	7.89697280546326e-138
ENSG00000058056	USP13	-2.33821024540933	2.59529675184432e-108
ENSG00000234918	N.A.	-2.33947323729886	2.82783429065858e-07
ENSG00000099250	NRP1	-2.34401081998586	0
ENSG00000136147	PHF11	-2.34963733283904	1.1408276674071e-21
ENSG00000126561	STAT5A	-2.35200568137403	5.0865241447909e-25
ENSG00000047230	CTPS2	-2.35430085385862	1.55008029960354e-86
ENSG00000118407	FILIP1	-2.35491110839132	1.93009203412203e-17
ENSG00000136205	TNS3	-2.35634730980507	0
ENSG00000127831	VIL1	-2.35821341142326	1.47167147332905e-22
ENSG00000148057	IDNK	-2.36153134895566	5.30592348988763e-19
ENSG00000184925	LCN12	-2.365162676892	0.000104620926500531
ENSG00000215808	LINC01139	-2.37433371487491	0.000252051948389345
ENSG00000279516	FAM230C	-2.37938289796257	3.8136733456584e-13
ENSG00000250799	PRODH2	-2.38179357887963	5.47252961439701e-29
ENSG00000015475	BID	-2.38252910264516	1.15439139958658e-107
ENSG00000113621	TXNDC15	-2.38270076570403	1.63216990886901e-198
ENSG00000112029	FBXO5	-2.38420309830853	1.40247940705885e-87
ENSG00000152939	MARVELD2	-2.38693467572598	1.03054262096286e-82
ENSG00000198113	TOR4A	-2.38848501272421	1.43635221216324e-141
ENSG00000251537	N.A.	-2.38893121380293	2.26527838962341e-05
ENSG00000120075	HOXB5	-2.3893111186668	2.7777008764312e-28
ENSG00000091428	RAPGEF4	-2.38960255232984	0.000259250006077025
ENSG00000179943	FIZ1	-2.38996917279697	4.64810996981049e-53
ENSG00000071967	CYBRD1	-2.39169766389483	1.4016216569776e-124
ENSG00000172086	KRCC1	-2.39193098926399	5.22124261993185e-66
ENSG00000155363	MOV10	-2.39337523642258	6.9646841542955e-143
ENSG00000164342	TLR3	-2.39420519755658	4.66960372656654e-11
ENSG00000184363	PKP3	-2.3980447829026	2.55316364044969e-121
ENSG00000106686	SPATA6L	-2.40245439458848	2.66682587317977e-23
ENSG00000100342	APOL1	-2.40277716511551	1.13980762422009e-09
ENSG00000070526	ST6GALNAC1	-2.40733367282304	2.75539359512038e-18
ENSG00000161267	BDH1	-2.40809382212603	4.09589464997288e-19
ENSG00000084453	SLCO1A2	-2.40911618274812	0.000160725633820279
ENSG00000165730	STOX1	-2.41213949202057	5.10478348152296e-17
ENSG00000056736	IL17RB	-2.41523728937307	2.7715581095619e-08
ENSG00000154678	PDE1C	-2.42313703509308	1.41224571377091e-117
ENSG00000091583	APOH	-2.42376961730595	9.26739755915626e-69
ENSG00000101049	SGK2	-2.42511384137158	3.44396703310822e-56
ENSG00000104611	SH2D4A	-2.42616109144239	4.23338998327825e-187
ENSG00000105137	SYDE1	-2.43034451706287	9.47545377688181e-55
ENSG00000231595	N.A.	-2.43036392644586	4.95604945186927e-05
ENSG00000169084	DHRSX	-2.43333000844111	4.23321171616292e-107
ENSG00000104998	IL27RA	-2.44066602589007	5.64011810762725e-22
ENSG00000035681	NSMAF	-2.44216632525725	9.5020292144239e-134
ENSG00000250067	YJEFN3	-2.44381338489079	1.35964101163531e-10

ENSG00000135318	NT5E	-2.44569608066035	0
ENSG00000019485	PRDM11	-2.44726332354005	1.07969176735231e-63
ENSG00000128268	MGAT3	-2.45203178982518	3.95543067151582e-06
ENSG00000266835	GAPLINC	-2.45349402213105	5.37119815781965e-07
ENSG00000019505	SYT13	-2.46097992711759	4.956188829562e-52
ENSG00000164403	SHROOM1	-2.47154020488371	3.40952200601063e-38
ENSG00000125864	BFSP1	-2.47368470492185	1.49233569386505e-17
ENSG00000139679	LPAR6	-2.47518939313141	1.85949329244124e-19
ENSG00000173193	PARP14	-2.47592370087509	2.38785562385324e-216
ENSG00000225361	PPP1R26-AS1	-2.47673831026301	0.00137481771600196
ENSG00000099377	HSD3B7	-2.48421000424259	3.77650852994508e-21
ENSG00000173209	AHSA2	-2.49249503130978	1.14085023588262e-192
ENSG00000267530	N.A.	-2.49444884350629	3.97718398752268e-16
ENSG00000165671	NSD1	-2.49667464170018	0
ENSG00000164362	TERT	-2.49990788686502	5.33877497909399e-14
ENSG00000187288	CIDEC	-2.50020736690864	3.79369268690785e-12
ENSG00000197122	SRC	-2.50519301188297	0
ENSG00000171631	P2RY6	-2.50546566086981	1.06433317869213e-49
ENSG00000138738	PRDM5	-2.50962633489575	1.93140018558579e-38
ENSG00000182057	OGFRP1	-2.51160000311894	0.00244224025332601
ENSG00000275649	N.A.	-2.51369166938463	0.00117654483856022
ENSG00000106714	CNTNAP3	-2.51458218497642	1.65334545328216e-169
ENSG00000076344	RGS11	-2.51570476534539	2.91766402145584e-08
ENSG00000133466	C1QTNF6	-2.51994567295424	4.76333319387e-44
ENSG00000234311	N.A.	-2.52421857851756	0.000225695986019263
ENSG00000165449	SLC16A9	-2.52446411993809	3.80632379571668e-11
ENSG00000003436	TFPI	-2.52598393952464	8.25002042384311e-251
ENSG00000278175	GLIDR	-2.52731246411512	2.58633132121933e-09
ENSG00000226562	CYP4F26P	-2.52850196391555	3.07698187441858e-06
ENSG00000135736	CCDC102A	-2.53448839370776	2.53101869379853e-35
ENSG00000139998	RAB15	-2.53648480932941	0
ENSG00000172159	FRMD3	-2.53663080939512	4.34336616778144e-17
ENSG00000142303	ADAMTS10	-2.53961154983844	3.88839270056091e-36
ENSG00000258181	N.A.	-2.53970814085972	0.000106657029807
ENSG00000242282	N.A.	-2.54105631817331	4.81259608364218e-12
ENSG00000171747	LGALS4	-2.54339121836336	9.52679243876567e-18
ENSG00000103044	HAS3	-2.54474034835268	1.7892941051017e-150
ENSG00000253428	N.A.	-2.54747736192924	1.76792250915213e-06
ENSG00000280239	N.A.	-2.54861825924869	7.2630576116251e-08
ENSG00000149948	HMGA2	-2.54997148296363	7.65707438953831e-138
ENSG00000180066	C10orf91	-2.55701428417938	5.48251704766707e-09
ENSG00000151474	FRMD4A	-2.56236791667919	5.10153565559913e-79
ENSG00000115221	ITGB6	-2.5632944826879	1.0553871613377e-06
ENSG00000109794	FAM149A	-2.56525145250946	4.60913928807672e-23
ENSG00000163735	CXCL5	-2.56742467144411	1.47139512162683e-114
ENSG00000272269	N.A.	-2.57154279282629	0.00523490362610779
ENSG00000215417	MIR17HG	-2.57181940140023	1.11127863129403e-09
ENSG00000198053	SIRPA	-2.57339296256562	5.34863332841145e-192
ENSG00000162989	KCNJ3	-2.57404663442888	7.39568656980009e-06

ENSG00000196074	SYCP2	-2.57915513989007	1.87947533802567e-27
ENSG00000096070	BRPF3	-2.57936868919564	5.83701253854284e-93
ENSG00000054983	GALC	-2.58035016918902	4.88197647595207e-138
ENSG00000163053	SLC16A14	-2.58305469980511	2.48317914066834e-29
ENSG00000101311	FERMT1	-2.58892090236012	7.06495924836268e-102
ENSG00000153404	PLEKHG4B	-2.59021472948596	4.72986566055885e-24
ENSG00000073849	ST6GAL1	-2.5956534544572	6.17612840827442e-38
ENSG00000139211	AMIGO2	-2.59639485774486	0
ENSG00000137273	FOXF2	-2.59821353464393	9.61730368914893e-14
ENSG00000171564	FGB	-2.6016153611088	0
ENSG00000165168	CYBB	-2.60348346980192	4.77309628815677e-06
ENSG00000267194	N.A.	-2.60806121881848	1.54356841047101e-42
ENSG00000255366	N.A.	-2.61618686000114	6.16641263395241e-06
ENSG00000162624	LHX8	-2.61747793729558	2.30040221594848e-59
ENSG00000162105	SHANK2	-2.61870625571763	8.99201117838484e-115
ENSG00000260963	N.A.	-2.62268267353235	2.45247543544651e-05
ENSG00000101096	NFATC2	-2.62660335224231	5.83284479706411e-29
ENSG00000019991	HGF	-2.63031211463923	1.06667271805748e-05
ENSG00000054793	ATP9A	-2.63096760992806	0
ENSG00000156966	B3GNT7	-2.63170844448126	9.44589794308071e-15
ENSG00000234264	DEPDC1-AS1	-2.63199992636855	0.00901484670127884
ENSG00000183770	FOXL2	-2.63953976625622	1.18205820325895e-06
ENSG00000128602	SMO	-2.6433276005669	7.57995679402353e-127
ENSG00000276386	CNTNAP3P2	-2.64606717778611	6.96071508703173e-14
ENSG00000116774	OLFML3	-2.64906930598964	0.000361875457941806
ENSG00000176153	GPX2	-2.65045634147358	0
ENSG00000087077	TRIP6	-2.65501694439159	4.39339299268532e-102
ENSG00000074410	CA12	-2.6571209262608	0
ENSG00000197724	PHF2	-2.66099200885951	6.19831803515413e-129
ENSG00000113361	CDH6	-2.66708878741451	1.26585198028663e-21
ENSG00000115594	IL1R1	-2.66784195433552	2.37890499290358e-149
ENSG00000023839	ABCC2	-2.6693373348091	0
ENSG00000119403	PHF19	-2.67015272530306	6.22725596339014e-172
ENSG00000188368	PRR19	-2.6760764534907	1.0300748278019e-07
ENSG00000110092	CCND1	-2.67667861351724	0
ENSG00000006756	ARSD	-2.67671811732402	3.6675535642177e-168
ENSG00000196814	MVB12B	-2.69185184556028	2.04260812762085e-64
ENSG00000198774	RASSF9	-2.69312964529134	8.46947061849145e-91
ENSG00000113273	ARSB	-2.6970600069663	4.28222921744174e-104
ENSG00000198121	LPAR1	-2.69784169312811	1.1654175670149e-81
ENSG00000198732	SMOC1	-2.71159991007575	1.07372322824373e-17
ENSG00000157657	ZNF618	-2.71504472870274	1.6428370262732e-264
ENSG00000214960	ISPD	-2.71512355863801	2.68669724124206e-08
ENSG00000259375	N.A.	-2.71700214730789	0.00161915477435655
ENSG00000015171	ZMYND11	-2.71885936429563	2.63790349231923e-296
ENSG00000267696	N.A.	-2.71987846774275	0.000785179444015609
ENSG00000178202	KDELC2	-2.72140207334148	5.00722742872292e-186
ENSG00000173175	ADCY5	-2.72235706335621	4.19710006931831e-87
ENSG00000168079	SCARA5	-2.72317716696721	7.12641814852332e-61

ENSG00000155115	GTF3C6	-2.72520552332784	1.45008499531 58e-180
ENSG00000206190	ATP10A	-2.7266086566071	7.52718482583331e-77
ENSG00000266235	MIR3176	-2.72845448660136	0.00332180758348922
ENSG00000141510	TP53	-2.73006876643496	2.92270307522714e-285
ENSG00000270246	N.A.	-2.73461560176088	1.14711053038883e-05
ENSG00000272173	N.A.	-2.74051855361208	1.25548979993414e-06
ENSG00000140682	TGFB1I1	-2.74860491276012	1.14774632035688e-57
ENSG00000175505	CLCF1	-2.749100962007	2.49651671317083e-50
ENSG00000169330	KIAA1024	-2.7542332936354	1.39359195325388e-12
ENSG00000101842	VSIG1	-2.76369500428105	2.23115809032058e-05
ENSG00000259674	N.A.	-2.77653789462629	1.96440826248628e-19
ENSG00000185186	LINC00313	-2.77957748442116	0.00559833906263559
ENSG00000236345	N.A.	-2.78318092296124	9.92776777257968e-05
ENSG00000160094	ZNF362	-2.78856905610217	1.02722906807662e-67
ENSG00000005108	THSD7A	-2.78902372903872	0
ENSG00000100557	C14orf105	-2.79398441178364	9.53804666708057e-101
ENSG00000214814	FER1L6	-2.79526018139541	0.000228199869540976
ENSG00000159899	NPR2	-2.79595804976332	2.05568803416761e-23
ENSG00000158089	GALNT14	-2.79873462691416	5.40047423316473e-109
ENSG00000165025	SYK	-2.79929370259187	3.86357020210647e-10
ENSG00000117226	GBP3	-2.8035132571537	2.89425398024557e-22
ENSG00000255031	N.A.	-2.80477641488106	7.00651908606932e-15
ENSG00000103154	NECAB2	-2.80699057569976	7.43791367728042e-29
ENSG00000186197	EDARADD	-2.81740317611589	1.70743091278342e-52
ENSG00000136161	RCBTB2	-2.81740467402001	2.09117893309869e-08
ENSG00000121310	ECHDC2	-2.82158295487762	2.88594604752477e-57
ENSG00000064655	EYA2	-2.82263662376922	9.87471848408215e-18
ENSG00000112186	CAP2	-2.82763997974268	7.91349323988667e-113
ENSG00000184937	WT1	-2.82827802367843	1.33675380630531e-23
ENSG00000268061	NAPA-AS1	-2.83129885434835	3.48159196285307e-05
ENSG00000174502	SLC26A9	-2.86081789245746	1.33633238640347e-14
ENSG00000115648	MLPH	-2.8643131427298	0
ENSG00000132744	ACY3	-2.87032560533607	1.67236227549026e-19
ENSG00000234155	N.A.	-2.87496970526682	3.65109156408053e-13
ENSG00000189431	RASSF10	-2.87962221557347	1.07133811998081e-104
ENSG00000121281	ADCY7	-2.88036598221645	2.71588591413516e-130
ENSG00000041353	RAB27B	-2.88129228908438	2.18148963256019e-246
ENSG00000171984	C20orf196	-2.88149957700156	2.55006960307917e-30
ENSG00000164488	DACT2	-2.88195470490078	5.96495831744126e-13
ENSG00000231160	KLF3-AS1	-2.88200247338998	1.78168098951727e-05
ENSG00000124564	SLC17A3	-2.89011323082677	0.000899138173896126
ENSG00000261884	N.A.	-2.89242724466477	0.00088478870744725
ENSG00000143772	ITPKB	-2.89906908671407	7.83495930737957e-47
ENSG00000153956	CACNA2D1	-2.90365378138634	5.50828864208857e-264
ENSG00000163083	INHBB	-2.9048213535215	1.29807033611477e-239
ENSG00000188850	N.A.	-2.91176658541629	0.00797697818921611
ENSG00000166823	MESP1	-2.91310830277513	7.50760952045595e-14
ENSG00000004848	ARX	-2.91346245202037	9.3760250589517e-10
ENSG00000280018	N.A.	-2.9214678857416	8.83449039204173e-17

ENSG00000123609	NMI	-2.92659011218034	3.87483191036153e-49
ENSG00000113231	PDE8B	-2.93077209560899	5.61671564879305e-105
ENSG00000159231	CBR3	-2.9360079738992	1.53197767893742e-25
ENSG00000174808	BTC	-2.93614514458793	2.34853401977050e-15
ENSG00000260037	N.A.	-2.93693578397578	0.00726246471861141
ENSG00000091262	ABCC6	-2.96787455263256	1.64817177613189e-10
ENSG00000138172	CALHM2	-2.96956516448913	2.136517935774e-20
ENSG00000267414	N.A.	-2.97800476979729	0.00111366601347594
ENSG00000173917	HOXB2	-2.97970415057138	5.7893335338981e-09
ENSG00000167711	SERPINF2	-2.98059231694498	2.67658496660003e-37
ENSG00000010704	HFE	-2.98223365403916	5.16095917860353e-183
ENSG00000129514	FOXA1	-2.98418816736734	2.00186222028037e-208
ENSG00000164626	KCNK5	-2.98512523305167	2.27170899265262e-115
ENSG00000244509	APOBEC3C	-2.98879193935399	1.73447569775657e-140
ENSG00000016391	CHDH	-2.99569435193889	2.38712623293248e-76
ENSG00000116771	AGMAT	-3.00033011659636	4.36631362908052e-47
ENSG00000158163	DZIP1L	-3.00345136323969	5.6071621572119e-22
ENSG00000215440	NPEPL1	-3.00902662188389	1.05552556623383e-32
ENSG00000026508	CD44	-3.01170563499486	0
ENSG00000154027	AK5	-3.01293508800567	1.40575867641972e-11
ENSG00000268357	VN1R81P	-3.01651230054406	0.000654892087359582
ENSG00000172164	SNTB1	-3.01918083480705	9.42318041752615e-90
ENSG00000226812	N.A.	-3.02444992168015	0.0019418770438293
ENSG00000135905	DOCK10	-3.03109340140271	1.45586176150734e-158
ENSG00000169933	FRMPD4	-3.03507280749383	0.00351918007458368
ENSG00000126262	FFAR2	-3.03764465305055	0.00499945749219473
ENSG00000104760	FGL1	-3.04022329052384	0
ENSG00000265962	GACAT2	-3.04270451980835	3.30594682055318e-100
ENSG00000223764	N.A.	-3.04404849160116	3.43274873875733e-33
ENSG00000259176	N.A.	-3.05056905455293	0.00178815865909725
ENSG00000186529	CYP4F3	-3.05199898137241	8.79386969637423e-170
ENSG00000182836	PLCXD3	-3.05445459143452	8.49161955201074e-203
ENSG00000205488	CALML3-AS1	-3.05605871747408	0.00775699618798599
ENSG00000144063	MALL	-3.05739977651436	0
ENSG00000147408	CSGALNACT1	-3.05861003924589	0
ENSG00000154529	CNTNAP3B	-3.06280003950369	0
ENSG00000179776	CDH5	-3.06443639177193	5.13482435797878e-07
ENSG00000160783	PMF1	-3.0688721895616	1.99715111559522e-58
ENSG00000157193	LRP8	-3.07109937326965	0
ENSG00000251138	N.A.	-3.08131809879292	5.87940855185903e-06
ENSG00000005844	ITGAL	-3.08316424785085	1.49124142020445e-06
ENSG00000139182	CLSTN3	-3.09107821702168	2.84607376660414e-147
ENSG00000249395	CASC9	-3.09336389418557	1.97298076590843e-20
ENSG00000139173	TMEM117	-3.09407509322109	7.49841411394117e-30
ENSG00000198788	MUC2	-3.09419363216758	0.00731563659895451
ENSG00000006534	ALDH3B1	-3.09485043851024	0
ENSG00000204128	C2orf72	-3.10324292967073	2.12743925661084e-96
ENSG00000157388	CACNA1D	-3.10799455844667	3.05628042926945e-79
ENSG00000170382	LRRN2	-3.10931755919237	7.62028374503075e-58

ENSG00000229656	N.A.	-3.1143916338309	8.17962214511979e-34
ENSG00000128342	LIF	-3.11828359676297	9.2228498456506e-123
ENSG00000152049	KCNE4	-3.12590403775101	7.15204951442704e-50
ENSG00000204528	PSORS1C3	-3.12755673599283	0.00030621758527149
ENSG00000134363	FST	-3.14540068134266	9.44562132203906e-69
ENSG00000158234	FAIM	-3.14829704177863	5.37191041097617e-49
ENSG00000148704	VAX1	-3.16466364264006	1.48724219357834e-08
ENSG00000205885	C1RL-AS1	-3.17038322360489	1.19029493823615e-75
ENSG00000103490	PYCARD	-3.17483608790302	2.7358131570938e-20
ENSG00000107829	FBXW4	-3.18840388978698	7.03864037621472e-143
ENSG00000137672	TRPC6	-3.18899756637425	2.8134803066808e-93
ENSG00000078401	EDN1	-3.1997717805917	4.12801345803347e-291
ENSG00000140675	SLC5A2	-3.2083652742412	0.00424040313261523
ENSG00000108379	WNT3	-3.21694518556008	1.96166878866917e-66
ENSG00000130038	CRACR2A	-3.21766057788732	1.29116459788775e-20
ENSG00000179546	HTR1D	-3.22005077945881	1.63279576771953e-77
ENSG00000167363	FN3K	-3.22134829422428	2.24593730595839e-41
ENSG00000164855	TMEM184A	-3.23533848812308	2.35259884673936e-159
ENSG00000188761	BCL2L15	-3.24131500379969	1.46730379744382e-30
ENSG00000256940	N.A.	-3.24329662254783	9.85234292509612e-11
ENSG00000099812	MISP	-3.24396269169438	0
ENSG00000163762	TM4SF18	-3.2464001730625	2.20529198868317e-251
ENSG00000254231	N.A.	-3.24838433680993	0.000192347185434527
ENSG00000140479	PCSK6	-3.25519203520328	1.45330439454195e-94
ENSG00000071991	CDH19	-3.25658405554304	8.45432145679456e-13
ENSG00000155974	GRIP1	-3.26178478608851	2.599035231247e-40
ENSG00000249199	N.A.	-3.27257751845544	8.99546402494846e-09
ENSG00000267015	N.A.	-3.27505117222964	6.41125164157569e-11
ENSG00000263368	N.A.	-3.28674908927674	0.00450145159930094
ENSG00000009709	PAX7	-3.30477847891864	4.57772777148942e-07
ENSG00000151617	EDNRA	-3.30573974441347	5.56700976156763e-16
ENSG00000187634	SAMD11	-3.30696495829579	6.13366040974865e-163
ENSG00000267374	N.A.	-3.31056312134033	2.90784903851728e-16
ENSG00000116663	FBXO6	-3.31741511051887	5.94227303788681e-34
ENSG00000132561	MATN2	-3.32319139503385	2.13810435087041e-99
ENSG00000159958	TNFRSF13C	-3.32388127273318	0.00172872098554799
ENSG00000150594	ADRA2A	-3.32440809546714	5.41912032397149e-08
ENSG00000237476	N.A.	-3.33081744278904	0.00203250497154072
ENSG00000059377	TBXAS1	-3.3313762420888	2.7735009638856e-54
ENSG00000139865	TTC6	-3.33275381566878	6.54122768380463e-14
ENSG00000049540	ELN	-3.33398348151562	3.30705807347203e-24
ENSG00000108187	PBLD	-3.33767042860321	2.49332250193927e-66
ENSG00000169857	AVEN	-3.33997336583088	9.17402024427648e-74
ENSG00000169903	TM4SF4	-3.34085305059471	1.48138713508272e-75
ENSG00000165246	NLGN4Y	-3.35277200911955	4.78808339859771e-29
ENSG00000223855	N.A.	-3.3552806230778	3.05580293909836e-05
ENSG00000267288	N.A.	-3.36460728270703	3.73388356610906e-16
ENSG00000049449	RCN1	-3.37897676572646	0
ENSG00000176890	TYMS	-3.38604960122385	0

ENSG00000260400	N.A.	-3.38831856694748	0.00192538791260849
ENSG00000099284	H2AFY2	-3.38964951172952	2.55927404597009e-100
ENSG00000227471	AKR1B15	-3.39429221666657	7.68091665595813e-123
ENSG00000100219	XBP1	-3.39810627868721	0
ENSG00000178726	THBD	-3.41604041680496	7.60674446812432e-123
ENSG00000214455	RCN1P2	-3.42698040526907	1.46120186170687e-11
ENSG00000228109	MFI2-AS1	-3.44131677300618	6.33487716208045e-10
ENSG00000047457	CP	-3.44191224935581	2.69145356598691e-165
ENSG00000115935	WIPF1	-3.44388473193248	3.27196372772535e-161
ENSG00000138079	SLC3A1	-3.44487046856616	2.38040359616505e-92
ENSG00000267121	N.A.	-3.44776565529198	2.67604859775922e-11
ENSG00000248807	KRTAP9-12P	-3.49456757660515	9.30839677566162e-13
ENSG00000080493	SLC4A4	-3.510253847056	3.43922451785007e-171
ENSG00000185332	TMEM105	-3.52622911403591	2.44127781168293e-08
ENSG00000183773	AIFM3	-3.52644368482635	5.49499123412268e-12
ENSG00000146477	SLC22A3	-3.54866298409521	0
ENSG00000141668	CBLN2	-3.55475075543399	1.4630222677181e-14
ENSG00000078399	HOXA9	-3.55598517946866	1.21059444997342e-05
ENSG00000196620	UGT2B15	-3.55908419375515	0.00651486572256415
ENSG00000258498	DIO3OS	-3.55978574216526	0.000770168089293577
ENSG00000144057	ST6GAL2	-3.57171756506711	2.75735968449957e-21
ENSG00000261713	SSTR5-AS1	-3.6032440078935	0.00729404368356088
ENSG00000170624	SGCD	-3.61365640289312	2.26458201049423e-99
ENSG00000135324	MRAP2	-3.62371936898318	4.37465836823065e-36
ENSG00000129354	AP1M2	-3.63268307980391	3.60117278138718e-253
ENSG00000237517	DGCR5	-3.64104666904248	1.51870644923188e-40
ENSG00000120211	INSL4	-3.64229048767111	0
ENSG00000137731	FXYD2	-3.64603749576258	2.77176624679222e-15
ENSG00000278035	N.A.	-3.66009083254343	0.000589458627627528
ENSG00000158315	RHBDL2	-3.67423008576065	2.08863707349449e-21
ENSG00000211448	DIO2	-3.67665899197482	1.32845197118704e-18
ENSG00000161103	N.A.	-3.69161570948088	0.00253799420100973
ENSG00000174827	PDZK1	-3.70672493399082	3.03872547651537e-20
ENSG00000157399	ARSE	-3.70673249413966	3.94597721483700e-243
ENSG00000136371	MTHFS	-3.72260128421588	4.49327233914717e-10
ENSG00000280202	N.A.	-3.73914360424777	9.65765003466279e-06
ENSG00000219755	N.A.	-3.75402207779064	0.00391718667312385
ENSG00000133519	ZDHHC8P1	-3.75461837485545	8.10520526356178e-29
ENSG00000146678	IGFBP1	-3.75914939782922	3.34666823787671e-19
ENSG00000220785	MTMR9LP	-3.76065935484536	4.56661947247388e-16
ENSG00000125999	BPIFB1	-3.77332285269493	2.53907520198452e-13
ENSG00000108846	ABCC3	-3.78254389188657	0
ENSG00000187824	TMEM220	-3.80191513873194	4.51338754118239e-13
ENSG00000254827	SLC22A18AS	-3.81648264187206	1.43786192617048e-27
ENSG00000160180	TFF3	-3.81792767157296	6.03740197322253e-10
ENSG00000166126	AMN	-3.82183372278631	0.00016529691850926
ENSG00000104361	NIPAL2	-3.83051811525572	1.52716985968519e-70
ENSG00000205220	PSMB10	-3.84557218688427	1.7332694716998e-13
ENSG00000259439	N.A.	-3.84631559653606	1.56014640301839e-26

ENSG00000259408	N.A.	-3.84746070636583	0.00225110572443534
ENSG00000064787	BCAS1	-3.85844814366948	2.05654026931745e-195
ENSG00000135697	BCO1	-3.88011751540273	5.88922966821336e-57
ENSG00000128298	BAIAP2L2	-3.88331863993854	1.62427053725578e-63
ENSG00000183032	SLC25A21	-3.89682339789832	8.75303863344481e-05
ENSG00000134538	SLCO1B1	-3.90432502671742	0.000120685441634783
ENSG00000227039	ITGB2-AS1	-3.92313144489445	2.14668041021355e-05
ENSG00000070731	ST6GALNAC2	-3.9274916331802	1.30835596175876e-18
ENSG00000168955	TM4SF20	-3.9739614185433	3.39923329640241e-36
ENSG00000135914	HTR2B	-3.97651876817682	0.00256528572278966
ENSG00000232324	N.A.	-4.00075019136464	5.11323679362192e-13
ENSG00000135100	HNF1A	-4.04176484368187	1.16754668539291e-129
ENSG00000170837	GPR27	-4.0444759012913	2.50845908392689e-11
ENSG00000231752	EMBP1	-4.05108078713977	1.510507698296e-06
ENSG00000198074	AKR1B10	-4.07433772430421	0
ENSG00000207547	MIR25	-4.08955865336984	0.000914394307181369
ENSG00000277196	N.A.	-4.09124478393334	3.34789181605698e-30
ENSG00000234928	N.A.	-4.09757662033073	0.000891229644343518
ENSG00000232306	N.A.	-4.10142222690445	0.000826779446880159
ENSG00000125966	MMP24	-4.10310447278696	0
ENSG00000136881	BAAT	-4.12239235794547	2.80000542186845e-23
ENSG00000279970	N.A.	-4.1680264845851	0.000331870932283897
ENSG00000225614	ZNF469	-4.19667685191498	8.72021809738972e-44
ENSG00000100033	PRODH	-4.20081666476106	1.52003271523189e-77
ENSG00000057593	F7	-4.20132980625494	2.11146021557945e-05
ENSG00000181982	CCDC149	-4.21123176465914	1.11443526531864e-23
ENSG00000249599	BMPR1B-AS1	-4.22271363990055	2.32590766977217e-11
ENSG00000100314	CABP7	-4.23742290175893	1.74064415803847e-05
ENSG00000104537	ANXA13	-4.2735487187814	5.53386439415301e-94
ENSG00000224417	N.A.	-4.27494294797904	1.90259783065969e-14
ENSG00000177706	FAM20C	-4.31955557361847	2.32426769570624e-160
ENSG00000257743	MGAM2	-4.32507149137383	3.07300137652806e-34
ENSG00000180525	PRR26	-4.35646107968422	4.6283113321183e-31
ENSG00000203666	EFCAB2	-4.36623333322765	8.30058909538239e-25
ENSG00000142149	HUNK	-4.36997989669998	1.53390590788641e-53
ENSG00000169507	SLC38A11	-4.37043787442692	0.000219195383418194
ENSG00000215182	MUC5AC	-4.38998121081487	1.19116359192494e-265
ENSG00000179023	KLHDC7A	-4.40897140497803	1.00625918794159e-76
ENSG00000259974	LINC00261	-4.42840666476905	1.27229516374948e-36
ENSG00000117983	MUC5B	-4.44500172030829	3.25558605694938e-284
ENSG00000249430	N.A.	-4.4705230093276	0.000107331001391506
ENSG00000100079	LGALS2	-4.4881843581062	2.00576015479947e-09
ENSG00000257335	MGAM	-4.49026428104346	1.07386530990161e-208
ENSG00000170482	SLC23A1	-4.49933103070938	1.4385761041233e-32
ENSG00000120054	CPN1	-4.51340858344335	2.42519634279715e-26
ENSG00000278937	N.A.	-4.5606127665342	0.00892134519203077
ENSG00000232654	FAM136BP	-4.59627276109523	0.00880723647590104
ENSG00000162878	PKDCC	-4.62079857059039	0
ENSG00000259605	N.A.	-4.64565961768086	0.00548279013138004

ENSG00000164749	HNF4G	-4.68375137289894	2.45735467147722e-70
ENSG00000175311	ANKS4B	-4.68961257610638	1.30842591421576e-106
ENSG00000130433	CACNG6	-4.71357085895761	4.17784861418674e-51
ENSG00000148702	HABP2	-4.73545993455231	2.04438182474647e-118
ENSG00000153885	KCTD15	-4.74847950516703	7.58154207796254e-259
ENSG00000164761	TNFRSF11B	-4.7777868457919	8.54438617941208e-14
ENSG00000183145	RIPPLY3	-4.86499294623511	9.77621538672982e-14
ENSG00000244161	FLNB-AS1	-4.8689174886602	3.1100771330901e-05
ENSG00000188107	EYS	-4.90531952210963	0.00405607560505662
ENSG00000169562	GJB1	-4.93494014017484	0.00335240236075448
ENSG00000278192	N.A.	-4.99360387712316	0.00288354251074528
ENSG00000176046	NUPR1	-5.06587393327248	9.06938710140575e-43
ENSG00000162975	KCNF1	-5.09380292030893	1.14333117046999e-10
ENSG00000181143	MUC16	-5.14888128421194	0
ENSG00000167281	RBFOX3	-5.18702998255523	1.02423272802332e-31
ENSG00000226674	TEX41	-5.28888558470563	0.00167098612138733
ENSG00000080618	CPB2	-5.32599927906864	0.000752009051307488
ENSG00000203635	N.A.	-5.32914811141828	0.0013074709734468
ENSG00000189045	ANKDD1B	-5.35074858569469	0.00100725250777307
ENSG00000172367	PDZD3	-5.3746022487841	3.84910898687695e-09
ENSG00000258867	LINC01146	-5.43132924676679	0.000757309058429485
ENSG00000198758	EPS8L3	-5.4575304126167	3.05458991013572e-30
ENSG00000113924	HGD	-5.52257009408909	2.85201268019783e-234
ENSG00000277297	ATP5A1P10	-5.58488186360251	0.000846067720150959
ENSG00000170477	KRT4	-5.68242477482055	0
ENSG00000188959	C9orf152	-5.6891235231343	1.3962714348548e-38
ENSG00000175920	DOK7	-5.89926208580102	8.1259412857133e-14
ENSG00000248771	LINC01207	-6.00955409227164	5.23671168478242e-18
ENSG00000241388	HNF1A-AS1	-6.02355018601161	1.44720842349064e-35
ENSG00000123201	GUCY1B2	-6.08271481541703	2.71047333837551e-08
ENSG00000255774	N.A.	-6.10616614663217	2.43709604789374e-15
ENSG00000235142	N.A.	-6.16979596447282	0.000111382760407622
ENSG00000145920	CPLX2	-6.87186243974087	0
ENSG00000135960	EDAR	-6.93503533206772	7.5908060799287e-06
ENSG00000124664	SPDEF	-6.98127398541948	9.70239615455802e-135
ENSG00000162383	SLC1A7	-7.78650733479796	0.00935215503766686
ENSG00000259456	ADNP-AS1	-7.99379514256039	0.00643732817947389
ENSG00000169994	MYO7B	-9.48724723447417	0.000885693500534825
